# Genome-wide identification and functional characterization of magnesium transporter (MGT) gene family in soybean (*Glycine max* L.) and their expression profiles in response to aphid infestation, dehydration, and salt stresses

**DOI:** 10.1371/journal.pone.0330440

**Published:** 2025-08-29

**Authors:** Nasrin Akter, Md Shohel Ul Islam, Fatema Tuz Zohra, Raihan Ur Rahman Ratno, Md. Shahedur Rahman, Shaikh Mizanur Rahman, Md. Abdur Rauf Sarkar

**Affiliations:** 1 Department of Genetic Engineering and Biotechnology, Faculty of Biological Science and Technology, Jashore University of Science and Technology, Jashore, Bangladesh; 2 Department of Genetic Engineering and Biotechnology, Faculty of Biological Sciences, University of Rajshahi, Rajshahi, Bangladesh; Nuclear Science and Technology Research Institute, IRAN, ISLAMIC REPUBLIC OF

## Abstract

The divalent cation, Magnesium (Mg2+), is an essential mineral element for plant growth and development. Magnesium transporter (MGT) plays a vital role in maintaining Mg2 + homeostasis within plant cells. Although extensive research has been conducted in several crop species, no comprehensive study has yet been carried out on the MGT gene family in soybean (*Glycine max* L.), an economically valuable oil crop species. In this study, a total of 29 *MGT* genes encoding proteins (GmMGT) were identified in the soybean genome through comprehensive bioinformatics analysis. The GmMGT proteins were subsequently categorized into MRS2, CorA, and NIPA groups, with the majority predicted to be localized to the plasma membrane. Analyses of gene structures, conserved domains, and motifs indicated strong structural and functional similarities across the subgroups. Gene duplication, selection pressure, and synteny analyses demonstrated that *GmMGT* genes had undergone purifying selection, with only 12 segmentally duplicated gene pairs being identified. Gene ontology (GO) analysis revealed the involvement of all *GmMGT* genes in organism localization and ion transportation. *Cis*-acting regulatory element (CAREs) analysis identified 53 CAREs involved in light responsiveness, tissue-specific, phytohormone, and stress responses. Notably, nine major CAREs were abundantly found in the promoter regions of *GLYMA.06G159100* and *GLYMA.10G180200*. Through the promoter analysis, we identified 81 miRNAs and 29 transcription factor families (TFFs), overrepresented under different stress conditions. RNA-seq data from 14 different soybean tissues demonstrated higher expression in flower tissue and lower levels in nodules with *GLYMA.05G168200*, *GLYMA.10G180200*, *GLYMA.12G030100*, *GLYMA.12G168000*, *GLYMA.16G003900*, *GLYMA.20G210300* exhibiting elevated expression patterns. Transcriptomic analysis further revealed that, 10 *GmMGTs* were upregulated under biotic stress at 4h, 8h, 24h, and 48h after aphid infestation, with *GLYMA.02G285600* and *GLYMA.13G368400* being the most upregulated genes. Under abiotic stresses, *GLYMA.03G159400, GLYMA.05G196600*, and *GLYMA.15G125900* were upregulated in response to dehydration, while *GLYMA.04G005200*, *GLYMA.08G126600*, *GLYMA.10G180200* were induced at 1h, 6h, and 12h under salinity stress. These findings shed light on the versatile roles of *GmMGT* genes in plant growth, development and stress response, and they may serve as a valuable resource for further functional characterization of *GmMGT* genes within the soybean genome.

## 1. Introduction

Magnesium (Mg2+) plays significant roles in plant developmental processes, including nucleic acid synthesis, protein synthesis, membrane stability, catalytic activity, and photosynthesis. It activates more than 300 enzymes [1,2]. Despite being an essential nutrient, recent research has revealed reduced Mg2 + concentrations in soil used for cereal crop cultivation [3]. Heavy rainfall can result in acidic soils saturated with Ca2 + , Mn2 + , Al2 + , K + , and H + , reducing Mg2 + availability for plant roots [4]. Increasing Mg2 + might significantly mitigate the detrimental effects of these ions. Although Mg2 + performs essential functions in plants, excessive concentrations can also be harmful [5]. In plants, Mg channels regulate Mg2 + concentration in the tonoplast and facilitate accumulation in the mesophyll tissues through vacuoles [1]. Indeed, MGTs, comprising NIPA, CorA, and MRS2 groups of proteins, are essential for maintaining Mg2 + homeostasis in plants [6,7]. The secondary structure pattern in NIPA, Cor-A, and MRS2 proteins is highly similar and includes one acidic N-terminal periplasmic domain and two C-terminal transmembrane (TM) domains [8–12]. A conserved GMN tripeptide motif comprising the Gly-Met-Asn residues has been identified in the hydrophobic portion of the protein, which is expected to represent the catalytic region of the Mg2 + transporter [13]. However, slight sequence modifications were observed in maize [14] and rice [15]. Mutation analyses revealed that any specific amino acid alteration within the GMN motif leads to a loss of Mg2 + transport functionality by the MGT protein [15].

The *MGT* gene family has been extensively studied in various plant species, including arabidopsis (*Arabidopsis thaliana*) [16], maize (*Zea mays*) [14], rice (*Oryza sativa* L.) [15], sugarcane (*Saccharum officinarum*) [17], tomato (*Solanum lycopersicum* L.) [18], banana (*Musa paradisiaca* Linn.) [19], and pear (*Pyrus communis*) [20]. MGT proteins have primarily been found in arabidopsis among plant species, followed by rice [15,16]. Beyond sequence variations, different *MGT* genes in plants have shown diverse expression patterns. For example, in arabidopsis, *AtMGT1* is responsible for Mg2 + absorption [16], while *AtMGT4*, *AtMGT5*, and *AtMGT9* play role in pollen formation [21,22]. *AtMGT10* is involved in Mg2 + transport within the chloroplast [23], and *AtMGT2* and *AtMGT3* regulate vacuolar Mg2 + levels [24]. In rice, *OsMGT1* is responsible for Mg2 + uptake by roots [25]. *ZmMGT12* in maize has been identified as a circadian rhythmic transporter responsive to light [26], while *ZmMGT10* facilitates Mg2 + absorption from soil [27]. In *Dendrobium officinale*, *DoMGT1* is expressed in root, leaf, and steam tissues [28]. *MGTs* enhance plant endurance to environmental stresses. For instance, *OsMGT1* contributes to salt stress and aluminum stress tolerance in rice [25]. The diversity in expression profiles among *MGT* genes reflects their functional complexity in plants.

Soybean (*G. max* L.) is one of the most economically valuable legumes, native to East Asia, and a significant source of oil (16–21%) and protein (37–48%) for both animal and human consumption. Soybeans are also rich in nutrients, minerals, and other beneficial compounds, including isoflavones, which provide multiple health benefits such as preventing age-related disorders, heart diseases, cancer, and osteoporosis [29]. However, soybean productivity, growth, and seed quality can be adversely affected by various environmental factors, such as drought, cold, salinity, toxic metals, and nutrient deficiencies, causing yield loss of up to 70% [30]. Therefore, developing stress-resistant soybean varieties is essential for ensuring future food security. Modern soybean varieties require effective nutritional management and Mg2 + is one of the most critical macronutrients for soybean seed development, productivity, and oil production [31].

To date, the comprehensive study of soybean (*G. max* L.) *MGT* gene family and their expression analysis under biotic and abiotic stress conditions remain unknown. The availability of the soybean (William 82) genome sequence has provided an opportunity to identify and characterize important gene families [32]. However, identification and functional characterization of targeted gene family members in wet lab conditions are challenging due to limitations in research grants, laboratory facilities, technical expertise, time constraints, and ethical considerations. Thus, comprehensive bioinformatics approaches were conducted to identify and characterize the *MGT* genes in the soybean genome. These analyses included phylogenetic analysis, gene structure, domain and motif analysis, Ka/Ks analysis, collinearity analysis, synteny analysis, chromosomal distribution analysis, duplication analysis, subcellular localization analysis, CARE analysis, GO analysis, transcription factor (TF) analysis, RNA-seq analysis of different tissues and expression analysis under various biotic and abiotic stresses. Our findings may provide valuable insights into the detailed functional roles of soybean *MGT* genes and uncover potential strategies to address Mg2 + deficiency and enhance soybean seed production efficiency.

## 2. Materials and methods

### 2.1. Database search and retrieval of MGT protein sequences in the soybean genome

The MGT DNA-binding domains of arabidopsis (*A. thaliana*)*,* rice (*O. sativa* L.)*,* and chickpea (*Cicer arietinum*) were initially utilized to retrieve MGT gene-encoded proteins from the *G. max* L. genome via Phytozome v13 (https://phytozome-next.jgi.doe.gov/), using BLAST-P (Protein-basic local alignment search tool) [33]. The Hidden Markov Model (HMM) profiles for the Mg transporter domains, PF05653 and PF01544, were obtained from the Pfam database [34]. These HMM profiles were subsequently used to screen the soybean proteomes through the HMMER package v3.0. Proteins lacking the MGT conserved domain (PF05653 and PF01544) were removed from the candidate list (https://pfam.xfam.org/). The retrieved amino acid sequences were then analyzed for conserved MGT domains using the SMART (Simple Modular Architecture Research Tool, http://smart.embl-heidelberg.de/) [35] and the NCBI CDD (Conserved Domain Database) (http://www.ncbi.nlm.nih.gov/Structure/cdd/wrpsb.cgi/) [36].

### 2.2 Determination of physicochemical properties of GmMGT proteins

The ProtParam online tool (http://web.expasy.org/protparam/) [37] was used to predict basic physicochemical properties, including amino acid residues (aa), molecular weight (MW), isoelectric point (pI), instability index, aliphatic index, and Grand Average of Hydropathicity (GRAVY) of MGT proteins.

### 2.3. Phylogenetic analysis of MGT proteins of arabidopsis, rice, soybean, and chickpea

MGT protein sequences from *A. thaliana*, *O. sativa* L., *G. max* L.*,* and *C. arietinum* (S1 Data) were retrieved from Phytozome v13 (https://phytozome.jgi.doe.gov/pz/portal.html/) and a phylogenetic tree was generated with MEGA11 software [38]. The ClustalW program was used to align the sequence with the Maximum Likelihood (ML) technique with default parameters [39], except for a 1000 bootstrap value and Pearson correction. The constructed tree was visualized with the Chiplot online tool (https://www.chiplot.online/).

### 2.4. Gene structure analysis

The coding sequences (CDS), genomic DNA sequences in FASTA format (S2 Data and S3 Data), and soybean gf3 files were obtained from Phytozome and submitted to the online tool Gene Structure Display Server, GSDS v2.0 (http://gsds.gao-lab.org/) [40] to evaluate the gene structure of *MGTs*.

### 2.5. Conserved domain and motif analysis

Conserved MGT domains were identified using the Pfam database (http://pfam.xfam.org), and the results were visualized using the TBtools software version-v2.0 [41]. The structural motifs of GmMGT protein sequences (S4 Data) were investigated using the Multiple EM for Motif Elicitation (MEME) (http://meme.nbcr.net/meme/), with default parameters and a fixed number of 20 motifs [42]. The motifs were visualized using MEME and the motif scanning method (MSA), available through its interface.

### 2.6. Gene duplication analysis and synonymous (Ks) and non-synonymous (Ka) substitution ratios calculation

The Ka/Ks Calculation tool (http://services.cbu.uib.no/tools/kaks) was used to estimate the substitution ratios of the soybean *MGT* gene family using duplicated MGT CDS sequences. The rates of molecular evolution were predicted using the Ka/Ks ratios for each pair of paralogous genes. The period of duplication and divergence (millions of years ago/MYA) was estimated using a synonymous mutation rate of substitutions per synonymous site per year as T = Ks/2λ × 10^−6^ (λ = 6.5 × 10^−9^) [43].

### 2.7. Collinearity and synteny analysis

To confirm gene duplication, collinearity and synteny analyses were conducted by using the Plant Genome Duplication Database (http://chibba.agtec.uga.edu/duplication/index/locus) [44]. Furthermore, TBtools version-v2.0 was used to illustrate the obtained soybean *MGT* collinear and syntenic pairs with arabidopsis*,* rice, and chickpea. The Circos program (http://circos.ca/) was used to illustrate the figures for collinearity and synteny analyses [45].

### 2.8. Analysis of chromosomal location

Chromosomal length and location of the 29 *GmMGT* genes were obtained from the Phytozome v13 database. Their genomic locations were mapped using MG2C (http://mg2c.iask.in/mg2c_v2.0/) [46].

### 2.9. Prediction of the subcellular localization and Gene Ontology (GO) analyses of *GmMGTs*

The subcellular localization of GmMGT proteins was predicted using the Wolf PSORT program (https://wolfpsort.hgc.jp/) [47]. To identify the functional relationships of the soybean *MGT* genes with various biological processes and molecular functional pathways, GO analysis was performed using the Plant Transcription Factor Database (PlantTFDB), PlantTFDB 4.0 (http://planttfdb.cbi.pku.edu.cn//) [48] and the Chiplot online tool (https://www.chiplot.online/) was used to visualize the analyzed data.

### 2.10. *Cis*-acting regulatory elements (CAREs) analysis of soybean *MGT* gene promoters

A 2000 bp sequences from 5′ untranslated region (5′ UTR) of *GmMGT* genomic sequences were extracted from the Phytozome v13. The *cis*-elements were analyzed using the PlantCARE online tool (http://bioinformatics.psb.ugent.be/webtools/plantcare/html/) [49] and verified in the PLACE databases (http://www.dna.afrc.go.jp/PLACE/) [50]. The obtained data were visualized using the R program (version R-4.2.1) [51].

### 2.11. Putative microRNA target site analysis

Soybean micro-RNA (miRNA) datasets were obtained from the plant microRNA encyclopedia (http://pmiren.com/) [52]. To identify miRNAs potentially targeting *GmMGTs*, the CDS sequences were examined for sequences complementary to miRNAs using the default parameters of psRNATarget (https://plantgrn.noble.org/psRNATarget/analysis?function=3) [53].

### 2.12. Transcription factor (TF) analysis of *GmMGTs*

To identify the important TFs associated with the identified *GmMGT* genes, PlantTFDB 4.0 was used. A regulatory network was constructed illustrating the interaction between *MGT* genes and predicted TFs using Cytoscape 3.9.1 [54].

### 2.13. Expression pattern analysis of *GmMGT* genes in different tissues

RNA-seq expression data for the identified *MGT* genes in various tissues were obtained from the RNA-Seq Atlas on SoyBase (https://www.soybase.org/) [55], and the BAR database (https://bar.utoronto.ca/). RPKM (reads per kilobase million) values on a log2 transformed scale were used to represent the expression value. The retrieved data were visualized using the R program (version R-4.2.1).

### 2.14. Transcriptomic analysis of *GmMGT* genes in response to soybean aphid infestation

Previously generated transcriptomic data in response to aphid infestation between susceptible and resistant soybean cultivars at five different sampling time points: 0h (no aphids), 4h, 8h, 24h, and 48h after aphid infestation were analyzed to study the transcriptomic profiling of identified *GmMGT* genes [56]. The fragments per kilobase million (FPKM) values were transformed to log2 format and compared with the control. The heatmap of transcriptomic data was visualized using the R program (version R-4.2.1).

### 2.15. Transcriptomic analysis of *GmMGT* genes under dehydration and salt stress

Transcriptomic data of *GmMGTs* in response to abiotic stresses (drought and salt) at three different time points (1h, 6h, and 12h) with control (0h) were used to analyze the specific expression patterns of *GmMGT* genes [57]. The FPKM values were transformed to log2 format and compared with the control. Heatmaps were generated using the R program (version R-4.2.1).

## 3. Results and discussions

Subsections 3.1 to 3.4 represent the general characterization of the studied genes; subsections 3.5 and 3.6 emphasize their evolutionary aspects; subsections 3.7 and 3.8 provide the information on gene localization; subsections 3.9 to 3.12 highlight their functional characteristics; and subsections 3.13 to 3.15 describe their RNA-Seq expression profiles.

### 3.1. Identification of GmMGT proteins and determination of their physicochemical properties

In this study, a total of 29 *MGT* gene-encoded proteins of soybean (*G. max* L.) were identified through genome-wide analysis using MGT protein sequences of arabidopsis, rice, and chickpea as query sequences. The predicted number of *MGT* genes varied across plant species, with some species possessing a higher number compared to soybean, while others had lower counts (Table 1).

**Table 1:** Comparison of the number of *MGT* genes in soybean with those in other plant species.

Plant Species	Scientific Name	Number of Genes	References
Camelina	*Camelina sativa* L.	62	[11]
Durum wheat	*Triticum turgidum* L.	41	[11]
Rapeseed	*Brassica napus* L.	36	[58]
Soybean	*Glycine max* L.	29	
Rice	*Oryza sativa* L.	23	[59]
Wheat	*Triticum aestivum* L.	18	[60]
Maize	*Zea mays* L.	12	[14]
Arabidopsis	*Arabidopsis thaliana* L.	10	[16]
Banana	*Musa paradisiaca* L.	10	[19]
Grape	*Vitis vinifera* L.	9	[61]

The basic physicochemical properties of GmMGT proteins, including size, MW, pI, protein instability index, aliphatic index, and GRAVY, were analyzed (Table 2). The length of amino acid sequences varied from 127 aa (GLYMA.06G208700) to 556 aa (GLYMA.15G125900) with relative MW 14.05 kDa (GLYMA.06G208700) to 62.27 kDa (GLYMA.15G125900). The pI values varied from 4.66 (GLYMA.13G368400) to 9.91 (GLYMA.06G053100). It was found that 48.28% of GmMGT proteins were acidophilic (pI values < 7.00), in comparison to 61% of OsMGT in the rice genome showed an acidophilic nature [59]. The instability index indicated that 16 GmMGTs (55.17%) were stable proteins (instability index < 40). Moreover, the aliphatic index ranged from 86.10 (GLYMA.09G019600) to 135.24 (GLYMA.06G208700), suggesting that most GmMGT proteins were thermally stable. The GRAVY values ranged from −0.09 (GLYMA.02G117100) to 1.37 (GLYMA.06G208700), with 19 GmMGT proteins (65.42%) exhibiting positive values, suggesting their hydrophobic nature. A similar trend was observed in the rice genome, where 56.52% of OsMGT proteins are also hydrophobic [59]. This finding suggest that the *MGT* gene family has significantly expanded in soybeans compared to other species.

**Table 2:** List of 29 *GmMGT* genes and their basic physiochemical characterizations.

SI	Gene identifier	Size (aa)	Molecular weight (kDa)	pI	Instability index	Aliphatic index	GRAVY
1	*GLYMA.02G068000*	444	48.93139	8.69	30.71	108.71	0.481
2	*GLYMA.02G117100*	415	46.43603	5.13	37.25	96.14	−0.089
3	*GLYMA.02G280800*	350	37.88685	8.16	32.76	117.31	0.670
4	*GLYMA.02G285600*	543	60.89483	6.30	40.93	95.35	−0.130
5	*GLYMA.03G159400*	389	43.66696	5.10	44.09	102.04	−0.156
6	*GLYMA.04G005200*	335	36.26084	6.70	26.38	126.68	0.700
7	*GLYMA.05G153000*	322	35.07050	8.29	28.21	109.31	0.776
8	*GLYMA.05G168200*	452	50.67294	5.87	61.93	95.85	−0.275
9	*GLYMA.05G196600*	345	37.15873	6.58	31.42	119.62	0.696
10	*GLYMA.06G005000*	363	39.34139	7.07	23.04	122.49	0.625
11	*GLYMA.06G053100*	339	36.87612	9.91	40.38	123.73	0.688
12	*GLYMA.06G159100*	346	37.64531	6.88	35.89	115.88	0.642
13	*GLYMA.06G208700*	127	14.05412	9.00	18.83	135.24	1.371
14	*GLYMA.08G126600*	451	50.61472	5.82	60.09	96.07	−0.279
15	*GLYMA.09G019600*	463	51.82573	6.43	37.64	86.10	−0.327
16	*GLYMA.10G180200*	391	43.45459	4.94	41.95	101.10	−0.114
17	*GLYMA.11G105300*	181	20.13566	4.74	24.78	126.11	0.661
18	*GLYMA.11G255400*	327	35.41056	8.36	21.41	106.75	0.569
19	*GLYMA.12G030100*	374	40.94906	7.63	25.46	111.05	0.457
20	*GLYMA.12G168000*	351	38.08081	7.04	45.40	111.43	0.612
21	*GLYMA.13G368400*	416	46.44155	4.66	42.32	93.49	−0.246
22	*GLYMA.14G033700*	350	37.84578	8.11	33.64	114.81	0.679
23	*GLYMA.14G097400*	350	37.85816	9.41	44.09	123.70	0.669
24	*GLYMA.15G125900*	556	62.26694	6.16	44.16	95.86	−0.244
25	*GLYMA.16G003900*	348	37.88368	7.04	42.87	112.39	0.625
26	*GLYMA.16G149500*	444	49.00137	8.48	35.07	104.74	0.477
27	*GLYMA.17G227100*	344	37.12332	9.61	44.27	123.62	0.663
28	*GLYMA.18G091200*	162	18.06860	7.87	20.13	124.10	1.057
29	*GLYMA.20G210300*	396	44.02708	4.94	43.88	98.81	−0.148

### 3.2. Phylogenetic analysis of arabidopsis, rice, soybean, and chickpea MGT proteins

**Fig 1:**
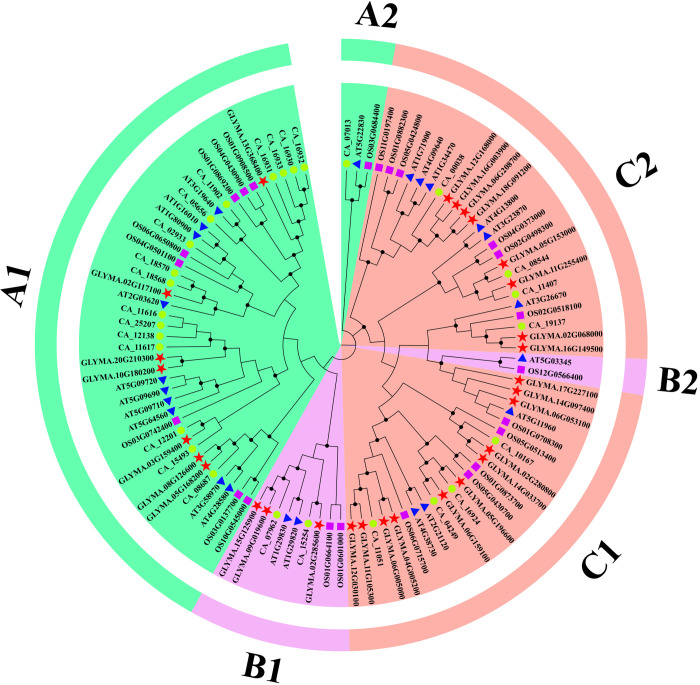
Phylogenetic relationship of 29 GmMGTs, 23 AtMGTs, 23 OsMGTs, and 27 CaMGTs proteins. MGT proteins were classified into 3 groups A for MRS2, B for CorA, and C for NIPA type MGTs including 6 subfamilies (A1, A2, B1, B2, C1, and C2) and marked by various colors. The GmMGTs are marked by a red star, the AtMGTs are marked by a blue triangle, the OsMGTs are marked by a pink rectangle, and the CaMGTs are marked by orange circle.

To elucidate the evolutionary relationship of MGT protein family members, a phylogenetic tree was constructed using 102 MGT protein sequences comprising 29 GmMGTs, 23 AtMGTs, 23 OsMGTs, and 27 CaMGTs proteins in soybean, arabidopsis, rice, and chickpea (Fig 1). Based on the phylogenetic analysis, the MGT proteins were categorized into three groups (A, B, and C), including six subgroups (A1, A2, B1, B2, C1, and C2). Subgroups A1 comprised 7 MRS2-type GmMGT proteins, B1 contained 3 CorA-type GmMGT proteins. The subgroups C1 and C2 included 11 and 8 NIPA-type GmMGT proteins (S5 Data). Arabidopsis and rice MGT family proteins have previously been categorized into six clusters [59]. MGT proteins in durum wheat and camelina were clustered into nine categories, indicating increased diversity in MGT isoforms [11]. Four subgroups (except A2 and B2) contained MGT proteins from the above three species, whereas no MGT proteins of soybean were clustered in the A2 and B2 subgroups. Group C, consisting of 47 proteins, was the largest, followed by Group A (44 proteins), while Group B was the smallest, containing only 11 MGT proteins. Notably, the number of NIPA-type GmMGT clade-related proteins was much larger than the others, indicating a certain duplication event in this clade during the evolution process, which may be highly useful in soybean stress adaptation. The pronounced diversity of NIPA-type GmMGTs further implies that MRS2 and CorA proteins may have evolved from ancestral NIPA proteins.

### 3.3. Gene structure analysis

**Fig 2:**
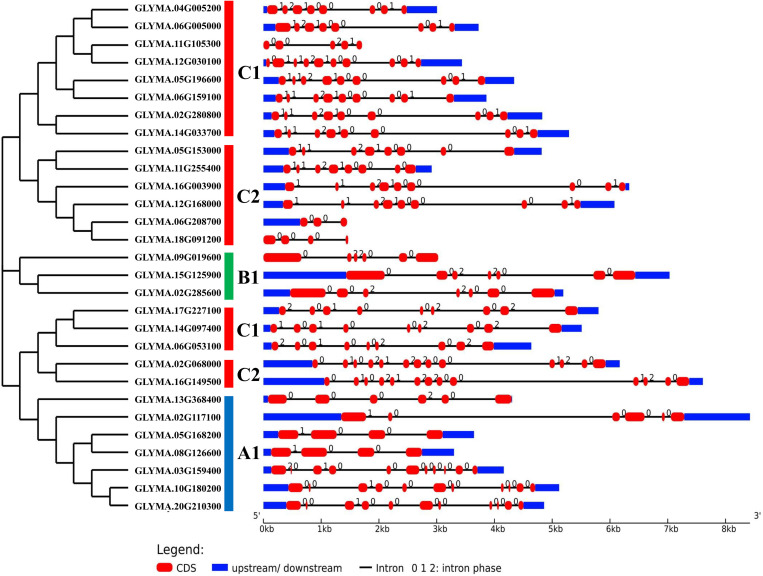
The gene structure of *GmMGT* genes. In *GmMGT* gene structure, black lines represent introns, red-bolled lines represent exons and blue-bolled lines represent UTRs. The exon/intron structure of each *GmMGT* gene is proportionally displayed according to the scale at the bottom.

The gene structures of *GmMGT*s were analyzed to determine their clade-specific characteristics and evolutionary significance. This analysis revealed that 25 *GmMGT* genes have both 5′ UTR and 3′ UTR, while 3 *GmMGT* genes (*GLYMA.11G105300*, *GLYMA.18G091200*, and *GLYMA.09G019600*) lack UTR sequences, and *GLYMA.06G208700* has only 5′ UTR sequences (Fig 2; S6 Data and S7 Data). The number of exons ranged from 3 (*GLYMA.06G208700*) to 13 (*GLYMA.02G068000* and *GLYMA.16G149500*), with *GLYMA.02G117100* having the largest gene sequence of 8 kb long. The exon number in a gene may enhance the diversity of the coding gene by influencing post-transcriptional modifications such as alternative splicing [62,63]. Genes with fewer introns are known to respond quickly to stress and contribute significantly to environmental adaptation [64]. Interestingly, in most genes, the first exon and the last exon were relatively longer than the middle exons, implying a complex gene structure. These findings suggest that the *GmMGT* genes exhibit structural diversity, resulting in a wide range of gene activities.

### 3.4. Conserved domain and motif analysis

**Fig 3:**
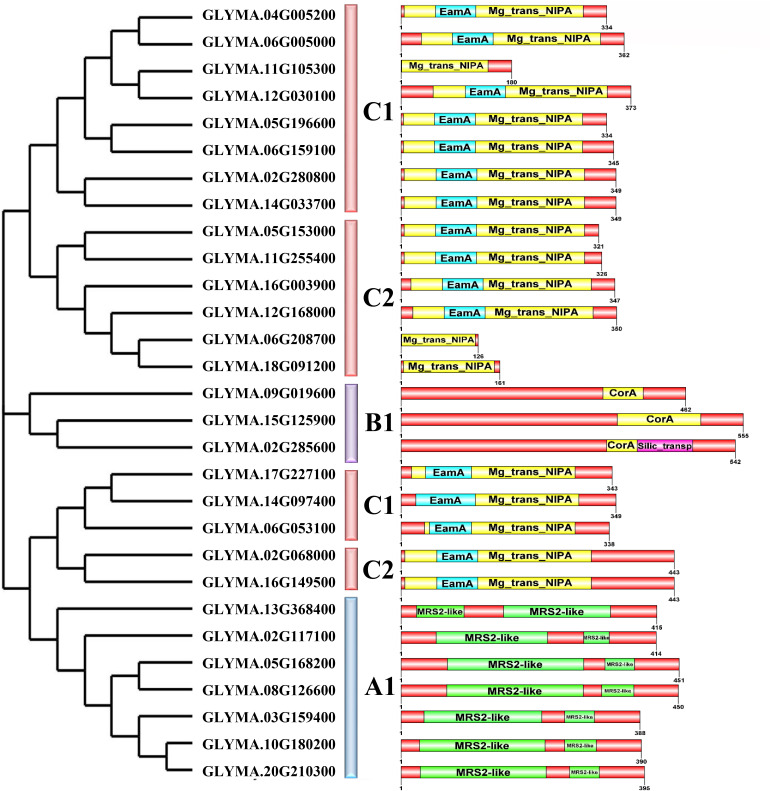
Distribution of conserved domain within the GmMGT proteins. The relative positions of each domain are shown in color boxes.

For definitive categorization, GmMGT protein sequences were subjected to conserved domains, and motifs identification based on phylogenetic tree topologies. Three types of signature domains were determined, such as MRS2 in A1, A2, and CorA, along with silic_trans domain in B1, and NIPA, along with the Eama additional domain in subgroups C1 and C2 (Fig 3). The NIPA domain is responsible for various cellular processes, including magnesium homeostasis, metal detoxification, vesicle trafficking, plant development, and stress responsiveness. The CorA domain is involved in cell division, stress responses, and regulating cellular osmotic pressure [65]. The MRS2 domain facilitates the transport of Mg2+ from cytosol to the mitochondrial matrix to regulate mitochondrial metabolism and enhance inter-organellar communication [66].

**Fig 4:**
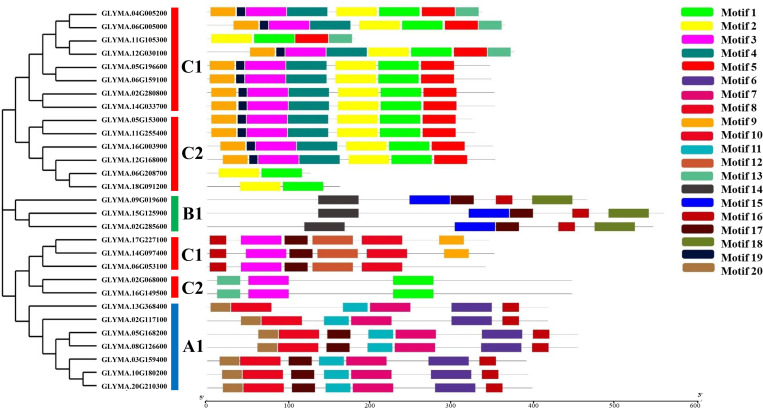
Distribution of conserved motifs of GmMGT proteins. Each colored box aligned on the right side of the figure indicates a specific motif. Different colors represented various motifs distributed in the domains of the proteins.

**Fig 5:**
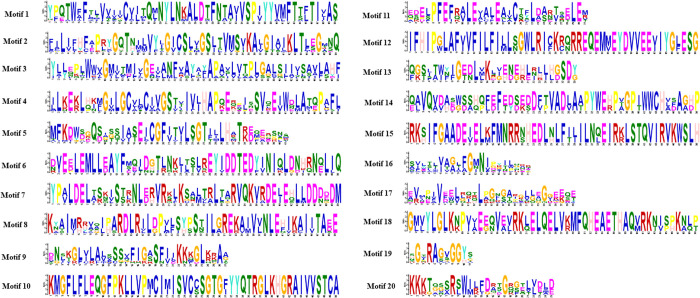
MEME suite logo of the identified motifs. The motif logo illustrates the conserved amino acid positions across the aligned protein sequences. The overall height of each stack represents the information content of that position in the motif in bits. The height of the individual letters indicates the relative frequency of the corresponding amino acid at that position.

Twenty conserved motifs were determined, ranging in length from 11 aa to 50 aa in length (Fig 4 and Fig 5; Table 3). GmMGT proteins in group C had the highest number of motifs, including motif 1, motif 2, motif 3, motif 4, motif 5, motif 9, motif 10, motif 12, motif 13, motif 16, motif 17, and motif 19. Groups A and B had 7 and 5 conserved motifs, respectively. Motif 16 and motif 17 were found across all groups, suggesting their potential role in functional divergence. Although there were some variations, the motif numbers and the arrangements of start and end motifs were highly conserved within each subgroup, which align with the motif organizations in grape (*V. vinifera L*) [61] and orange (*Citrus sinensis*) [67]. Overall, the conserved domains and motifs act as markers of the GmMGT family and can be used to verify the candidate genes using various approaches.

**Table 3:** Conserved motifs in the amino acid sequences of GmMGT proteins.

Motif	Width	Consensus sequence
1	50	YPQTWFFTTVVIICCITQMNYLNKALDTFNTAVVSPIYYVMFTTFTIVAS
2	50	FILIFHFEPRYGQTHMMVYIGICSLVGSLTVMSVKAIGIAIKLTLEGMNQ
3	50	YLYEPWWWVGMITMIVGECANFVAYAYAPAVLVTPLGACSIIVSAVFAHF
4	50	ILKEKLHKMGILGCVLCIVGSTTIVIHAPQEQPIHSVQEIWDLATQPAFL
5	41	MFKDWDGQDPSQIASEICGFITVLSGTFMLHKTKDMEQSNQ
6	50	DVEELEMLLEAYFMQIDGTLNKITSLREYIDDTEDYINIQLDNHRNQLIQ
7	50	YPALDELTSKISTRNLERVRKLKSNMTRLTARVQKVRDEIEHLLDDDDDM
8	50	KYAIMRHVQIPARDLRILDPVFSYPSTILGREKAIVVNLEHIKAIITAEE
9	31	DNSKGLCLAVCSSVFIGASFIIKKKGLKRAA
10	50	KMGFLFLEQGFPKLLVPMCIMISVCCSGTGFYYQTRGLKHGRAIVVSTCA
11	31	EDELPFEFRALEVALEAICTYLDARTSELEM
12	50	IFHIPGLAFVVFILFILLSGWLRICKRQRREQEMMEYDVVEEVIYGLESG
13	29	QGSITWYIGEDLMKDIENEHLNHLHGSDY
14	50	QAVQVDADWSSHQFEFEDSEDDFTVADLAAPYWEHPVGPIWWCHVDAGHP
15	50	RKHIFGAADEIELKFMNRRNHEDLNLFILILNQEIRKLSTQVIRVKWSLH
16	21	SVYTTVAGTFGMNIPCTWQKK
17	29	EVMPIVEWLRQQLPGNGARKLQEGGEEQE
18	50	GMVYLGLKNPVVEEQVEVRKLELQELVKMFQHEAETHAQVRKNIPPKNLP
19	11	HGTRAGVGGYS
20	25	KKKTQGSRSWIRFDRSGQGELVDLD

### 3.5. Ka/Ks analysis of *GmMGT* gene family

**Fig 6:**
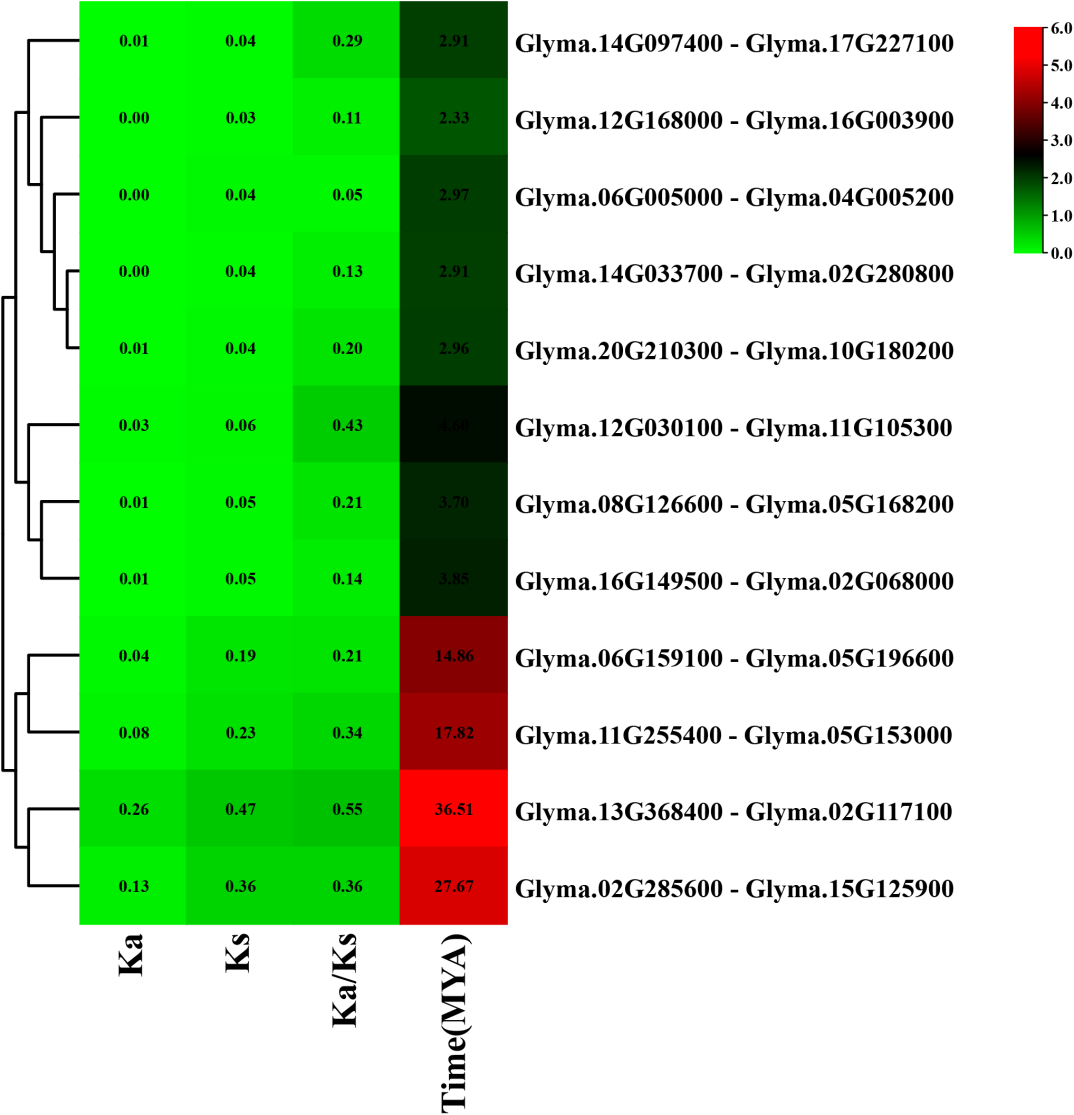
Gene duplication estimation for different paralogous gene pairs of *GmMGT* genes based on Ks and Ka values. The number of nonsynonymous substitutions per nonsynonymous site is presented by Ka and the number of synonymous substitutions per synonymous site is represented by Ks. Ka/Ks represents the ratio of nonsynonymous (Ka) versus synonymous (Ks) changes.

To determine the selective pressure and possible evolutionary relationship of the *GmMGT* genes, the Ka/Ks ratios of 12 segmental duplicated gene pairs of *GmMGT* were evaluated (Fig 6 and S8 Data). The substitution pressure of *GmMGT* duplicated gene pairs ranged from 0.05 (*GLYMA.06G005000*-*GLYMA.04G005200*) to 0.55 (*GLYMA.13G368400*-*GLYMA.02G117100*), revealing their purifying selection (Ka/Ks < 1). These segmentally duplicated genes were found to undergo strong environmental pressure and maintain stable protein functions [68]. This finding is consistent with other gene families in soybeans, such as SWEET [69], NHX [70], GRAS [71], CBF/DREB1 [72], Aux/IAA [73], IQD [74], and NOX [75]. However, positive selection (Ka/Ks > 1) was observed in the majority of the duplicated *GmPOD40* genes [76]. As natural selection has no effect on the Ks values, the Ks values were utilized to determine the divergence period of the duplicated gene pairs using a clockwise rate of 6.5 × 10^–9^ mutations per synonymous site per year. Here, the divergence period varied from 2.33 MYA (*Glyma.12G168000-Glyma.16G003900*) to 36.51 MYA (*Glyma.13G368400-Glyma.02G117100*) with an average of 10.26 MYA. This analysis implies that the *GmMGT* genes primarily evolved through purifying selection which was a major driving force for *GmMGT* evolution at the protein level.

### 3.6. Collinearity and synteny analyses

**Fig 7:**
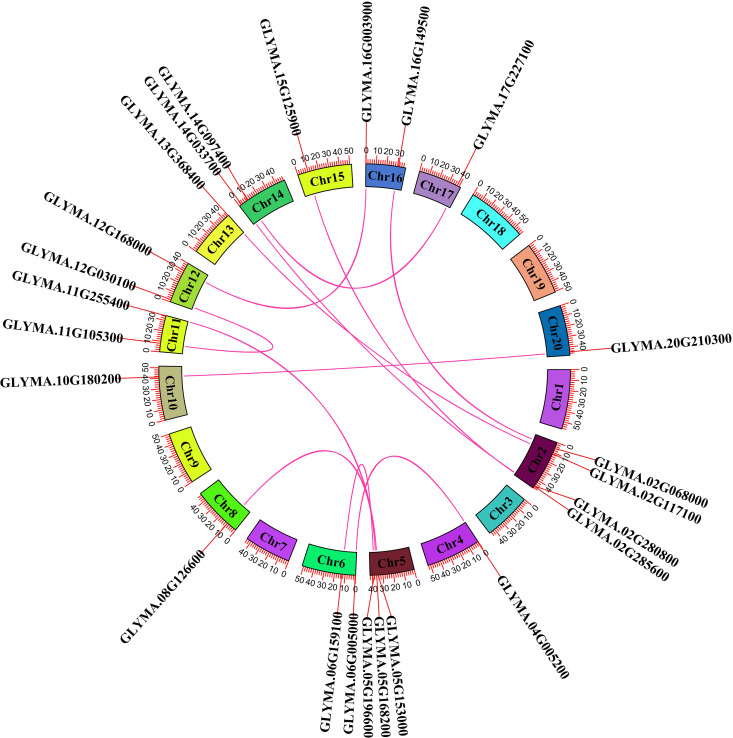
Collinearity analysis of the *MGT* genes in soybean. Chromosomes 1-20 are represented by various colored rectangles. Different colored lines indicate collinear blocks in the soybean genome, while colored lines *GmMGT* collinear gene pairs.

**Fig 8:**
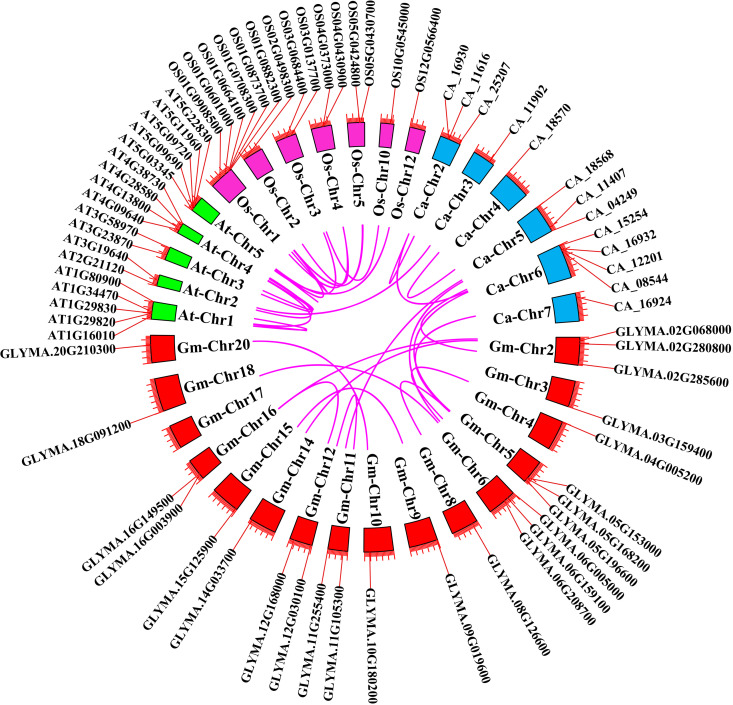
Synteny analysis of *MGTs* between soybean, arabidopsis, rice, and chickpea. Red color blocks represent the syntenic blocks of soybean and light green, pink, and aqua color blocks represent the syntenic blocks of arabidopsis, rice, and chickpea respectively. The colored lines represent the syntenic gene pairs of *MGTs*.

Collinearity and synteny analyses were conducted to determine the changes in evolution and replication events in the *GmMGT* gene family. Collinearity, a form of synteny, requires conserved gene order. A total of 12 *GmMGT* collinear gene pairs were identified, with the most collinear genes (4 genes) located on chromosome 2 (Fig 7). Conversely, the lowest number of collinear genes (only 1 gene) was identified on Chr4, Chr8, Chr10, Chr13, Chr15, Chr17, and Chr20. Synteny relationships were established across soybean, arabidopsis, rice, and chickpea, identifying 9, 7, 6, and 3 syntenic gene pairs, respectively (Fig 8). The number of synteny gene pairs between chickpea and arabidopsis, rice and arabidopsis, chickpea and soybean were 1 (*Ca_11902-AT3G19640*), 3 (*OS01G0708300*-*AT5G11960*, *OS03G0684400*-*AT5G22830*, and *OS12G0566400-AT5G03345*), and 6 (*CA_12201-GLYMA.03G159400, CA_15254-GLYMA.02G285600, CA_04249-GLYMA.06G159100, CA_16924-GLYMA.05G196600, CA_11407-GLYMA.11G255400, CA_08544-GLYMA.05G153000)*, respectively. The highest number of syntenic genes (6 genes) was identified on Chr1 in rice. Interestingly, no syntenic gene pairs were identified in soybean with rice, and arabidopsis, implying a closer genetic relationship with chickpea than arabidopsis and rice. The significant synteny predicted between rice-maize and arabidopsis-rice *MGT* genes indicated close evolutionary relations due to chromosomal arrangement and duplication processes [15,77]. These collinearity and synteny analyses at the gene level can reveal complex evolutionary relations and chromosomal distribution of soybeans over genome evolution.

### 3.7. Chromosomal location and gene duplication events for *GmMGTs*

**Fig 9:**
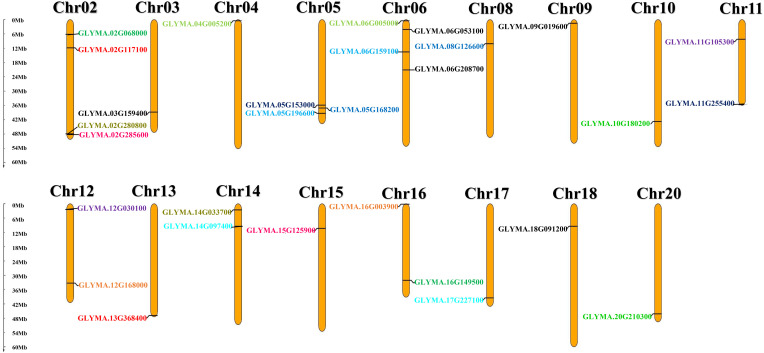
Chromosomal locations and duplications of *GmMGT* genes. The chromosome number is indicated above each bar. The chromosome scale is in millions of bases (Mb), indicating chromosomal length on the left. Each gene pair of segmental duplication is indicated by a similar color.

The chromosomal mapping demonstrated that *GmMGT* genes was unevenly distributed over 17 of the 20 chromosomes within 60 Mb chromosomal length (Fig 9). The highest number of *GmMGT* genes were identified on chromosomes Chr02, and Chr06 (4 genes each), followed by Chr5 (3 genes), Chr11, Chr12, Chr14, and Chr16 (2 genes each). Only one gene was found on Chr03, Chr04, Chr08, Chr09, Chr10, Chr13, Chr15, Chr17, Chr18, and Chr20. In general, no relationship was found between chromosomal length and gene number,. The majority of these genes may not have evolved from a similar evolutionary subclade. The duplication analysis identified only 12 segmental duplications and no tandem duplication in the *GmMGT* genes, similar to the findings in banana [19] and maize [14]. Segmental duplication enhances the rapid adaptation of plant genomes to new environmental conditions [78]. Therefore, it is hypothesized that the expansion of the *MGT* genes through segmental duplication plays an essential role in soybean adaptation to environmental changes.

### 3.8. Prediction of subcellular localization of GmMGT family members

**Fig 10:**
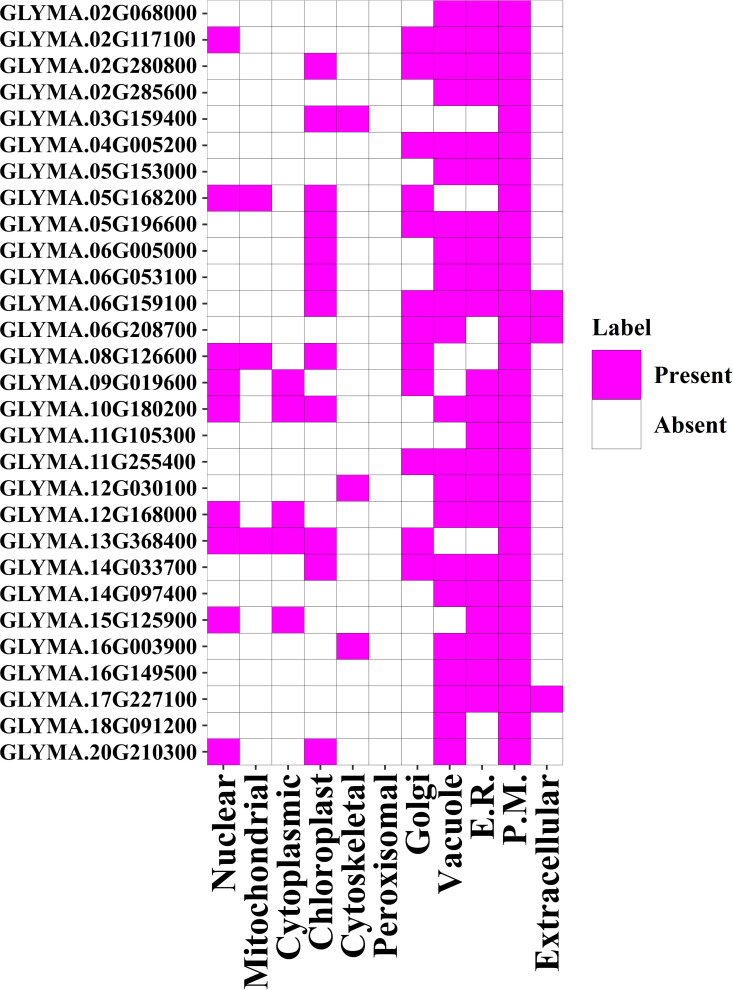
Sub-cellular localization analysis for the GmMGT proteins, represented by a heatmap. Sub-cellular localization of GmMGT proteins in different cellular organelles is represented by a heatmap. The name of each GmMGT protein is shown on the left side of the heatmap and the name of each corresponding cellular organelle is shown at the bottom of the heatmap. The color pink in the heatmap represents presence and the color white-absence. Here endoplasmic reticulum is represented by E.R., and the plasma membrane is represented by P.M.

**Fig 11:**
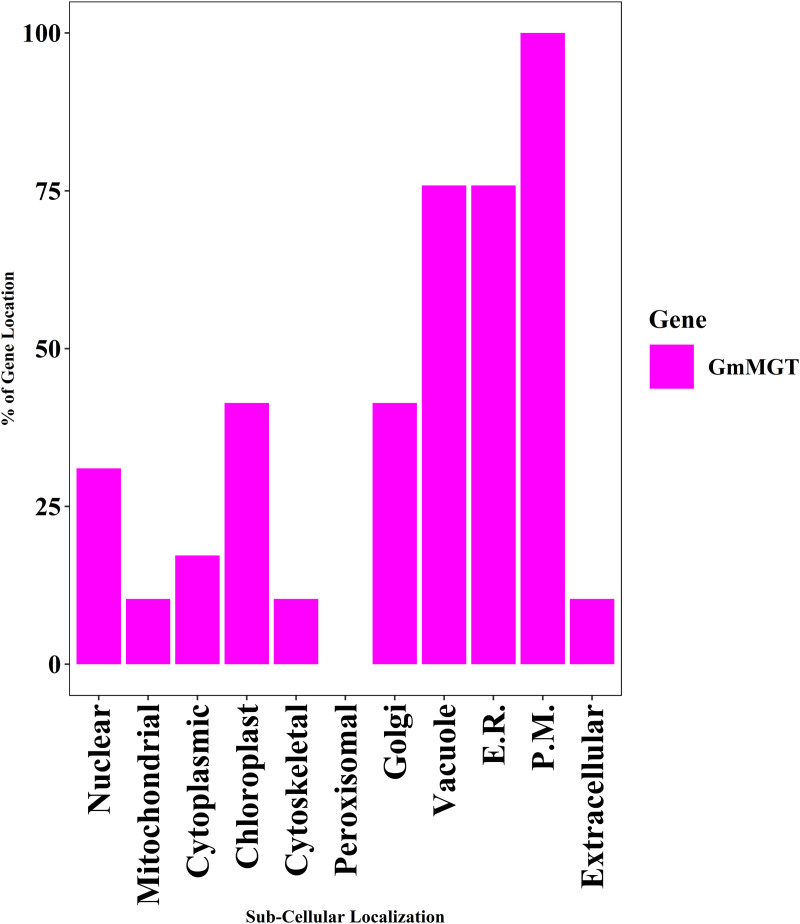
Sub-cellular localization analysis for the GmMGT proteins, represented by bar diagram. The percentage of GmMGT protein that appeared in different cellular organelles is shown on the left side of the bar diagram. The cellular organelles including the nucleus, mitochondria, cytoplasm, chloroplast, cytoskeletal, peroxisome, Golgi, vacuole, **E.**R., **P.**M., and extracellular organelles, are presented at the bottom of the bar diagram.

Proteins are distributed throughout cells to participate in various physiological processes. According to web-based predictions, GmMGT protein signals were identified in the nucleus, mitochondria, cytoplasm, chloroplast, cytoskeleton, peroxisome, Golgi, vacuole, endoplasmic reticulum (E.R), plasma membrane (P.M), and extracellular organelles (Fig 10 and Fig 11; S9 Data). The maximum protein signals were detected on P.M (100%), followed by 75.86% (22 GmMGTs) in both E.R and vacuole, 41.38% (12 GmMGTs) in both Golgi and chloroplast. In banana and orange*,* maximum protein signals (70% MaMGT and 71.43% CsMGT) were also identified in the P.M [19,67]. Therefore, a better understanding of the proteins present in the plasma membrane could help in exploring techniques to enhance plants’ natural defenses [79]. GmMGT proteins are assumed to be important in glycosylation, transporting and storing nutrients and metabolites, as they were also predominantly found in E.R. and vacuole. In wheat, cytoplasmic proteins TaMGT3D.1 and TaMGT4D were upregulated after stripe rust pathogen infection [60]. Thus, GmMGT proteins predicted in the cytoplasm might be upregulated under biotic stresses. This study demonstrated that the GmMGT proteins are organelle-specific, with most GmMGTs functioning intracellularly in various microenvironments.

### 3.9. Gene Ontology (GO) analysis of *GmMGT* genes

**Fig 12:**
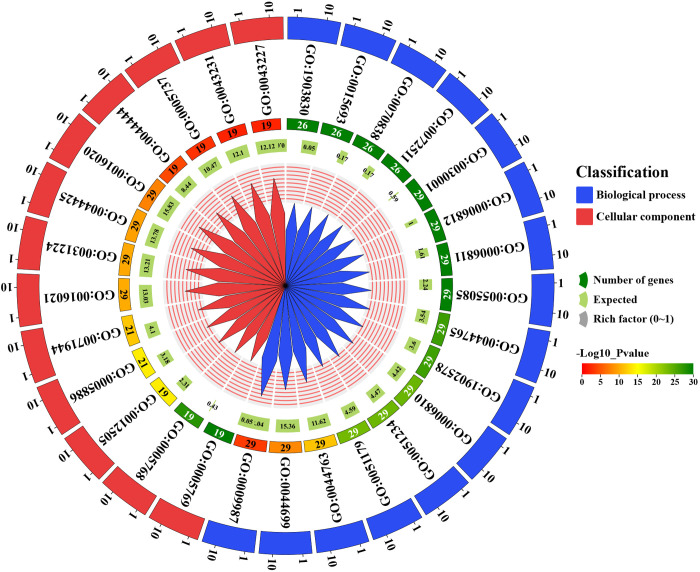
GO analysis of *GmMGT* genes. GO enrichment analysis of *GmMGT* is represented by a diagram. GO terms of *GmMGT* genes associated with biological processes, and cellular components are denoted by blue and red triangles, respectively. The number of the corresponding *GmMGT* genes (19 to 29) is shown inside of the circle by different colors.

To specifically identify the gene functions, GO analysis was performed for 29 *GmMGTs* (Fig 12 and S10 Data). GO provides a comprehensive framework for categorizing genes based on their biological processes, molecular functions, and cellular components in which their respective gene products are active. The biological process is the most diverse and largest group among these three GO categories [80]. The *GmMGT* genes were annotated with two fundamental GO categories, including biological processes and cellular components. A total of 16 GO terms were associated with the biological processes category, and 13 with cellular components. The most significantly enriched biological process GO terms include transmembrane transport (GO:0055085), metal ion transport (GO:0030001), magnesium ion transmembrane transport (GO:1903830), and divalent inorganic cation transport (GO:0072511), with *p*-value: 1.00E-30. In terms of cellular components, the most enriched GO term was the early endosome (GO:0005769, *p*-value: 1.00E-30). Notably, *GmMGTs* were mostly related to membrane cellular components, including intrinsic component of membrane (GO:0031224, *p*-value: 1.20E-10), and integral component of membrane (GO:0016021, *p*-value: 8.30E-11). Previous studies have reported that the prevalent biological process GO category like the “cellular process” is enhanced during abiotic stresses [81]. This annotation analysis results indicate that *GmMGT* genes are enriched in the membrane and involved in various ion, and organism transportation.

### 3.10. *Cis*-acting regulatory elements (CAREs) analysis of *GmMGT* gene promoters

**Fig 13:**
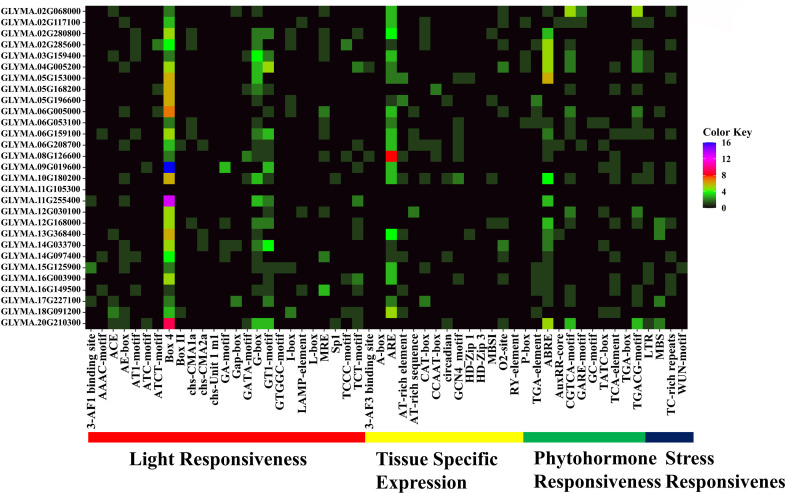
The distribution of putative CAREs on the 2.0 kb promoter region of *GmMGT* genes, represented by a heatmap. The name of each *GmMGT* gene is represented on the left side of the heatmap. The number of putative CAREs of each *GmMGT* gene is shown on the right side of the heatmap and represented by four different colors (black = 0, green = 1-4, red = 5-8, pink = 9-12, and purple = 13-16). Light responsiveness, tissue-specific expression, phytohormone responsiveness, and stress responsiveness functions associated with CAREs of the corresponding genes are shown at the bottom of the heatmap and denoted by red, yellow, green, and blue-colored bolled lines, respectively.

CAREs are essential for regulating the expression and functions of genes. To evaluate the regulatory activity of *GmMGT* genes, a total of 53 CAREs were identified using 2000 bp of 5’ UTR (Fig 13; S11 Data and S12 Data). *GmMGT* contained 24 *cis*-acting elements related to light responsiveness, 14 to tissue-specific expression, 11 to phytohormone responsiveness, and 4 to stress responsiveness, revealing the potential of these genes during the diverse developmental and stimulus processes [58]. Nine major *cis*-elements were dominantly associated with four different functions, including light-responsive elements: Box 4, G-box, and glycosyltransferase 1 (GT1-motif); tissue-specific elements: AU-rich element (ARE); phytohormone responsive elements: abscisic acid-responsive element (ABRE), CGTCA-motif, and TGACG-motif; and stress responsive elements: long terminal repeat (LTR), and thymine-cytosine-rich repeats (TC-rich repeats).

The most abundant light-responsive element, Box 4, was present in all *GmMGT* genes, specifically enriched in *GLYMA.09G019600* and *GLYMA.11G255400.* Moreover, G-box and GT1-motif were abundantly identified in 21 and 19 *GmMGTs* gene promoters, respectively. These four *cis*-elements are effective in gene regulation by light signals and photosynthetic mechanisms, ultimately stimulating genes involved in defense processes [82]. Tissue-specific elements were abundant in *GLYMA.06G15900*, *GLYMA.08G126600*, and *GLYMA.10G126600*. Furthermore, the highly detected tissue-specific element, ARE, and phytohormone-responsive element, ABRE, are involved in numerous biological functions linked to soybean growth and development. Phytohormone-responsive elements were identified abundantly in the *GLYMA.02G068000* gene promoter, while stress-responsive CAREs were abundant in *GLYMA.12G168000* and *GLYMA.20G210300*. LTR and TC-rich repeats were found abundantly in only 9 and 10 *GmMGT* genes, respectively. These two motifs regulate the expression of genes involved in stress response, allowing plants to grow and adapt to adverse conditions [83,84]. Similar findings have been reported in rice [59], orange [67], and banana [19]. Moreover, nine major *cis*-elements were identified in *GLYMA.06G159100*, and *GLYMA.10G180200* promoter regions, and *GLYMA.10G180200* contained the highest number of motifs (18) followed by *GLYMA.02G285600* (17), *GLYMA.06G159100* (17), indicating their higher efficiency in several biological processes, including soybean growth, development and stress resistance.

### 3.11. Putative microRNA target site analysis

A total of 81 different miRNAs were found, ranging from 20–24 nucleotides in length, targeting 27 out of the 29 *GmMGT* genes (S13 Data). In this analysis, the number of miRNAs per gene ranged from 1–28, and the lowest number of miRNAs was identified in *GLYMA.03G159400*, *GLYMA.06G005000*, and *GLYMA.06G208700*. *GLYMA.17G227100* was targeted by the highest number (28 miRNAs) of miRNA, followed by *GLYMA.14G097400* (20 miRNAs), *GLYMA.02G117100* (16 miRNAs). Gma-miR169 was highly abundant (31), comprising 23 members (a-h, j-m, p, q, s, u-z, aa, and ab). Gma-miR169 targeted *GLYMA.02G117100*, *GLYMA.10G180200*, *GLYMA.14G097400*, and *GLYMA.17G227100.* Previous studies have demonstrated that the miR169 is susceptible to several abiotic conditions such as salinity, drought, and cold stresses in various plant species [85]. In Arabidopsis, miR169 regulates stress-induced flowering by suppressing the transcription factor *AtNFYA* [86]. This suggests that *GmMGTs* are involved in promoting vegetative growth as well as stress responses in soybeans.

### 3.12. Transcription factor (TF) analysis of *GmMGT* genes

**Fig 14:**
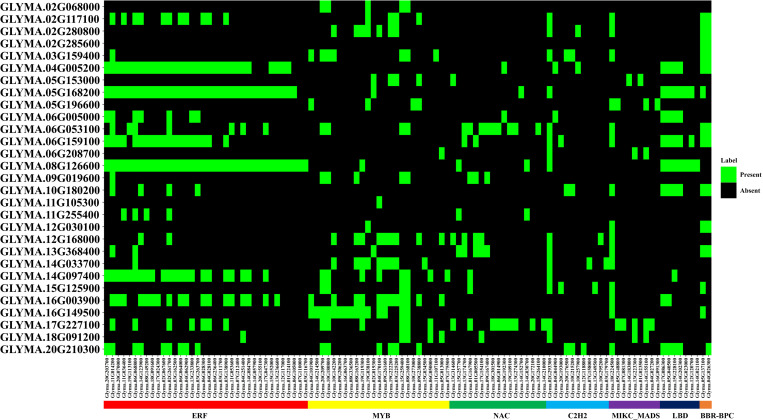
TF analysis in the promoter region of the *GmMGT* family, presented by heatmap. The name of each *GmMGT* gene is shown on the left side of the heatmap. The major seven TFs are shown on the bottom of the heatmap and represented by seven different colors (ERF = Red, MYB = Yellow, NAC = Green, C2H2=Light blue, MIKC_MADS = Purple, LBD = Dark blue, BBR_BPC = Orange). Black colored boxes represent the absence and green colored boxes- the presence of TFs, aligned on the right side of the figure.

**Fig 15:**
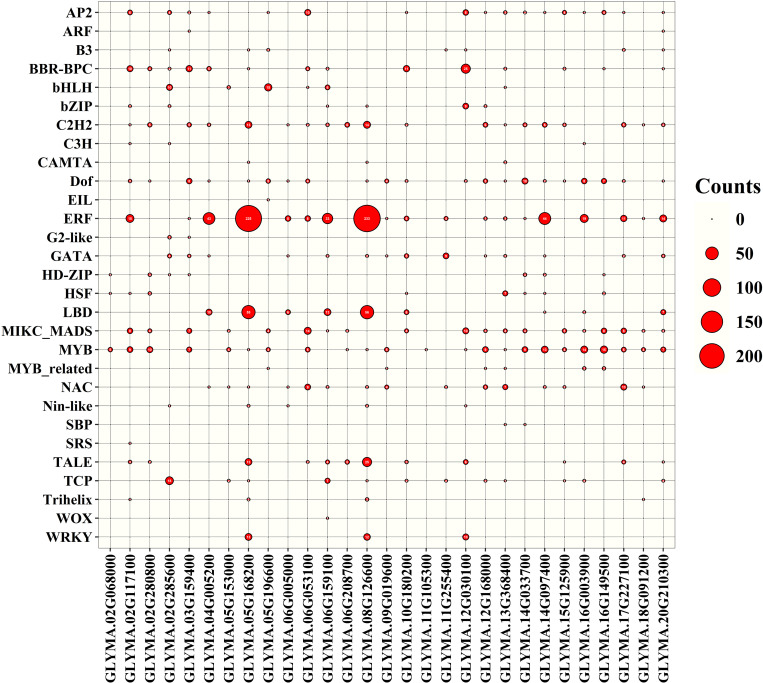
TF analysis in the promoter region of the *GmMGT* family, presented by bubble chart. The name of each *GmMGT* gene is shown at the bottom of the bubble chart. The transcription factors of each *GmMGT* gene are shown on the left side of the bubble chart. The number of transcription factors binding sites (count = 0-200) of each *GmMGT* gene is shown on the right side of the bubble chart.

**Fig 16:**
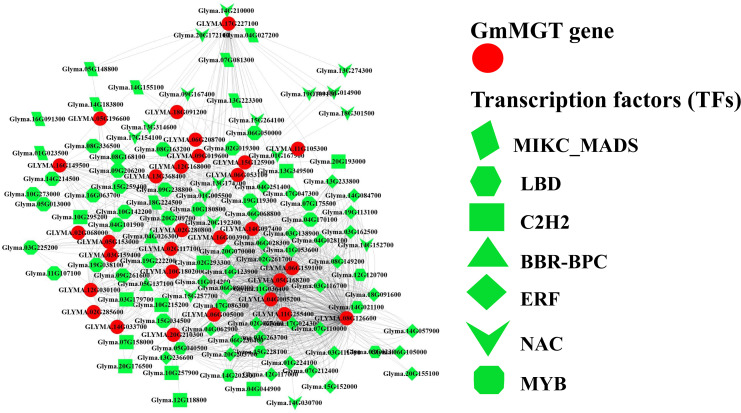
TF analysis in the promoter region of the *GmMGT* family, presented by TF network. Regulatory network nodes of interaction between *GmMGT* genes and their regulatory transcription factors are represented by the TF network. The *GmMGT* genes and major TF families are shown on the right side of the figure with particular shapes.

TFs are proteins characterized by the presence of at least one DNA-binding domain and are responsible for regulating transcriptional pathways in plant cells. Based on network and sub-network analysis, we identified 29 TFFs in the promoter region of targeted 29 *GmMGT* genes in which the transcription factor binding sites (TFBSs) varied in number and distribution (Figs 14–16 and S14 Data). For instance, TFBFs were more numerous in *GLYMA.08G126600*, while only one TFBF was identified in *GLYMA.11G105300*. According to our findings, 7 TFFs, including ERF, MYB, NAC, C2H2, MIKC-MADS, LBD, and BBR-BPC were considered as major TFFs, comprising 74.22% of the total TFs.

Among the TFs, the ERF family contained the highest number of TFs (36), followed by MYB (25), NAC (17), C2H2 (11), MIKC-MADS (9), LBD (7), and BBR-BPC (2). ERF-associated TFBSs accounted for 52.9% of all binding sites and were overrepresented in 65.6% of *GmMGT* genes. The ERF regulates plant resistance to various abiotic stresses such as drought, salt, cold, and other adversities [87]. On the other hand, MYB is engaged in various biological activities, including circadian rhythm, stress responsiveness, cell identity, seed and floral development, and metabolic control in plants [88]. The NAC family has been demonstrated to influence a wide range of developmental processes, and the overexpression or knockdown of NAC gene expression impacts plant defense and signaling systems [89]. The abundance of various TFs in the *GmMGT* gene promoters suggests that *GmMGT* genes are complexly integrated in various signal-transduction mechanisms with a potential reservoir of functional diversity.

### 3.13. Expression pattern analysis of *GmMGT* genes in different tissues

**Fig 17:**
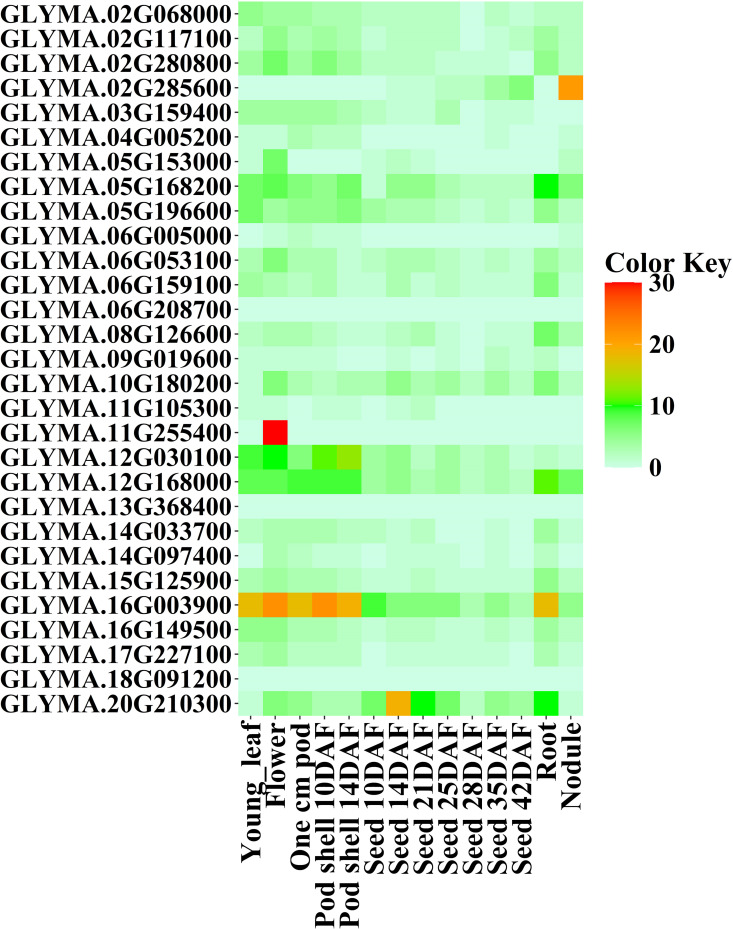
Expression profiles of *GmMGT* genes in 14 tissues of soybean based on the Soybase data. RPKM normalized values of expressed genes were log2-transformed and visualized as a heatmap. The name of each *GmMGT* gene is shown on the left side and the tissue types are represented at the bottom of the heatmap based on data retrieved from the SoyBase database. The expression values were mapped using a color gradient from white to red (count = 0-30) shown on the right side of the heatmap.

To analyze the tissue-specific expressions of *GmMGT* genes, available RNA-seq data were collected from SoyBase and Bar databases. Expression profiles were visualized using two heatmaps encompassing 14 tissues, including four common types (leaf, flower, root, and nodule). According to SoyBase data, the maximum *GmMGT* genes exhibited different expression patterns in young leaf, flower, one cm pod, pod shell 10 DAF (Days after flowering), pod shell 14 DAF, seed 14 DAF, seed 21 DAF, and seed 35 DAF (Fig 17 and S15 Data). Additionally, *GLYMA.05G168200*, *GLYMA.05G196600*, *GLYMA.06G159100*, *GLYMA.06G208700*, *GLYMA.10G180200, GLYMA.12G030100*, *GLYMA.12G168000*, *GLYMA.15G125900*, *GLYMA.16G003900*, *GLYMA.16G149500, GLYMA.20G210300* were differentially expressed in each tissue type. Conversely, no expression was detected for *GLYMA.06G208700*, *GLYMA.13G368400*, and *GLYMA.18G091200*. Notably, *GLYMA.12G030100*, *GLYMA.12G168000*, *GLYMA.16G003900*, and *GLYMA.20G210300* exhibited elevated expression patterns in various tissues. Particularly, *GLYMA.16G003900* was highly induced in young leaf, flower, one cm pod, pod shell 10 DAF, pod shell 14 DAF, seed 10 DAF, and root. The induced expression of 25 *GmMGTs* was observed in flower tissue, with *GLYMA.11G255400* having the highest expression profiles among them. In contrast, maximum genes exhibited the lowest expression pattern in nodules, except for *GLYMA.02G285600* which expressed highly in nodules.

**Fig 18:**
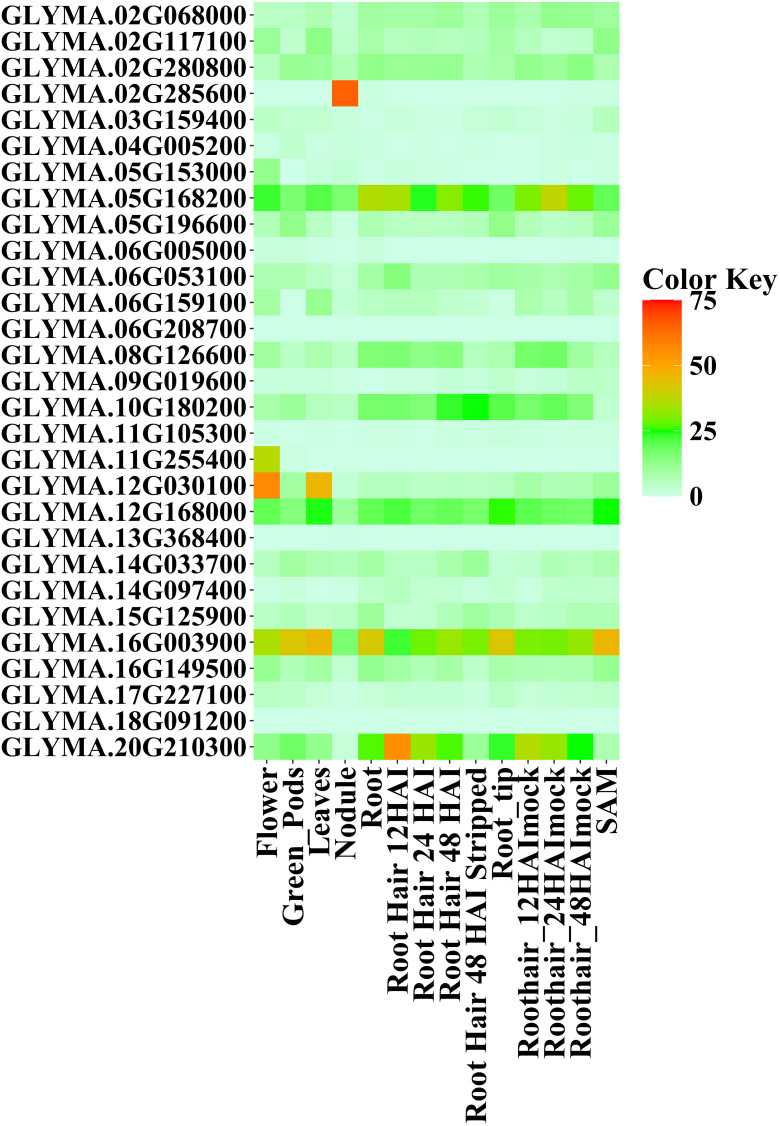
Expression profiles of *GmMGT* genes in 14 tissues of soybean based on Bar uTorrent data. RPKM normalized values of expressed genes were log2-transformed and visualized as a heatmap. The name of each *GmMGT* gene is shown on the left side and the tissue types are represented at the bottom of the heatmap based on data retrieved from the Bar uTorrent database. The expression values were mapped using a color gradient from white to red (count = 0-75) shown on the right side of the heatmap. The abbreviations “HAI” and “SAM” on the tissue label represent “Hours after inoculation” and “Shoot apical meristem”, respectively.

BAR database analysis revealed that most *GmMGT* genes were expressed in flower, root hair 12 HAI, and in the shoot apical meristem (SAM) tissues in Soybean (Fig 18 and S16 Data). *GLYMA.02G280800*, *GLYMA.02G068000, GLYMA.02G117100, GLYMA.03G159400, GLYMA.05G168200*, *GLYMA.05G196600*, *GLYMA.06G053100*, *GLYMA.08G126600*, *GLYMA.10G180200*, *GLYMA.12G030100*, *GLYMA.12G168000*, *GLYMA.12G030100*, *GLYMA.14G033700*, *GLYMA.15G125900*, *GLYMA.16G003900*, *GLYMA.16G149500*, and *GLYMA.20G210300* were expressed differentially in each tissues whereas no expression profile was identified in *GLYMA.06G208700*, and *GLYMA.18G091200.* Six *GmMGTs*, including *GLYMA.05G168200*, *GLYMA.10G180200*, *GLYMA.12G030100*, *GLYMA.12G168000*, *GLYMA.16G003900*, and *GLYMA.20G210300*, showed elevated expression in different types of tissues. *GLYMA.16G003900* exhibited higher expression patterns in flower, green pods, leaf, roots, root hair 48 HAI, root hair 48 HAI stripped, root tip, root hair 48 HAI mock, and in SAM, being selected as a highly expressed candidate gene. The highest expression profiles of the largest number of genes (25 *GmMGTs*) were detected in flower tissue. Nevertheless, all of the *GmMGT* genes showed the lowest expression pattern in nodules, while *GLYMA.02G285600* was highly induced in nodules compared to others. Based on the findings from both databases, the majority of genes showed higher expression in flower tissues and lower expression in nodules. This variation in expression patterns indicates that *GmMGT* genes may have diverse roles, with tissue-specific regulation. Similar variations in expression patterns have been observed in arabidopsis [90] and rice [91], suggesting that the expression of *GmMGT* genes during different developmental stages is highly tissue-specific.

### 3.14. Transcriptomic analysis of *GmMGT* genes in response to soybean aphid infestation

**Fig 19:**
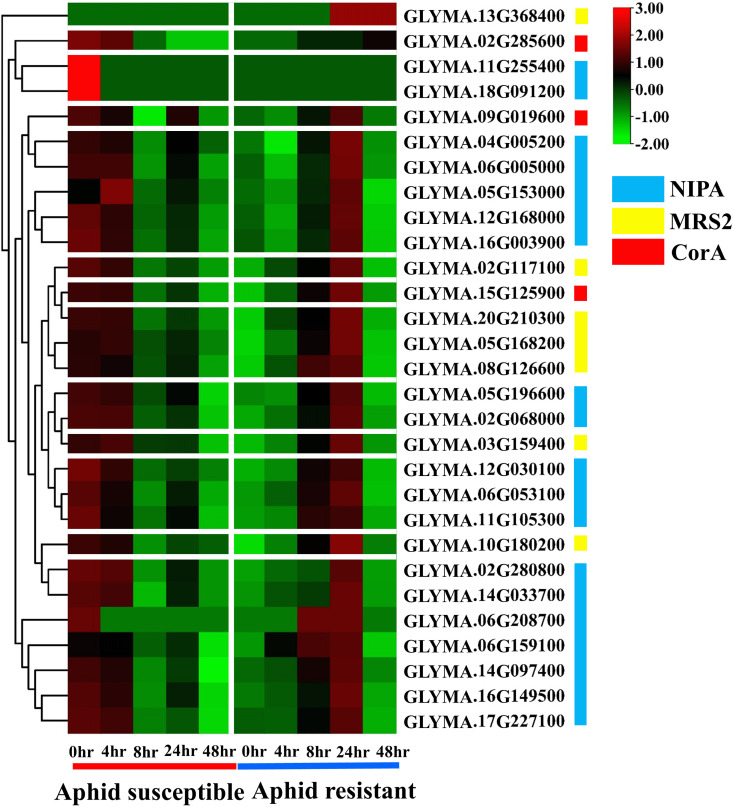
Gene expression analysis between susceptible and resistant *GmMGTs* at different periods in response to aphid infestation. Clustering of *GmMGT* genes from three clades (NIPA, CorA, and MRS2) are shown according to their expression profiles between susceptible and resistant soybean cultivars in different periods (4h, 8h, 24h, 48h) with control (0h; no aphid). The expression values were mapped using a color gradient from low to high (green to red color) shown on the right side of the heatmap. NIPA, MRS2, and CorA type *MGT* genes were denoted by light blue, yellow, and red color respectively. Separators have been used to distinguish between different conditions on the heatmaps.

In this study, previously observed gene expression levels (log2 fold change) between susceptible and resistant *GmMGTs* were further studied using top leaves of soybean cultivars, sampled at five different sampling times: 0h (no aphids), 4h, 8h, 24h, and 48h with 15 adult aphids [56] (Fig 19 and S17 Data). NIPA-type *GLYMA.11G255400* and *GLYMA.18G091200* showed no expression at any time point in both susceptible and resistant soybean genotypes. In susceptible varieties, only MRS2-type *GLYMA.03G159400* and *GLYMA.05G168200*, and NIPA-type *GLYMA.05G153000* were expressed at 4h after infestation. In resistant varieties, 17 *GmMGTs* were upregulated at 4h, while 26 *GmMGTs* were upregulated at 8h after infestation. Among them, 27 *GmMGT* genes were induced at 24h, but only 10 *GmMGTs* remained upregulated after 48h of aphid infestation. Notably, 10 *GmMGT* genes (NIPA-type *GLYMA.02G068000*, *GLYMA.02G280800*, MRS2-type *GLYMA.03G159400*, *GLYMA.05G168200*, *GLYMA.08G126600*, *GLYMA.10G180200*, *GLYMA.13G368400*, *GLYMA.20G210300* and CorA-type *GLYMA.02G285600*, *GLYMA.15G125900*) exhibited higher expression level under all four time point (4h, 8h, 14h, 48h) in resistant genotype. The expression pattern of MRS2-type *GLYMA.13G368400* was highest after 24h and 48h of infestation, while CorA-type *GLYMA.02G285600* exhibited an induced expression pattern in 8h, 24h, and 48h after aphid infestation in the resistant soybean genotype. Aphids have a major impact on the photosystem pathways of susceptible soybean cultivars, which also explains the adverse effects of aphid infestation on soybean yield. Differentially expressed gene clusters have been identified in various aphid-plant systems studies, such as soybean [56] and sugarcane [92], which provide insights into genes and processes responsible for resistance to aphids. An aphid-resistant line of sorghum (*Sorghum bicolor*) displayed greater upregulation of lipid and protein-regulating genes, cellular catabolic processes, and enhanced transcriptional initiation after sugarcane aphid infestation than the susceptible line, supporting our findings [92]. Therefore, the upregulated genes may serve as potential candidates for conferring aphid resistance provided by the resistant line of the soybean MGT gene family.

### 3.15. Transcriptomic analysis of *GmMGT* genes under dehydration and salt stress

**Fig 20:**
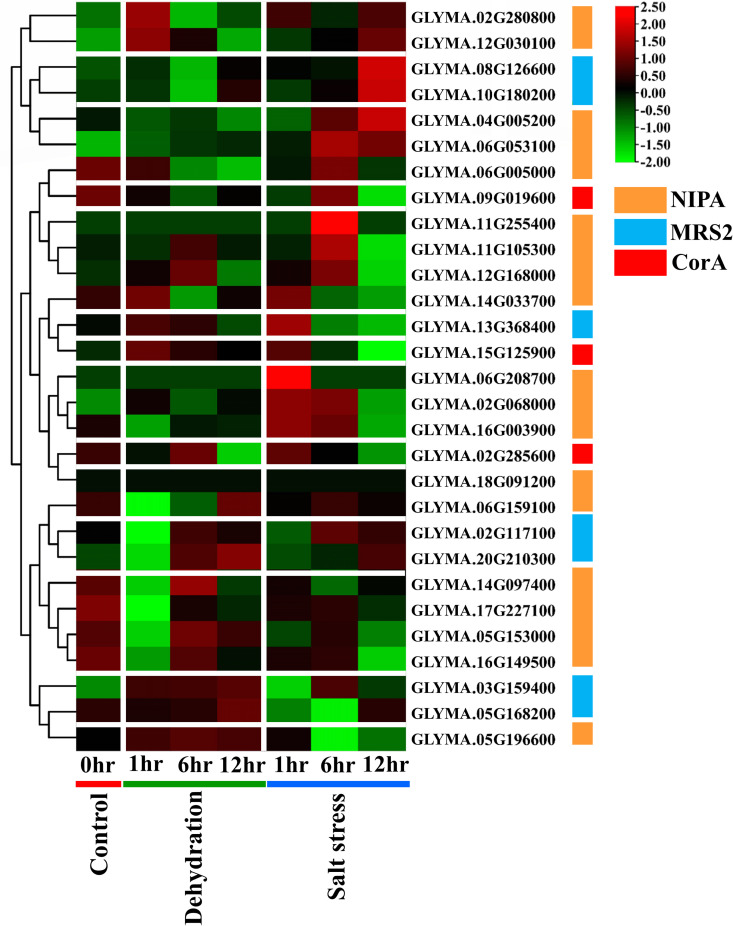
Expression profiles of *GmMGT* genes at different time points under dehydration and salt stress treatments. Clustering of *GmMGT* genes from three *GmMGT* clades (NIPA, CorA, and MRS2) are shown according to their expression profiles under dehydration and salt stress treatments in different periods (1h, 6h, 12h) with control (0h). The expression values were mapped using a color gradient from low to high (green to red color) shown on the right side of the heatmap. NIPA, MRS2, and CorA type *MGT* genes were denoted by orange, light blue, and red color, respectively. Separators have been used to distinguish between different conditions on the heatmaps.

To identify *GmMGT* genes responding to abiotic stresses, RNA-seq data were analyzed at three different time points (1h, 6h, 12h) with control (0h) under dehydration and salt stresses [57] (Fig 20 and S18 Data). Among 29 *GmMGTs*, NIPA-type *GLYMA.02G068000*, *GLYMA.05G196600*, *GLYMA.06G053100*, MRS2-type *GLYMA.03G159400*, and CorA-type *GLYMA.15G125900* exhibited elevated expression in response to dehydration. *GmMGTs* are engaged in both early and late responses to abiotic stresses. For instance, the NIPA-type *GLYMA.05G196600* and MRS2-type *GLYMA.03G159400* consistently upregulated at all three time points, while CorA-type *GLYMA.15G0125900* was upregulated after 1h of treatment. These genes could be positive regulators for enhancing dehydration stress endurance in soybeans. It has been observed that Mg2 + deficiency may trigger oxidative stress and stomatal closure, which can reduce plant gene transpiration [93]. Moreover, MGTs can modify water usage efficiency and drought tolerance by regulating antioxidant enzymes, photosynthesis, and stomatal closure [4,94].

Under salt stress, NIPA-type *GLYMA.02G068000*, *GLYMA.04G005200*, *GLYMA.06G053100*, *GLYMA.12G030100*, MRS2-type *GLYMA.08G126600*, *GLYMA.10G180200* were highly induced. The expression levels of NIPA-type *GLYMA.04G005200* and MRS2-type *GLYMA.08G126600*, *GLYMA.10G180200* showed the highest expression levels after 12h of salt stress treatment, considered as strong candidates under salt stress conditions. Notably, CorA-type *GmMGT* genes showed no significant differences in expression under salt stress, while NIPA genes were more expressed than MRS2 genes. The *OsMGT1* gene may significantly boost *OsHKT1* activity, which decreases excess Na+ in rice tissues and enhances resistance under salinity stress [93]. In summary, this transcriptomic study indicates that *GmMGT* genes with differential expression levels under various abiotic stresses may be involved in the Mg2 + transporting system and many cellular pathways.

## 5. Conclusion

In this study, 29 *GmMGT* genes were identified and characterized in the soybean genome for the first time, using comprehensive bioinformatics tools. These genes were found to be unevenly distributed across 17 chromosomes, with 12 segmental duplicated gene pairs. Segmental duplication was found to be the main driving force for the expansion of *GmMGT* genes. The structural variabilities in gene sequences confirm the diverse roles of these genes in plant developmental processes. Notably, most of the *GmMGT* genes in the same subgroups exhibited highly similar gene structure, conserved domains, and motif organization. The collinearity and synteny analyses lay a foundation for understanding crop evolution. Furthermore, our exploration of GO terms associated with various biological processes suggested that the majority of *GmMGT* genes were involved in the ion transportation process. The abundance of light-responsive *cis*-elements in *GmMGT* gene promoters indicates their potential involvement in photosynthesis. The presence of TFs and miRNA molecules in the promoter region indicates the signal transduction process of *GmMGTs* involved in response to various environmental stresses. At the same time, the expression profiles in various tissues and stress conditions confirm the overlapping functions of these genes. Highly induced genes, *GLYMA.02G285600,* and *GLYMA.13G368400* in aphid infestation, *GLYMA.03G159400,* and *GLYMA.05G196600*, *GLYMA.15G0125900* in dehydration, *GLYMA.04G005200*, *GLYMA.08G126600*, and *GLYMA.10G180200* in salt stress, can be used as potential targets for developing stress-resistant soybean cultivars. This study provides the theoretical groundwork for further functional exploration of *GmMGT* genes in stress responses and plant developmental processes.

## Supporting information

S1 Data Protein sequences of AtMGT, OsMGT, GmMGT, and CaMGT. (S1 Data.DOCX)

S2 Data CDS of *GmMGT* gene families. (S2 Data.DOCX)

S3 Data Genomic sequences of *GmMGT* genes. (S3 Data.DOCX)

S4 Data Protein sequences of *GmMGT* genes. (S4 Data.DOCX)

S5 Data Distribution of *GmMGT* genes among groups based on phylogenetic analysis with *Arabidopsis*, rice, and chickpea *MGT* genes. (S5 Data.DOCX)

S6 Data *In silico* predicted the number of introns and exons in *GmMGT* genes. (S6 Data.DOCX)

S7 Data Exon-intron sequences of *GmMGT* genes to identify gene structure. (S7 Data.XLSX)

S8 Data Time of gene duplication estimatsed for different paralogous pairs of *GmMGT* genes based on Ka and Ks values to demonstrate gene evolution. (S8 Data.XLSX)

S9 Data Predicted protein signals of GmMGTs in subcellular organelles. (S9 Data.XLSX)

S10 Data The details GO analysis of the predicted *GmMGT* genes was performed using the Plant TFDB. (S10 Data.XLSX)

S11 Data Promoter region of *GmMGT* genes. (S11 Data.DOCX)

S12 Data The predicted CAREs of the upstream promoter region (2.0 kb genomic sequences) of *GmMGT* gene family members are predicted to identify the gene response to light, tissue, hormone, and stress. (S12 Data.XLSX)

S13 Data miRNA targeted prediction of GmMGTs. The miRNA data was downloaded from the plant micro RNA encyclopedia (http://pmiren.com/).
(S13 Data.DOCX)

S14 Data Identified main 7 TF families associated with the regulation of identified *GmMGT* genes in *G. max* genome. (S14 Data.XLSX)

S15 Data Tissue-specific expression profiles of *GmMGT* genes retrieved from SoyBase database. (S15 Data.XLSX)

S16 Data Tissue-specific expression profiles of *GmMGT* genes retrieved from Bar uTorrent database. (S16 Data.XLSX)

S17 Data Expression profiles of *GmMGT* genes under biotic stress like Aphid infestation. (S17 Data.XLSX)

S18 Data Expression profiles of *GmMGT* genes under abiotic stress like dehydration and salt stresses. (S18 Data.XLSX)
