# Supplementary material for: Genome-wide identification and functional characterization of magnesium transporter (MGT) gene family in soybean (Glycine max L.) and their expression profiles in response to aphid infestation, dehydration, and salt stresses
Source: PLoS One. 2025 Aug 29;20(8):e0330440. doi: 10.1371/journal.pone.0330440 (PMC12396710; doi:10.1371/journal.pone.0330440)
Supplement: S1 Data — (S1 Data.DOCX) [file pone.0330440.s001.docx]

>AT1G29820

MGENGENHSVSQESKSKKLESKNHSGVPKSDLWTDGIICAFEFIRGPKKHVDSKQLEKGALKQEDGPISHSFPGHNPFVDSSPVDDLRSRSVSSLDFKEAHLLPSGHVERYEGSHWVPIGWARITELVQMVQVNAEWPNLELIDDEEDVPVTDLAAPYWERPGGPTWWCHLSAGNSFVEGWLRSATWLHPAISLALRDESKLISERMRHLLYEVPVRVAGGLLFELLGQSVGDPVISEDDVPVVFRSWQAKNFLVSVMHIKGNVTNTNVLGITEVEELLYAGGYNVPRTVHEVIAHLACRLSRWDDRLFRKSIFGAADEIELKFMNRRNYEDLNLFSIILNQEIRKLSRQVIRVKWSLHAREEIIFELLQHLRGNIARHLLDGLRKNTREMLEEQEAVRGRLFTIQDVMQSSVRAWLQDKSLRVSHNLAVFGGCGLVLTIIVGLFGINVDGIPGAQNTPYAFGLFTFLMVLLGAILIVVGLVYLGLKKPITEEQVEVRKLELQDVVKIFQHEAETHAQLRRNNLSPTAGDVFDADYILIQ

>AT1G29830

MGENGENHSVSQESKNHSGVPKSDLWTDGIICAFEFIRGPKKHVDSKQLYKGYLKQEDGPIRHSFPGHNPFIDSPAVDYLRSRSVSSLDFKEAHVLPSGHVERSEGSHWVPIGWTRITKLVQQVQVNAEWPNLKLIDDEEDVPVTDLAAPYWERPGGPTWWCHLTAGNSFVEGWLRSATWLHPAISLALRDESKLISERMRHLLYEVPVRVDGGLLFELLGQSMGDPVIGEDDVPVVFRSWQAKNFLVSVMHIKGNVSKSNVLGITEVEELLYAGSYNVPRTIHEVIAHLACRLSRWDDRLFRKSIFGAADEIELKFMNRRNHEDLNLFSIILNQEIRKLARQTIRVKWSLHAREEIILELLQHLRGNIPRHLLEGLRNNTREMLEEQEAVRGRLFTIQDNIQSNIRSWLQDQSLNGSHNLAIFGGCGLVLTIILGLFSVNLDGVPGVKHTPYAFVLFSVFLVLIGIVLIAFGLRYLGPKKPITEEHVEARKLELQNVVKIFQHEAETHAQLRRNNLSPTAGDVFDADYFLIQ

>AT5G03345

MNLGFLVGVFGVLILSHAAYSTIQYRGLLKIMEEEFSRPPINVILELIIGLALCMWAALTFPGKFLSIHPDSDENRAVFLPDNSDFMIFNHRGRLFPPQIDMKF

>AT2G03620

MGEQLDPFSASNLPDFISSQKIGRPVNFEGQTNRGHPFSGLKKRGQSSRSWVKIDQDGNSAVLELDKATIMKRCSLPSRDLRLLDPLFIYPSSILGRERAIVVSLEKIRCIITAEEVILMNARDASVVQYQSELCKRLQSNHNLNVKDDLPFEFKALELVLELSCLSLDAQVNELEMEVYPVLDELATNISTLNLEHVRRLKGRLLTLTQKVQKVCDEIEHLMDDDDDMAEMYLTEKKERAEAHASEELEDNIGEDFESSGIVSKSAPVSPVGSTSGNFGKLQRAFSSIVGSHKSLLSSSSIGENIDQLEMLLEAYFVVVDNTLSKLSSLKEYIDDTEDLINIKLGNVQNQLIQFQLLLTAATFVAAIFAAVTAVFGMNLQDSVFQNPTTFQYVLLITGIGCGFLYFGFVLYFKHKKVFPL

>AT4G28580

MGSLRRSTSNRSRKKGTAVKMNRMPSSLSPPTPPCSAIVGGTGKSKKRRGGVCLWTRFDRTGFMEVAGCDKSTIIERSSVSAKDLRTAFSHSSKILAREKAIVLNLEVIKAVITSEQVMLLDSLRPEVLTLTDRLKHHFPRKDGPENILQASSHGHQEGGEEGLKSKLPFEFRVLEIAFEVFCSFVDSNVVDLETQAWSILDELTKKVSNENLKDLRSLKTSLTHLLARVQKVRDEIEHFLDDKEDMEDLYLTRKWIQNQQTEAASNSIVSQPNLQRHTSNRISTSMVTEEDDIDDMEMLLEAYFMQLEGMRNKILLMKEHIDSTEAYVKILQNSRRNGLIHLMMLVNIGNYAITAGTVVVNLFGMNIPIGLYSTPDIFGYVVWAVVALCIVLFIVTVGYAKWKKLLD

>AT1G80900

MSELKERLLPPRPASAINLRGDAGSRPSPSGRQPLLGVDVLGLKKRGQGLKSWIRVDTSANSQVIEVDKFTMMRRCDLPARDLRLLDPLFVYPSTILGREKAIVVNLEQIRCIITADEVLLLNSLDNYVLRYVVELQQRLKASSVTEVWNQDSLELSRRRSRSLDNVLQNSSPDYLPFEFRALEVALEAACTFLDSQASELEIEAYPLLDELTSKISTLNLERARRLKSRLVALTRRVQKVRDEIEQLMDDDGDMAEMYLTEKKKRMEGSLYGDQSLPVYRTNDCFSLSAPVSPVSSPPESRRLEKSLSIVRSRHDSARSSEDATENIEELEMLLEAYFVVIDSTLNKLTSLKEYIDDTEDFINIQLDNVRNQLIQFELLLTTATFVVAIFGVVAGIFGMNFEIDFFEKPGAFKWVLAITGVCGLVVFLAFLWYYKRRRLMPL

>AT1G16010

MSELKERLLPPRPASAMNLRDASVTRPSASGRPPLLGVDVLGLKKRGQGLRSWIRVDTSGNTQVMEVDKFTMMRRCDLPARDLRLLDPLFVYPSTILGREKAIVVNLEQIRCIITADEVLLLNSLDNYVLRYVVELQQRLKTSSVGEMWQQENSQLSRRRSRSFDNAFENSSPDYLPFEFRALEIALEAACTFLDSQASELEIEAYPLLDELTSKISTLNLERVRRLKSRLVALTRRVQKVRDEIEQLMDDDGDMAEMYLTEKKRRMEGSMYGDQSLLGYRSNDGLSVSAPVSPVSSPPDSRRLDKSLSIARSRHDSARSSEGAENIEELEMLLEAYFVVIDSTLNKLTSLKEYIDDTEDFINIQLDNVRNQLIQFELLLTTATFVVAIFGVVAGIFGMNFEIDFFNQPGAFRWVLIITGVCGFVIFSAFVWFFKYRRLMPL

>AT3G58970

MGKGPLSFRRLSSIRHRKKGSAVKDDSAQTSTPSSPPPPLPIHAGGSAVGATGKAKKKTGGARLWMRFDRTGAMEVVECDKSTIIKRASVPARDLRILGPVFSHSSNILAREKAIVVNLEVIKAIVTAEEVLLLDPLRPEVLPFVERLKQQFPQRNGNENALQASANVQSPLDPEAAEGLQSELPFEFQVLEIALEVVCSFVDKSVAALETEAWPVLDELTKNVSTENLEYVRSLKSNLTRLLARVQKVRDELEHLLDDNEDMADLYLTRKWIQNQQTEAILAGTASNSIALPAHNTSNLHRLTSNRSASMVTSNTEEDDVEDLEMLLEAYFMQLDGMRNKILTVREYIDDTEDYVNIQLDNQRNELIQLQLTLTIASFAIAAETLLASLFGMNIPCPLYSIHGVFGYFVWSVTALCIVLFMVTLGYARWKKLLGS

>AT3G19640

MRGARPDEFNFSTNPSTPNTGQPTPTYPAGVGGGGGGRKKGVGVRTWLVLNSSGQSEPKEEGKHSIMRRTGLPARDLRILDPLLSYPSTVLGRERAIVINLEHIKAIITAQEVLLLNSKDPSVSPFIDELQRRILCHHHATKPQEEQNSGGEPHTRVDPAQGEAGTEQSSGDQGSEAKKDAKQSLENQDGSKVLPFEFVALEACLEAASSSLEHEALRLELEAHPALDKLTSKISTLNLERVRQIKSRLVAITGRVQKVRDELEHLLDDDEDMAEMYLTEKLAQKLEDSSNSSMNESDTFEVDLPQGDEDDRLPPEFASEANRDGRYLQANDAHELLMSTQSALSRNSRGTHTSSTRSAMTNKLDVEELEMLLEAYFVQIDGILNKLSTLREYVDDTEDYINIMLDDKQNHLLQMGVMLTTATLVMSAFIAVAGVFGMNITIELFTDNKHGPSRFIWTVIGGSIGSICLYVGAIGWCKYKRLLE

>AT5G22830

MALTPIPSTFTSLFNFSDHSPYPSPSLHYLLPGSSPSFSLQLSALSRTPIYFEALKVLSRSKCFAKSPTTAEDFVGDYESLNVSDDDDGSDSNSSDGDNGGGRDDSKKIDSSSSSSSSDSTSLGIREPVYEVVEVKATGAISTRKINRRQLLKSSGLRPRDIRSVDPSLFMTNSVPSLLVREHAILLNLGSLRAIAMRDRVLIFDYNRRGGRAFVDTLMPRLNPRSMNGGPSMPFELEAVESALISRIQRLEQRLMDIEPRVQALLEVLPNRLTADILEELRISKQRLVELGSRAGALRQMLLDLLEDPHEIRRICIMGRNCTLRRGDDDLECTLPSDKLIAEEEEEEIEMLLENYLQRCESCHGQAERLLDSAKEMEDSIAVNLSSRRLEVSRFELLLQVGTFCVAVGALIAGIFGMNLRSYLEEQASAFWLTTGGIIIGAAVAFFLMYSYLSRRKIF

>AT5G09690

MSPDGELVPVDSSAVVTAKRKTSQLSRSWISIDATGQKTVLDVDKHVIMHRVQIHARDLRILDPNLFYPSAILGRERAIVLNLEHIKAIITAEEVLIRDSSDENVIPVLEEFQRRLPVGNEAHGVHGDGDLGEEDESPFEFRALEVALEAICSFLAARTTELEKFAYPALDELTLKISSRNLERVRKLKSAMTRLTARVQKVRDELEQLLDDDGDMADLYLTRKLVGASSSVSVSDEPIWYPTSPTIGSMISRASRVSLVTVRGDDETDVEELEMLLEAYFMQIDSTLNKLTELREYIDDTEDYINIQLDNHRNQLIQLELMLSAGTVCVSVYSMIAGIFGMNIPNTWNHDHGYIFKWVVSLTGTFCIVLFVIILSYARFRGLIGS

>AT5G64560

MAQNGYLVPADPSAVVTVKKKTPQASWALIDATGQSEPLDVDKYEIMHRVQIHARDLRILDPNLSYPSTILGRERAIVLNLEHIKAIITSEEVLLRDPSDENVIPVVEELRRRLPVGNASHNGGQGDGKEIAGAQNDGDTGDEDESPFEFRALEVALEAICSFLAARTAELETAAYPALDELTSKISSRNLDRVRKLKSAMTRLTARVQKVRDELEQLLDDDDDMADLYLSRKLSSASSPISSIGEPNWYTTSPTIGSKISRASRASLATVHGDENDVEELEMLLEAYFMQIDSTLNRLTTLREYIDDTEDYINIQLDNHRNQLIQLELVLSSGTVCLSMYSLVAGIFGMNIPYTWNDGHGYMFKYVVGLTGTLCVVVFVIIMSYARYKGLVGS

>AT5G09720

MLPNEELVPVKRITPQSSWSWISIDATGKKTVLDVDKYVIMHRVQIHARDLRILDPNLFYPSAILGRERAIVLNLEHIKAIITAKEVLIQDSSDENLIPTLEEFQTRLSVGNKAHGGQLDGDVVEEDESAFEFRALEVALEAICSFLAARTIELEKSAYPALDELTLKLTSRNLLRVCKLKSSMTRLTAQVQKIKDELEQLLEDDEDMAELYLSRKLAGASSPAIDSGEHINWYPTSPTIGAKISRAKSHLVRSATVRGDDKNDVEEVEMLLEAHFYANRQNFEQINRATRVCG

>AT5G09710

MSIDSDVDPSEVSTAKRKPSRSWLLIDAAGNSTMLNVDSYAIIRRVHIYARDLRVFESSISSPLSIRTREGAIVLNLEHIKVIITADEEFERRLGVENRERRGQPDGKEDSGAEVDAEKDESPFEFRALEVALEAICSFLAARTTELEKSGYPALNELASKDDDDLGDLCLSRKIATTSSPVSDSDEQINSYPTSPTIGAKISRAKSHLVRSATVRGDDQNDVEEVEMLLEAHYMQIDRTLNKLAELREYLDDTEDYINFQFEVIITAGSVCISVYSLVVGILSTNIPFSWNTKEHMFKWVVSATATLCAIFFVIIISYARYKKLVGN

>AT2G21120

METDNGKGLILAVASSVFIGSSFILKKKGLKRAGAIGTRAGYGGYTYLLEPLWWAGMVTMIVGEAANFVAYIYAPAVLVTPLGALSIIISAVLAHFLLKEKLKKMGVLGCVSCIVGSVVIVIHAPKEQTPNSVEEIWNLATQPAFLIYVAITMSIVLALILHFEPLCGQTNILVYIGICSLMGALTVMSIKAIGIAIKLTMEGVSQIGYPQTWLFVMVAVTCVVTQLIYLNKALDTFNAAIVSPVYYVMFTTLTIVASAIMFKDWSGQDAASVASELCGFITVLTGTMILHGTREEEQQQASSEHVRWYDSRKSMNEEHLVSLYSPEY

>AT4G38730

MVSDNEMGLVLAVSSSVFIGSSFILKKKGLKRAAANGTRAGFGGYTYLLEPLWWVGLVTMTFGEIANFVAYVYAPAVLVTPLGALSIIISAVLAHFLLDEKLRKMGVWGCVCCIVGSVMIVIHAPQEQTPNSVEEIWKLAMQPAFLIYVAISMSIVLALILYCEPLCGQTNILVYIGICSLMGSLTVMSIKAVGIAIKLTFEGINQIWYPETWFFAMVAAICVVMQMIYLNKALDTFNAAIVSPIYYVMFTTLTIVASAIMFKDWNGQNTDSIASEICGFITVLTGTVILHSTREEEQASPRRMRWQDSGKSFDEEHLTSLYSPEY

>AT4G09640

MVYSSGSWRDAYKGMSSDNVKGLVLALSSSIFIGASFIVKKKGLKKAGASGLRAGSGGYSYLLEPLWWIGMITMIVGEIANFAAYAFAPAILVTPLGALSIIISASLAHIILQEKLHTFGILGCALCIVGSVTIVLHAPQEQDIVSVLEVWNLATEPAFLFYAAAVVGAAIVLIVQFIPLYGQSHVMVYIGVCSLIGSLSVMSVKALGIALKLTFSGTNQLGYPQTWVFTVIVLFCVITQMNYLNKALDTFNTAVVSPIYYVMFTSLTILASVIMFKDWDRQSGTQIMTELCGFVTILSGTFLLHTTTDMVDGESKGNLSSEEDSHLLLRIPKHSEDSNGFVQDGIILSLRRQESAKSPRPARQNKQLEDDLEAVPLRRQESSLRS

>AT4G13800

MEEMSPDNIHGVILAVSSSIFIGSSFIIKKKGLKKAGVSGARAGEGGYGYLYEPWWWAGMITMIVGEIANFAAYAFAPAILVTPLGALSIIFSAVLAHFILEEKLHMFGILGCVLCVVGSTTIVLHAPHEQGIESVKQVWHLATEPGFLAYSAVVLVVVLALIFYYEPRYGKTHMIVYVGICSLMGSLTVMSVKAVAIAIKLTFSGMNQFKYFHAWIFIIVVTICCILQINYLNKALDNFNTAVISPVYYVMFTTFTILASMIMFKDWASQSGLQIATELCGFVTILSGTFLLHKTKDMGNSTSLRGSTSHSPRDTPVFINSGSSRSSNSTRPAIL

>AT1G71900

MAESSGSWRDSYKGMSSDNIKGLVLALSSSLFIGASFIVKKKGLKKAASTGTRAGVGGYSYLYEPLWWIGMTTMLLGEIANFAAYAFAPAILVTPLGAVSIIISAVLAHIILREKLHIFGILGCALCVVGSTTIVLHAPQEREIDSVIEVWNLATEPAFMFYASLVIGAAVFLIIRFVPQYGQTNVMVYIGICSLVGSLSVMSVKALGIALKLTFSGTNQLFYPQTWIFTLVVLTCVVTQLNYLNKALDTFNTAIVSPIYYVMFTSLTILASVIMFKDWDRQNGTQIVTEICGFVTILSGTFLLHRTKDMVEGSSVILPLRISKHINEEEGIPLRRQESLRSP

>AT1G34470

MASLSGSWRDAYKGMSSDNIKGLVLALSSSLFIGASFIVKKKGLKRAGASGLRAGSGGYSYLLEPLWWVGMITMIVGEIANFAAYAFAPAILVTPLGALSIIISAALAHVILHEKLHTFGLLGCVLCVVGSITIVLHAPQEQEIDSVLQVWNLATEPAFLLYAAAVVGAAIILIVQFVPQYGQSHVMVYIGVCSLVGSLSVMSVKALGIALKLTFSGMNQLIYPQTWVFTLIVLTCVITQMNYLNKALDTFNTAVVSPIYYVMFTSLTILASVIMFKDWDRQDGTQIVTELCGFVTILSGTFLLHKTKDMVDGSSSLGNLALRLPKQLEDSNGFEQEGIPLTLRRHECTKSPRPMRQFILPQDGPEAV

>AT3G23870

MDQMSPDNINGVILAVSSSIFIGSSFIIKKKGLKKAGASGVRAGEGGYGYLKEPWWWAGMITMIVGEVANFAAYAFAPAILVTPLGALSIIFSAVLAHFILKEKLHMFGILGCILCVVGSTTIVLHAPHEQKIESVKQIWQLAIEPGFLVYSAVIVIVVAILIFYYEPRYGKTHMIVYVGICSLMGSLTVMSVKAVAIAIKLTFSGTNQFKYFNTWIFILVVATCCILQINYLNKALDTFNTAVISPVYYVMFTTFTIIASMIMFKDWASQSGLKIATELCGFVTILSGTFLLHKTKDMGNSASGRGSISMPTRDTPVFTNSGSGRSSSSDKVAS

>AT3G26670

MGEWVIGAFINIFGSVAINFGTNLLKLGHNERERLALQDGGGKMPLKPIIHNQTWRVGILVFLLGNCLNFISFGYAAQSLLAALGSIQFVSNIAFAYVVLNKMVTVKVLVATAFIVLGNVFLVAFGNHQSPVFTPEQLAEKYSNVTFLVYCGILILIVAVHHFLYRKGEVLISTPGQEISSYWKMLLPFSYAVVSGAIGSCSVLFAKSLSNLLRLAMSSSYQLHSWFTYSMLLLFLSTAGFWMTRLNEGLSLYDAILIVPMFQIAWTFFSICTGCIYFQEFQVFDALRTTMFILGMMCVFIGISLLAPDDTRGNETKDNSSSLDSIVSSSVPTEEDRLIPQSSEDGHSKDTRVVVQGMYMKAADLIAKTKTACLAALGFGEDSINASAILVMPMVSSKITGFRGNGLERAKILSMRGSGWSKLAMEEEGTRMLEKTISSKA

>AT5G11960

MWESICLTLAATAGNNIGKVLQKKGTIILPPLSLKLKVLRAYAENKPWALGFLMDIVGALLMLRALSLAPVSVVQPVSGCGLAILSVFSHFYLKEVMNVFDWIGITVAGIGTIGVGAGGEEQEASLISVFQLLWLALVVAILFVLLNAWLHIFKRQRREQELGEYEVVEEIIYGLESGILFGMASVVSKMGFVFVEQGFSTMFIPMCISISICCSGTGFFYQTRGLKHGRAIVVSTCAAVASIVTGVVAGMFALGEKLPTSPSGRLLLLLGWLLIMLGVVLLVTSSRLIRHLPRSFRRSRQTSLERGFNIRRTTSHTPKDTNPSAVIQAATLHHLLSSPSKDKD

>OS01G0601000

MVNGEPLWHGVRHGGGGGDARKQHHHHHQLSHGFGAKDASASSPWSSSDGLWTDGLVCAFEFVRGGGGAHGFVTPANLCRSKCCSLLQSKDLAVQDRRRSLAAKIGDNGDEPRPPTVAPAESLWAPIGWRRITQLVGMVGGDAAAWHDDGQSMSLMEHDGGGDEQCDDITVADVAAPYWQRAAGPTWWCHVAAGHPAVDAWLAAARWLHPAICVALRDESVLISEKMKHLLYEVPVRVAGGLLFELLGQSVGDPARDEEDIPIVLRAWQAQNFLITALHVKGSAPNVNVIGVTEVQELLSACGSTGTAPKNIQEVIAHLASRLARWDDRLWRKYVFGAADEIELKFVNSVFRRKQEDLKLLCMIFNQDIRRLATQVIRVKWSLHAREEIIFELLKYLGGSTTKSLLEAIKKDARQMIEEQEAVRGRLFTIQDVMQSTLRAWSQEKSLRITHNLTIFGGCGLVLSIIAGLFGINVDGIPGAENTPYAFALFSALLFLVGLLLIIVGIVYFGLQKPISDEQVQVRKLELQELVSMFQHEAETHARVKEGVLRTDLPPRAADLICDDNGDSRLLVCDC

>OS01G0664100

MADVEGQQPHQVSGVSRAQDGVAKVSLGKEHVPGSELWTDGLICAFELIKGHKKLVQHKSWPTIDSMQEKEVPMHMKRHISRNGHHVATMKPEECDVVENPRQTEFANDPSLFKDRPVHVRAILDHKWVPIGWSRIAELVQRVQSDASWDSEPAEMTDSEDDYTVADVAAPYWQRPVGPTWWCHVTAGHPSVDAWLNSAHWMHPAIRTALRDESRLISDRMKYLLYEVPVRVAGGLLFELLGQSVGDPNREEEDIPIVLRSWQAQNFLVTAMHVKGPSSNINVLGVTEVQELLSAGGSQTPRSAHEVIAHLIGRLSRWDDRLFRKYVFGEADEIELKFVNRRNHEDLNLVSIILNQEIRRLATQVIRVKWSLHAREEIIIELLRHLRGNTTRVILDSIRKDTREMLEEQEAVRGRLFTIQDVMQSTVRAWLQDRSLRITHNLAIFGGGGMVLSIITGLFGINVDGIPGAQNTPYAFGLFAGLLFFVGFVLIGVGILYLGLQNPVTNEKVKVRKLELQDLVSAFQHEAEQHGKVREGLSRHSSSPKSSSASNVDYVLIS

>OS12G0566400

MGIGHVLGVLGGALLAHAAYATIQYRAVLKITEEEFSSPPMDVMMQLLLGLALCMWAGLAVPAKFLSVLPHSEENRIVSLPANLDFMIFNHRGRALPSDPDLKLKT

>OS10G0545000

MGRRSGGRKLPFFASNASTSSSTKRTRSARRLPSLTRPRASSSPSPASPSPPPPSASHPAPPSPPLAVSPAGAGKVGKKKAGARLWMRLDRWGVSETLHLDKGSIIRRAGLPPRDLRILGPVFSDSSSILAREKAMVINLEFIRAIVTADEILLLDPLTIDVIPFVEQLTHHLPLKNLVCGNGQPGGDDHGEKHDDSHGDQVPRLNEATGAEHELPFEFQVLELALETVCSSFDVNVSGLERRATPVLEELTKNVSTRNLDRVRTLKSDLTRLLAHVQKVRDEIEHLLDDNEDMAHLYLTRKQLQNQQVEALISSAASNSIVPGGTSLSRLNNSFRRSVSIATSMHLDNDVEDLEMLLEAYFMQLDGIRNRILSVREYIDDTEDYVNIQLDNQRNELIQLQLTLTIASFGIAVNTFIAGAFAMNIQSKLYSIDDGSFFWPFVGGTSSGCFMICIVLLWYARWKKLLGP

>OS04G0430900

MAARRRHVAAGAGAPAPAAGEWAAVTAGGGAAWALSPVEEVGTKQELMRRTGLPPRDLRALDPALSSAASASSCRPSAITGRDRAVVVNLDRARAVITASEVLVPSPRDPAVAPLVRELRARLALAASPTPAPSPSPPQHGMAVGMDGSISPSQASRGGEEAAGNGKDGEALGGGDKALPFEFRALEVCLEFACKSLEHETCTLEKEAYPALDELTSKVSTLNLERVRQIKSRLVAISGKVQKVRDELEHLLDDDMDMAALHLTEKLAYQSSRFDIDKEASELEDHSSRDEEGVEGGGGGDGDDETIAGGGSFSPNTDELEILLESYFVQIDGTLNSLSTLREYVEDTEDYINMMLDEKQNQLLQMGILLSTGTLVSSCAIAVTGVFGINVHISLYDSPASSAAFPCAAAGIVAGSLALYLAALLCYKRAGILQ

>OS04G0501100

MDHDPKERLLLPPRAAAAAAANGPHRRAAPAAGGGGGGVAIDVHGLKRRGGGRRSWVRVDAATGASEAVEVAKPALMRRLDLPARDLRLLDPLFVYPSAILGRERAVVCNLERIRCIITADEALILRDPDVAGGGAETEEAVRRYVAELQRRLVDRADDLPFEFIALEVALEAACSFLDAQAVELEADAYPLLDELTTKISTLNLERVRRLKSKLVALTRRVQKVRDEIEQLMDDDGDMAEMYLTEKKRRMEASLLEEQAFQGMGNSGFGSSFSAPVSPVSSPPASRRLEKELSFARSRHDSFKSADSSQYSIEELEMLLEAYFVVIDYTLSKLTSLKEYIDDTEDFINIQLDNVRNQLIQFELLLTTATFVVAIFGVVSGVFGMNFEVDLFNVPHAFEWTLVITGVCGLVIFCCFIWYFKKRRFFPL

>OS01G0908500

MRPSAAAGGGGGGGGRRKAAAAAAAASREWLVVPASGQARVEEAGKHAVMARTGLPARDLRVLDPLLSYPSTILGRERAIVVNLERVKAVITAAEVLLPNSKDPAFASFVCDLQARVLASSSDQAAEFTDMEGESSAVTSPFPALTSTTPNELEMTNKNSNVVGGMTHSNSMPTLTAAKDGNTKVLPFEFRALEVCLESACRSLEEETSTLEQEAYPALDELTSKISTLNLERVRQIKSRLVAISGRVQKVRDELEHLLDDEMDMAEMYLTEKLTRQEISETSSRVEVDDPSQLEVDRDEDYRSEADVSNGTFIGYKPHIEELEMLLEAYFVQIDGTLNKLSHLREYVDDTEDYINIMLDDKQNQLLQMGVMLSTATVVITAGVAVVGLFGMNIGISLYADPTNEEEKRASNMKFWETTLGTIAGCTVMYIVAMGWGKRSGLLQ

>OS01G0869200

MERRAQPVSAAVAPVTGRRKGAAASRKWMVVPAVGEERRVEFGKHQIMKMTGLPGRDLRVLDPVLSYPSTILGRDRAIVVRLQGVKAIITATEVLVPDHDDVLLASFLLDLRSRLSLPDAAPSTNPAAADRGNGTEQGDQGSVPGLAISGAGNAKIPPFEFKVLEVCLEHACKDLESQTRSLEKEAYPALDKLGSKVSTLNLDHVRNLKSRMVDLSGRVQKIRDELEHLLDDDMDMSEMYLTRKLSFQGLSGSLSRADSHKYASVDHDDDREEEDHDDETESGRESSVYVKPDIEELEMLLEAYFVQIDGTLNTLYHIREYADDTEDYINIMLDEKQNQLLQMGVMLTTATVVVTAGIVVVSLFGMNIHIDLMKDPETPEMVRMSNMHFWETTFGTVAGCIAIYLLAIYAGRKSKILQ

>OS03G0137700

MALPCAFLSAAAAANATSFSSSPESRRCRSVHRVPSRPRPPLAPPARVMGKGNSKRKAANTRLWMRLDRRGGCEMILCDKSFVARRSGLPARDLRVLSPLLSRSPSILAREKAMVINLEFVRAIVTADEVLVLEPLAQEVLPFVEKLRKHFPLKSLDVDDVSTHMHTENQDGELAQDVSCYEVEGANHELPFEFQVLDFALEAVCLSYNSTISDLNRSAIAVLDDLMKSVSTRNLERVWSLKSSLTRLLASVQKVRDEVEHILDDNEAMAHLCTARKTKGQKDLLNTILFPETRLCRTHSSIENSTGIRTCVPSDSDAHILDMLLEAYFKQLDGIRNRIFLVRQYIVDTEDYISIQLDNKRNELLGLQLTLIIASFGIAINTFIAAAFAMNIPHRGYHFVIGVPFGQFVGATSFLCMSIVILLFTYAWRNRLLCT

>OS03G0742400

MAAAVVVAGEAAAAAAAAGAGGKKRGASRSWILFDAAGEERVLDADKYAIMHRVDINARDLRILDPLLSYPSTILGRERAIVLNLEHIKAIITAEEVLLRDPLDDNVIPVVEELRRRLAPSSATQHDVEGAEEDESPFEFRALEVTLEAICSFLGARTTELESAAYPALDELTSKISSRNLDRVRKLKSGMTRLNARVQKVRDELEQLLDDDDDMADLYLSRKLAGAASPVSGSGGPNWFPASPTIGSKISRASRASAPTIHGNENDVEELEMLLEAYFMQIDGTLNKLTTLREYIDDTEDYINIQLDNHRNQLIQLELFLSSGTVCLSLYSLVAGIFGMNIPYTWNDNHGYVFKWVVLVSGLFCAFMFVSIVAYARHKGLVGS

>OS03G0684400

MASVSSSPSYSSQAAVLLLLHQPPHQHGHGGACLRYRGSQSQGRGNAVATSLGLSAAGRGGAGGLLLLPPLPALRAAEGKDGRAVTKDEEEEAAAAAVEEEGEVEVRREEDKPGDDGSREAAARGSGSGRFSADYISLGIREPVYEVIEVKSNGRMSTKKISRRQLLKSSGLRLRDTRSVDPSLWLMNSMPSLLVREQAILVNLGSLRAIAMHERVLIFNYNSPGGKAFLDSLLPRLNPRNINGGPAMPFQLEVVEAALLSRIQRLERRLMRIEPRVGALLEVLPNRLTADVLEQLRLSKQALVELGSRAGDLKQMLIDLLDDPHEIRRICIMGRNCTLDKLSDNMECSVPLEKQIAEEEEEEIEMLLENYLQRCESIHGQAERLLDSAREMEDSIAVNLSSRRLEVSRVELLLQVGTFCVAIGALIAGIFGMNLKSYLETNAWAFWATTGGIVVGAVAGFFIMYSYLKTRKIL

>OS06G0650800

MSAAAASSAAGDSAKQPLLHHQRGNPPHVASVSSPSLPSAPPGALAGGRRFPGGLDVPNLKKRGGGTRSWIRVEAATASVQTLEVDKATMMRRCELPARDLRLLDPLFVYPSTILGRERAIVVNLEQIRCVITADEVLLLNSLDSYVLQYAAELQRRLLQRAEGDELPFEFRALELALEAACSFLDAQAAELEIEAYPLLDELTSKISTLNLERVRRLKSRLVALTRRVQKVRDEIEQLMDDDGDMAEMYLSEKKLRTEASFYGDQSMLGYNSVGDGTSFSAPVSPVSSPTESRKLEKAFSLCRSRHDSVKSSDNTATEHIQELEMLLEAYFVVIDSTLNKLTSLKEYIDDTEDFINIQLDNVRNQLIQFELLLTTATFVVAIFGVVAGIFGMNFETSVFSIQNAFQWVLIITGVIGAFIFCGFLWFFKYKRLMPL

>OS04G0373000

MVMSIDNVRGFALATSSSAFIGSSFVIKKIGLKKAGDAGVRAGSGGYSYLYEPLWWIGMTAMILGEVANFAAYAFAPAILVTPLGALSIIFSAVLAHFILKERLHMFGIVGCILCVVGSVGIVLHAPKEKKIDSVNEIWHLATQPGFIVYSCMAVVVALILIFWVVHRTEQRKMLAYIAICSLMGSLTVISVKAVAIALKLSFNGVNQFIYVPTWFFIVVVVICCLVQLNYLNKALDSFNTAVVSPVYYVMFTILTIIANMIMYKDWASQNATQIATELCGFVTIVAGTFLLHKTRDMGNEQSESSSLRGECELQNH

>OS01G0708300

MWESVALTLAGAAGNNVGKVLQKKGTHILPPLSFKLKVIRAYALNRLWISGFLMDMCGAALMLTALSQAPVSVVQPIAGCGLAILCVFSHFYLKESMNGLDWVAITLAGLGTIGVGVGGEEQKVDKIPLFNIPWLVLSIVILFVLLNTWLHIYKRQRREQELTGPEVIEEIIYGLESGILFGISSVISKTGFVMSEMGFPKIVVPAAISCSVGCSAVGFVYQTRGLKHGRAIVVSTCTSVASIVSGVVAGMIALDEHLPTAPTGRFFLLLGWFFIITGVILLVSSTRIIARLPRSMQKFLKSNVERTHSIRRPSSARGKDPIPSTTIHASTLHLLTSPSKEKA

>OS01G0873700

MSPDATGEAGGGGGGGGDLFAANLKGSLLAVASSAFIGVSFIVKKKGLLRAGAAGSRAGVGGYGYLLEPLWWVGMVTMLVGEIANFIAYMFAPAVLVTPLGALSIIVSAVLAHFTLNEKLQRVGVLGCVLCIVGSTVIILHAPQERTPSSVDEIWHLAIQPDFLCYATAAVAVSLFLMIYCAPRYGQMNIMVYVGICSVIGSLTVMSIKAVGIAIKLTIEGINQAGYFQTWLFAVISITCIAVQLVYLNKALDTFNAAVVSPIYYAMFTTLTILASAIMFKDWSGQSASKIASEICGFLTVLAGTLVLHSTREPDQTLSADLYAPLPPKIYWHIQGNGDIGKQKEDDSLPCDIITVMRQDYFV

>OS01G0882300

MATEASTSAAAGAGGGSWVEGMSADNIKGLVLALSSSFFIGASFIVKKKGLKKAGASGVRAGVGGYSYLYEPLWWAGMITMIVGEVANFAAYAFAPAILVTPLGALSIIISAVLADIMLKEKLHIFGILGCVLCVVGSTTIVLHAPQEREIDSVAEVWALATEPAFLFYAVTVLAATFVLIFRFIPQYGQTHIMVYIGVCSLVGSLSVMSVKALGIALKLTFSGMNQLIYPQTWMFTIVVVACILTQMNYLNKALDTFNTAVVSPIYYTMFTSLTILASVIMFKDWDRQNPTQIVTEMCGFVTILSGTFLLHKTKDMVDGLPPTLPIRIPKHDEDGYAAEGIPLRSAAEGLPLRSPRAAE

>OS02G0518100

MGDWVIGALINIVGSVAINFGTNLLKLGHDQREKLSTTNNNQGNDKFVPKSVMHFQTWRIGILFFAAGNCLNFMSFAYAAQSLLAALGSIQFVSNIAFAYFVLNKTISVKVMVATTFIVFGNIFLVSFGNHQSPVYTPEQLVAKYSNLVFVLYCMSLVFVVAFNQYLYRSGETIISDSAKHTGSHWRTLLPFSYAIVSGAIGSCSVLFAKSLSNMLRLTMSSRYQFHSWFTYSMLLLFLFTAGFWMARLNEGLSLFDAILIVPMFQIAWTFFSICTGFVYFQEYQVFDTLRIVMFVLGMTSVFIGISLLAPDDSKVDTKDGSSATQEPAIDANRPGKMQTEETEVDGTNSFTSSVKVKAKRILSKAKSACSMSLGLGEETISASSVLAMPMVSSRTTGFRGIATDRSKYIPLRSTDWDNL

>OS02G0498300

MITMILGEVANFAAYAFAPAVLVTPLGALSIIFSAVLAHFVLKEKLHMFGVVGCILCVVGSVGIVLHAPKEREIDSIDEIWHLATEPGFIVYSCVAVVSVLFLIFWVAERSGHRKMLVYIAICSTMGSLTVISVKAVAIALKLSFGGSNQFIYIQTWFFIVVVIVCCLVQLNYLNKALDSFNTAVVSPVYYVMFTILTIFANMIMYKDSFSRNATQIATQLCGFVTIVAGTFLLHKTRDMGNEPPLPDDEICLDGGSVRPDRLSQSSS

>OS06G0715700

MGVSDNTVGLSLAVASSAFIGASFILKKIGLIRAGKGGVRAGGGGYTYLLEPLWWAGMMTMLLGEIANFVAYTFAPAVLVTPLGALSIIVSSFLAHFVLKERLEKLGVLGCVSCIVGSVIVVIHAPQEHMPNSVEEIWNLAIQPGFLTYAVATLVVVAALVLFFEPRYGQTNIMIYLGICSSMGSLTVVSIKAIGVAIKLTLDGMNQVAYPHTWLFVIIAIICVVSQINYLNKALDTFDLAVVSPIYYVMFTTLTIVASGIMFKDWAGQSFSSIASEFCGLITILTGTIMLHTAKEEETGSSAALPWPLDRGSISWCISLGSDNLLKNVNEDYFAALQSSPAPV

>OS05G0430700

MSRAPPDAAGDLFAANLKGSLLAVASSAFIGVSFIVKKKGLRRAGAAGPRAGVGGYGYLLEPLWWVGMITMLIGEIANFVAYMFAPAVLVTPLGALSIIVSAVLAHFILNEKLQRMGVLGCVLCIVGSTVIILHAPEEETPSSVEQIWHLATQPAFLCYVAFALVVSLILMAHCAPRYGQTNIAVYIGICSVIGSLTVMSIKAVGIAIKLTIEGINQAGYFQTWLFATVSAICIIIQLIYLNKALDTFNTAVVSPIYYAMFTSLTILASAIMFKDWSGQSISSIASEICGFLTVLSGTVVLHSTREYDQTISPDLYTPLPPIYWHIQGNGETVKQKEDDSLSADFITVVRQDYFV

>OS05G0424800

MGGARLGRWVEGMSADNVKGLLLALSSSLFIGASFIVKKKGLKKAGASGVRAGVGGYSYLLEPLWWAGMTAMIVGEIANFAAYAFAPAILVTPLGALSIIISAVLAHIILREKLHIFGILGCILCVVGSTSIVLHAPPERQIESVAEVWDLATEPAFLLYAAIVLAAAFVLIFHFVPQYGQTHIMVYIGVCSLVGSLSVMSVKALGIALKLTFSGMNQLVYPQMWVFLLFVVACIVTQMNYLNKALDTFNTAVVSPIYYTMFTSLTILASVIMFKDWDRQDPTQIVTEMCGFVTILSGTFLLHKTKDMVDGLPPNLPIRLPKHAEEDGYAAEGIPLRSAADGIPLRSPRATESFRATL

>OS05G0513400

MEGGGGGGQELSTDNVKGIVLALLSSGFIGASFIIKKKGLRRAAVASGIRAGVGGYSYLLEPLWWVGMITMIVGEVANFVAYAFAPAVLVTPLGALSIIVSAVLAHFILNERLHALGVLGCVMCIAGSVVIVIHAPQEQEITSVREIWNMAIQPAFLLYVASVIVVVFVLVFHFSPLYGQSNVLIYTAICSLMGSLSVMSVKALGTSLKLTFEGTNQLVYPETWFFVLIVATCVLTQMNYLNKALDTFNTAIVSPIYYVMFTTLTILASVIMFKDWSGQSLGSITSEICGLIVVLSGTILLHVTKDYERIPQSRSIYAPLSPSLTARLNGDLLKHVEDDRNPDEEKALRRQEMY

>OS11G0197400

MAAPASAAAAAGLAGMSTDNAKGLVLAVSSSAFIGASFIVKKMGLRRAADSGVRAGYGGFSYLMEPLWWIGMISMIVGEIANFAAYAFAPAILVTPLGALSIIISAALAHAILQEKLHTFGILGCVLCVVGSITIVLHAPQERNIDSVREVWDLATEPGFLCYAAIVVAAALVLIYFVVPQHGQTNIMVYIGVCSLLGSLTVMSVKALGIALKLTFSGVNQLFYPQTWAFALIVATCVSTQINYLNKALDTFNTAVVSPIYYVMFTSLTILASVIMFKDWDRQNPTQIVTELCGFVTILSGTFLLHKTKDMTDSTGPSLPTSRSKSASQNRFSIEVVPLKYRDSVDEETLPLSLPKADNRYLMEDFPVRYKDLNIA

>GLYMA.09G019600

MSDTEGQSHSSGITKKLDSHKTYHGRDPNNGNDLWKDGLICAFEYIRGQNRSAKLSSSSSKITDGMHGQHSKMHHVPSDDKKKLSDPSSVNVSRESLFGGSDDDKESQTPKAGQSKKYEGGHWVPIGWARISELVQAVQVDAEWSSHQFEFEYSKDDFTVADLAAPYWEHPTGPIWWCHASAGHPTWLSNAQWLHPAVSLALRDESRLISERMKHLFYETSNVSHACIDLSALFFPTAFYVNFKLFRKSIFGAADEIELKFMNRRNHEDLNLFILILNLEIRKLSTQVIRVKWSLHARNEIVFELLQHLKGNGARNLLEGIKKSTREMIEEQEADRSLRVTHNLAVFGGVGVVLTIITGLFGINVDGIPGAEHTPYAFGVFTAILVFLGVVLIAVGMVYLGLKNPVAEGQVEVRKLELQELVKMFQHEAETHAQVRKNISPKNLPPTAGDGFRSDADYLVIQ

>GLYMA.15G125900

MSDTEGKSHSSGITRKLDSHKTYHGRDPNHGNNLWKDGLICAFEYIKGQNRSVKSSSSSKITDRLHVNGQHSKMHVPSDDKKKLSDPSSVNVSRDSLFGGSDDDKEGQAHKAGQSKKYEGGHWVPIGWARISELVQAVQVDADWSSHQLEFEDSEDDFTVADLAAPYWEHPAGPIWWCHVFAGHPTVEAWLSNAQWLHPAVSLALRDESRLISERMKHLLYEVPVRVAGGLLFELLGQSAGDPLVEEDDIPIVLRSWQSQNFLVTVMHIKGSVSRINVLGITEVQELLSAGGYNMPRTVHEVIALLACRLSRWDDRLFRKSIFGAADEIELKFMNRRNHEDLNLFILILNQEIRKLSTQVIRVKWSLHARDEIVFELLQHLKGNGARTLLEGIKKSTREMIEEQEAVRGRLFTIQDVMQSTVRAWLQDRSLRVTHNLAVFGGVGVVLTIITGLFGINVDGIPGAEQTPYAFGVFTAILVVLGVVLIAVGMVYLGLKNPVVEEQVEVRKLELQELVKMFQHEAETHAQMRKNISPKNLPPTAGDAFRSDADYLVIQ

>GLYMA.02G285600

MVVEALVPLLESNMQAMNEDYSASFTSKMKKEGNHKTYSSTRDSNNNMQQGGELWTNGLICAFEFMRGNGPTKKKDYCLGRIGNSLNESDSHGDDFHLYCKEDLPRRYWRPIGWDRISELVQAVHSGDAQPFDFTDDESDVPVADVATPYWERPVGPTWWCHLDAADPFVTAWFGSSRWLHPAISIALQEESRLISDRMKHLLYEVPVRVAGGLLFELLGQSAGDPFAEEDDIPVVLRAWQAQNFLVTALHVKGSASNINVLGILEVQELLAAGGAKNPCSIHEVVAHLASRLARWDDRLFRKHIFGAADEVELMFMNRRSHEDLHLFTIILNQEIRRLSTQVIRVKWSLHAREEIVFELLKQLRGNAARALLEGVMKSTRQMIGEQEAVRGRLFIIQDVTQSTVRAWLQDRSLTVTHNLGIFGGCGLVLSIITGLFGINVDGIPGSSGTPYAFLLFTMILFVLGVVLIGIGLLYLGLKKPIIEENVALRKQELQELVRMFQHEAETHAQVRKTVPHKAQTAAVRPPNGANHRFIMSKLCSH

>GLYMA.10G180200

MARDGSVVPADPQAMAVVKKKTQSSRSWILFDATGQGTLLDMDKYAIMHRVQIHARDLRILDPLLSYPSTILGREKAIVLNLEHIKAIITAEEVLLRDPTDENVIPVVAELQRRLPRLGAGLKQEGDGKEYLGGQNDAEAAEEDESPFEFRALEVALEAICSFLAARTSELEMAAYPALDELTSKISSRNLDRVRKLKSAMTRLTARVQKVRDELEQLLDDDDDMADLYLSRKAGSASPVSGSGAANWFAASPTIGSKISRASLATVRLEENDVEELEMLLEAYFSEIDHTLNKLTTLREYIDDTEDYINIQLDNHRNQLIQLELFLSSGTVCLSFYSLVAAIFGMNIPYTWNDNHGYMFKWVVIVSGVFSAVMFLIITAYARKKGLIGS

>GLYMA.13G368400

MRRKGVGTTGVKSWMVVSETGHARLEDVGKHSIMRRTGLPARDLRVLDPVLSYPSSILGRERAIVVNLEHVKAIITASEVLLINSSNPFFLSFLQDLHIRLSNLNPSSMSNDMDGGYEEKPLANDSRNGSPVRIPEDSDADFLVRADSLKSSAETGTGTGTGTPAPKPLPFEFKVLEACIESACRCLESETSTLEVEAYPALDELTSQLSTLNLERVRQIKSRLVALSGRVQKVADELEHLLDDDNDMAEMYLTDKLNARLCDQTSLKEGYNSEFEDNDQSDESNSEKYDRFLCPKLDVEELEMLLEAYFEQTNGILQRLTSLSEYVDDTEDYINIMLDDKQNELLQAAIIFDTINMILNAGIVVVGLFGMNIQIDLFNGQPRQFWATTGGTFGGCLLLFLVCLWWGKKRYFLSH

>GLYMA.02G117100

MDETQDHYYSSSLPESSLSHDGGGRSYFNGQINRGTAISGLKKRGHGSRSWIKIGQDGNFQTVTLDKATIMRYCSLPSRDLRLLDPMFIYPSTILGREKAIVVNLEQIRCIITADEVILMNSLDGSVGQYRLELCNRLQNEKADDLPFEFRALELALELTCTSLDAQVNELEMEIYPVLDELASSISTLNLERVRRFKGHLLALTQRVQKVRDEIEHLMDDDGDMAEMCLTEKKRRSDTCTFNDCFQTRASGRLISKSAPASPERTISGVQMLQRAFSSIGNSSKHGSSMGSSDNGERIEPLEMLLEAYFIVIDNTLNTILSLKEYIDDTEDFINIKLGNIQNQLIQFELLLTAATLVAAVFAAVAGVFGMNFETTVFDYPSGFHWVLVITGIACIALYFALLFYFRYKKVLAA

>GLYMA.03G159400

MALASSVVELQPSSVKKKTAVSRSWILLDHYGKGTVLDADKYAIMRLVQIHARDLRILDPLLSYPSTILGREKVIVLNLEHIKAIITADEVLLRDPMDDDVVPIVEELRRRLPQVSAAEQGQGKEEACAQDGEGGEENEFPFEIRALEALFEAICSFLDARTRELETSAYPALDELISKISSRNLDRVRKLKCAMTRLTIRVQKIRDELESLLDDDDDMADLYLSRKLDASSSPTSSSDAPYWLYGSPNTGSKRHKSSRVSGTTVQRENDVEELEMLLEAYFMQIDGTLNKLATLREYIDDTEDYINIQLDNHRNQLIQLELFISVGTVCMSLYSLVAAIFGMNIPYTWKAPGHEHVFKWVVIFGGMVCASLFLSIVSYARRKGLVGS

>GLYMA.20G210300

MARGDGSVVPTDPQTMAVVKKKTQSSRSWILFDATGQGSLLDVDKYAIMHRVHIHARDLRILDPLLSYPSTILGREKAIVLNLEHIKAIITAEEVLLRDPTDENVIPVVEELQRRLPQLSATGLQQQGDGKEYLGGQNDAEAAEEDESPFEFRALEVALEAICSFLAARTTELEMAAYPALDELTSKISSRNLDRVRKLKSAMTRLTARVQKVRDELEQLLDDDDDMADLYLSRKAGSASPVSGSGAANWFAASPTIGSKISRASRASLATVRLDENDVEELEMLLEAYFSEIDHTLNKLTTLREYIDDTEDYINIQLDNHRNQLIQLELFLSSGTVCLSFYSLVAAIFGMNIPYTWNENHGYMFKWVVIVSGVFSAVMFLMITAYARKKGLVGS

>GLYMA.05G168200

MGKGPFSFRRSASRRRPKKTAAPPPPPSPPQPPYAAGIATSPDDNNNRLIAAGAGSSALTKAKKKTGGARLWMRFDRSGRSELVELEKNAIIRHAAIPARDLRILGPVFSHSSNILAREKAMVVNLEFIKAIVTAEEVLLLDPLRQEVLPFVEQLRQQLPGKSQPKLLGGVEEQEGEMQVSNGRQWLPMPEAADGLQSELPFEFQVLEIALEAVCTYLDSNVADLERGAYPVLDELARNVSTKNLEHVRSLKSNLTRLLARVQKVRDEIEHLLDDNEDMAQLYLTRKWLQNQQFEEAHLGATTSNNFPNTSRSVRRLGSNRSESLVTCHYEDDNNVEDLEMLLDAYFMQLDGTRNKILSVREYIDDTEDYVNIQLDNHRNELIQLQLTLTIASFAIAIETMIAGAFGMNIPCNLYHIDGVFWPFVWITSAACVLLFLLILAYARWKKLLGS

>GLYMA.08G126600

MGKTPFSFRRSASRRRPKKTAAPPPPPSPPQHPYAAGFAASPDDNNRLIAAAAGSSALTKAKKKTGNVRLWMRFDRSGRSELVELEKNAIVRHAAIPARDLRILGPVFSHSSNILAREKAMVVNLEFIKAIVTAEEVLLLDPLRQEVLPFVEQLRQQLPGKSQPKLLGGTEEQEGEMHVSNGRQWLPTPEAADGLQSELPFEFQVLEIALEAVCTYLDSNVADLERGAYPVLDELARNVSTKNLEHVRSLKSNLTRLLARVQKVRDEIEHLLDDNEDMAQLYLTRKWLQNQQFEEAHLGATTSNNFPNTSRSVRQLGSIRSESLVTSHYEDDNNVEDLEMLLDAYFMQLDGTRNKILSVREYIDDTEDYVNIQLDNHRNELIQLQLTLTIASFAIAIETLIAGAFGMNIPCNLYNIDGVFWPFVWTTSAACVLLFLLILAYARWKKLLGS

>GLYMA.17G227100

MWESILLTVAATAGNNIGKILQKKGTIILPPLSFKLKVIRSYALNKTWVVGFLIDIFGALLMLRALSLAPVSVIQPVSGCGLAILSIFSHFYLKEVMNAVDWVGITLAGFGTIGVGAGGEEQEVVALSIFHIPGLAFIVFILFILLSGWLRICKRQRREQEMMEYDVVEEVIYGFESGILFGMSSVISKMGFLFLEQGFPKLLVPMCIMISVCCSGTGIYYQTRGLKHGRAIVVSTCAAVASILTGVLAGMLALGERLPSEPKARLALLLGWLLIIVGVILLVGSTRLVRFLSCSSRQKRSNVEKNFGLRGATSSRVREPSPSAVIQAATLNHLLSSSSKEKA

>GLYMA.04G005200

MGIAENSKGLVLAVASGVFIGASFVLKKKGLKQAATHGTRAGVGGYSYLLQPLWWAGMLTMLIGEVANFVAYIYAPALLVTPLGALSIIVSAVLAHFLLKEKLQKMGILGCVFCIVGSVLIVIHAPQEHALNSVQEIWDLATQPLFLVYVAAAVSVVLALILHFEPRYGQTNMLVYLGICSLIGSLLVMSTKAIGIAIKLTLEGTSQLTYPQTWFFLTVTVICIITQLNYLNKALDTFNTAIVSPVYYVMFTTLTIIASVIMFKDWSEQSAGSIASEICGFVIVLSGTILLHATREQEQSNKQGSLTWYIGEDLVKRIEDGHLNLLHGSDYVEK

>GLYMA.02G280800

MGLSKENLKGLILALVSSGFIGASFIIKKQGLRRAAAVSGVRAGVGGYYYLLEPLWWVGMITMIVGEVANFVAYAFAPAVLVTPLGALSIIVSAVLADIILKEKLHNLGILGCIMCIAGSIIIVIHAPKEQPITSVLEIWNMATQPAFLAYVGSVIVLVFILVFHFAPRCGHTNVLVFTGICSLMGSLSVMSVKALGTSLKLTFEGKNQLIYPETWFFMLVVAICVIMQMNYLNKALDTFNTAIVSPIYYVMFTTLTILASVIMFKDWDGQSGGTIVSEICGFIIVLSGTIMLHATKDFERSSSFRGSDPLSPTLSARLFTGNGDSLLKQDEENGSPESNRRQELY

>GLYMA.02G068000

MGEWIVGAFINLFGSIAINFGTNLLKLGHNERERHLLGSDGVNGKMNLKPIIYFQSWRIGIVFFFLGNCLNFISFGYAAQSLLAALGSVQFVSNIAFAYFVLNKMVTVKVLVATAFIVLGNVFLVAFGNHQSPVYTPEQLTEKYTNISFLLYLLALISIVALHHSIYKRGELLLGVSGHDLRPYWSMLLPFSYAVVSGAVGSCSVLFAKSLSNLLRLALSNGYQLHSWFTYSMLLLFLSTAGFWMTRLNEGLSLFDAILIVPMFQIAWTLFSICTGFIYFQEYQVFDALRTTMFMLGMMCVFIGISLLAPDESKVSGPETKDSSLDSMVSSAISTEANRLVVSPEEAQNKDTRSLVKAILIKITDLLVKAKTTCALSLGFGEDTINASSVLVMPMMSSRMTGFRGNGLERARILSMRNGWRKIPMDEDAGKLLETSSVVPPSP

>GLYMA.05G153000

MGASSDNVTGFVLAVCSSVFIGSSFIIKKMGLKKAGATGKRAGAGGHAYLYEPWWWFGMISMIVGEVANFAAYAFAPALLVTPLGALSIIFSAILAHFILKERLHIFGVLGCALCMVGSTTIVLHAPHERVIHSVKEVWQLATEPGFLIYMCIVVVVVCILIFYCAPRYGTTYLVIYVGICSLTGSITVMSVKAVSIAMKLTLEGNNQFIYFQTWFFTIIVIGCCLLQINYLNKALDTFNTAVVSPIYYVMFTSFTIFASIIMFKEWDTQDASQIATEVCGFITILSGTFLLHKTKDMGNRPIESPVFVSTPQNVSSHSGT

>GLYMA.05G196600

MSSSNLTGFVLAVLSSAFIGSSFIIKKKGLQLASANGPRASVGGYGYLLQPLWWVGMITMIVGEIANFVAYIYAPAVLVTPLGALSIIVSAVLAHFLLKEKLQKMGMLGCLLCIVGSTVIVLHAPEEKSLSSVQEIWELAIQPAFLSYTASAIAVTLFLVLYCAPRHGQTNILVYTGICSIVGSLTVMSVKAVGIAIKLTLEGANQAFHFQAWVFAMVSVTCIIVQLNYLNMALDNFNTAVVSPIYYALFTSFTILASAIMFKDYSGQSISSIASELCGFITILSGTTILHSTREPDPPVVADLYTPLSPKVSWYIQGNSEPWKQEEDVSPLNLIAIIRQDHFK

>GLYMA.11G255400

MGKTHDNVVGLILAISSTVFIGSSFIIKKMGLKKAADHGNRAATGGHSYLYEPWWWAGMISMIAGEIANFAAYAFAPAILVTPLGALSIIFSSVLAHFILKEKLHIFGVLGCALCVVGSTSIVLHAPKEKDIHSVKEVWELATGPGFIVYICAIVILVCVLHFRFVRSHGQTHMMVYLGICSPTGSITVMGVKAVGIALKLTFEGTNQFVYFETWIFTVVVIGCCLLQINYLNKALDAFSTAVVSPVYYVMFTSFTIVASIITFKEWAKQDSTQIATELCGFVTILSGTFLLHRTKDMGNKPSDASVHSSPEDNNSNTKTPLSNQI

>GLYMA.11G105300

MVSVVLPLIIHFEPHYGQTNMLVYLGICSLVGSLTVVSIKAIGIAIKLTLDGISQIVYPQTWFFLTVAIICVITQLNYLNRALDTFNATIVSPVYYVMFTTLTIIATAIMIGPGQDISSIASEICGFITVLTGTIILHMTREQEESNMQKTFTWFIGEDLMKDVENEHLILIHDSDYLER

>GLYMA.14G097400

MWESVVLTVAATAGNNIGKILQKKGTIILPPLSFKLKISLCWQVIRSYALNKTWVVGFLMDILGALLMLRALSLAPVSVIQPVSGCGLAILSIFSHFYLKEVMNAVDWVGITLAGFGTIGVGAGGEEQEVVALSIFHIPGLAFVVFILFILLSGWLRICKCQRREQEMVEYDVVEEVIYGLESGILFGMSSVISKMGFLFLEQGFPKLLVPMCIMISVCCSGTGFYYQTRGLKHGRAIVVSTCAAVASILTGVLAGMLALGERLPSEPKARLALLLGWLLIIVGVILLVGSTRLVRFLSCSSQRKRSNVDKNFDLRRATSSRVRETSPSAVIQAATLNHLLSSSSKEKA

>GLYMA.14G033700

MGLSKENLKGLILALVSSGFIGASFIIKKQGLRRAAAVYGVRAGVGGYYYLLEPLWWVGMITMIAGEVANFVAYAFAPAVLVTPLGALSIIVSAVLADIILKEKLHNLGILGCIMCIAGSIIIFIHAPKEQPITSVLEIWNMATQPAFLAYVGSVIVLVFILVFHFAPRCGHTNVLVFTGICSLMGSLSVMSVKALGTSLKLTFEGKNQLIYPETWFFMLVVAICVIMQMNYLNKALDTFNTAIVSPIYYVMFTTLTILASVIMFKDWDGQSGGTIVSEICGFIVVLSGTIMLHATKDFERSSSFRGSAPSSPTLSARLFTGNGDSLLKQDEENGSPESNMCSRRQELY

>GLYMA.12G168000

MATSSSSSSTSSWREGMSSDNIKGLCLALSSSFFIGASFIVKKKGLKKAGASGIRAGSGGYSYLYEPLWWVGMITMIVGEIANFAAYAFAPAILVTPLGALSIIISAALAHIILRERLHIFGILGCVLCVVGSTTIVLHAPQEREIESVSEVWDLAMEPAFLFYAALVITATFILIFHFIPLYGQTHIMVYIGVCSLVGSLTVMSVKALGIVIKLTLSGMNQLIYPQTWAFTLVVIVCVLTQMNYLNKALDTFNTAVVSPIYYVMFTTFTIVASVIMFKDWDRQSPTQVITEICGFVTILSGTFLLHKTKDMADGLQPSLSVRLPKHSEEDGFDGGEGIPLRRQEAP

>GLYMA.12G030100

MRGERKKVKNENIRVACEGMSHRPTPVQQFSVTKFDLSFFLSLAGMTETGVSDNFKGLILAMGSSAFIGSSFILKKKGLKRAAARGTRAGVGGYTYLLEPLWWAGMVTMIIGEIANFVAYIYAPAVLVTPLGALSIIVSAVLSHFLLKERLPKMGVLGCVSCIVGSIVIVIHAPQEQTPSSVQEIWDLATQPVSVVLALIVHFEPRYGQTNMLVYLGICSLVGSLTVVSIKAIGIAIKLTLDGISQIAYPQTWFFLTVATICVITQLNYLNRALDTFNATIVSPVYYVMFTTLTIIASAIMFKDWSGQDVSSIASEICGFITVLTGTIILHMTREQEESNMQKTSTWFIGEDLMKGVENEHLIRIHDSDYLER

>GLYMA.06G053100

MWESIVLTVVATAGNNIGKILQKKGTVILPPLSFKLKVIRAYALNKTWLIGFVMDIFGALLMLRALALAPVSVIQPVSGCGLAILSVFSHFYLKEVMNIVDWVGITLAGFGTIGVGAGGEEQEAAALSIFHIPWLAFVVFILFIMLNGWLRIFKRNRREQEMMEYDVVEEIIYGLESGILFGMASVISKMGFLFLEQGFPKLLVPICIIISVCSSGTGFYYQTRGLKHGRAIVVSTCAAVASILTGVLAGMLALGERLPSAPKARLLLLLGWLLIIVGVILLVGSTKLVRFFRFSSHRFKNYGPRRSGTSRVREPSPTAVIQAATLNHLLSSSSKEKA

>GLYMA.06G159100

MYSTNLIGFILAVVSSAFIGSSFIIKKKGLQRASLNGSRASGGGYGYLLQPLWWLGMVTMIVGEIANFVAYVYAPAVLVTPLGALSIIVSAVLAHFMLNEKLQKMGMLGCLLCIVGSTVIVLHAPQEKPLSSVEEIWQLALQPAFLLYTASTIAVAFFLILYCAPRFGQTNILVYIGICSIIGSLTVMSIKAIGIAIRLTIEGADQFVQFQTWIFTMVAISCIITQLNYLNMALDTFNTAVVSPIYYALFTSFTILASAIMFKDYSGQSISSIASELCGFITVLSGTTVLHSTREPDPPVNTDLYSPLSPKVSWYIQGNGEPWKQKEEDGPPFNLITVIRQDHFK

>GLYMA.06G005000

MSMRGRFRGGNENDEKVVKCVESVRVIVMGIAENSKGLVLAVASGVFIGASFVLKKKGLKQAATHGTRAGVGGYSYLLQPLWWAGMLTMLIGEVANFVAYIYAPALLVTPLGALSIIVSAVLAHFLLKEKLQKMGILGCVFCIVGSVLIVIHAPQEHALNSVQEIWDLATQPLFLVYVAAAVSVVLALVLHFEPRYGQTNMLVYLGICSLIGSLLVMSTKAIGIAIKLTLEGTSQLTYPQTWFFLTVTVICIITQLNYLNKALDTFNTAIVSPVYYVMFTTLTIIASVIMFKDWSDQSAGSIASEICGFVIVLSGTILLHATREQEQSNKQGSLTWYIGEDLVKSIEDGHLNLLHGSDYVEK

>GLYMA.06G208700

MAFLFYAALVITAIFILIFHFIPLYGQTHIMVYIGVCSLVGSITVMSVKALGIVIKLTLSGMNQLIYPQTWAFTLVVIVCVLTQMNYLNKALDTFNTAVVSPIYYVMFTTFTIVASVIMFKVSFVT

>GLYMA.18G091200

MIMLLSIFATTQEHNLTTNQEHNFSTAAFLFYAALVITVTFILIFHFIPLYGQTHIMVYIGVYSLIGSITVMSVKALGIVIKLTMSGMNQLIYPQTWAFSLVVIVCVLTQMNYLNKAVDTFNAAVVSPIYYVMFTAFTIVASVIMFKGFIAYALNYGLITW

>GLYMA.16G003900

MATSSSSSSWREGMSSDNIKGLCLALSSSFFIGASFIVKKKGLKKAGASGIRAGSGGYSYLYEPLWWVGMITMIVGEIANFAAYAFAPAILVTPLGALSIIISAALAHIILRERLHIFGILGCVLCVVGSTTIVLHAPQEREIESVSEVWDLAMEPAFLFYAAMVITATFILIFHFIPLYGQTHIMVYIGVCSLVGSLTVMSVKALGIVIKLTLSGMNQLIYPQTWAFTLVVLVCVLTQMNYLNKALDTFNTAVVSPIYYVMFTTFTIVASVIMFKDWDRQSPTQVITEICGFVTILSGTFLLHKTKDMADGLQTSLSIRLPKHSEEDGFDGGEGIPLRRQESMRLP

>GLYMA.16G149500

MGEWIVGAFINLFGSIAINFGTNLLKLGHNERERHLLGSDGVNGKMNLKPIIYFQSWRIGIVFFFLGNCLNFISFGYAAQSLLAALGSVQFVSNIAFAYFVLNKMVTVKVLVATAFIVLGNVFLVAFGNHQSPVYTPEQLTEKYTNIAFLLYLLALISIVALHHSIYKRGELLFAVSGHDLRPYWSMLLPFSYAVVSGAVGSCSVLFAKSLSNLLRLAMSNGYQLHSWFTYSMLLLFLSTAGFWMTRLNEGLSLFDAILIVPMFQITWTFFSICTGFIYFQEYQVFDALRTTMFILGMMCVFIGISLLAPDESKVSGPETKDSSLDSMVSSAMSTETSRLVVSPEEAQNKDSRSFVKAILIKVTDLLVKAKTSCALSLGFGEDTINTSSVLVMPMMSSRMTGFRGNGLERARILSMRNGWSKIPMDEDAGKLLETSSVVPPSP

>CA16933

MGKTATARGGRRRKGGGTAVKKSWMVVSETGESHVEDVDKHYIMRRTGLPARDLRVLDPALFHPSSILGRDKAIVVNVECVKAIITANQVFMINSTDPFFIRFLQDLQQRVPPNNNRTPSRTSNEMDGDCEEKPLLQDGSPLLQSGIDSNPPPEIFDHGTPISNIAVTTAPKKLPFEFRALEACIESACSVLEFETQRLEEETYPALDELTSKISSLNLDIVDQIEHLLDDDNDMAEMYLTQKLKASVSDLASVTEEYNSEVEDIDESDDSRSVRDKSYGIKPDVEELEMLLEAYFAQINGILQKLTSLSEYVDDTEDYINIMLDDKRNQLLQV

>CA08687

MGGKKKGLFSFRRRSHKKLPVPPHSPQPTLEISGSPSDNRLVTKAKKKTGGARLWMRFDRSGRSELVEWEKNTIIRHAAIPARDLRILGPVFSQSSNILAREKAMVVNLEFIKAIVTAEEVLLLDPLRQEVLPFVEQLRQQLPHKTQPKLLGGGGGDEQESEMQVSSSRQWLPVPEATEGLHSELPLPFEFQVLEIALEVLCTYLDSNVADLEKGAYPVLDELARNVSTKNLEHVRSLKSNLTRLLSRVQKVRDEIEHLLDDNEDMAQLYLTRKWLQNQQLEAHLGATSSNNLSNSAHVVRRLSSTRSASIVTSNDDNDVEDLEMLLEAYFMQLDGTRNKILSVREYIDDTEDYVNIQLDNHRNELIQLQLTLTIASFAIAFETLIAGAFGMNIPCTLYNQDGIFWPIVAGMTAVSIVLFLLVLAYAKWKKLLGS

>CA11051

MGVSENYKGLILAVCSSGFIGASFILKKKGLKRAASRGTRAGGGGYTYLLEPLWWAGMITMITGEAANFVAYIYAPAVLVTPLGALSIIVSSVLAHFLLKERLQKMGVLGCVSCIVGSIVIVIHAPQEHTPNSVQEIWELATQPDFMMYAAATVSVVLVLILNFEPRYGQTNMLVYLGICSLMGSLTVVSIKAIGIAIKLTLDGISQIAYPQTWFFVIVAVICVITQLNYLNKDWSGQDVSSIASEICGFITVLSGTIILHGTKEQEESTRQGSLTWFIKEDSIKCVEDEHLIVINGSDYLQN

>CA08544

MGLKKAGTTGKSAATGGHAYLYEPWWWFGMISMIVGEIANFAAYAFAPAILVTPLGALSIIFSAVLAHFILKERLHIFGVLGCVLCMVGSTTIVLNAPHERIIHSVKEVWQLATEPGFIVYTLAVVIMVSVLIIYCVPRYGQRHLVVYVGICSLTGSLTVMGVKAVGIAMKLTFEGTNQFTYFQTWFFTLAVIGCCLLQINYLNKALDTFNTAVVSPVYYVMFTSFTIFASMIMFKDWATQNKSQIATEFCGFVTILSGTFLLHKTKDMGDKPTENSSLDRATIINNTDT

>CA16924

MISTNLTGLVLAMISSAFVGSSFIIKKKGLQLARVNGPPASVGGYGYLLQPLWWVGMVTMIVGEIANFVAYIYAPAVLVTPLGALSIIVSAVLAHFLLKEKLLKMGMLGCLLCIIGSIIIVLHAPEEMSLSSVQQIWKLAIQPAFLSYTASAIAVTLFLVLYCAPRFGQTNILVYIGICSIIGSLTVMSVKAIGIAIKLTFEGANQFFYFQTWVFTMVAITCVIAQLNYLNMALDNFNTAVVSPIYYALFTAFTILASAIMFKDYSGQSISSIVSELCGFITVLSGTTLLHSTKEPDPPVNPDLYTPLSPRVSWYIQGNNESWKQKEEDVSPFNLIAIIRQDHFK

>CA16930

MVEEEMGKTARGVRRKGGGTAVKKSWMVVSETGESHVEDVDKHSIMRRTGLPARDLRVLDPALFHPSSILGRDKAIVVNVECVKAIITANQVFMINSTDPFFIRFLQDLQQRVPPNNNRTPSRTSNEMDGDCEEKPLLEDGSPLLQSGIDSNPPPEIFDHGTPISNIAVTTAPKKLPFEFRALEACIESACSVLEFETQRLEEEAYPALDELTSKISSLNLDIVDQIEHLLDDDNDMAEMYLTQKLKASVSDLASVTEEYNSEVEDIDESDDSRSVRDKSYGIKPDVEELEMLLEAYFAQINGILQKLTSLSEYVDDTEDYINIMLDDKRNQLLRVSITLNTINMIANAGIVVVGLFGMNIHIDLFDGQPRQFWATTVGTLVGCVLLFLVYVWWGKQRYLLFQ

>CA16931

MGKTATARGGRRRKGGGTAVKKSWMVVSETGESHVEDVDKHSIMRRTGLPARDLRVLDPALFHPSSILGRDKAIVVNVECVKAIITANQVFMINSTDPFFIRFLQDLQQRVPPNNNRTPSRTSNEMDGDCEEKPLLQDGSPLLQSGIDSNPPPEIFDHGTPISNIAVTTAPKKLPFEFRALEACIESACSVLEFETQRLEEETYPALDELTSKISSLNLDVAEMYLTQKLNASVSDLASVTEEYNSEVEDINESDDSRSVRDKSYGIKLDVEELEMLLEAYFAQINGILQKLTSLSEYVDDIEDYINIMLDDKRNQLLQVSITLNTINMIVNAGIVVVGLFCMNIHIDLFDGKPRQFWATTAGTLVGCILLFLVSVWWGKKRYLLSQ

>CA16932

MVEEEMGKTARGVRRKGGGTAVKKSWMVVSETGESHVEDVDKHSIMRRTGLPARDLRVLDPALFHPSSILGRDKAIVVNVECVKAIITANQVFMINSTDPFFIRFLQDLQQRVPPNNNRTPSRTSNEMDGDCEEKPLLEDGSPLLQSGIDSNPPPEIFDHGTPISNIAVTTAPKKLPFEFRALEACIESACSVLEFETQRLEEETYPALDELTSKISSLNLDRVRHIKNRLVTLSGRVQKIADQIEHLLDDDNDMAEMYLTQKLKASVSDLASVTEEYNSEVEDIDESDDSRSVRDKSYGIKPDVEELEMLLEAYFAQINGILQKLTSLSEYVDDTEDYINIMLDDKRNQLLRVSITLNTINMIANAGIVVVGLFGMNIHIDLFDGQPRQFWATTVGTLVGCVLLFLVYVWWGKQRYLLFQ

>CA25207

MARDGSVVPADPQAMVVVKKKTQTSRSWILIDAMGQGSMLDVDKYAIMHRVQINARDLRILDPLLSYPSTILGREKAIVLNLEHIKAIITAEEVLLRDPTDENVVPVVEELQRRLPKLSDIHQQQGDGKEYIGCQHDAEAADEDESPFEFRALEVALEAICSFLAARTYELELIAYPALDELTSKISSRNLDRVRKMKSAMTRLNARVQKVRDELEQLLDDDDDMADLYLSRKAGSVSPVSGSGAANWFATSPTNGSKISRASRASIATARFDENDVEELEMLLEAYFMQIDSTLNKLNTLREYIDDTEDYINIQLDNHRNQLIQLELFLSSGTVCLSFYSLVAGIFGMNIPYSWNDNHGYMFKWVVIVSGAFSAIMFLMITIYARKKGLIGS

>CA12138

MARDGSVVPADPQAMVVMKKKTQTSRSWILIDATGQGSMLDVDKYAIMHRVQINARDLRILDPLLSYPSTILGREKAIVLNLEHIKAIITAEEAMVVVKKKTQTSRSWILIDAMGQGSMLDVDKYAIMHRVQINARDLRILDPLLSYPSTILGREKAIVLNLEHIKAIITAEEVLLRDPTDENVVPVVEELRRRLPKLSDIHQQQGDGKEYIGGQHDAEAADEDESPFEFRALEVALEAICSFLAARTYELELIAYPALDELTSKISSRNLDRVRKMKSAMTRLNARVQKVRDELEQLLDDDDDMADLYLSRKAGSVSPVSGSGAANWFATSPTNGSKISRASIATARFDENDVEELEMLLEAYFMQIDSTLNKLNTLREYIDDTEDYINIQLDNHRNQLIQLELFLSSGTVCLSFYSLVAGIFGMNIPYSWNDNHGYMFKWVVIVSGAFSAIMFLMITIYARQKGLIGS

>CA05656

MGMRKIGRKGSMARTWLVMSMEGKREMVEAGRPLIMRWTGLTARDLRVLDPLLSYPSMIVGRERAMVLNLEHIKAIITAHEVLLLNSTDPSVMPFVNHLHSRILYQHLLQEDPYGKDKRGMTKNTIPFEFVVLEACLEEVISSLENETNIFEQEAHPALDKLTSKISTVNLERVRLIKSRLVALTSRVQRVRDELEHLLDDDGDMAELYLTDKLVQQKFENSSTASSMNNSINGTPNEITLEHEDDENDVDTNRGEISGTQAGSVYSHVTNVEELEMILGAYFVQIGGTLNKLFALREYVEDTEDYIDITLDDTQNRILQTGVKIGTASVVLNMFINVTGIFGMNIHIGLYDYGGMPEFIGVIFGCTAVCIILYVFAMVWYKKKRLLE

>CA15493

MAEGTLKSILRKTSFSPTNNWIKLDGNGHSSILDIDKYEIMRLVRIDARDLRIVDPLLAYPSTILSREEVIVLNLEHIKAIITAKEVFLQDPTSKDVAPIVKELQRKLFTTGTNERDVQDNGPLDVQVDEEDESPFEFRALEIFLEAICSFLDARVGELEIDTYPTLDELTKKVSSRNLDKVRKLKSAMTRLTARVQKVKEEIEHLMDDDEDMADLYLSRKLVDASSSPISESVAGNWFASSPTIKSKSVATFHRDDENNVDELEMLLEAYFMQIDDTLNRLTTLRGYIDDTEDYINIHIDNHRNQLIQLELLLNAGELSLAVYAVVTGILGMNLPFSWTKDHDYMFKWVVIFTGVFTFFLFLIIVAYARKKGLVGS

>CA11617

MARDGSPVPADPQAMVVVKKKTQTSRSWILIDAMGQGSMLDVDKYAIMHKIQINARDLRILDPLLSYPSTILGREKAIVLNLEHIKAIITAEEVS

>CA11616

MARDESVVPADPQAMVVVKKKTQTSRSWILIDAMGQGSMLDVDKYAIMHRVQINARDLRILDPLLSYPSTILGREKAIVLNLEHIKAIITAEEVLLRDPTDENVVPVVEELQRRLPKLSDIHQQQGDGKEYIGGQHDAEAADEDESPFEFRALEVALEAICSFLAARTYELELIAYPALDELTSKISSRNLDRVRKMKSAMTRLNARVQKVRDELEQLLDDDDDMADLYLSRKAGSVSPVSGSGAANWFATSPTNGSKISRASRASIATARFDENDVEELEMLLEAYFMQIDSTLNKLNTLREYIDDTEDYINIQLDNHRNQLIQLELFLSSGTVCLSFYSLVAGIFGMNIPYSWNDNHGYMFKWVVIVSGAFSAIMFLMITIYARKKGLIGS

>CA02933

MADLKERLLPPKPLPAFNGREATNRPSPSGRQPIQVVDLTGLKKRGQGLRSWIRVDTSGNSQVLEVDKFTMMRRCDLPARDLRLLDPLFVYPSTILGREKAIVVNLEQIRCIITADEVLLLNSLDSYVLHYVMELQRRLTTTGVGEVWQSDNSDSNRWRGSANFESTYSNTSPDYLPFEFRALEVALESACTFLDSQPETSFVETRCRVFRISCDNLLEKATELEIEAYPLLDELTSKISTLNLERVRRLKSRLVALTRRVQKVRDEIEQLMDDDGDMAEMYLTEKKRRMELSFYGDQSIGYRSVDGASISAPVSPVSSPPDSRRLEKSLSIARSRHESSESNTENIEELEMLLEAYFVVIDSTLNKLTSLKEYIDDTEDFINIQLDNVRNQLIQFELLLTTATFVVAIFGVVAGVFGMNFEIPLFNVPSAFQWAFYQTLSLPSQNQNQYAPFDFNLDLDDPDLLQFLQFLKKDDDHSLTSLEPNRDLVCSAIWVLREDWKPALRAFKWNSRYNDEKACNLMIWVLGTHAKFSTAWSIIRDMHASCLSTHHAMLIIIDRYAAANNSAKAIETFNFMNNFRLTPDQEAFRALLTALCKYGNVEEAEEFMLVNKKLFPLEIESFNIILNGWCNITNDVYEAKRVWRDMSKYCIMPDATSYSHMISCFSKEGNLFDSLRLYDQMKKREWIPGIEVYNSLVYVLTRENCPKEALKTIDKLREQGLQPDSATFNSMILPLCEAGKLAMARIVLNTMVEENISPTVETYHAFFEGTDYHGTIEFLSRMKGSGLGPNKDSFLLILEKFLKLKQPVNALKIWAEMKKYDVVPSCIHYRKMVEGLVTCRWFIKARDFYEEMISNGCSEDPKLNKLIQKHVLNSGDKRKQDVRKANSDKV

>CA00038

MASSSSSSSSSSSSSSSTSSWREGMSSDNIKGLVLALSSSFFIGASFIVKKKGLKKAGASGIRAGSGGYSYLYEPLCAALAHIILRERLHIFGVLGCALCVVGSTTIVLHAPQEREIESVPEVWDLAMDPAFLFYAALVITATFVLIFHVIPQYGQTHIMVYIGVCSLVGSLSVMSVKALGIAIKLTLSGMNQLIYPQTWALDTFNTAVVSPIYYVMFTTLTIVASVIMFKDWDRQSPTQVITEICGFVTILSGTFLLHRTKDMTDGLQGSSIRLNKHSEEDGLDDGEGIPLRRQDST

>CA07962

MSDAENQLYSLGNKWKPDSYKTYHGKDPNPGNDLWTDGLICAFEFVRGQKRPVTSRSASKIVNRLHFDCQHSKMRTPSNGLTEASSTRPHKKKLSRGSLFDASDDDKEGQVLQAGQSNAPEKHEGNHWVPIGWSRISELVQAVQVDAVWSSHQFEFEDSEDDFTVADLAAPYWERPAGPIWWCHVSAGHPSVESWLGNAQWLHPAVNLALRDESRLISERMKHLLYEVPVRVAGGLLFELLGQSVGDPLVEEDDIPIVLRSWQAQNFLVTVMHVKGSVSRINVLGITEVQELLSAGGYNVPRTVHEVIAQLACRLSRWDDRLFRKSIFGAADEIELKFVNRRNHEDLNLFVIILNQEIRKLSTQVIRVKWSLHARDEIVFELLQHLKGNGARNLLEGIKKTTREMIEEQEAVRGRLFTIQDVMQSTVRAWLQDRSLRVTHNLAVFGGVGVVLTIITGLFGINVDGIPGAENTPYAFGVFTAILIFLGAVLIVVCLVYLGLKKPIAEDQVEVRKLELQELVKMFQHEAETHAQVRKNVSRNNLPPTAGDAFCRDADYLVIR

>CA07013

MSLTLTCPHQQHFRFQPSLTRTKYFFSDSRTLLRRREISVTSPPALKPVKCLSRSTEEKQWSDAETVASDSDEVVDDSGNGQTPSRTSSVESQKIVTTSSGDSLSLGIREPVYEVVEVKSDGTVSTRKINRKQLLKSSGLRPRDIRSVDPSLFLTNSMPSLLVREYAILLNLGSLRAIAMKDCVLIFDYTRKGGQAFLESLLPRLNPKNNNGGPSMPFELEVVEAALLSRIQRLERRLMDLEPRVQALLEALPNRITGDILEQLRTSKQTLVELGSRAGALRQMLLDLLEDPNEIRRICIMGRNCTLNKGNNNVECSVPLEKQIADEEEEEIEMLLENYLQRCESCHGQAERLLDSAREMEDSIAVSLSSRRLEVSRVELLLQVGTFCVAIGALVAGIFGMNLRSYLEERVFAFWLTTAGIIIGGIIVFFLMYNYLRERKIF

>CA11902

MRGGNVTGNGNGNEDRWGTTGTAIRKKGTGVRAWLVVDGTGEAQVVEAGKHAIMRRTGLPARDLRILDPLLSYPSTVLGRERAIVINLEHIKAIITANEVLLLNSRDPSVTPFVNELQARILRHHNATTAPHPDNQDESHGGGIKILPFEFVALEACLEAACSVLENEAKTLEQEAHPALDKLTSKISTLNLERVRQIKSRLVAITGRVQKVRDELEHLLDDDEDMAEMYLTEKILQQQLEQTSSSEEEKEEEEDVDVNHDHDHDDDHVDIHQRAQGAEISSEAGIGGGESNEDADDQNQNSGEEMYGGALNIISRDSRGTRASTTYSAATNKLDVEELEMLLEAYFVQIDGTLNKLSTLREYVDDTEDYINIMLDDKQNHLLQMGVMLTTATLVVSAFVVVAGIFGMNIRIELFDPNLYGMREFLWTVGGGTAGTIFLYVVAIAWCKHKRLLE

>CA12201

MAVSGSETELRVQATMIKTTISRSWVLLDRDGRDTVLDVDKYAIMRLVEINARDLRILDPLLSYPSTILGREKVIHIKAVITADEVLVRDPMDDDVIPVVEELRRRLPLKVSFGGQDQGEEESSIGEQNEFPFEFRALEVVLEAICSFLDARTRELETTAYPALDELTSKISSRNLDKVRKLKSAMTRLTNRVQKIREELENLLDDDDDMAELYLSRKLGVSSSPSSSSSGPNWHQNSPNQGSKIHKSSRGSATTLQEENDVEELEMLLEAYFVQIDGTLNKLTTLREYIDDTEDYINIQLDNHRNKLIQLELFLSSGTVCLSIYSLVVAIFGMNIPYTWREDHGYVFKWVVIFTGMACSSVFLSVVSYARRKGLVGS

>CA19137

MGEWVVGAFINLFGSIAINFGTNLLKLGHNERERHLLGNDGVHGKVTLKPIIYFQSWRIGILFFFLGNCLNFISFGYAAQSLLAALGSVQFVSNLAFAYFVLNKLVTVKVLVATAFIVLGNVFLVAFGNHQSPVYTPEQLTEKYTNAAFLLYLLALIVIVALHHSVYKRGELQLAVSGHDLKPFWSMLLPFSYAVVSGAIGSCSVLFAKSLSNLLRLAMSNGYQLHSWFTYSMLLLFLSTAGFWMTRLNEGLSLFDAILIVPMFQIAWTFFSICTGFIYFQEYQPDFDARTCGLCIFDPATDLSFSFPSQVFPSLLLSHLEVGTCFPWLVPCCISHKPSGSSSSTDMGKMCHVNLIFLKAGETKDSSLDSGVSPAISTEMNRLIVSSEEAHNKDPRSFVNGVLVKITNMWVKAKTSCALSLGFGEDTINASSVFVMPMMSSRMNGFRGNGLERARILSMRNSGWSKVPMDEDAVKLLDTNPIVPPSP

>CA15254

MVVEALVPLLEYDMQGVNEDYSASLDNEVKEQENHKVRCSSKDNLHTKELWTDGLICAFEFIRSSRKVHSSVAVREVAKKKDFQGSQINSLKRNPGGNGGFQESSLPVDESGGLDLEDFNNSNCFGKEGYPRSYWKPIGWARVSELVQAVHSDASWASQLHDFTDDESDVPVADVATPYWERPVGPTWWCHLNAADPYVATWLASSQWLHPAISIALHDESRLISDRMKHLLYEVPVRVAGGLLFELLGQSAGDPFAEENDIPIVLRAWQAQNFLVTVLHVKGSASNINVLGILEVQEMLAGGGANIPRSIHEVVAHLACRLARWDDRLFRKHVFGAADEVELMFMNRRNQEDLHLFTIILNQEIRRLSTQVIRVKWSLHAREEIMFELLQQLRGNVTRSLLAGVMKSIRQMIEEQEAVRGRLFTIQDVMQSAVRAWLQDRSLTVTHNLGVFGGCGLVLSIITGFFGINVDGIPGSATPYAFTIFSVILVVVGAVLIGIGLLYLGLKKPIVEENVTVRKLELQELVRMFQHEAESHAQVRKTVPHKDVPQTAAAVRPPNGASRRFMISKLFSP

>CA10167

MSLLKENVTGLILALVSSAFIGSSFIIKKQGLRRAASVFGVRAGVGGYYYLLEPLWWVGMITMIVGEVANFVAYAFAPAVLVTPLGALSIIVSAVLADLILKEKLHKLGVLGCVMCIAGSIIIVIHAPKEDPISSVLQIWNMATQPAFLAYVGSVVVLVFILVFHFAPRCGHTNVLVYTGICSLMGSLSVMSVKALGTSLKLTIEGKNQLVYPETWFFMLVVAICVVMQMNYLNKETHLPGHGYNPIA

>CA18568

MEETHGQYYLSGLPESSLSHDSSERYDFNRQVNRGAGISGLKKRGHGTRSWIKIDQNGNSETVTLDKATIMRHCSLPSRDLRLLDPMFIYPSSILGREMAIVVNLEQIRCVITADEVILMNSLDGTVGQYRSELCNRLRREKSDDLPFELRALELALELTCTSLDAQVNELEMEIYPVLDELASSISTLNLERVRRFKGHLLALTQRVQKVRDEIEHLMDDDGDMAEMCLTEKRKRSDTYPSNDCFQTRTLSGNMTSKSAPVSPERSLSGVQMLQRTFSSFGNSSKYGSLSGSTDNDERIQPLEMLLEAYFIAIDHTLNTLLSLKEYIDDTEDFINIKLGNIQNRLIQFELLLTAATLVAAVFATVAGIFGMNFETSVFDYSTGFHLVLVVTGIACVSLYFALLFYFRYKKVLPA

>CA18570

MTSKSAPVSPERSLSGVQMLQRTFSSFGNSSKYGSLSGSTDNDERIQPLEMLLEAYFIAIDHTLNTLLSLKEYIDDTEDFINIKLGPSLDTLSITGKYSKPPDTV

>CA11407

MWISSDNIIGLVLAISSSIFIGSSFIIKKMGLKKAANNGNRAATGGHSYLYEPQWWVGMSSMIIGEIANFAAYAFAPAILVTPLGALSIIFSAVLAHFILNERLHIFGVLGCVLCMVGSTTIVLHAPHEKDIHSVKEVWQLATEPGFLVYSCGVVALVLVLIFCFAKKYGHSHMIIYVGICSLTGSITVMSVKALGIALKLTFEGMNQFKYFETWFFTIVVLGCCLLQINYLNKALDTFNTAVISPVYYVMFTSFTIIASTIMFKEWDTQNASQIATELALIFEACEQHFSFNYVPSQEAKHTKLATVWF

>CA04249

MYSSNLTGFILAVVSGAFIGSSFIIKKKGLQRASLNGTPASVGGYGYLLQPLWWIGMFTMIVGEIANFVAYIYAPAVLVTPLGALSIIVSAVLAHFMLGEKLQKMGMLGCLLCIVGSTVIVLHAPQEKSLSSVLEIWLLAVQPAFLLYTASAMAVAFFLILYCAPRYGQTNIFIYVGICSIIGSLTVMSVKAIGIAIKLTLEGADQFVYFQTWIFTMVAVSCIITQLNYLNMALDTFNTVVVSPIYYALFTSFTILASAIMFKDYSGQSISSIASELCGFITVLSGTTVLHSTREPDPPAGTGKGLMHLGEDSFDDVRSA
