# Supplementary material for: Genome-wide identification and functional characterization of magnesium transporter (MGT) gene family in soybean (Glycine max L.) and their expression profiles in response to aphid infestation, dehydration, and salt stresses
Source: PLoS One. 2025 Aug 29;20(8):e0330440. doi: 10.1371/journal.pone.0330440 (PMC12396710; doi:10.1371/journal.pone.0330440)
Supplement: S2 Data — (S2 Data.DOCX) [file pone.0330440.s002.docx]

>GLYMA.02G068000

ATGGGGGAGTGGATTGTTGGAGCTTTCATCAACCTCTTTGGTAGTATTGCAATAAACTTTGGGACCAATCTTCTCAAACTAGGGCATAATGAGAGAGAAAGACATTTACTTGGAAGTGATGGGGTAAATGGAAAGATGAATCTGAAGCCTATTATATATTTCCAGAGTTGGAGAATTGGCATTGTATTTTTCTTTCTTGGAAATTGCCTTAATTTCATTTCCTTTGGGTACGCTGCTCAGTCACTTCTTGCAGCGCTGGGATCTGTTCAGTTTGTATCTAACATTGCCTTCGCTTACTTTGTCTTGAACAAAATGGTGACAGTCAAGGTACTGGTTGCCACAGCTTTCATTGTTCTTGGGAATGTTTTTCTAGTTGCTTTTGGCAATCACCAATCGCCTGTTTATACGCCAGAGCAGTTGACAGAGAAATATACCAATATTTCATTCCTTCTATACCTTCTAGCTTTGATCTCAATTGTTGCCTTGCATCACTCCATCTACAAGAGGGGAGAACTTCTGCTTGGAGTATCAGGACATGACCTCAGACCCTATTGGAGCATGCTACTGCCCTTCTCATATGCTGTAGTTTCAGGGGCTGTAGGTTCATGCTCAGTGTTGTTTGCTAAATCACTTTCTAACCTATTACGACTGGCTTTGTCCAATGGTTATCAGTTGCACAGCTGGTTCACGTATTCCATGCTTCTTTTATTTCTTAGTACTGCTGGATTTTGGATGACCAGGTTGAATGAAGGACTGTCATTGTTTGATGCAATTCTTATTGTTCCCATGTTTCAGATAGCATGGACTTTGTTCTCAATATGTACAGGATTTATATATTTTCAAGAATATCAGGTATTTGATGCATTAAGGACAACAATGTTTATGCTTGGAATGATGTGTGTGTTTATTGGCATTTCTTTGCTGGCACCTGATGAATCAAAAGTTTCAGGTCCTGAGACTAAAGATAGTTCTTTGGATTCCATGGTGTCTTCTGCCATATCTACAGAAGCTAACAGGCTGGTAGTGTCTCCTGAAGAAGCACAAAACAAAGATACGAGATCATTAGTTAAAGCAATACTAATAAAGATTACAGATTTGTTGGTAAAGGCAAAGACTACTTGTGCATTGTCTCTTGGTTTTGGGGAGGATACCATCAATGCATCATCAGTTCTTGTGATGCCAATGATGTCATCGAGAATGACTGGATTCAGAGGTAATGGGCTTGAGAGAGCAAGAATTTTGTCCATGAGAAATGGTTGGAGAAAGATCCCAATGGATGAAGATGCTGGGAAATTGCTTGAAACTAGTTCGGTTGTTCCTCCTAGCCCTTAA

>GLYMA.02G117100

ATGGATGAAACACAAGACCACTATTATTCTTCCAGCCTACCCGAGTCTTCTTTATCTCATGATGGTGGTGGGAGGTCTTATTTCAATGGGCAAATAAATCGTGGGACTGCTATATCAGGCCTGAAGAAAAGAGGTCATGGAAGTCGCTCTTGGATTAAAATTGGTCAGGATGGGAATTTTCAGACTGTGACACTTGACAAGGCAACTATAATGAGATATTGTTCTTTGCCTTCTAGAGATCTCCGGCTGTTGGATCCGATGTTCATTTATCCTTCTACAATATTAGGACGGGAGAAGGCTATTGTAGTCAACCTTGAGCAAATCCGGTGTATAATCACTGCTGACGAGGTCATCCTAATGAATTCATTGGATGGTAGTGTTGGTCAGTATAGGTTAGAATTATGCAACCGGCTTCAGAATGAAAAAGCGGATGATCTACCTTTTGAATTTAGGGCACTGGAGTTGGCTCTAGAATTGACATGCACATCTTTAGATGCTCAGGTAAATGAACTGGAAATGGAAATATATCCTGTGCTGGATGAACTAGCCTCATCTATCAGTACTCTAAATCTGGAACGTGTTCGAAGATTTAAAGGTCACTTGCTTGCTTTGACTCAACGAGTTCAGAAGGTTCGTGATGAAATAGAACATCTCATGGATGATGATGGTGACATGGCTGAGATGTGCCTAACTGAGAAAAAGAGAAGATCGGATACTTGCACTTTTAATGATTGTTTTCAAACTCGTGCATCAGGTAGACTAATTTCAAAGTCGGCTCCTGCTTCACCAGAGCGAACAATTAGTGGAGTCCAGATGTTGCAAAGGGCTTTCAGCAGCATTGGAAATTCTAGTAAACATGGTAGTTCAATGGGTTCGTCTGATAATGGGGAAAGGATTGAGCCACTGGAAATGTTGCTTGAAGCATATTTTATTGTCATTGATAATACTCTTAACACGATATTGTCGCTCAAAGAATACATTGACGACACAGAAGATTTTATCAACATAAAATTGGGAAATATTCAAAACCAGCTAATACAGTTTGAGTTGCTTCTTACAGCAGCTACATTGGTAGCTGCAGTATTTGCTGCTGTAGCAGGAGTATTTGGAATGAACTTTGAAACCACAGTTTTTGACTATCCATCGGGATTCCATTGGGTTTTGGTAATTACTGGAATTGCTTGTATAGCATTGTATTTTGCCCTCCTATTCTATTTTAGGTACAAGAAAGTGCTCGCAGCTTAA

>GLYMA.02G280800

ATGGGTCTGTCCAAGGAGAATCTGAAAGGTCTCATACTAGCTTTGGTGTCAAGTGGGTTCATTGGGGCAAGTTTTATCATTAAGAAGCAAGGCCTTAGAAGAGCTGCAGCAGTTTCTGGTGTCAGGGCTGGTGTTGGTGGTTATTATTATCTCTTGGAGCCACTATGGTGGGTGGGAATGATCACAATGATTGTAGGAGAGGTTGCAAACTTCGTTGCATATGCATTTGCTCCAGCAGTCCTAGTTACTCCTCTTGGTGCATTAAGTATTATTGTGAGTGCTGTTTTGGCTGACATTATTCTGAAAGAGAAGCTACACAATCTTGGGATATTGGGCTGTATAATGTGCATTGCTGGTAGTATCATTATTGTTATTCATGCTCCTAAGGAACAACCTATTACATCTGTTCTGGAAATATGGAATATGGCCACTCAACCAGCTTTTCTGGCATATGTGGGCTCAGTAATAGTGTTGGTTTTCATTCTGGTCTTCCATTTTGCACCAAGATGTGGGCATACGAATGTGCTAGTTTTTACTGGAATTTGTTCATTGATGGGTTCCCTCTCTGTGATGAGTGTTAAAGCCCTTGGAACTTCTTTGAAATTAACTTTTGAAGGGAAAAATCAGTTAATCTACCCAGAGACATGGTTTTTTATGTTAGTTGTTGCTATATGTGTCATCATGCAAATGAATTATCTTAATAAGGCTCTTGACACCTTCAACACAGCAATTGTATCTCCTATATACTATGTCATGTTCACAACACTTACAATACTAGCCAGTGTAATAATGTTTAAGGATTGGGATGGACAAAGTGGTGGAACTATTGTGTCAGAAATATGTGGCTTCATCATTGTGCTCTCTGGAACAATAATGTTGCATGCGACTAAGGACTTCGAGAGAAGCTCTTCTTTTAGAGGTAGTGATCCTTTATCGCCTACGCTATCTGCCCGACTTTTTACCGGAAATGGTGACTCATTACTTAAGCAAGATGAGGAAAATGGATCTCCCGAGAGTAATATGCGCTCAAGAAGGCAAGAATTGTATTAG

>GLYMA.02G285600

ATGGTTGTGGAGGCTCTAGTTCCTCTTCTTGAGAGCAACATGCAAGCTATGAATGAAGACTATTCAGCTTCATTTACCAGTAAAATGAAGAAGGAGGGGAATCACAAAACCTATTCTTCCACCAGAGATAGTAACAATAACATGCAGCAGGGTGGGGAACTTTGGACTAATGGACTTATTTGTGCTTTTGAATTCATGAGGGGAAATGGACCTACCAAGAAGAAGGATTATTGCTTAGGGAGAATTGGAAACAGTTTGAATGAATCAGATTCACATGGAGATGATTTTCATTTGTATTGCAAAGAGGATCTTCCAAGGAGATACTGGAGACCAATTGGTTGGGATAGAATTTCTGAACTTGTTCAGGCAGTGCATAGTGGTGATGCACAGCCGTTTGATTTCACGGATGATGAGAGTGATGTTCCTGTTGCAGATGTAGCGACTCCTTACTGGGAACGCCCGGTGGGGCCCACGTGGTGGTGTCATTTGGATGCTGCTGATCCTTTTGTTACTGCCTGGTTTGGTAGTTCTCGCTGGTTGCATCCTGCCATCAGCATCGCTTTGCAAGAAGAAAGTAGATTAATAAGTGATAGAATGAAACACCTTCTGTACGAGGTTCCAGTTCGAGTTGCTGGAGGATTATTATTTGAACTTCTAGGGCAGTCTGCTGGTGATCCATTTGCTGAGGAAGATGACATTCCCGTTGTCTTACGGGCATGGCAGGCACAAAACTTTTTGGTGACTGCTTTGCATGTTAAGGGCTCTGCTTCAAATATCAATGTGTTAGGTATTTTAGAAGTTCAGGAACTGCTGGCTGCAGGAGGTGCTAAAAATCCATGCAGCATTCATGAGGTTGTAGCACACCTAGCTAGCCGACTTGCACGATGGGATGATAGGTTGTTTCGTAAACACATCTTTGGGGCTGCAGATGAAGTTGAATTGATGTTTATGAACAGGAGGAGCCATGAGGATCTGCATCTGTTCACTATAATACTGAACCAAGAAATTAGAAGATTATCAACCCAGGTTATAAGAGTGAAATGGTCACTCCATGCAAGAGAGGAAATTGTTTTTGAACTTCTTAAACAGTTGAGAGGAAATGCAGCAAGAGCATTGCTGGAAGGAGTAATGAAGAGTACAAGGCAAATGATTGGGGAGCAAGAAGCAGTTCGAGGTCGCTTATTCATAATCCAGGATGTGACGCAAAGCACAGTCCGGGCATGGTTGCAGGACAGAAGCCTCACAGTTACCCATAACTTGGGAATATTCGGGGGTTGTGGCCTTGTTCTTTCCATCATAACTGGACTATTTGGCATAAATGTGGATGGAATACCTGGATCTTCAGGAACTCCTTATGCCTTTCTTCTATTCACCATGATCCTCTTTGTGCTAGGGGTCGTGCTAATTGGAATTGGATTGCTTTACCTTGGCCTGAAAAAACCCATCATTGAAGAAAATGTTGCACTGAGAAAGCAAGAGCTTCAAGAGCTAGTCAGGATGTTTCAACATGAAGCAGAAACTCATGCACAAGTACGGAAAACGGTGCCTCACAAGGCTCAAACTGCAGCAGTTCGGCCACCAAATGGTGCAAATCATCGTTTCATAATGTCAAAGTTGTGCAGTCACTAG

>GLYMA.03G159400

ATGGCTCTTGCTAGCTCTGTGGTTGAGCTTCAACCCTCTTCAGTGAAGAAGAAAACTGCAGTTTCCAGAAGTTGGATTCTGCTGGACCACTATGGCAAGGGCACTGTTCTGGATGCCGATAAGTATGCTATCATGCGCCTGGTTCAAATTCACGCAAGAGATCTTCGGATTCTCGACCCTCTCTTATCTTATCCTTCCACCATTTTGGGCAGAGAGAAAGTCATTGTTCTCAATTTAGAGCACATTAAAGCTATCATCACTGCAGATGAGGTGTTACTAAGAGACCCAATGGATGATGACGTTGTGCCAATTGTTGAGGAACTTCGACGACGGTTACCCCAAGTAAGTGCTGCTGAGCAAGGCCAAGGAAAAGAAGAGGCTTGTGCTCAAGATGGTGAAGGTGGAGAAGAAAATGAATTTCCATTTGAGATACGGGCTTTGGAAGCTTTATTTGAGGCAATTTGTAGTTTTCTTGACGCACGAACGAGAGAATTAGAGACTTCTGCTTATCCAGCTTTGGACGAACTGATCTCTAAGATCAGCAGTCGTAATTTGGATAGAGTGCGAAAATTGAAATGTGCAATGACGAGGTTGACAATTCGAGTTCAAAAGATTAGAGACGAACTAGAAAGCCTACTTGATGATGACGACGACATGGCTGATCTTTACTTATCAAGAAAATTGGATGCTTCATCCTCTCCAACTAGTAGCTCTGATGCTCCATACTGGCTTTATGGGTCTCCAAATACAGGTTCAAAAAGACACAAATCAAGCAGAGTTAGTGGAACAACAGTTCAAAGGGAGAATGATGTTGAGGAGCTCGAAATGTTACTTGAGGCCTATTTCATGCAAATTGACGGCACATTAAATAAATTGGCCACATTGCGAGAATATATCGATGACACAGAAGATTACATCAACATACAGCTGGACAATCACAGAAATCAATTGATTCAGCTAGAGCTCTTCATCAGTGTCGGGACTGTCTGTATGTCCTTATATTCATTGGTGGCTGCAATATTTGGTATGAACATACCATATACATGGAAAGCACCAGGCCACGAACATGTGTTTAAATGGGTGGTGATCTTTGGGGGAATGGTTTGTGCATCCTTGTTTTTATCCATTGTATCGTATGCTCGACGCAAAGGCCTTGTTGGGTCTTGA

>GLYMA.04G005200

ATGGGGATTGCAGAGAACTCAAAGGGTTTGGTTCTTGCTGTGGCATCGGGTGTGTTCATAGGAGCAAGCTTCGTCCTCAAAAAGAAGGGTCTTAAGCAGGCTGCCACCCACGGCACTCGCGCAGGAGTTGGCGGCTATTCTTATCTACTCCAGCCACTTTGGTGGGCTGGCATGCTTACAATGCTTATTGGGGAGGTTGCAAATTTTGTCGCTTATATATATGCTCCTGCACTTCTCGTTACTCCCCTTGGTGCACTAAGTATTATTGTCAGTGCTGTTTTGGCTCACTTCTTGCTCAAGGAAAAGCTTCAGAAGATGGGCATTTTGGGATGTGTCTTCTGCATTGTGGGTTCAGTTCTCATTGTCATCCATGCACCTCAAGAGCATGCTTTAAATTCTGTCCAAGAAATATGGGATCTCGCCACTCAACCGTTATTTCTAGTTTATGTGGCGGCGGCAGTTTCGGTAGTTTTAGCCTTGATTTTGCATTTTGAACCTCGCTACGGACAGACAAATATGCTGGTTTACTTGGGAATTTGCTCGTTAATTGGCTCACTTTTGGTTATGAGCACAAAAGCCATAGGGATCGCAATCAAGCTTACATTGGAGGGAACAAGTCAATTAACATATCCTCAAACTTGGTTTTTTCTTACCGTGACTGTCATATGTATCATTACACAGTTGAATTACCTGAACAAGGCACTGGATACATTTAACACCGCAATTGTCTCTCCCGTATATTATGTCATGTTCACAACTCTGACCATTATTGCTAGTGTGATAATGTTTAAGGATTGGTCTGAGCAGAGTGCAGGTAGCATAGCCTCTGAGATATGTGGATTTGTCATTGTTCTTTCAGGAACAATCTTATTGCATGCGACAAGAGAACAAGAACAATCCAACAAACAAGGGTCCTTAACATGGTACATTGGTGAGGACTTGGTAAAGCGCATTGAGGATGGACACCTGAACCTTTTACATGGTTCAGATTATGTTGAAAAGTGA

>GLYMA.05G153000

ATGGGGGCGTCTTCGGACAACGTAACTGGGTTTGTTTTGGCTGTCTGTTCCAGCGTTTTCATTGGCTCTAGCTTCATAATAAAAAAGATGGGCCTTAAAAAGGCTGGTGCCACAGGGAAAAGAGCAGGCGCGGGAGGACATGCATACCTATATGAACCTTGGTGGTGGTTTGGAATGATCTCTATGATTGTTGGGGAAGTAGCCAATTTTGCAGCTTATGCATTTGCTCCTGCACTACTTGTAACTCCTTTGGGAGCTTTAAGTATCATTTTCAGTGCAATACTAGCTCACTTTATCTTAAAAGAGAGGTTGCACATATTTGGTGTGCTTGGATGTGCTCTTTGTATGGTGGGATCTACAACTATTGTATTGCATGCTCCCCATGAAAGAGTTATTCACTCTGTTAAGGAAGTGTGGCAACTTGCTACAGAACCAGGCTTTCTAATCTACATGTGTATAGTTGTGGTTGTGGTTTGCATCCTTATTTTCTATTGTGCTCCACGATATGGGACAACCTATCTGGTTATATATGTTGGAATATGCTCTCTCACAGGCTCAATTACGGTTATGAGTGTGAAAGCAGTGTCAATAGCTATGAAGTTAACATTGGAAGGCAACAACCAATTCATTTACTTTCAGACCTGGTTCTTTACGATTATTGTGATAGGATGTTGTCTTTTGCAGATTAACTACTTGAACAAGGCTTTGGACACCTTTAACACTGCAGTTGTATCACCAATTTACTATGTCATGTTTACATCATTCACCATCTTTGCCAGCATAATCATGTTTAAGGAATGGGACACACAAGATGCATCTCAAATTGCTACCGAGGTTTGTGGCTTTATCACAATTTTATCTGGGACCTTTCTTCTTCACAAAACCAAAGATATGGGAAATAGACCCATAGAGTCTCCTGTTTTTGTAAGTACTCCACAAAATGTTAGTAGTCACTCAGGGACTTAA

>GLYMA.05G168200

ATGGGTAAGGGTCCCTTTTCCTTCCGCCGCTCCGCCTCTCGCCGCCGACCCAAGAAGACCGCCGCCCCGCCTCCTCCTCCGTCTCCGCCGCAGCCTCCCTACGCGGCCGGAATCGCCACCTCACCCGACGACAACAACAACCGTCTCATTGCTGCCGGCGCCGGCAGCAGCGCCTTGACGAAGGCGAAGAAGAAGACCGGCGGCGCCCGCTTGTGGATGAGGTTTGACCGGTCGGGCCGGTCAGAACTGGTGGAGTTGGAGAAGAACGCCATCATCCGCCACGCAGCGATTCCCGCTAGAGACCTGAGAATCTTGGGCCCCGTCTTCTCCCACTCCTCCAACATCCTCGCTAGAGAGAAAGCAATGGTGGTTAATTTGGAGTTTATAAAGGCAATCGTGACTGCTGAAGAAGTGTTATTGCTTGATCCTCTTCGGCAGGAGGTTCTTCCCTTTGTTGAGCAACTCAGGCAACAGCTTCCTGGGAAAAGTCAACCTAAACTTCTCGGCGGCGTGGAGGAACAGGAAGGCGAAATGCAGGTCTCTAATGGCAGGCAATGGTTGCCTATGCCGGAGGCGGCTGATGGTTTGCAGTCAGAGCTTCCGTTTGAGTTTCAAGTTCTGGAGATTGCTTTGGAGGCTGTGTGTACTTATCTGGACTCGAATGTGGCGGACCTTGAGCGAGGTGCTTACCCTGTGTTGGATGAATTGGCTCGGAATGTTAGCACCAAGAATCTTGAACATGTGAGGAGTTTGAAGAGTAATCTTACGCGGTTGCTGGCAAGAGTGCAGAAGGTGCGAGATGAAATTGAACATCTGTTAGATGACAACGAAGATATGGCACAACTATATTTGACGAGGAAGTGGTTACAGAATCAACAATTTGAGGAGGCTCATTTGGGTGCCACAACCTCAAATAACTTTCCTAATACCTCACGTTCTGTTCGTCGACTTGGTTCTAACAGAAGTGAAAGTCTTGTGACCTGCCATTATGAGGATGATAACAATGTGGAGGACTTGGAGATGTTGCTTGATGCATATTTCATGCAGTTGGATGGAACTCGTAACAAAATATTATCTGTTAGGGAGTATATTGACGACACTGAAGACTATGTCAACATTCAACTTGATAACCACCGAAATGAACTTATTCAGCTGCAGTTGACGTTGACTATTGCATCATTTGCTATTGCTATTGAAACTATGATTGCTGGTGCATTTGGTATGAACATTCCTTGTAACTTGTATCACATTGATGGAGTATTTTGGCCCTTCGTTTGGATCACGTCTGCAGCTTGCGTATTGCTTTTCTTGCTTATTTTAGCATATGCAAGATGGAAGAAGTTGCTGGGATCATAA

>GLYMA.05G196600

ATGAGCTCCAGCAATTTGACGGGGTTTGTGTTGGCCGTGCTTTCCAGCGCTTTCATTGGCTCCAGCTTCATCATCAAGAAAAAGGGTCTCCAACTCGCCAGTGCCAATGGCCCACGTGCCAGTGTTGGTGGCTATGGCTACTTGCTTCAACCTCTCTGGTGGGTCGGAATGATTACCATGATTGTTGGAGAGATTGCTAATTTTGTAGCCTACATTTATGCCCCTGCTGTTCTTGTTACTCCACTCGGTGCTTTGAGCATTATTGTTAGTGCTGTCTTGGCACATTTTCTCTTGAAGGAGAAGCTGCAGAAAATGGGCATGTTGGGGTGTCTTCTGTGCATTGTGGGATCAACTGTTATTGTGTTGCATGCACCCGAAGAGAAATCTCTTAGTTCTGTACAAGAAATATGGGAATTGGCCATTCAACCTGCATTCCTCTCGTACACTGCCTCAGCAATTGCCGTGACATTGTTCTTGGTTTTGTATTGTGCTCCCCGTCATGGCCAGACTAATATTTTGGTTTATACTGGAATATGCTCGATAGTTGGGTCCTTGACAGTCATGAGTGTAAAAGCAGTTGGCATTGCGATAAAACTTACACTGGAAGGTGCAAACCAGGCTTTCCACTTTCAGGCATGGGTTTTTGCAATGGTTTCTGTCACCTGCATCATTGTCCAACTAAATTACCTTAATATGGCATTGGATAATTTTAACACAGCAGTTGTTTCTCCAATCTATTATGCATTGTTCACTTCTTTTACAATATTGGCCAGCGCAATCATGTTTAAGGACTATTCTGGTCAAAGTATAAGCAGTATTGCATCAGAGCTATGTGGTTTCATCACTATTTTATCTGGAACGACTATATTGCACAGTACAAGAGAGCCAGATCCTCCAGTCGTTGCAGATTTATATACACCATTGTCTCCAAAAGTGTCATGGTATATCCAAGGCAACAGCGAACCCTGGAAACAGGAGGAGGATGTGTCACCCTTGAATTTAATTGCGATTATACGGCAAGACCATTTCAAGTGA

>GLYMA.06G005000

ATGAGTATGAGAGGAAGGTTCCGCGGAGGAAATGAGAATGATGAAAAAGTGGTGAAGTGTGTAGAGAGCGTTAGAGTGATAGTGATGGGGATTGCAGAGAACTCAAAGGGTTTGGTTCTTGCTGTGGCATCGGGTGTGTTCATAGGAGCAAGCTTCGTCCTCAAAAAGAAAGGTCTTAAGCAGGCTGCTACCCACGGCACTCGCGCAGGAGTTGGCGGCTATTCTTATCTACTCCAGCCACTTTGGTGGGCTGGCATGCTTACAATGCTTATTGGTGAGGTTGCAAATTTTGTCGCTTATATATATGCTCCTGCACTTCTCGTTACTCCCCTTGGTGCACTAAGTATTATTGTCAGTGCTGTTTTGGCTCACTTCTTGCTCAAGGAAAAGCTTCAGAAGATGGGCATTTTGGGATGCGTCTTCTGCATTGTGGGTTCAGTTCTCATTGTTATTCATGCACCTCAAGAGCATGCTTTAAATTCTGTCCAAGAAATATGGGATCTCGCCACTCAACCATTATTTCTAGTTTATGTGGCGGCAGCAGTTTCGGTAGTTTTAGCCTTGGTTTTGCATTTTGAACCTCGCTACGGACAGACAAATATGCTGGTTTACTTGGGAATTTGCTCATTAATTGGCTCACTTTTGGTTATGAGCACAAAAGCCATAGGGATCGCAATCAAGCTTACATTAGAGGGAACAAGTCAATTAACATATCCTCAAACTTGGTTTTTTCTTACCGTCACTGTCATATGTATCATTACACAGTTGAATTACCTGAACAAGGCACTGGATACATTCAACACAGCAATTGTCTCTCCCGTATATTATGTCATGTTCACAACTCTGACCATTATTGCTAGCGTGATAATGTTTAAGGATTGGTCTGATCAGAGTGCAGGCAGCATAGCCTCTGAGATATGTGGATTTGTAATTGTTCTTTCTGGAACAATCTTATTGCATGCGACAAGAGAACAAGAACAATCCAACAAGCAAGGGTCCTTAACATGGTACATTGGTGAGGACTTGGTAAAGAGCATCGAAGATGGACACCTGAACCTGTTACATGGTTCAGATTATGTTGAAAAGTGA

>GLYMA.06G053100

ATGTGGGAATCAATTGTTTTGACGGTGGTTGCCACTGCCGGCAACAACATCGGCAAAATCCTTCAGAAGAAGGGCACTGTCATTCTTCCCCCTCTCTCTTTCAAGCTCAAGGTCATAAGGGCATATGCTTTGAACAAAACCTGGCTCATAGGTTTTGTAATGGATATATTTGGGGCACTGTTGATGTTAAGGGCATTAGCTCTTGCTCCTGTCTCCGTCATCCAACCAGTTTCTGGCTGTGGATTGGCAATTCTCTCAGTCTTTTCTCATTTTTATCTCAAGGAAGTTATGAATATTGTTGATTGGGTGGGCATTACCTTGGCAGGTTTTGGCACAATAGGAGTTGGTGCTGGAGGTGAGGAGCAAGAGGCTGCTGCTCTATCTATATTTCACATACCGTGGCTGGCATTTGTTGTTTTCATCTTGTTTATAATGCTTAATGGATGGCTTCGCATATTCAAACGCAATCGAAGAGAACAAGAGATGATGGAATATGATGTTGTTGAGGAAATTATTTACGGCTTAGAATCTGGAATTTTGTTTGGGATGGCATCTGTAATATCAAAGATGGGGTTTCTGTTCTTGGAGCAAGGCTTCCCCAAGCTGTTGGTTCCTATTTGCATCATCATAAGTGTCTGTTCAAGTGGTACAGGCTTTTACTACCAGACACGTGGTCTAAAACATGGGCGGGCTATTGTAGTTTCTACATGTGCCGCAGTGGCATCAATTTTGACTGGTGTACTTGCTGGGATGCTTGCTTTGGGTGAGCGACTCCCTTCGGCCCCAAAAGCTCGCTTGTTACTTCTTCTTGGATGGCTACTTATAATTGTTGGCGTGATTTTACTTGTTGGTTCTACAAAGCTAGTGAGATTCTTTCGATTTTCTTCACACCGCTTTAAGAATTATGGCCCTAGAAGATCTGGAACTTCCCGTGTTAGGGAACCTAGCCCAACTGCTGTCATTCAAGCAGCGACCTTAAATCATTTACTCTCATCATCTTCCAAAGAAAAAGCTTGA

>GLYMA.06G159100

ATGTACTCCACCAATTTGATCGGTTTCATTCTGGCCGTCGTCTCCAGCGCCTTCATCGGCTCCAGCTTCATCATCAAGAAAAAAGGCCTCCAACGTGCCAGTCTCAACGGCTCACGTGCCAGTGGCGGAGGCTACGGTTACTTGCTGCAACCTCTTTGGTGGCTCGGAATGGTTACCATGATTGTCGGAGAGATAGCGAATTTCGTGGCGTACGTTTATGCCCCCGCGGTGCTTGTCACGCCGCTTGGTGCTTTGAGTATTATTGTTAGTGCTGTGTTGGCGCATTTCATGTTGAACGAGAAGCTGCAGAAAATGGGCATGCTGGGGTGTCTTCTGTGCATTGTGGGGTCCACTGTGATTGTGCTCCATGCACCTCAAGAGAAGCCTCTTAGTTCTGTAGAAGAAATTTGGCAGTTAGCACTTCAACCGGCATTCTTGTTGTACACTGCCTCGACCATCGCTGTAGCTTTCTTTTTGATATTGTATTGTGCTCCTCGCTTTGGCCAGACTAATATTTTAGTTTATATTGGAATATGCTCCATAATTGGATCCTTGACTGTCATGAGCATAAAAGCCATTGGCATTGCTATAAGACTTACAATTGAGGGTGCCGATCAGTTTGTTCAGTTTCAGACATGGATTTTTACGATGGTTGCTATTTCCTGCATCATTACGCAGTTAAATTATCTTAATATGGCATTGGATACTTTTAACACAGCAGTTGTTTCTCCGATCTACTATGCCTTGTTCACATCTTTTACAATATTAGCTAGTGCAATCATGTTTAAGGACTATTCTGGTCAAAGTATAAGCAGTATTGCATCAGAGTTATGTGGTTTCATTACTGTTTTATCTGGTACAACTGTATTACACAGTACAAGAGAGCCAGATCCTCCAGTCAATACAGATTTGTATAGTCCCTTGTCTCCAAAAGTATCGTGGTATATCCAAGGCAATGGCGAACCTTGGAAACAGAAAGAAGAAGATGGGCCACCCTTTAATTTAATTACAGTTATCCGGCAAGACCATTTCAAGTGA

>GLYMA.06G208700

ATGGCATTTCTCTTTTATGCAGCTTTGGTAATAACAGCTATTTTTATCCTTATCTTCCACTTCATTCCTCTCTATGGCCAGACACACATAATGGTTTATATCGGTGTTTGTTCCCTTGTAGGTTCTATAACGGTTATGAGTGTTAAGGCTCTTGGAATTGTCATAAAGTTAACACTGTCTGGGATGAATCAGCTAATTTACCCTCAAACTTGGGCATTCACTCTAGTTGTAATTGTTTGTGTTCTTACCCAAATGAATTATTTAAATAAGGCACTGGATACTTTTAATACGGCAGTGGTATCTCCCATATATTATGTTATGTTCACAACATTTACCATTGTGGCAAGTGTTATTATGTTTAAGGTAAGTTTTGTTACCTAA

>GLYMA.08G126600

ATGGGCAAGACTCCTTTCTCCTTTCGTCGCTCCGCCTCTCGCCGCCGCCCCAAGAAGACCGCCGCCCCGCCCCCTCCTCCCTCTCCGCCGCAACATCCCTACGCGGCCGGATTCGCCGCCTCACCCGACGACAACAACCGTCTCATCGCCGCCGCCGCCGGCAGCAGCGCCTTGACGAAGGCGAAGAAGAAGACCGGCAACGTCCGCTTGTGGATGAGGTTTGACCGGTCCGGCCGGTCAGAACTGGTGGAGTTGGAGAAGAACGCCATCGTCCGCCACGCAGCGATTCCCGCTAGAGACTTGCGAATTTTGGGCCCCGTCTTCTCCCACTCCTCCAACATCCTCGCTAGAGAGAAAGCAATGGTGGTTAATTTGGAGTTTATAAAGGCAATTGTGACTGCTGAAGAAGTGTTATTGCTTGATCCTCTTCGGCAGGAGGTTCTTCCCTTTGTTGAGCAACTCAGGCAACAGCTTCCTGGCAAAAGTCAACCTAAACTTCTCGGCGGCACGGAAGAGCAGGAAGGCGAAATGCATGTCTCTAATGGAAGACAATGGTTGCCTACACCGGAGGCAGCCGATGGTTTGCAGTCTGAGCTTCCGTTTGAGTTTCAAGTTCTGGAGATTGCTTTGGAGGCTGTGTGCACTTATCTGGACTCGAATGTGGCGGACCTTGAGCGAGGTGCTTACCCTGTGTTGGATGAATTGGCTAGGAATGTTAGCACCAAGAATCTTGAGCATGTGCGGAGTTTAAAGAGTAATCTTACGCGGTTGCTGGCACGAGTGCAGAAGGTGCGAGATGAAATTGAACATCTGTTAGATGACAATGAAGATATGGCACAACTATATTTGACAAGGAAGTGGTTGCAGAATCAACAATTTGAGGAGGCTCATTTGGGTGCCACAACCTCAAATAACTTTCCTAATACCTCACGTTCTGTTCGTCAACTTGGTTCTATCAGAAGTGAAAGTCTTGTGACCAGCCATTATGAGGATGATAACAATGTGGAGGACTTGGAGATGTTGCTTGATGCATATTTCATGCAGTTGGATGGAACTCGTAACAAAATATTATCAGTTAGGGAGTATATTGACGACACTGAAGACTATGTCAACATCCAACTTGATAACCACCGAAATGAACTTATTCAGCTGCAGTTGACGTTGACTATTGCATCATTTGCTATTGCTATTGAAACTTTGATTGCTGGTGCATTTGGTATGAACATTCCTTGTAACTTATATAACATTGATGGAGTATTTTGGCCCTTCGTTTGGACCACGTCTGCGGCTTGCGTATTACTTTTCTTGCTTATTTTAGCATATGCAAGATGGAAGAAGTTGCTGGGATCATAA

>GLYMA.09G019600

ATGAGTGATACAGAAGGTCAATCACATTCCTCGGGAATAACGAAGAAACTAGATAGCCATAAAACTTATCATGGTAGAGATCCCAATAATGGAAATGACCTTTGGAAAGATGGACTTATTTGTGCTTTTGAATACATTCGAGGACAAAACAGATCGGCTAAATTGAGTTCCTCCTCATCAAAGATCACAGACGGAATGCATGGCCAACATTCAAAGATGCATCATGTCCCTTCGGATGATAAAAAGAAGCTCTCAGATCCTTCATCTGTAAATGTCTCGAGGGAGAGTTTGTTTGGTGGCTCTGATGACGACAAGGAGAGCCAGACCCCTAAGGCTGGCCAATCTAAAAAGTATGAGGGTGGTCATTGGGTACCAATTGGATGGGCAAGAATTTCAGAACTTGTCCAAGCAGTTCAGGTTGATGCTGAGTGGTCTTCTCATCAATTTGAATTTGAGTATTCTAAAGATGATTTTACTGTAGCAGATTTGGCAGCTCCCTATTGGGAGCATCCCACTGGGCCTATATGGTGGTGCCATGCTTCTGCAGGTCACCCCACTTGGCTCAGCAATGCTCAATGGCTACATCCTGCTGTTAGTTTAGCTCTGAGAGACGAAAGTAGACTTATAAGTGAGCGGATGAAACATCTTTTCTATGAGACATCCAATGTATCTCATGCTTGCATTGATCTTAGTGCACTCTTTTTTCCAACTGCATTTTATGTAAACTTCAAGTTATTCCGTAAATCTATATTTGGGGCAGCAGATGAGATTGAATTGAAGTTTATGAACAGGAGAAACCATGAAGATTTGAATCTTTTCATCCTAATCTTAAATCTAGAAATTAGAAAGTTATCAACACAGGTTATCAGGGTTAAGTGGTCACTCCATGCAAGAAATGAGATTGTCTTTGAGCTTCTTCAACATCTAAAAGGAAATGGAGCAAGAAACCTGTTAGAGGGAATAAAAAAGAGCACAAGAGAAATGATTGAGGAGCAAGAAGCTGACAGAAGCCTTCGAGTAACCCATAATTTAGCTGTATTTGGTGGCGTTGGCGTTGTCCTCACCATCATTACCGGATTATTTGGGATCAACGTTGATGGGATACCTGGGGCAGAACATACTCCGTATGCATTCGGTGTTTTCACGGCCATCCTTGTCTTTCTGGGAGTAGTGCTGATCGCAGTTGGCATGGTTTACCTTGGGCTGAAAAACCCTGTTGCTGAGGGACAGGTTGAAGTTAGGAAGCTTGAGCTGCAAGAATTGGTGAAGATGTTTCAGCATGAAGCAGAGACTCATGCTCAAGTCCGGAAAAATATTTCTCCCAAAAACTTACCTCCTACTGCTGGTGATGGTTTCCGCAGTGATGCAGATTATCTTGTCATACAATAG

>GLYMA.10G180200

ATGGCTCGTGATGGGAGTGTCGTCCCTGCGGACCCGCAGGCAATGGCGGTTGTGAAGAAGAAGACGCAGTCTTCGAGGAGTTGGATTCTGTTTGACGCCACTGGGCAAGGCACGTTGCTCGACATGGACAAATATGCCATCATGCATAGGGTTCAGATTCATGCGCGTGATCTCAGAATCCTTGATCCCTTGCTCTCTTACCCCTCTACCATTCTTGGTCGTGAGAAGGCCATTGTTCTTAACTTGGAGCATATCAAGGCAATTATCACCGCTGAAGAGGTATTGCTGAGAGATCCAACAGATGAAAATGTGATCCCTGTTGTTGCGGAACTGCAAAGGCGGTTGCCTCGATTAGGTGCTGGTCTTAAACAGGAAGGAGATGGTAAAGAGTATCTTGGTGGCCAAAATGATGCTGAAGCAGCTGAAGAAGACGAGTCACCCTTTGAATTCCGGGCCCTGGAGGTTGCTTTAGAAGCCATTTGTAGTTTTCTTGCTGCACGTACATCAGAATTGGAGATGGCTGCTTATCCTGCATTAGATGAACTTACCTCCAAGATTAGTAGTCGAAATTTGGACAGAGTTCGAAAACTGAAGAGTGCAATGACAAGGCTGACTGCTAGGGTTCAAAAGGTCAGAGATGAGCTTGAACAATTGCTGGATGATGATGATGATATGGCTGACCTATACCTGTCAAGAAAGGCTGGTTCAGCATCACCAGTTAGTGGATCAGGTGCTGCTAATTGGTTTGCTGCCTCTCCTACCATAGGATCAAAGATATCTAGAGCAAGTTTAGCAACAGTTCGTTTAGAAGAAAATGATGTGGAAGAGCTTGAAATGTTACTCGAGGCTTATTTCAGTGAAATCGACCACACATTGAACAAATTAACCACACTGCGAGAGTACATTGATGATACCGAAGATTATATTAATATTCAACTTGACAACCATCGTAATCAGCTGATTCAGTTGGAGCTCTTTCTTAGCTCAGGAACTGTTTGTCTATCTTTCTACTCTTTGGTGGCTGCTATATTTGGCATGAATATCCCATATACTTGGAACGATAACCATGGTTACATGTTCAAATGGGTAGTTATTGTCTCAGGAGTATTTTCTGCGGTGATGTTTCTCATTATTACAGCCTATGCTCGCAAGAAGGGGTTAATAGGATCGTGA

>GLYMA.11G105300

ATGGTTTCGGTAGTTTTGCCCTTGATCATACACTTTGAACCTCACTATGGACAGACTAACATGCTGGTCTACTTGGGAATTTGTTCATTAGTTGGCTCACTTACGGTTGTGAGCATAAAGGCCATTGGAATTGCAATAAAGCTTACACTTGATGGAATAAGTCAAATAGTTTATCCTCAGACTTGGTTTTTTCTTACCGTGGCCATAATCTGTGTCATTACACAGTTGAATTACCTTAATAGGGCTCTGGATACATTCAATGCCACAATTGTTTCGCCTGTATATTATGTAATGTTCACCACTCTTACTATTATTGCCACTGCAATAATGATTGGTCCCGGTCAGGATATCAGCAGCATAGCCTCTGAGATATGTGGATTCATCACTGTTCTTACAGGAACAATCATATTGCACATGACTAGAGAACAGGAAGAATCCAATATGCAAAAGACCTTTACATGGTTTATAGGTGAGGATTTGATGAAGGATGTTGAGAATGAACACCTGATTCTTATACACGATTCGGATTACCTAGAACGTTGA

>GLYMA.11G255400

ATGGGGAAAACACACGACAACGTAGTTGGGCTTATATTGGCCATCTCTTCCACTGTTTTCATCGGTTCTAGCTTTATAATTAAAAAAATGGGTCTTAAAAAAGCTGCCGACCACGGCAACAGAGCAGCCACGGGAGGGCATTCGTATCTGTATGAGCCGTGGTGGTGGGCTGGAATGATTTCAATGATCGCTGGGGAAATAGCCAATTTTGCAGCTTATGCGTTCGCTCCTGCAATCCTTGTAACTCCTTTGGGAGCTTTGAGCATCATTTTCAGTTCAGTGTTGGCTCACTTCATATTAAAAGAGAAATTGCACATTTTTGGCGTGCTTGGGTGTGCTCTGTGTGTGGTGGGATCTACGTCTATTGTTTTGCATGCACCGAAAGAGAAAGACATTCATTCTGTCAAGGAAGTGTGGGAACTTGCTACAGGACCAGGTTTTATTGTCTACATTTGCGCTATAGTGATATTAGTTTGCGTCCTTCATTTCCGTTTTGTGCGAAGCCATGGGCAGACTCATATGATGGTGTATCTCGGAATATGTTCTCCCACCGGCTCCATTACGGTTATGGGTGTCAAAGCAGTGGGAATCGCTTTGAAGCTTACATTTGAAGGGACGAATCAATTTGTTTACTTTGAGACCTGGATATTTACAGTGGTTGTGATAGGATGTTGCCTTTTGCAGATTAACTACTTGAACAAGGCTTTGGACGCCTTTAGCACTGCTGTGGTGTCACCAGTTTACTACGTGATGTTCACATCATTTACAATCGTGGCCAGCATTATCACGTTTAAGGAATGGGCGAAGCAGGATTCAACGCAGATTGCTACTGAGTTGTGTGGTTTTGTGACAATATTATCTGGGACCTTCCTCCTTCACAGAACTAAGGATATGGGAAATAAACCCTCCGACGCCTCCGTTCATTCAAGTCCTGAAGATAACAATAGTAATACTAAGACACCTCTAAGCAACCAAATTTGA

>GLYMA.12G030100

ATGAGAGGAGAGAGAAAGAAGGTGAAAAATGAAAATATCCGTGTAGCGTGTGAAGGGATGAGCCACCGACCAACACCTGTGCAGCAATTCTCTGTCACCAAATTTGATCTTTCTTTCTTTCTTTCTTTGGCGGGAATGACAGAAACGGGGGTTTCTGATAATTTCAAGGGCTTAATACTGGCGATGGGTTCTAGTGCATTCATCGGTTCCAGTTTCATCTTGAAGAAGAAGGGTCTTAAGCGTGCTGCTGCACGCGGTACTCGTGCAGGAGTTGGTGGCTATACTTATCTACTAGAGCCGCTTTGGTGGGCTGGCATGGTGACAATGATTATTGGGGAGATTGCAAACTTTGTTGCTTATATCTATGCTCCGGCAGTTCTGGTTACTCCCCTTGGTGCACTTAGTATTATTGTCAGTGCTGTTTTGTCTCACTTCTTGTTGAAGGAACGGCTTCCGAAGATGGGGGTATTGGGGTGTGTATCCTGCATTGTGGGATCAATTGTTATTGTCATCCATGCACCACAAGAGCAAACTCCGAGTTCTGTCCAAGAAATATGGGATCTGGCCACTCAACCAGTTTCGGTAGTTTTGGCCTTGATCGTACACTTTGAACCTCGCTATGGACAGACTAATATGCTGGTCTACTTGGGAATTTGTTCATTAGTTGGCTCACTTACGGTTGTGAGCATAAAGGCCATTGGAATTGCAATAAAGCTTACACTTGATGGAATAAGTCAAATAGCTTATCCTCAGACTTGGTTTTTTCTTACCGTGGCCACAATCTGTGTCATTACGCAGTTGAATTACCTTAATAGGGCTCTGGATACATTCAATGCCACAATTGTTTCTCCTGTATATTATGTAATGTTCACAACTCTTACTATTATTGCCAGTGCAATAATGTTTAAGGATTGGTCCGGTCAGGATGTCAGCAGCATAGCCTCTGAGATATGTGGATTCATCACTGTTCTTACAGGAACAATCATATTGCACATGACTAGAGAACAGGAAGAATCTAATATGCAAAAGACCTCAACATGGTTTATAGGTGAGGATTTGATGAAGGGTGTTGAGAATGAACACCTGATCCGTATACATGATTCAGATTACCTCGAACGTTGA

>GLYMA.12G168000

ATGGCGACTTCTTCTTCTTCTTCTTCGACTTCGAGCTGGCGCGAGGGCATGTCCTCCGACAACATAAAGGGACTGTGTCTCGCTCTCTCCTCTAGCTTCTTCATCGGTGCCAGCTTCATTGTCAAAAAGAAGGGTTTGAAGAAGGCCGGTGCTAGTGGAATCAGGGCCGGAAGTGGAGGTTATTCTTACTTGTATGAGCCGCTTTGGTGGGTGGGAATGATAACAATGATTGTTGGGGAGATTGCCAATTTTGCAGCTTATGCATTTGCCCCAGCTATATTGGTCACCCCTCTTGGTGCTCTTAGCATTATTATCAGTGCTGCTCTTGCTCATATTATTTTACGGGAGAGGCTACATATTTTTGGAATTCTTGGTTGCGTTTTGTGTGTCGTGGGATCTACAACAATTGTTTTGCATGCTCCTCAAGAACGGGAAATTGAATCTGTTTCAGAAGTGTGGGATCTTGCTATGGAACCAGCATTTCTCTTTTATGCAGCTTTGGTAATAACAGCTACTTTTATCCTTATCTTCCACTTCATTCCTCTCTATGGCCAGACACACATAATGGTTTATATCGGTGTTTGTTCCCTTGTAGGTTCTCTAACGGTTATGAGTGTTAAGGCTCTTGGAATTGTCATAAAGTTAACACTGTCGGGGATGAATCAGCTAATTTACCCTCAAACTTGGGCATTCACTCTAGTTGTAATTGTTTGTGTTCTTACCCAAATGAATTATTTAAATAAGGCACTGGATACTTTTAATACGGCAGTGGTATCTCCCATATATTATGTTATGTTCACTACATTTACCATTGTGGCTAGTGTTATTATGTTTAAGGACTGGGATAGACAAAGTCCAACACAAGTTATCACAGAAATATGTGGGTTTGTGACCATTCTATCAGGAACTTTTCTTCTTCACAAAACTAAGGATATGGCTGATGGTTTACAACCATCTTTATCTGTTAGACTTCCTAAGCATTCAGAAGAGGATGGCTTTGATGGTGGTGAAGGCATTCCTCTTAGACGGCAAGAAGCCATGAGATCGCCATGA

>GLYMA.13G368400

ATGAGGCGAAAGGGTGTGGGAACGACGGGAGTGAAGAGTTGGATGGTGGTGTCGGAGACAGGGCATGCGCGGCTTGAAGATGTTGGAAAACACTCCATAATGCGGCGAACTGGGTTGCCGGCGCGTGACCTTAGGGTCCTCGATCCCGTTCTCTCGTACCCTTCTTCCATCCTAGGACGCGAAAGAGCTATTGTTGTCAACTTGGAACACGTCAAAGCCATCATCACTGCCTCCGAGGTCCTCCTCATCAACTCCTCCAACCCTTTCTTTCTTTCCTTCCTTCAAGATCTTCACATCCGTCTTTCCAATCTAAATCCCTCCTCGATGAGCAATGACATGGATGGTGGTTACGAAGAAAAACCTCTAGCGAATGATTCCCGAAACGGTTCACCTGTGAGAATACCTGAGGACTCTGATGCCGACTTTCTTGTAAGAGCAGATAGCCTTAAGAGCAGTGCAGAGACTGGAACAGGAACAGGAACAGGAACACCAGCTCCTAAGCCGTTACCTTTTGAGTTTAAAGTACTTGAGGCATGTATTGAATCTGCTTGCAGGTGCCTTGAATCTGAGACTTCAACACTGGAGGTAGAGGCTTACCCAGCTTTAGATGAATTGACCTCTCAACTTAGCACACTCAATCTTGAACGTGTTAGACAAATCAAGAGTCGTTTGGTTGCACTCTCCGGTCGTGTGCAGAAGGTAGCAGATGAACTGGAACATTTGTTGGACGATGATAATGACATGGCTGAAATGTACTTGACGGACAAGCTTAATGCTCGTTTATGTGATCAAACATCATTAAAAGAAGGCTACAATTCCGAATTTGAAGATAATGATCAAAGTGATGAATCAAATTCAGAAAAGTATGACAGATTCCTTTGTCCTAAACTTGACGTTGAGGAATTGGAGATGCTTCTAGAAGCATATTTTGAGCAGACAAATGGAATCTTACAAAGATTGACTAGTTTGAGTGAGTACGTGGATGACACGGAGGACTACATCAACATAATGTTGGATGATAAGCAAAACGAGCTTCTGCAGGCAGCAATAATATTCGACACCATAAACATGATACTTAATGCCGGTATTGTGGTGGTAGGATTGTTTGGCATGAATATTCAGATTGACCTCTTCAATGGTCAACCTCGTCAATTTTGGGCTACAACAGGGGGTACATTTGGAGGATGTCTACTTCTATTCCTTGTATGTTTATGGTGGGGCAAGAAAAGATATTTTCTCTCTCACTAG

>GLYMA.14G033700

ATGGGTCTGTCCAAGGAGAATCTGAAAGGTCTCATACTAGCTTTGGTGTCAAGTGGGTTCATTGGGGCAAGTTTTATCATTAAAAAGCAAGGCCTTAGAAGAGCTGCAGCAGTTTATGGTGTCAGGGCTGGTGTTGGTGGGTATTATTATCTCTTGGAGCCATTATGGTGGGTGGGAATGATCACAATGATTGCAGGAGAGGTTGCAAACTTTGTTGCATATGCGTTTGCTCCTGCAGTCCTAGTTACCCCTCTTGGTGCACTAAGTATTATTGTGAGTGCTGTTTTGGCTGACATTATTCTGAAAGAGAAGCTACACAATCTTGGGATTTTAGGCTGTATAATGTGCATTGCTGGTAGTATCATTATTTTTATTCATGCTCCTAAGGAACAACCTATTACATCTGTTCTGGAAATATGGAATATGGCTACTCAACCAGCTTTTCTGGCATATGTGGGCTCAGTAATAGTATTGGTTTTCATTCTGGTCTTCCATTTTGCACCAAGATGCGGGCATACGAATGTGCTAGTTTTTACTGGCATTTGTTCATTGATGGGTTCCCTCTCTGTGATGAGTGTTAAAGCCCTTGGAACTTCTTTGAAATTAACTTTTGAAGGGAAAAATCAGTTAATCTACCCAGAGACATGGTTTTTTATGTTAGTTGTGGCTATATGTGTCATCATGCAAATGAATTATCTTAATAAGGCTCTTGACACCTTCAACACAGCAATTGTATCTCCTATATACTATGTCATGTTCACAACACTTACAATACTAGCCAGTGTAATAATGTTTAAGGATTGGGATGGCCAAAGTGGTGGAACTATTGTGTCAGAAATATGTGGCTTCATCGTTGTACTCTCTGGAACAATAATGTTGCATGCGACTAAGGACTTCGAGAGAAGCTCTTCTTTTAGAGGCAGTGCTCCTTCATCGCCTACGCTATCTGCCCGACTTTTTACCGGAAATGGGGACTCATTACTTAAGCAAGATGAGGAAAATGGATCTCCCGAGAGTAATATGTGCTCAAGAAGGCAAGAGTTGTATTAG

>GLYMA.14G097400

ATGTGGGAATCTGTTGTGTTGACGGTAGCTGCCACCGCCGGCAACAACATCGGAAAGATCCTTCAGAAGAAGGGCACTATCATTCTTCCACCTCTCTCTTTCAAACTCAAGATCTCATTGTGTTGGCAGGTCATAAGGTCTTATGCTTTAAACAAAACCTGGGTGGTAGGTTTTCTAATGGATATATTAGGGGCATTATTGATGTTAAGGGCATTGTCTCTGGCTCCAGTGTCTGTCATCCAACCAGTTTCTGGCTGTGGACTAGCAATTCTTTCAATCTTTTCTCATTTTTATCTCAAGGAAGTCATGAATGCTGTTGATTGGGTTGGCATTACATTAGCAGGTTTTGGCACAATAGGAGTTGGTGCTGGAGGTGAGGAGCAAGAGGTGGTTGCTCTATCTATTTTTCACATTCCAGGGCTGGCATTTGTTGTTTTCATCTTGTTTATACTTCTTAGTGGATGGCTTCGAATATGCAAGTGCCAACGAAGAGAACAAGAGATGGTGGAATATGATGTTGTTGAGGAAGTCATTTATGGCTTGGAATCTGGAATTTTGTTTGGTATGTCATCTGTAATATCGAAGATGGGATTTCTATTCCTAGAGCAAGGTTTTCCCAAGCTGTTGGTTCCTATGTGCATCATGATTAGTGTGTGTTGTAGTGGCACTGGCTTTTACTACCAGACACGCGGTCTAAAGCATGGAAGGGCTATTGTAGTTTCCACATGTGCCGCTGTGGCATCAATTTTGACTGGTGTTCTTGCTGGGATGCTTGCTTTGGGTGAACGACTTCCTTCGGAACCAAAAGCTCGCTTGGCACTTCTTCTTGGATGGCTACTTATTATAGTTGGTGTGATTTTACTTGTTGGTTCAACACGGCTAGTGAGATTCCTTTCTTGTTCTTCACAGCGAAAAAGAAGCAATGTGGATAAGAATTTTGACCTTAGAAGAGCCACTTCTTCCCGTGTGAGGGAAACAAGTCCAAGTGCTGTCATTCAAGCAGCAACATTAAATCATTTACTATCATCATCTTCCAAAGAAAAAGCTTGA

>GLYMA.15G125900

ATGAGTGATACAGAAGGTAAATCACATTCCTCGGGAATCACGAGGAAACTAGATAGCCATAAAACTTATCACGGTAGAGATCCCAATCACGGAAATAACCTTTGGAAAGATGGACTTATTTGTGCTTTTGAATACATTAAAGGACAAAACAGATCGGTTAAATCGAGCTCCTCATCAAAGATCACAGACAGACTACATGTTAATGGCCAACATTCAAAGATGCATGTCCCTTCAGATGACAAAAAGAAGCTCTCAGATCCTTCATCTGTAAATGTCTCAAGGGACAGTTTGTTTGGTGGCTCAGATGACGACAAGGAGGGCCAGGCCCATAAGGCTGGGCAATCTAAAAAGTATGAGGGAGGTCATTGGGTACCAATTGGATGGGCAAGAATTTCAGAACTTGTCCAAGCAGTTCAGGTTGATGCTGACTGGTCTTCTCATCAATTGGAATTTGAGGATTCTGAAGATGATTTTACAGTAGCAGATTTGGCAGCTCCTTATTGGGAGCATCCGGCTGGGCCTATATGGTGGTGCCATGTTTTTGCAGGTCACCCCACTGTTGAGGCTTGGCTCAGCAATGCTCAATGGCTACACCCTGCTGTTAGTTTAGCCTTGAGAGACGAAAGTAGGCTTATAAGTGAGCGAATGAAACACCTTCTCTATGAGGTCCCAGTCAGAGTTGCAGGAGGGCTGTTATTTGAGCTCTTGGGACAATCCGCAGGTGATCCTCTTGTTGAAGAAGATGACATTCCAATTGTTCTTAGGTCTTGGCAATCTCAAAACTTCCTTGTAACTGTAATGCATATAAAAGGATCAGTATCAAGGATAAATGTTCTGGGTATAACAGAAGTTCAGGAGCTTCTTTCTGCTGGAGGGTATAATATGCCGAGAACAGTGCATGAAGTTATAGCACTACTTGCTTGTCGTCTCTCACGGTGGGATGATAGGTTATTCCGTAAATCTATATTTGGGGCGGCAGATGAGATTGAATTGAAGTTTATGAACAGGAGAAACCATGAAGATTTGAATCTTTTCATCTTAATCTTAAATCAAGAAATCAGAAAGTTATCAACACAGGTTATCAGAGTGAAGTGGTCACTCCATGCAAGAGATGAGATTGTCTTTGAGCTTCTCCAGCATCTAAAAGGAAATGGAGCAAGAACCTTGTTAGAGGGAATAAAAAAGAGCACAAGAGAAATGATTGAGGAGCAAGAAGCTGTTCGTGGCCGCCTGTTTACCATTCAAGATGTTATGCAAAGCACTGTTCGAGCTTGGTTGCAGGATAGAAGCCTTCGAGTAACCCATAATTTAGCTGTATTTGGTGGTGTTGGCGTTGTCCTCACCATCATTACTGGGTTATTTGGGATCAACGTTGATGGGATACCTGGGGCAGAACAGACACCTTATGCATTTGGTGTTTTCACGGCCATCCTCGTCGTTCTGGGAGTAGTGCTGATAGCAGTTGGCATGGTTTACCTTGGGCTGAAAAACCCCGTTGTTGAGGAACAGGTTGAAGTAAGGAAACTTGAGCTGCAAGAATTGGTGAAGATGTTTCAGCATGAAGCAGAAACTCATGCTCAAATGCGGAAAAATATTTCTCCCAAGAACTTACCTCCTACTGCTGGTGATGCTTTCCGTAGTGATGCGGATTATCTTGTAATACAGTAG

>GLYMA.16G003900

ATGGCGACTTCTTCGAGTTCTTCGAGCTGGCGCGAGGGCATGTCCTCCGACAACATAAAGGGACTGTGCCTCGCTCTCTCTTCTAGCTTCTTCATCGGCGCCAGCTTCATTGTCAAAAAGAAGGGTTTGAAGAAGGCCGGTGCTAGTGGAATCAGGGCCGGAAGTGGAGGTTATTCTTACTTGTATGAGCCGCTTTGGTGGGTGGGAATGATAACAATGATTGTTGGAGAGATTGCCAATTTTGCAGCTTATGCATTTGCCCCAGCTATATTGGTCACCCCTCTTGGTGCTCTTAGCATTATTATCAGTGCTGCTCTTGCTCATATTATTTTACGGGAGAGGCTACATATTTTTGGAATTCTCGGTTGTGTTTTGTGTGTTGTGGGATCTACGACAATTGTTTTGCATGCACCTCAAGAACGGGAAATTGAATCTGTTTCAGAAGTGTGGGATCTTGCTATGGAACCAGCATTTCTCTTTTATGCAGCTATGGTTATAACAGCTACTTTTATCCTTATCTTCCACTTCATTCCTCTCTATGGCCAGACACACATAATGGTTTATATTGGTGTTTGTTCCCTTGTAGGTTCTCTAACGGTTATGAGTGTTAAGGCTCTTGGAATTGTCATAAAGTTAACACTGTCTGGGATGAATCAGCTAATTTACCCTCAGACTTGGGCATTCACTCTAGTTGTACTTGTTTGTGTTCTTACCCAAATGAATTATTTAAATAAGGCACTGGATACTTTTAATACGGCAGTGGTATCTCCTATATATTATGTTATGTTCACAACATTTACCATTGTGGCTAGTGTTATTATGTTTAAGGACTGGGATAGACAAAGTCCAACACAAGTTATCACAGAAATATGTGGGTTTGTGACCATTCTATCAGGAACTTTTCTTCTTCACAAAACTAAGGATATGGCTGATGGTTTACAAACATCTTTATCTATTAGACTTCCTAAGCATTCAGAAGAGGATGGCTTTGATGGTGGTGAAGGCATTCCTCTTAGACGGCAAGAATCCATGAGATTGCCATGA

>GLYMA.16G149500

ATGGGGGAGTGGATTGTTGGAGCTTTCATCAACCTCTTTGGTAGTATTGCAATAAACTTTGGGACCAATCTTCTCAAATTAGGGCATAATGAGAGAGAAAGACATTTACTTGGAAGTGATGGGGTAAATGGAAAGATGAATCTGAAGCCTATTATATACTTCCAAAGTTGGAGAATTGGCATTGTATTTTTCTTTCTTGGAAATTGCCTTAATTTCATTTCCTTTGGGTATGCTGCTCAGTCACTTCTTGCAGCACTAGGATCTGTTCAGTTTGTATCTAACATTGCCTTCGCTTACTTTGTCTTGAACAAAATGGTGACAGTAAAGGTACTGGTTGCAACAGCTTTCATTGTTCTTGGGAATGTTTTTCTAGTCGCTTTTGGCAATCACCAATCACCTGTTTATACGCCAGAGCAGTTGACAGAGAAATATACCAATATTGCATTCCTTCTATACCTTCTAGCTTTGATCTCTATTGTTGCCTTGCATCACTCCATCTACAAGCGGGGAGAACTTCTGTTTGCAGTATCAGGACATGACCTCAGACCCTATTGGAGCATGCTACTGCCCTTTTCATATGCTGTAGTTTCGGGGGCTGTAGGTTCATGCTCAGTCTTGTTTGCTAAATCGCTTTCTAACCTATTACGACTGGCTATGTCCAATGGTTATCAGTTGCACAGCTGGTTCACATATTCCATGCTTCTTTTATTTCTTAGTACTGCTGGATTTTGGATGACCAGGTTGAATGAAGGACTGTCATTGTTTGATGCAATTCTTATTGTTCCCATGTTTCAGATAACATGGACTTTCTTCTCAATCTGTACAGGATTTATCTATTTTCAAGAATATCAGGTATTTGATGCATTAAGGACAACGATGTTTATACTTGGAATGATGTGTGTGTTTATTGGCATTTCTTTGCTGGCACCTGATGAATCAAAAGTTTCAGGTCCTGAGACTAAAGATAGTTCTTTGGATTCCATGGTGTCTTCTGCCATGTCAACAGAAACTAGCAGGCTGGTAGTGTCTCCCGAAGAAGCACAAAACAAAGACTCAAGATCATTTGTCAAAGCAATACTAATAAAGGTTACAGATTTGTTGGTAAAGGCAAAGACTTCTTGTGCATTGTCTCTTGGTTTTGGGGAGGATACCATCAACACATCATCGGTTCTTGTGATGCCAATGATGTCATCAAGAATGACTGGATTCAGAGGAAATGGGCTTGAAAGAGCAAGAATATTGTCCATGAGAAATGGTTGGAGCAAGATTCCAATGGATGAAGATGCTGGCAAATTGCTTGAAACTAGTTCAGTTGTTCCTCCTAGCCCTTAG

>GLYMA.17G227100

ATGTGGGAATCTATTCTGTTAACGGTGGCTGCCACCGCTGGCAACAACATCGGAAAGATCCTTCAGAAGAAGGGCACTATCATTCTTCCACCTCTCTCTTTCAAACTCAAGGTCATAAGGTCTTATGCTTTAAACAAAACCTGGGTGGTAGGTTTTCTAATAGATATATTTGGGGCATTATTGATGTTAAGGGCATTGTCTCTGGCTCCAGTCTCTGTCATCCAACCAGTTTCTGGCTGTGGACTAGCAATTCTTTCAATCTTTTCTCATTTTTATCTCAAGGAAGTCATGAATGCTGTTGATTGGGTTGGCATTACATTAGCAGGTTTTGGCACAATAGGAGTTGGTGCTGGAGGTGAGGAGCAAGAGGTGGTTGCTCTATCTATTTTTCACATACCAGGGCTGGCATTTATTGTTTTCATCTTGTTTATACTTCTTAGTGGATGGCTTCGAATATGCAAGCGCCAACGAAGAGAACAAGAGATGATGGAATATGACGTCGTTGAGGAAGTCATCTATGGCTTTGAATCTGGTATTTTGTTTGGGATGTCATCTGTAATATCAAAGATGGGATTTCTATTCCTAGAGCAAGGCTTTCCCAAGCTGTTGGTTCCTATGTGCATCATGATCAGCGTGTGTTGTAGTGGCACAGGCATTTACTACCAGACACGTGGTCTAAAGCATGGGAGGGCTATTGTAGTTTCCACATGTGCAGCTGTGGCCTCAATTTTGACTGGTGTTCTTGCTGGGATGCTTGCTTTGGGTGAACGACTTCCTTCGGAGCCAAAAGCTCGCTTGGCACTTCTTCTTGGATGGCTACTTATTATTGTTGGTGTGATTTTACTTGTTGGTTCTACACGGTTAGTAAGATTCCTTTCTTGTTCTTCACGACAAAAAAGAAGCAATGTGGAGAAGAATTTTGGCCTTAGAGGAGCCACTTCTTCCCGTGTGAGAGAACCAAGTCCAAGTGCTGTCATTCAAGCAGCAACATTAAATCATTTACTATCATCATCTTCCAAAGAAAAAGCTTGA

>GLYMA.18G091200

ATGATTATGCTTCTTTCCATTTTTGCCACGACACAAGAACATAATTTAACGACTAACCAAGAACATAATTTTTCTACTGCAGCATTTCTGTTTTATGCAGCTTTGGTAATAACAGTTACTTTTATCCTTATCTTCCACTTCATTCCTCTCTATGGCCAGACACACATAATGGTTTATATCGGTGTTTATTCCCTTATAGGTTCTATAACGGTTATGAGTGTTAAGGCTCTTGGAATTGTCATAAAGTTAACAATGTCTGGGATGAATCAGCTAATTTACCCTCAAACTTGGGCATTCTCTCTAGTTGTAATTGTTTGTGTTCTTACCCAAATGAATTATTTAAATAAGGCAGTGGATACTTTTAATGCGGCAGTGGTATCTCCCATATATTATGTTATGTTCACAGCATTTACCATTGTGGCTAGTGTTATTATGTTTAAGGGATTTATTGCATATGCCCTTAATTATGGACTCATTACATGGTGA

>GLYMA.20G210300

ATGGCTCGGGGGGATGGGAGCGTCGTCCCTACGGACCCGCAGACAATGGCGGTTGTGAAGAAGAAGACGCAGTCTTCGAGGAGTTGGATTCTGTTTGACGCCACAGGGCAAGGCTCCTTGCTCGACGTCGACAAATATGCCATCATGCATAGGGTTCATATTCATGCGCGTGATCTCAGAATCCTTGATCCCTTGCTCTCTTACCCCTCCACCATTCTCGGTCGTGAGAAGGCCATTGTTCTTAACTTGGAGCATATTAAGGCAATTATCACCGCTGAAGAGGTATTGCTGAGAGATCCAACAGATGAAAATGTGATCCCTGTTGTTGAGGAACTGCAAAGGCGGTTGCCTCAATTGAGTGCCACCGGTCTTCAACAGCAAGGAGATGGTAAAGAGTATCTTGGTGGCCAAAATGATGCTGAAGCCGCTGAAGAAGATGAGTCACCCTTTGAATTCCGGGCCCTGGAGGTTGCTTTAGAAGCCATTTGTAGTTTTCTTGCTGCACGTACAACAGAATTGGAGATGGCTGCTTATCCTGCATTAGATGAACTTACTTCCAAGATTAGTAGTCGTAATTTGGACAGAGTTCGTAAACTGAAGAGTGCAATGACAAGGCTGACTGCTAGGGTTCAAAAGGTCAGAGATGAGCTTGAACAATTGCTGGATGATGATGATGATATGGCTGACCTGTACCTGTCAAGAAAGGCTGGTTCAGCATCACCAGTTAGTGGATCAGGTGCTGCAAATTGGTTTGCTGCCTCCCCCACCATAGGATCAAAGATATCTAGAGCAAGTAGAGCAAGTTTAGCAACAGTTCGTTTAGATGAAAATGATGTGGAAGAGCTTGAAATGTTACTTGAGGCTTATTTCAGTGAAATCGACCACACATTGAACAAATTAACCACACTGCGAGAGTACATTGATGATACTGAAGATTATATTAATATTCAACTTGACAACCATCGTAATCAGCTGATTCAGTTAGAGCTCTTTCTTAGCTCTGGAACTGTTTGTCTATCTTTCTACTCTTTGGTGGCGGCTATATTTGGCATGAATATCCCATATACTTGGAACGAAAACCATGGTTACATGTTCAAATGGGTAGTTATTGTCTCGGGAGTATTTTCTGCTGTGATGTTTCTCATGATTACAGCCTATGCTCGCAAAAAGGGGTTAGTAGGATCGTGA
