# Supplementary material for: Genome-wide identification and functional characterization of magnesium transporter (MGT) gene family in soybean (Glycine max L.) and their expression profiles in response to aphid infestation, dehydration, and salt stresses
Source: PLoS One. 2025 Aug 29;20(8):e0330440. doi: 10.1371/journal.pone.0330440 (PMC12396710; doi:10.1371/journal.pone.0330440)
Supplement: S3 Data — (S3 Data.DOCX) [file pone.0330440.s003.docx]

>GLYMA.02G068000

AAAACCCTTTTTCATAATATTTCCCTTCCCTCTGAATCCACCTTTCTTTCTTTATACAACCCACGCACGCGTCCAACCTCGCAATTTCAATTTTTTTTTTCTGTAGCAATAGAAGAGGGGAATTGATTGATTGTCTCTCAGGTAATCGAGTACCTTTTTCAAGCTTGCACGGTTGTTCCCATTTCACCACAATGCATTTTGGGTTTTAATTTCCTTTTTCTAAATTTTTTTATTTTTGCAATGGTTTAGTTGGATCGGTCTTGCGGGTTCAATTTACTTTCAAACATAGCAGATCTGGGTTCTTCAAGGTTAGAAACCTGCCTCTTTTTTTGTTTTGTTTTGTCTTAATTAAATGTTGAAAGATCAGAAGATTGAAAGAGTTATATTGTGTTGTTTATTCCATCAATTAATTAGACTTTTTGTGTCCCCTTGTTAATTTTTTTTTATAAAAAAAAATGATTTTTGGTGCACAAATTGATTAGTCTTACTTTTTTCTGGTTCGAAATTGAGCCAATTGGGTTTGTGTTTTCATATTGAATGTTTTCTATTTCTTGTTATAGCCCCCTTTATCAGCTTCATTTATTTTACTCATAGATCTCCTAGGTTTTAGTAGGTAATAACATTGTTGAAGTTGTGAACTTTGATTTGATGGTTTGATTTGTGTTTGGTGGTGACACAGGAGTATTGTTGTTCTGACACTTGGTGTCCAGAAGAAGGGCTATTTTCCTTTCTATCCGTGTTTTGCATTGTGTACTAGAGTTTGAGAACTAACTTCTGATAGACAGAGCCATCGGATTTTTTTTCTGCTGCATGTGGTGTCTCCTTGGTGGTGCCACATCATTTCATTAATGGGGGAGTGGATTGTTGGAGCTTTCATCAACCTCTTTGGTAGTATTGCAATAAACTTTGGGACCAATCTTCTCAAACTAGGGCATAATGAGGTATGGCCAAATTCTTTTTTCTGGTTGGAACTATTTGTAAAGTACATTTTTGTATATATGTTTCTCTCTTTTTTGTGTTGTGGTACAGAAATTGGCTTTGCTGTTATAATTTTTTTCAGATTTCAGCTGACATGGTAAATCTTGACTTCATCAATGGGTCAATGTACTGTTTCTGTACACAACTGTATTTTTTTACATAATTGCATTAGGCATTGTCTACATGATGCTTTGGATTAGGGACTTATGCTATTAAAGTGAAGTGCTGAAACTTCCTTACACTTAATTACATCTGACTCTGAGTTTCAGATTTATTTAGGTTCATGGCATATTACATCTTTGAGGGTCAATATTAACCTGCAATGTGCTACTATTTCTTGAAAGTTGCAACTTTTAGTATCAAATTATTTGGGAAAACTGAGTTAGTTTTTTTTTGGCAGAGAGAAAGACATTTACTTGGAAGTGATGGGGTAAATGGAAAGATGAATCTGAAGCCTATTATATATTTCCAGAGTTGGAGAATTGGTATAATCTAATTGCATTTATATATGGGTTTGTGCATATTATCTAGGTGGATGATTCCTGATTTTTTGCTTTCATTGCATAATTCTTGATATGCAGGCATTGTATTTTTCTTTCTTGGAAATTGCCTTAATTTCATTTCCTTTGGGTACGCTGCTCAGGTATGTTTCCCCTATTTAATTGGGATTTTTTGTTCAATAATATATGGTGTAAACTGTAAATTATGATAGTATGTAGTAGTTTTGCATTTTTTTATTTGGTTGGCATATATTCGGTGAAATATGCGATTTCTTATGATTGATTTAATGGATGTATTTTCATTTGTTCTGATTACTTATTCTTCTGTGTGCTGTGCAGTCACTTCTTGCAGCGCTGGGATCTGTTCAGTTTGTATCTAACATTGCCTTCGCTTACTTTGTCTTGAACAAAATGGTGACAGTCAAGTATGCTAAATTTGAACTGAGATTACCGTTTCATTTTTGTTTGTTTTCACAACTCTGTTCTAGCTGTAATTCTTGTAGCTTAATTGATGGCATTTGATCAGGGTACTGGTTGCCACAGCTTTCATTGTTCTTGGGAATGTTTTTCTAGTTGCTTTTGGCAATCACCAATCGCCTGGTATGTAACATTTTGCCCAGTTCAATTAAATTTGCTTCTAGTCTTTCTTGCAAAAACAATGTTAATTAATCTGATAGATGTGCATACAGTATCAAGTATTGTCTGCAATCAAATGACTAAACTGCTAATTTGGTGAACTACAACTTAAGTATGCTAGGCTTTTATATGTTAGAGATAGGAGGGAGCATTTTGTACTGCCTCTTCTGTAAACTTAATTTGATTTGTTGTTTAATATATTCTTTATGCTTACTGAATATTATGATTCATTGCTGTTAAACAGTTCAATCATTGATTTCAATGTTGCCAATGCAAACTTATATGTGGTTAACTACTTGTGTTTCAGTTTATACGCCAGAGCAGTTGACAGAGAAATATACCAATATTTCATTCCTTCTATACCTTCTAGCTTTGATCTCAATTGTTGCCTTGCATCACTCCATCTACAAGTAATTAGTAGGATTTAATTATTTGTTGAGTCTAATCAAGCTATTTTTCTTCTTTATGATTAACATGTGGAATATGCAGGAGGGGAGAACTTCTGCTTGGAGTATCAGGACATGACCTCAGACCCTATTGGAGCATGCTACTGCCCTTCTCATATGCTGTAGTTTCAGGGGCTGTAGGTTCATGCTCAGTGTTGTTTGCTAAATCACTGTAAGTCAGCTACTCCAACATGTTGCCATGCTTCATGATACAATAGCCATTGTGCCTAATGTCAACTTTTTGGCAGTTCTAACCTATTACGACTGGCTTTGTCCAATGGTTATCAGTTGCACAGCTGGTTCACGTATTCCATGCTTCTTTTATTTCTTAGTACTGCTGGATTTTGGGTAAGGCTTTTTCTTGGGTAAAGATTCTTTGTTGGGCTTTATCAATTTGTGCTAATGTTTTGATGAGGTAACTTTTTGAAGTTAATTTTTGACAAATGTGAATGCTAACGAAGCCTTTTCCAATTGTGCAGATGACCAGGTTGAATGAAGGACTGTCATTGTTTGATGCAATTCTTATTGTTCCCATGTTTCAGATAGCATGGACTTTGTTCTCAATATGTACAGGATTTATATATTTTCAAGAATATCAGGTACTTCCTCTTTAACTTGATACACACAGGTAGCCTTTGCCTTTCATGGGCACTCTAAGAAGATTTAATCATATTATTGAAGAAAACTAATTAAATATCAACTACTTTGATGGAATGGAGTCACTTGAGTATATATTTCAGAAAATCAAATCCGTTACATCATTTAGAAACACCATGGGGCATGGGTTCAACATACAAAACCAGACCTAGCCTAGATAATGTAAGTCAAACTCAGATAAATGACTTAATCTTGCTTCTATGTACATTTGCCAAGTTACCAAGATGTGGTATATTATAGTGTAACTGCTTGTTAGGTTCATTCTCTGGTGACATCTATCAACTGTTTTTTCTGCAAGGTCTTGACCTACTGAAATTTCCCATTCTGGTTATAAAATTTTCTCAAACAAGAAATTAATCTTCTGTTTTTACCAGTGGAGCTTTGAGCCATGGGTCATTGCTTACTGCTGGGGAACAATATGAACTTCTAAGTTCTGATATTACTGCAGAGAATGCAAGCAAATCTAAACAAATTAAATAAAATAGAAACTAATAAGGAAGATATTGCATGGTAGTTAAAATCGAGATTTTACTTAAGGATTGGGAAGGGAGTTAAGACCACAAATCACAGGAACAATGGAGGAGAAGAAGATAGCATGCAGCAGTGCTTTCAGCTTTAAAATGGCTGAAAATGGGGGTAAATTTTGCAAATTTAGGATTCAGAACTACCTGGATATTTCATGTTGCAAAACAGGCTGCTGGTCAAAAACCTAACAATGGACTTCAGGTGTGCTGAGGTTACAAGATCCCAGCAATCCTATGAACTTGCCCTGTGATCTCAGTCAATTTAGTAGTGTAACTCTCTTGTCCAAGATGAAGGAATTTATAGACCATATAAAGAGCAAAGCCATTGTAGATTTAGAAATTCCTTGTAAGACCATTTGTCTCCATCCAATAGATTTAAAATGTTCAATATGAACAAAATACAAAATGTTCAAACAGAAAATATAAATATTGCAGAGCATCACAAAATTCATAGAAACTGAACTACATAGAATGACAAAAGAAATGTTTATTTCAAAATTCAAAATGTGCTAAGTGTCTAACTGAAGGAGATGCATGTTAGATAGAAGAGAATAGCTGGGAAGGAATGACTCTACTCTCAAGAAAAGTCTTATCTTTCATATATAGAGATTGATTGGTCCCCTTGACATCATTGAATTAACTAGGATCATAGTTTTGAAAATTTTGGGCAAGTGCAAAATATGGATTGTTGCTGAACCATGCCTGATGGATCCAAAATTGTTTCATTCTTTTATCATCAGATTCAGGGTGTTATTTGGTTTGAATGATTCCCTTGGTTTTCCTCGCCTACCTTTCCCTTAAATTCAACTTTGATTGGAACATTTCTAGTTTTTTTTTTTTTCTTTTCAATATGTTGGAACCAGGTGCTTTTATTATTTTTACTTTTTTTTAATTGAATAGGGGTAAAAATTGTAAATATAAATTTGACTATTTTCTTGCAAAAATTTCTACCCAATGGTGAGATATGCTGCTAATAATTGCTGCTTGCCTTCACTAATTTATTTGATCTTTGGTCTATGGCAAGTAGACAGTCATCCAGCATCTTCTAGAATCTAGAAGTAAATGTTTGTCAGGCTTTGATGCTTGTATCTCTAGTATTTTTGTTTTGTTTGTTTTTGTATGGTGAATGTTTGAATCATTACATTGATGAATGCTGAATATGTTTTGATAATGAGTTTAGGTGTGATGTAATACTTACCTTCCTGAAAGCAGGTATTTGATGCATTAAGGACAACAATGTTTATGCTTGGAATGATGTGTGTGTTTATTGGCATTTCTTTGCTGGCACCTGATGAATCAAAAGGTACTATAATTTGATAATTATTTTATGGTTTTATATAATTCAAGGTATGCATTGCAGAGGATAATACGAAACTTTGCTTTTGAGAAGTTTCAGGTCCTGAGACTAAAGATAGTTCTTTGGATTCCATGGTGTCTTCTGCCATATCTACAGAAGCTAACAGGTACAGTATGAACCCTTTGTATGACTTACATAAAGTGAAGAACTTGTAGCCCCTATAGTTAACTATATGAAACATCACTGAAGGGAATTGAAATGAGTACTAGTTCATTGTGTTCATTGTATCATCACATTTTAACTTCTGCATTGACCTTCTAAACCAGCCACAGTTCTCTTAAAATTTGTTGACAGAATTATATATGGTTTGTATTTTCCTGTTTTTGGTTTCCTTTATTTCTTCATGCGTGTTACACATGCTCATGTTAATGTTGGTGACTTTCACTGATGCCTATACTGTTTATCTTGTATAGGCTGGTAGTGTCTCCTGAAGAAGCACAAAACAAAGATACGAGATCATTAGTTAAAGCAATACTAATAAAGATTACAGATTTGTTGGTAAAGGCAAAGGTATTATTACTAGTTGCATTAACATTCGTCGCAAATGGTGCCACTTGTTATGATGATAAATTGACAATTCAAGCTTGCTTCTTTCTATCTCTTTGCAGACTACTTGTGCATTGTCTCTTGGTTTTGGGGAGGATACCATCAATGCATCATCAGTTCTTGTGATGCCAATGATGTCATCGAGAATGACTGGATTCAGAGGTAATGGGCTTGAGAGAGCAAGAATTTTGTCCATGAGAAATGGTTGGAGAAAGATCCCAATGGATGAAGATGCTGGGAAATTGCTTGAAACTAGTTCGGTTGTTCCTCCTAGCCCTTAATAGTGGTTGATAGTGTCATAGGAATGTCCCACGGTATCATTTTTTACCCCTTGATTTCCCATTTTTTTAATGATTGGAGTAGCTGAGATATGAAACCGCCACAATTCAAATTTATTTGCTTTGTTAGCATCATGTTATTGGGAAAAAGAGGATAAAAAAATTGTTGTATATTGTATATACATATTGTAAATTTTTACTTGCACAATTGAAATTTGAAAGGAAAAAAAAATCCTATCTTGTCTTA

>GLYMA.02G117100

ACAGATCAAAGTGAAGTGTTTCACCATTACAACTTTGCAACTATTTTACTCTTTCTCAAAACAATAAAAGAAATAGTTAAATCAGAACTCAAAATGTTCACAAATCAATGACGAAAAAATCATTGCTTTTCGTATGAAAAATTCATTGCTACCATTTGAATCTATCATATTTGGTAATGCAACAATATTCATAAATCTTCTTCTTCGTCTCTACTCTTTGAGAATATAAACCTTTTTAGCATCATCTTACTTTGACCCATCCTCCACGGCTCCACCTATCAAAAGTTTTCTGCTGATTCTCGACCTTCGGGTAACTTTACTCTCTCTCTCTCTTCCTTTCTGGGGTTTCAATTTTTTTCCAATTTTTTCTATTATTTGAATCTAATTTGATAGAAATCATTAATATGCAAAAGGTGAAATCTTGGTTGAGACTCGGGGGAATTGTGGGTGCAGTTAGTGGAAGATTAAGGAGTGGGAAGATGGCTATTACTGATGATTTTAAAGTTACGATTTTGAGTGTGAGATTTAGTTCAATTTATGGAGAATTTAACTGATTCAATTTGTTTATGAGTTGGGTTGAAGTTAGTTGATTGAAGGACTGTTTAGCGAGAATTGCTATTTTGGCACAGCACTAGTGTTTACAAGTTTAAAGGAAAGTAATTTTATTGGCTAAACAATTTTTTGAATTTGAAGATATACTATGCATTGATTGATGGTAACTACCAGAGCATTGGGTTTATTCCCCTTTAGTTTTGTTTTTCTCTAATTTGATTGTTTCCTTTTTTATTGATTGATTATTTAGGTTAAGGTTTTATTATTCAATTGGATTGAAATCACTTGTCTCAGTTTCTTGTTCCTTCAATTAAAAGTGAAAGTGATATGTGGTTAAAATGGAACATTCTTGTTCCTATTTGTAGTTTTTCTTATCGGTTCACGCGTCGCTTTTTTTTTTCTTTCTTTTTTTTATTATGGGGGAACAGGAAAGGGAACAAGAAATGGGAAGAGAGGATTTGGTTGCATTCAATTCTTCTATTAGAAGAAGTACATGAATATGACCTCTTTCCTTTTATTTTTGTCCTTCCCCAGTCCAGTAATTTGCTTCTGGGATATGTTCTTGTTCGACTAACTAGCACATTGGTTCTATCTTATCTTCCAATTTCAGGTCTGAATTTAAGAAATGCACAAAGTATGGATCACCTTAGAGGCGGTTCTTGATTCCGGTTATCACCTGTAGATCAAGGTTGTGTTGGAAATGAATGCTGAGTAGTGAATACTGCTGAATACTACCTCAGCTAAAAGAAATACTGTATCAGTACTACTTGTGAGAAAAGTATGATACAATTGCCATGGATGAAACACAAGACCACTATTATTCTTCCAGCCTACCCGAGTCTTCTTTATCTCATGATGGTGGTGGGAGGTCTTATTTCAATGGGCAAATAAATCGTGGGACTGCTATATCAGGCCTGAAGAAAAGAGGTCATGGAAGTCGCTCTTGGATTAAAATTGGTCAGGATGGGAATTTTCAGACTGTGACACTTGACAAGGCAACTATAATGAGATATTGTTCTTTGCCTTCTAGAGATCTCCGGCTGTTGGATCCGATGTTCATTTATCCTTCTACAATATTAGGACGGGAGAAGGCTATTGTAGTCAACCTTGAGCAAATCCGGTGTATAATCACTGCTGACGAGGTCATCCTAATGAATTCATTGGATGGTAGTGTTGGTCAGTATAGGTTAGAATTATGCAACCGGCTTCAGAATGAAAAAGCGGGTAAGGAAGGATTGCAGCTAATATTGCTGTAACAAACACAATTAGGTGCAAGGAAGACTTGTGTGAAAAAATAATGTCTTATATGTATCTAGGTTCGTTATGACTTTATTAGCATAACTAATGGTCTTTTCTGTTGTAGCATTGTTAGGGAATGGATAAAGAATAATAAGTGATAGACATTGAGTGTTGTGAGCTATGAGGTCTTTTCTTGATAATACTATGAAAGCCTAAGTTGAATATGTTACTTCTTCTGATGTTGTGATTTATTTCTTAAAAGCAATGTTATATTTGCTATACTTTCAAGCCATTCAGAGTTTTTATTTTTATTTTTATGGAGGTAATTACTGCATAGAATTGCTTACTTTGTCTGCCATTTTTGAACAGATGATCTACCTTTTGAATTTAGGGCACTGGAGTTGGCTCTAGAATTGACATGCACATCTTTAGATGCTCAGGTATGAATTGACCTAAAAAGTCTTATTTGTTGGGAAATCTTTCACATTATTGGCTGTGAATTTTTTATAGTACATTTTCTACATTTTTCATTTTACATTGAAAATGGGAACAACGAGTTTATATGCTCGCAAGTATTTTGGTTAAATTACTGAAGGATTTGTCATGTTGAAAAAATTTTCATCAACCATCCCAAGTATTTACCTGAAAATTCATATAATCCATAAAGCTTGATCGTTATTACACTTTATTGACTGTCACACAATATTTTCCACCATTCACTGCAATCAAGATTCAGGCTGGTAAAAGCTGTAGGAATAATGTAGCACTCAAAAGAAATATGGGGAGACAAAAGAATGTGCAAGGAGATCTGAAGCACAAACAAAGGGGGGCAGGAGGAATATTCTAGCTAATATCTTAGGGAACTATAAGAAAACTTCTGTTACTAAGGAAACTGGATTCTTATTACAGCCTTGCGACCCCTTTGTATAGGATGAAAGCACTAATAATTAATTAAAAAGCAAATGCTAACCCTTAGGTCTAAATCCAAACCATCTAAAAAGCTAAAAGGAACTAATAGGCATACACAGAATAGAAAATAGAAATTCATGGTAGCAACTAGTACAGTAGCATGGGCAGTACTCTGTTTATTAGGTTGATAAATATCAAATTATAGTATGTCTAACTCATGATAAAAAAAGAGAAGAAAGGGAGAAGGAAAATCCTTTTTCCATGTTTTTTTTTTTGAACTGCAAAATTAATATACATATATATATATATATTAAACAATAAATAGTACCAGAGGTACTACAGGATAGTACAGGAGATAAGCTCTCCAGAAACAGAAAAACAAAAACAGGAAAACAAAAGCCCTACAAGAAACCCCCAGAAACCTGACCCTACAGACCGCACTCTAAATAAAAGCCATAGACATGGCTGAGGACCATTGATGGAAAGGGGCAGTAAAGTCCTTTTCCCACCCCCTCAGCCAGGACCAAGTGAGAAAGTTAGTGTTATCCACCAACTTAGATATGTGCAAAGGCTGATTATGAAAAATCACATCATTCCGGAGCTTCCATATGGAACTTGTAGCTGCTATCCACCAAATTTTCCTTCTTGTATTGGTAACCTGCGAACCTACTATGGAGGAGTGCTGGAGGAAATGATCCATGGGCCTACAGTGAAGAGCTCTATCCTCCTTAACCCAGGTGTTGAATTCCCACCACAAGGGCATAACTTTTTGACAATTGAAAAACAGATGAGAAGCAGATTCAGAATTGCTTTGACAGAAGGGACACAAATCATTGTCAAGCTCAACTTGTCTCCTGGATAAGTTCTCCTTAGAGGGAAGCCTATCCCAAAGTAATCTCCACGCAAAGGATAAGGCTCTAGGTGGAATTTTAATGTCCCAAAGCTGGCAGAAACCAAGGTGATGATCTTTAGAAGACTGCTCAGCTTTAATGCACAGATAAGCAGATTTAGTAGAGAAGATCCCATTAGATGCAGCTCCCCACACCCAAGTATCCTTTGATCTAGCATTTAGATTGATCACAGCAGTCTGATCAATAAAAGCTGAAGCTATCCCCAAAATCCTTTTTCCATGTAGCAAAGACCTCAAATCAAATCTTGAAGAATGCTTGCTTGAAGTTGAGGTTACATTAAATAATTTATACTAAACATATGTAAATGCTAAAAAATAAAAAAGTATCCAAAATTAAAGGCATATTCATGATTAATAAACAAAAGTATCCCAAAATTAAAGACATATTCATAATTAATAAACAGAAGTTATAATTTAAAGCTAAACACACATAAACCAACAATAGAGTACTGAGTACGTAAATAAAGAAAGGCCAAAGCTGAGCATGAGTGTAGAGAATATAACAAAGCAAGTTTTAGAGGTCTTGAGTTGGCCAGAAAACACTGGGAAACACTGAAAATTCTGGAGAACAGTGAGAGTTCATCCAGAGATATTTACAAGGAGAGACTAGGGAGGACTAAGCAGAGCAAGTCTAAGGTTGGTTTGAAAATGCTGGAAAGTGTTGAAGATGCTAGAGAAGAGTGGGTGTTTGTCCAGAGAAAATGCTTGATTAGATGTGTGAAAACATAGGTTTATTTTTGAAGTCTTTGATTCAATGATGAGAGGGTGGGGGTGGGATTGAAATTGCAAGATTGCCTCCCAAAACAAAGCAATGTATGGACAAAAAAAGATGTTTCACCTTTTTCCTGCTGTAAACACTGTAATAGTGGTCACAACTTGGGATTGCAAAAGTGTTGCACTGCTGCTATTGCGCGCTATTGACTATTGTTGTTTGTGTTGCTCCAAGATCCACCAAGATGATGAAATATTGTGACTGATGAGTGAGAGCTATGAGGGATTATGAAAGATGATCAATGATAAGTGTCTCCACTCTATAATTCCCTACACTTTTGGGATGGCAAAAAAATCCGTGCCCGCGAATAAAATCTTTTATGGATAGAAGATGGATAGTTTAAATGGATACCTGCAGGTAGCGGGCTGGGTATGTGTTTTTTTTACTTGCATGTTAACAAAAAGGGTTTGGGTATCATAGTATGTGTATCTGCGAGTACCCGCTACCCATTAAATGACTTATATGACTTATTTGGTGTTCTTTTTTATTTGGATATGGCTCTTTAATTCACTAGTTACTGTGGTTGGGATGCATTTTACTAGTAGGAATTGGGTCCAGAGATTCAAAATCACACCATATATGGTATCGTTCTTATTTCTTTGATTTTTTTTTTTTACAATACAAAATCTGATTTCCTTCATTAAAGACATGTAATCTTCACATATAAAATCTGAAAATAGTACTCCCTCCGTTCCTATTTATAAGACCCTGATTTAAAATGGTGCTCGTTCCTTTTTATAAGACCCAACCTATAATGTTCCTTACATTAATTATTTTTAGATTAGAAATGCCCCTAATTAAAAGAAGAGAGAGGAATGAACTGGCAAACAATTAGAAAAGAGAGGAGTATTAATGACAAGGATAGTTTTGAAAAAAGTTATAAATTTAAGACAAATTTATTGCTACCAACTAAATTAATCACTTTCCTTAATCTTGATGAAGTAGGTAATTGAGTCTTATAATTAGGAACAGAGGGAGTATAACTTTATTTTCAAATTTTATAGCAAATAAAGGGTCTTCTTGATAAAGTGTCTTTTAAAAGAATCACCTTTTTTTTAATTTGAAGTATCCACAAGTATCCATGGATACCTGCGGGTAATTTTCAAAGTTCAAACAGATACACCTGGTGGGTAGCGGGCACGGATTTTCCCCAGCTGTGCAGGTCACGGGAAGCCACTACCCATGCGGTGCCTGACCCAACCTTTTGCCATCCCTAGTATTTTGTTTGGATAGAATTTCTTTAAATTTATACCTCATAGAATAGATATCTCCAAAATTATCTTCATCAAATTCTTTTGTTGATATCTTCATAAAATCTTTTTGTTAATATCTCCATCAAACGTTGTAAACTATCCTTCATCTGAACTCATCCAATTCAAGAAATACGAAGGAATGAGGGTTTATCATGGCTGTCATACTTCTCTCTTTGGACCTAAGTCATTGTAGTGTAAAATTTTAACGGCTTCAGACTAGGTATAGTACTGTATCTTGAATTTTGAAAATTGTTGTTTTAGTGTTGGAATTCAGGTAATTGTCTTTATATGCCAAATTATATGCCTTGCTAAATGCATAGAAAAAGACATTGGAGAAGCTTCCAGCCATGTGTAAGTAAATTTTCATCTTGAATCCCGTATATTAAGAAGAATGTTGATTTGATAGGTAAATGAACTGGAAATGGAAATATATCCTGTGCTGGATGAACTAGCCTCATCTATCAGTACTCTAAATCTGGAACGTGTTCGAAGATTTAAAGGTCACTTGCTTGCTTTGACTCAACGAGTTCAGAAGGTTGATCAACGTAGAATTATTATTCTTGATTACTATTCTCATTTTTCTAGCTTTCTATTACCTGCAACTAGTAACTTATTCTTAATTCCCAGGTTCGTGATGAAATAGAACATCTCATGGATGATGATGGTGACATGGCTGAGATGTGCCTAACTGAGAAAAAGAGAAGATCGGATACTTGCACTTTTAATGATTGTTTTCAAACTCGTGCATCAGGTAGACTAATTTCAAAGTCGGCTCCTGCTTCACCAGAGCGAACAATTAGTGGAGTCCAGATGTTGCAAAGGGCTTTCAGCAGCATTGGAAATTCTAGTAAACATGGTAGTTCAATGGGTTCGTCTGATAATGGGGAAAGGATTGAGCCACTGGAAATGTTGCTTGAAGCATATTTTATTGTCATTGATAATACTCTTAACACGATATTGTCGGTATGAGAAATTTGACTTACTCAGCTGTAGCATTAGAATCTACCCATGTTGCTTATAACATATAACAGAGTATTTGTATGTTGGATATCATAGTTTTTGCTAATATAACTAAGGTGTACATATATAACATTAAATTAGCTCAGGCATGCCTTCAGCCAATCAGCTGTTACATTCTGATTTTTGCTAATGAGTAGTCCCCATAGTGACTTTTTATGTAAGCTAACAGCGTATGATTTTATTATTTAGGCATGGTGCTTCATGCAAATGCTAAGTATTGATTTTTTTCCTTCTGGTAATGCAGCTCAAAGAATACATTGACGACACAGAAGATTTTATCAACATAAAATTGGTATGCTAAGATTAATAATTTGCAACATTCTCTGTCTATTCCTTTATTTCTTTGATACTCTAAACTGTGTAAACCAGGCAGCGTTACTTTTCACTCTATCTATTACAGGGAAATATTCAAAACCAGCTAATACAGTTTGAGTTGCTTCTTACAGCAGCTACATTGGTAGCTGCAGTATTTGCTGCTGTAGCAGGAGTATTTGGAATGAACTTTGAAACCACAGTTTTTGACTATCCATCGGGATTCCATTGGGTTTTGGTAATTACTGGAATTGCTTGTATAGCATTGTATTTTGCCCTCCTATTCTATTTTAGGTACAAGAAAGTGCTCGCAGCTTAAATTGAGTGTGCATTCTACCTAAGCCATGTTGAACAAGGTGACAGCTTGCTGCCGCATACAACATCTAAGTCTACTCATAACTCACTGCTTCAACACGGCTGGATACATTGAAGCTTTCATCATTTCATCAATCAAGATGCCATTGTTATACTTGGGCAACTATCCATGGTGCAGGCCAAGATGTCTCTATAATCCCGTGATATTTTGAAGGAGTCATAAACCTTTCCATCTCTGCTCTTCTTGTACTCAAACAACAAATTTGAATGATCAAAAGCAGTTAGTTTTACAAACCCATAGTCATAGTCTTTAAAGATGCTCCATTTGGTTTTGAGGGAAGTAAATGCTGAAAGGCTTGCTCCACCACCACCAGCTACAACATGTATGGTCCCATTTAAGGTTCTTCCCTTATAGTGGTGTTTCTCTTCATTAGTGCAGATATTCTGTTGCAAAATGTAAAAAAAGGTTCTTAGTCGAGAAAATATGCATTCTCATTCCAACTCTTGCAGGTTTAAATGATGCTATTTGTGGCATGTAAAATGGTATTGTTTCATTTTTCTCAAGAAGAGCATATTAGGGATTAGGGAACTCGACATCTTGATTACTTAATGCTTAATTCTGGATGTTGTGTTGTTATTGACTTATTGTACATGTTAGCCTTATATTCATCAAAATTTGAATGGAAGAAGAGCACAAGGTTTAAGAAACCTGATATATGGGGCATGTCCTCTCATAATTGTGCACATGGCCATAGATGGCAATGTCCACCTTGTATTTCTGCCAGAGCTTTTGAAAACTCTCTCTCCCCATTGGTTCAGCAAATGATCCCTCTTCTGCATAACAGATACAAGAAGAATATCCAAGTACGCGATGTGCAAGGAAAATTATCCATGGCTGTTTTTGTCTGTCAACTGATGCCAAACAATGCTCAATGAACTTGTATTGTTCTGTGCCTTCTCTCCAATCATGTTCAGTGTCAGCAATACAGAAGCGGAACATGCCATAGTCAATTGAATACCTGGTGAAAGAGTGTAGAAACGTAACATTTTCCAAATACCTGATTAATTCTGTTGAGTATTTTCTACCTAAAGCTTTAATACCAAAAGCATTGGTGGACTTTGGTATAAAATTATTTCAAAG

>GLYMA.02G280800

CTTTTCCTGCTTTACATGCCACAATCATGAAACAGGCAATGATGATGATCTGAGGAACCGTGTTTGTCTCGTGTCTCATCTCATCCATGAAGAGAAGGAGAAGAAGGGATCCAAAATCAGAAGAAAGTTACGATTTTTCGAGATGGGTCTGTCCAAGGAGAATCTGAAAGGTCTCATACTAGCTTTGGTGTCAAGTGGGTTCATTGGGGCAAGTTTTATCATTAAGAAGCAAGGCCTTAGAAGAGCTGCAGCAGTTTCTGGTGTCAGGGCTGGTGGGGGAGTTTTCTCAATTATAATTTCTATTTGGAAGCTTGTTTTTGGTTATGCATGGTTTTGTAATTTTTTTTTCTCATTTTGTAGGTGTTGGTGGTTATTATTATCTCTTGGAGCCACTATGGTGGGTGGGAATGATCACAAGTAAGCTTTAAATACTGTTTAATTTTGCTTCTATGCTTTTAGTATTGTCATTTTTGTTTTTTTTTTTTTTTGGGTGTAGAATCTAGATTTTCTTGTACCTTAATGGGTTTTGTTTTGTCTCAATAATTGCTTTTGATTTTATCCATTCATTGTATGGATTTAATTGGATTTTATTCTTAATAGAATTTTTGAATCCCGTAAGTTGGCTAGAATTAATTTCGTTCTGCATTGTATAGTGGAACCTGGATTTGATGTTTCAGAGGCTTTACTTATTTGCTAGATTTCTTTATTATACACACGCACATGGTAGATAAGTTGTACTGCAGTTGAAGGTTAATTAAAATATGCGAATTTTAGTTTATCATGGTCATTTTTTCTTTATCTGATTTCTTGCAGAATTCTTTTCTGTATGGTTTATTGTTCAGTGATTGTAGGAGAGGTTGCAAACTTCGTTGCATATGCATTTGCTCCAGCAGTCCTAGTTACTCCTCTTGGTGCATTAAGTATTATTGTGAGGTATTCCCCTTAATTTTCATTGTTGATTGAGAGTTAAGGTGGCATTAGCTTGTAACATTGGCGACATAACATGGTTGCTTCTTCAATGATGTTTGCATGGTATCCTCTAGTGCTGTTTTGGCTGACATTATTCTGAAAGAGAAGCTACACAATCTTGGGATATTGGGCTGTATAATGTGCATTGCTGGTAGTATCATTATTGTTATTCATGCTCCTAAGGAACAACCTATTACATCTGTTCTGGAAATATGGAATATGGCCACTCAACCAGGTTAGAATGCACTGTATTTTTTTAAAAATGCAATTCACTGAAGTTCCTTGATAACAAAGATATCCTCCTTGCTTGCAGCTTTTCTGGCATATGTGGGCTCAGTAATAGTGTTGGTTTTCATTCTGGTCTTCCATTTTGCACCAAGATGTGGGCATACGAATGTGCTAGTTTTTACTGGAATTTGTTCATTGATGGGTTCCCTCTCTGTAAGTAAATTAATTTACTGTTATCTCTCCTGCAATGTCTCTTTGATGATTGATCTAAACTTCAAAGTTTTGCTTACTCAAAGTTATCAAGTTTAAAACATATACAAACTAAGGGACCTGATTTCATGTTGAAAGTAATTTGGTTTTCACCATTCATTCCTATTTTCTATTAATGAAGTTCATTTCATAAATATAGTTTCAGCTTCCATATAATTTTTATATATTTTTTTTGTGTAGTTATATGAGACTTCTATGGCCTAAAGTTTTTTCCCTTTCCCCCAATTACATGGATAATTATTTCTGTCATAATAACTGATTTACTGAAAAATGTTTTCTTTGTAGTAACACTGCAATAATATGGTGGCTGATCTATATTGAATTGCTATAATTTTGCAGGTGATGAGTGTTAAAGCCCTTGGAACTTCTTTGAAATTAACTTTTGAAGGGAAAAATCAGTTAATCTACCCAGAGACATGGTTTTTTATGTTAGTTGTTGCTATATGTGTCATCATGCAAATGAATTATCTTAATAAGGTTTGTTACCTGATGTCTCTTAAAATTAGCATGCTGGGCCTGTCGTACATATATTTTTATCAATGACCTGGAAGTTCAAACATTTGACAAATTCTTAAGTTGAAATATAACTTCTCGTTGTAATTTGTAAGTACATAGGATGCAAATTATCTCTTTTCTATTTTCTGTTTCATCCATTCTATCTTGATCCAATTATTTCATAGGAACTGCTCTTTGTTTTGAGCCTTTTGGGAGGGGGGGAGAAAGATTTACTGTAAAGAGGAGGGAATAAAATACTAGGGAGGAGGATATCATTGGGCCTAAGAGGCTCAAAAATGAAATTATGCCCATGAATAAGGATCTGAGAGATTATGGAGAAGAGATAAACAAGTTTCACAGGAGGCAGAAGTGGGGAGGAGTGCAACATGTGGATGTGGTTGAAGGAGAGAGAGAAAATAACTGGTGGCAAGGGGAGAGAGGGAGGAGAGAGTGAGAAGGATTATATGATGGGTGTGATAACTGAATAGGGAGAATGACAAAATTGGTATCAATATTGGCTACCCTGAGTGAAGAAAAGGGGTTCTCTAATTTCTGCTATTTGTTTATTTTGTATTGTTCATCTTGTAAGAAGCTAGTCTCCACCTATTACCCTAGCTTTCTTTTTCATTTTCTTGGGTTGCACCACTGTAGCATAGGTTTATACTCAATTTATAGTATTTGGTGTTTTGAGGAAAATATAGGTGGTCTTGTTGCCAGACTCTTCCTTTGAATTGGAATGCAGGGAAGCTTAGCTACAACTTCATATACAATTGAAGGAAGATGACAACTGAACATAAAACAGAATGGAAAAATCTTACGAACTGAAACTCTGCTAAGCTATTTAAAAGATCTAGAATTCTTTTAATACCTCTTTTTAATATTTCTTAAGTTAATCTTATCAGACAGTAAGAGGGAAATTACATTCCACAGGAGGACTGTTTCTGATAAGATAATCAGTAATTGCTCCTATGATTTTACTAGTTGCATGTTAAATCCTCCATACAACATACAATATTACAACATGTGAATTCCTATGTAATGTAAATTCAAGATAGAACAAACCTCAGAACTCAAGACAGAGTACCCGACACTAAACCTCAGAACTAAGAGATTACATAATACAGCAAAAGTCAAATCAAAGATACAACAAAAGTAAATGGTTTTACAACAGAACTAGTAAAGGTTTGGTGTTTGGTACTCTATCATTGCTAACTGATAATCTTATCAGAAAGAGTCCCCCACTATCAATTGATTATAAGTTCTCTATACAGCATGCAACATGATTTTACTTTTATAGTATTTATTAGCCTGATTAGGCTGTTACTTTTCTGTTTCTTTACTTTTGTTGGCTTCTTAATCTGTTTGACTGTGCTATCCCTTGATGTAATGATTTGATCTTTTAGATGTTTAAAATGAAAGAATTGTTTAATTGTTGAAAATGCTGGCATTTATATTGCTAAACTAGCGGTATCAAGAACTCCTCACAATGACATAGTGATCCATTATATTTTGTGAAGTTTATTATTCTTTTTTCTTTATGAAGTCTTGTTTAAAATGATGTCCAATTTATATTCAATGGAAAAAGAAATCAATTCTAGAGAATTCAGATTTTCTGTGTATTATATACAAAGTTCTTATATATATGAAATTTTGGATCAATTACTAATTACTCTTCACTGACATGATTGTATTGTAATTATTTCAGGCTCTTGACACCTTCAACACAGCAATTGTATCTCCTATATACTATGTCATGTTCACAACACTTACAATACTAGCCAGTGTAATAATGTTTAAGGTATAATCAATTAGTTTTGGCATTTAATGCTAAATGCATAAGATGTTTCTGTTGTCTACGGATTGTACAAGAACCTATTTACATTTAAAAATAATGCTTACAGGATTGGGATGGACAAAGTGGTGGAACTATTGTGTCAGAAATATGTGGCTTCATCATTGTGCTCTCTGGAACAATAATGTTGCATGCGACTAAGGACTTCGAGAGAAGCTCTTCTTTTAGAGGTGTGGTAAATTTGATCATAGCACTTAATTCTGCTGCCACTGACTAGCCAGATGAATGCATGGAACCATCATTGATATTCTTTCATATTTGCATGTGTTGAAACAATTGCAGGTAGTGATCCTTTATCGCCTACGCTATCTGCCCGACTTTTTACCGGAAATGGTGACTCATTACTTAAGCAAGATGAGGAAAATGGATCTCCCGAGAGTAATATGCGCTCAAGAAGGCAAGAATTGTATTAGATGGTTAAAGATCTTAATTTATTTAATTGTAGGGGGAAGAAGAAGCCTTTCCCTGGAGAATCTGAATGAGATGCCCATCAGATTATACTACACCATGAAGCTGTAGGTACCAATGAAACCATAAAATGATGGAAGATTTGTGAAGCCATTCTTTTACCCTGAGCATCTTAGGAATTTATCCTCATAGATTTTAGTTCTGACTTTGAAAGATGGTTCTGGAACCAATGCTTACATAATGTATAGCTTGTCCACCGTTTCTTGTGTCATGAATGGTTTGTTCACTAGTCAAAAGTAGCTAATATGCTCCAAATAGTTATTAGATTGAGATGATTAGTTGTATTTGTGAGGCTCTCTTTACATGAACCAAAAAAGTGAGTGGGGGATGGAGATTATTAGTTTGTGAAAATTATATGCATGGTGATTTTGCTTTCTTTTTCAATTTTATTTATTTGTTTTCTTTGTCAATTTGACTTAGCATAGAGAGATTCATTTGTGGTTTGTAAGTAATGAGATATGTACAGGAAGCTAAGAGACTTCCCTCAATTTTAGTTTGCTATGAATAAGAAATATGTCCTCATTCTTACATTAACATACTATGTTT

>GLYMA.02G285600

ATGGCACGTTGGCGTTACATGCAAAGAGGTTGAGTTGGTGACCATGACCATCATAGCGGTTCTTCTCGTCTTCTTCTCCTATAAACAAAATGCCTAGCTTCTCTTCTCTTTGCTTCCAGTATGCCTCTTTTTTCTCAATTCCCTCTCCATTTCAACTTTATTCAACAGTTAATTTGTGTTAATTGTTGCATGCGGTTTTTATTTTTGTGCTTTGCATGATTGCATCCAAATTGAAATCATCATCATCATAATAATACTAATACTTCTATTTGCATACATTGCATTAATGCTTGTGGCAAGGTAATAGTAAGTTAGGAACCTTGTGATTGATATATTGATTCAATTTGTCTAGTTTCTGGGGTTTTATGTTGTGAATCATCATCGTGATCAAGCTGACATTAATTGTTCATTTCTTGTGATGACGATGCTTTGATTTTAATTAGCATTTGACTCTCTCGTCAATGGTTGTGGAGGCTCTAGTTCCTCTTCTTGAGAGCAACATGCAAGCTATGAATGAAGACTATTCAGCTTCATTTACCAGTAAAATGAAGAAGGAGGGGAATCACAAAACCTATTCTTCCACCAGAGATAGTAACAATAACATGCAGCAGGGTGGGGAACTTTGGACTAATGGACTTATTTGTGCTTTTGAATTCATGAGGGGAAATGGACCTACCAAGAAGAAGGATTATTGCTTAGGGAGAATTGGAAACAGTTTGAATGAATCAGATTCACATGGAGATGATTTTCATTTGTATTGCAAAGAGGATCTTCCAAGGAGATACTGGAGACCAATTGGTTGGGATAGAATTTCTGAACTTGTTCAGGCAGTGCATAGTGGTGATGCACAGCCGTTTGATTTCACGGATGATGAGAGTGATGTTCCTGTTGCAGATGTAGCGACTCCTTACTGGGAACGCCCGGTGGGGCCCACGTGGTGGTGTCATTTGGATGCTGCTGATCCTTTTGTTACTGCCTGGTTTGGTAGTTCTCGCTGGTTGCATCCTGCCATCAGCATCGCTTTGCAAGAAGAAAGTAGATTAATAAGTGATAGAATGAAACACCTTCTGTACGAGGTAATTGAGACTGAAAGTCGAATTTGCTAATTTTTTTTATTTTTAAAAAATTCAATTTGCTGCAAATCGGGTGTCTTTAGAAAATGTCTTATTGGTTGTTGTGCTTCTTTCAGTAAAATCAATGACTCAATGAATAGGTGAAAAGGCTTTTTGATTTTATAAAATAAGCTTTTAATTATTAGTGTTTATTTTGATAGGTTCCAGTTCGAGTTGCTGGAGGATTATTATTTGAACTTCTAGGGCAGTCTGCTGGTGATCCATTTGCTGAGGAAGATGACATTCCCGTTGTCTTACGGGCATGGCAGGCACAAAACTTTTTGGTGACTGCTTTGCATGTTAAGGGCTCTGCTTCAAATATCAATGTGTTAGGTATTTTAGAAGTTCAGGTCAGTTAGTCTTTTTGCCCCCTCATTCATCACAAACAGATATGGATCCTCTGCTGTTGTGGATCTAATCTAACCATTGAAGTTTATTAAATTTAATTGGAAGATGTTAATTTATTATATAATGAAATAAATTGAATGAATGGGATTTATGCAGTTCTGAGAGTAATTCTAGAGGATTGAAATACTTAATCAAAGAATTATTCTGCCAGAAGCATAATTCTTTTATTATTTTTGTCCTTGGTTGCCTTCAACTATGTTATACAGGAACTGCTGGCTGCAGGAGGTGCTAAAAATCCATGCAGCATTCATGAGGTTGTAGCACACCTAGCTAGCCGACTTGCACGATGGGATGATAGGTCAATACTATTTACATCTTGTTACCATTTAGATTTGTATTGGTTTTGCTGTTGATGATTACTGTTTTCCCTTTAATTTATATTTTTTTTTTCTTCCACTGGTAATTCTATATATAACCTCCAAATATAAGATAAACATAATCTTGATTGAATCTGTTGGAGATGGTGTAATTAAATTCACACCTGAACTATGAATATCCAAATTGCTAAGAGGGTGTGTTTATTAGACAATGCTTTTTTTTTTATCAAGGTAAAGTGTAAACAAATTGCGATTCTTCAAATTAAATCTGCTATGTGGATACAATCTCATAAATACAAGCATATATGCATAGGTATACTATTTCTAGATTCTATTGCTAGTTAGATATGATTAAATTACACACAGAATGGAGAAAAAAAAATTGTAATGAATATTCGAGTAAAAGAGTATGTACAAAATTTGGGAGAATATATGAAATTGCTTTATCTCCTTGTCGTCAGAATTTTAAGGAACATGATAAAGGAATCTGGCGGATAACACATTAAGACCAAATGTATGAAGTTCAAAATATGATTTAGGTCTATTTGGGTTGTCTGATTGTTGCAACTGGGGCTTCAACAACTACTGTTTGCAGTTGGCTATTCCTTTCCATAGTCAGCAATCATATGCATATATGCTAAAGAGAAACATACCGCATGATGCAAGCCGCCATGTTCCATGTGAAGTGGAAATTCATGGTGTATGATAGTCATATATGGTTTTTTTCATCCTCTTCATGAAAGTGAAGATTATGAATACTGGAAGTCTTCATCATATTTTATATGACTTAAAAAAAATTAATCTCGTAGTAGATTTATTTATTCTTGGTTTTTGTTATCCAAGAAAAAATAATAAAGAGAAAATGAGTTACTTTTGATTGGAAGGAACACATTGCCCTCTTGAAAAACATGAATAGAGAGTGAAACTAGGTTTAAGAAATCCATGACTTTCAATAGAAAGAATAAAAAGAAAGGCATTGAACAGAGAAAGATAGAAACCCCCTTTTTGTTTTTTCTTATTACTGTCAAAAAGGTTCTGTTCAGTCACAAGGTTCAGTCACATGGCTATAGTTTAGTTCTTGTAACTCAATTAGAATTTCCCTCATAGATCCTGCTTTTACGGGAATCAAATTGGGTAGTGAGCGTAATGGCTTTAGCTCCAGTATTAATTAATGGACGTGTTTTGTTTTGTTTTTTATTTTTTATTTTGATATATCTAGACTGAATTTGGTTGTAATATCACCCTGTTTCCTTCTTTCTCTTGAATTTGTTGTTAACAATATTCTCTGAAACTGATAAATTTTCTATTCTTAAAATCTGATGCTGTTATTAGCATAGGCTATATTAATCAGTTTTCTTGGTGATAATTATAGAAGTTTTGGATCTTTGTGAATGGTTTACTTTTTAACATTCTATCGCAATCTAAAATCATACTTAGTTGGGATCACAATCTAATATGCAGTGTCATCATAATTATTTCTTACTAATGAACAAGATATCATAGGTTGTTTCGTAAACACATCTTTGGGGCTGCAGATGAAGTTGAATTGATGTTTATGAACAGGTGATTGCATGTGCTTTGGACCACTACATATAAGAACTTTTGTTTTTTTGAAAGAATATACAAGAACTTAAGAAGTGTAGCATTACTAGCATAGGTGCGCATTTTATCTTTTTTGTTTGTAACTTGGTTACACAATGTTCTTTCCTTCCTGGTAGGAGGAGCCATGAGGATCTGCATCTGTTCACTATAATACTGAACCAAGAAATTAGAAGATTATCAACCCAGGTAATTATGCAGATTACAACATGACTAATACTTTACTGGTAGAGTCTGCTATAAACTTGTAACTTTGTTAAATAAAAATAAGAAATGTTGTAGAGAGTGAAACTTTTACATATCGCACAATTTCATTGATTGCACTGCTCTAATCATTTATTTAAATAAGTAATTATGTAAAAATAAAATAAAATTTGGTACATAGGTAATTATGTAAATTGTGTACCCAAAATTTTATATCCTTATCTCTTGTATGACCTTACAGGTTATAAGAGTGAAATGGTCACTCCATGCAAGAGAGGAAATTGTTTTTGAACTTCTTAAACAGTTGAGAGGAAATGCAGCAAGAGCATTGCTGGAAGGAGTAATGAAGAGTACAAGGCAAATGATTGGGGAGCAAGAAGCAGTTCGAGGTCGCTTATTCATAATCCAGGATGTGACGCAAAGCACAGTCCGGGCATGGTTGCAGGTTTTTAATTTTTTACCAACTACTGTGCCATAATCTATAAATTAGTAAGCTACAAATGTTTCATTGAATTCATTTATGTACCTGAGTTCATACAGTAGCTCTATAACTATAAGTATGCAAAGAATTTATTTGGCATTATTACTGGTGCACGATATTTTGTATCCAATAAGATGAAAATGATAATGCACTTGAATTGGCCATTTATCTTTGGAGGACTATAGTAAGACTTTAATTTTTTAATCGAGTTAAAAAGTAATTCTTTTAATATATTCATTTAATTGGCTTTTATATCTCTGTAGCATTCATAATGCAAACTAAAATTTGTAGTACACTAGAAGAGTGTGTGTGCGCATACGTGTATCAGTTAGTTAGTGTATACAGGATACTTGATGAAATTGTATAATTATATATAGTACTATAGTCTTGATATATATATGTATTCCTCTGCATGTTGGTGTCAATCCAGTTTTTCATTTTATTTTTATTCAAATAAAGAAAATAGAGAGAATAGGGGACTTCATTATGTTAGTTAATTTCACTCTTCCTACTTATTTTAGGACAGAAGCCTCACAGTTACCCATAACTTGGGAATATTCGGGGGTTGTGGCCTTGTTCTTTCCATCATAACTGGACTATTTGGCATAAATGTGGATGGAATACCTGGATCTTCAGGAACTCCTTATGCCTTTCTTCTATTCACCATGATCCTCTTTGTGCTAGGGGTCGTGCTAATTGGAATTGGATTGCTTTACCTTGGCCTGAAAAAACCCATCATTGAAGAAAATGTTGCACTGAGAAAGCAAGAGCTTCAAGAGCTAGTCAGGATGTTTCAACATGAAGCAGAAACTCATGCACAAGTACGGAAAACGGTGCCTCACAAGGCTCAAACTGCAGCAGTTCGGCCACCAAATGGTGCAAATCATCGTTTCATAATGTCAAAGTTGTGCAGTCACTAGATTTTTGTAGGGCTATGGATATATGGCTCAAAAAATTATCATGCTGTCTTTGTTGTTAGCCTATTAGGTGATACCAATAAAAGATATGTTGAAATGTAAAACATATTTGTTTCCAAGGTTGATTTGGCTTAAGGAATTATTTATCTCTTG

>GLYMA.03G159400

GCAGTCACAACCCCACCAACCTGTCACCAAATCAATTCATGCAAAGATATCTTTGATTGCTCCTTAATAATGTTCCAAATGATACCATCACTGTCTCTTCTTCTTCTCATCCCCTTCCCCAGAACCCTCATTCTTGATGGCTCTTGCTAGCTCTGTGGTTGAGCTTCAACCCTCTTCAGTGAAGAAGAAAACTGCAGTTTCCAGAAGTTGGATTCTGCTGGACCACTATGGCAAGGGCACTGTTCTGGATGCCGATAAGTATGCTATCATGCGCCTGGTTCAAATTCACGCAAGAGATCTTCGGATTCTCGACCCTCTCTTATCTTATCCTTCCACCATTTTGGGCAGAGAGAAAGTCATTGTTCTCAATTTAGAGGCAAATTAAACACTTTGTTTCCATCATTTGTTTTGTTTTGCCTATTAAATTTGTTTTAACATCTTGTTGATTTCTTCCTTTTGCAGCACATTAAAGCTATCATCACTGCAGATGAGGTACCTTTGATGTTGATAGAGTACTAGAAAAATCAGAATTAGCGACAAAAAAATCTGTATGGAATTCTTAAAAAATCCGTGACTAGTATTTAGTGACAGAATAACTTCTCTGTCACTAAATTCTTTTATTAGTTCATTCATTGATTTTTAATTTATAATGATATGTCTCTCATTTTGAATGATCTTATTTGGATAAATTTTTCCAAAAGCAATAAGAAGATAAATAAATGAAATAAAGTTCTCCCATAAGCCTTTAGAATCAAAATAGGACCAATGGTACTGTTTGTTCTTTGAGAGCTTACAAGTGATGCAGTGGAAATGGAATCGGTTTTCAGTTTTGGCAGGTTGTGTTGTTTGTGCTTGGTAATGCAGGTGTTACTAAGAGACCCAATGGATGATGACGTTGTGCCAATTGTTGAGGAACTTCGACGACGGTTACCCCAAGTAAGTGCTGCTGAGCAAGGCCAAGGAAAAGAAGAGGCTTGTGCTCAAGATGGTGAAGGTGGAGAAGAAAATGGTGCACCTTTTGAAAACATTTTAATTTAATTTATTACTTTAATCAAACAGTGTTAGTGTTGTTCTGTTTCGTGTTCTTTAGTTTTTGCTTCTGTGCAATAATGGAAAATTGGCTTTTATGGCTGGTAGAATTTCCATTTGAGATACGGGCTTTGGAAGCTTTATTTGAGGCAATTTGTAGTTTTCTTGACGCACGAACGAGAGAATTAGAGACTTCTGCTTATCCAGCTTTGGACGAACTGATCTCTAAGGTAAAGCCTTTTGCATAAGAATTGCTTTTTTTTTTTTAATGTTTTCCATAGATTTTTTACCTTGGTACGAGAACTTGTTACATGGCTACCAAGGTTTTAAACTGCGGCTGTGATCTCCATTTAGTTGCACTTATTGATATTGTGGGCAAATGTGTCCGATGTGGCCACAATTGCAATCACCAACAATTGAAAAACCTTGATGCTGTGGACAAAGTCACGGTTATGGACCTTTTTTAAAACCTTGGCTATGAGTACTTTAAGACATACATTTACACTGTTGTCCATCATATTTGTACCCAGTTTCTCTTCCTATCTCTCTTTTTCAATCCAAATCAATATATTTTTCCTCTATGATGAAGCAAAATGCATGGACATGATATGACAAGCTGTGAGTTTTGAATTTTCTGTACATTTTAAATGATTATTGTACAATCCCCTGCTCTGTCCATTTAAGCATGATAATTCTTTACAAGCTTAAACTATCTTGATATCATTGTATTGTCATTTGTCAGCAAAATATGTCAACTTAAAAGCTTGGCTATGAAACAATTTTGTTCATAATTGGTTATATATAATTCTTTTTATTTTTTTTTAAACTATTCTCCCCATTTTATGCTAGCAAAGCGGTTCTGGAGTGAGATATATATTGATCAGAAAGACAGCACATAATCCTTATGGACTAAACCTAATACTGTTACTATTTTTTTCTGCTCTAAAACTTAGATTGCCTACATATCCATATCAAAATCTGTTACATAAAACCTTGAGTTTGTTAATTAATAATTGATTTCCCATTATCTATTATGAGTTGTGACAAAATTATGACACTTTCTCCCCCTTTCAATCACTCTGGTTGAATGACACTTGTGTCCAGATCAGCAGTCGTAATTTGGATAGAGTGCGAAAATTGAAATGTGCAATGACGAGGTTGACAATTCGAGTTCAAAAGGTATGTTTAAATAGCAAATAGATTTTTTAACACAATTTTATTCCCTTCCCTCTCACAAGTGAATTCCCCCTTTTTCTTTTTTCTTTTTTTTTTAACGTGTTGTTTGCTATACAATTAACAACTGTGCTCCAAAATCTCTTCTGTGGTATTGTTGTCTTCATTACTTAAGTAGTAGTTAACTTAACTGTCACTGATTTCCCCTTTCTTTAACATATTAGAATCCATATTTTTCTGTTTGCATTTACTTCACTAGCTTTCTTGTAGATTAGAGACGAACTAGAAAGCCTACTTGATGATGACGACGACATGGCTGATCTTTACTTATCAAGAAAATTGGATGCTTCATCCTCTCCAACTAGTAGCTCTGATGCTCCATACTGGCTTTATGGGTCTCCAAATACAGGTTCAAAAAGACACAAATCAAGCAGAGTTAGTGGAACAACAGTTCAAAGGGAGAATGATGTTGAGGAGCTCGAAATGTTACTTGAGGTTACAAAACTTTTCATCTTTAATTTTGCCACAGATACTACCGGATTAGTTAAATGAATTGCATTAAACTGAACAACTGTTGTTCTTTGCCTTTGCAGGCCTATTTCATGCAAATTGACGGCACATTAAATAAATTGGCCACAGTATGAACTGCATTTTCTTTTAATCTTTCCTAGTATATTTCTGTTTTGTACTAATTAAGTGATAAACATTGCTATTTTGATACTTGCCCTCATTTCAGTTGCGAGAATATATCGATGACACAGAAGATTACATCAACATACAGGTAAATTTTAGGCACAACTGCTTTGTAGATTTTAAGCTCTTACTATCAGAAATATAGGGGGTAAAAGTTTAAATTTTTTTAACAGAATTTGAACACACCACTTATAGCATAACAACTCTTTGTAACATGTCACACGTGAATGCAGCTGGACAATCACAGAAATCAATTGATTCAGGTATAAGCTTTTCAAGGCGGTTGTTTTCTCCTTATGAGATTATGTATCAATATGCCTATTCATACAAATATATTTTTGAAACATTTGTTACCAAAGATATGATTGATGTCATTAATTAAATACTACTTTTATGAGGTTATGTATCAATATGTCTATCATTCTTTGGGACAGCTAGAGCTCTTCATCAGTGTCGGGACTGTCTGTATGTCCTTATATTCATTGGTGGCTGCAATATTTGGTATGAACATACCATATACATGGAAAGCACCAGGCCACGAACATGTGTTTAAATGGGTATGTACACATCACTTGCAACAATATGAGAAAGGAATTTCAAATATCCTGACCAACTTTTGTCAGATAAAGGAATGTTATGTATGTGTCATGTGTGAGTGAGTGATAGAGAGAGTATATAGCTTTACCTGATTATTTTCAATTCATTTTTTTTAATATAATTTTAGGTGGTGATCTTTGGGGGAATGGTTTGTGCATCCTTGTTTTTATCCATTGTATCGTATGCTCGACGCAAAGGCCTTGTTGGGTCTTGAAAAATGAAAAGCATAAAGCATGCATGTCGGGAGATATCAGGTTAAGTCAAAAGTGGGCAATTTCGAACCATTGCCTCAAAACAATACTACAGTAATGTGCTTTTATCTTGTAACTAATTAGCTCGCTATTTGGATAAAATGGCTATTGGTTGGCAAGATGAATGCACTGAATATTATTTTTATAATAATTAGTTGCAAAACCCACTGTTAGTTTTATTTTTTATTTTTAGTTCATATTCCTTTTAGAGTGAATAATTCACTATAGTAAATGATCGAATGAGTAATTTCAATCATTGATGATAATTATGGGTGTATTGAAATATTAATCATTAGTTTTTTCCATCAATAATTAAGATCACTTGCTTTATCTTGTATATCTTAGAATAGCCGATTCCTTTCAGTACAAGTGACAAGAATTATTTGACTCTCAAGAGAAATTTAGCCGATTTCACCTGC

>GLYMA.04G005200

AAAAAAATGATGAAAAAGTGGTGAAGTGTGTAGAGAGCGTTAGAGTGAGAGTGATAGTGATAGTGATGGGGATTGCAGAGAACTCAAAGGGTTTGGTTCTTGCTGTGGCATCGGGTGTGTTCATAGGAGCAAGCTTCGTCCTCAAAAAGAAGGGTCTTAAGCAGGCTGCCACCCACGGCACTCGCGCAGGAGTTGGCGGCTATTCTTATCTACTCCAGCCACTTTGGTGGGCTGGCATGCTTACAAGTAACTAACTAACTCACTCGCTCTCTCTGTTAGACTCTTACTATGCTTTCTCACTAACACCAACTCTTTCTAATTCCTCACAGTGCTTATTGGGGAGGTTGCAAATTTTGTCGCTTATATATATGCTCCTGCACTTCTCGTTACTCCCCTTGGTGCACTAAGTATTATTGTCAGGTATTATATTAACAACACTTCTCATCACTCTTAGTATGACTTTCTTTTCAAACTCACTCAACCCCATCTTTATCTATCTGGATATATAGTGCTGTTTTGGCTCACTTCTTGCTCAAGGAAAAGCTTCAGAAGATGGGCATTTTGGGATGTGTCTTCTGCATTGTGGGTTCAGTTCTCATTGTCATCCATGCACCTCAAGAGCATGCTTTAAATTCTGTCCAAGAAATATGGGATCTCGCCACTCAACCGTGTTTGTTTTTCTTATTTTTCACTCTTATAATAAGTTCATTTCCGGTCCATAAAGTGGTGTTTCTCATAATCTCTCTTTTTTTCTTCAGTATTTCTAGTTTATGTGGCGGCGGCAGTTTCGGTAGTTTTAGCCTTGATTTTGCATTTTGAACCTCGCTACGGACAGACAAATATGCTGGTTTACTTGGGAATTTGCTCGTTAATTGGCTCACTTTTGGTACTTTTGTTTTCGCCAATCACTTTGATGGAAGCTTCATATTGATATTGTAATTCACAAACTAACTAAACTATTCAGGTTATGAGCACAAAAGCCATAGGGATCGCAATCAAGCTTACATTGGAGGGAACAAGTCAATTAACATATCCTCAAACTTGGTTTTTTCTTACCGTGACTGTCATATGTATCATTACACAGTTGAATTACCTGAACAAGGTGAGTGCTTAGTTATCAAATTTAGCCTCATGCAAAACCATATATTATTGTTCATCAATGTATTGAAGGTCACCTTTTTCTATATTTGTTTAAGCATTCCACAAATTGCTGTCTGCTCTCTTTGTTCAGTATTGGTTGAGGTAGAAGCTTGTGTTGATTGACTGACTATCTATGTGCTGATGTGCATGCTATATATGACCTGCAGATGCCTCATCATCTTTTCAAAATCTTTTCTGAAGAGGTTTTAGTAGTGCAATTTAAGGAGTTATATATATTATAGCCAAAATATGCGAGGCAATAGCATTTTCTGTAAAGTATATTATCAATTGGACCCACTTTACTAGTTAATTACATTTAACCTATGGTGTAAAATTATTGAGAGTGGAAAACGATACGTTGTGCTTGATAATTTGATAAGAAACTATAAATGTAGTTGGCATCAGATCAATCTTTAGAATTTTCACATTTTTGTGCATGAAAATATTGAGTTCTTGCACTTGATACAAACTTAAATAAACAACCCATTCAAATTAATTCTTAGAAGTATGAAGCCAAATCTTTTAAATGGTACTCTGTTAATCTTTCGGCATGTGTCTTTGATCTAAAGTTTTCTTATTTCAATACTGTTGTTTTTACTGCCCTAATATGGCTTTTCCTATCAGAGATCTAAGGCCTATCTTAGTTTCCTTGATCTTGTAACTAATCTTGAACATTTTGGCACTATGGAACTATGTTTCAGGCACTGGATACATTTAACACCGCAATTGTCTCTCCCGTATATTATGTCATGTTCACAACTCTGACCATTATTGCTAGTGTGATAATGTTTAAGGTATCATGACATATTCATATTCTTGGATTCATTTGGTGCTATATGGAAGGTCTTCTTTTGTTGAAAGCTTGACTTTTTGGTTGCTTTAATTTTCTGTGCAGGATTGGTCTGAGCAGAGTGCAGGTAGCATAGCCTCTGAGATATGTGGATTTGTCATTGTTCTTTCAGGAACAATCTTATTGCATGCGACAAGAGAACAAGAACAATCCAACAAACAAGGTACAAATTACATGTTACTTTCAATCAATTGCGCATGCCTAATTACCAATCATCAATTTATTTTTTGTTTGAAATGTGTCCAGGCCGAAATCCCTTGAATTATGGATTCGTGATTGTATCTTCTTATTTCCTGTAATTATCAATTGTGCATAGAATCTCTCGCAGCAGAAAGAGATTTCTTAATTTACAGGGAGTAAACCTGATCCAGTGATTACTTTGAATTGCTATTTGATGCAGGGTCCTTAACATGGTACATTGGTGAGGACTTGGTAAAGCGCATTGAGGATGGACACCTGAACCTTTTACATGGTTCAGATTATGTTGAAAAGTGACTTTTCCCGAGATTGACGTAAGAGAACCTATTCTACTAGCATCTAGCAATAATACAATGGTATAAACCTAGTGGATTCATGATTTGGAGCATTGCAGTGTAGATTGCTTGTGATGCTATAGTTTTCTGACTATTTGCAGGCTATTGTATACAACTACCATAGGAACCATCTTATACAATGTGTGATTCGCTAATCGCGATTCAAGCAATCTTTTTCAAACTGAAATGGGGATTGCTACCACATTGCAACACTCTTAAGAGTGATTTCATGGTATGTTTTATCATCCATGATAGCCAGTGCAATGTGTTTCTTTCCTGCTTTTTTTGTAGGACGTTAGCTCGATCGTACTTGTTATTCAGTTACCTGATTCTATTGATTCTTTTATTGATCAGGTATTAATATGTTGATTTCGGTTAACGAGCATTTAAATTTATTGTTAGCATGATCTTTTGTAGCAACTGTGGATTAGTTATTAGTTGGTGGATATAGTTTTCTTTCAACTCGAAACAAAATGAACTCAA

>GLYMA.05G153000

GTGAGTTGCGGAGAGTCCTAATTTGAGGTAATTGTTTCTGCAACGCATGCGTGACACCCATACAGTTGCGTCTTGTGTCTCCATTCCAATTCCATGATCTCTTCCTTCCTCACCTTTGACATCCACAATTAAGGCTTCAATTTTCATGCTCAGACAACCCTTCTCTTTAGTTTTGTGAGAGTCATGATTTTCTAGCCATAAAGTTGTATGTAAGGTGCCCAATAAGGTAAATAGCATACCCCTTCTAATGTTTAATGTTTCTATGTCTATACATGCATATTTAATGTTATACAATTCATTCAATTAATTTTCTTGTTAATGCATTTGTACGTTGAATCAATGGCTTGTAGGGTCAAGGGATTGAATTGGACATCTTGGCCCTGCAATTGTTCGTATCATCGAAAGATATTTTTTTGGGTATCATTTGTATGATAGTGACAAATGGGGGCGTCTTCGGACAACGTAACTGGGTTTGTTTTGGCTGTCTGTTCCAGCGTTTTCATTGGCTCTAGCTTCATAATAAAAAAGATGGGCCTTAAAAAGGCTGGTGCCACAGGGAAAAGAGCAGGTTCATGTTTCCTCACATTTTTGTTTTTGGTTTGCTAGGTTTCGAATTACCCTTTCTTTAATCATTGATATATACATTTATTTTGTTTTAATTTGATCAGGCGCGGGAGGACATGCATACCTATATGAACCTTGGTGGTGGTTTGGAATGATCTCTAGTATGTGATTTTTAGTTTTTTCTTAAAAATTATTTTGGTGCTGCATTTACTAAATTATAATATATCATGTAGCATTTTACCTGCAAAACACATTTGACAAGGCCTAATCATAAGCTACCTATTTTAATTTAAAGGGGATTTCTTGTGACCAATTGTATTGCATGTTTGATTTAATTCTTCTTTAATTGTCTGTTTGTATTTATTGTATTAATTCATGATACAAGCTTTTTTCTGTTGTGCATTTAGGTCATGTAGCTTTTAGACAATATATCAAGGTTTGGGTGTCTATGTAAAGCCGAGCCACCTTTATTATTAATTTTTCAGGTTGAGGCTGTGTGTTTTAGCTTGAAGCAAAGGAATAAGATGCTTGAACTTTTTAAAGTTTTTCAATTGGAAAGGTGTTTCGTTGGTTGTCTTTATCAGCTAATTTGACATTTTACAATTAAGATTATAATGGAGGGTGCAAAAAGATAAAGTGATGCAGAATAATTTCTATCACATTTCAAGTCTAGTGGACTACCAAAAATTCTTGAACCCAACCGATTTAAGTTTAATTCTTCTTAGACCAAGAAAATTATTTTTATTACTTTTAACTTTTAAGTTCTAATACTTTGACAATGTTTCCTTTGACTTTGGATTCAAATGTCTTAGTACATACATATAAATTTCATAGAGAAAATTTTCCATTATAACTCTTAGTACTCTTATAACTATAAATAACTAATTTGCTAATGTCCGCGACAATCTTATGAAAATTTCATCAACTAATTCTATATGTATGCTTACATTTTTGTAGTGATTGTTGGGGAAGTAGCCAATTTTGCAGCTTATGCATTTGCTCCTGCACTACTTGTAACTCCTTTGGGAGCTTTAAGTATCATTTTCAGGTAAGGAACATGGTATTCTTCATAACATTAATTATTAGGATGTTTCTGCTTTCTTTGACACAAATGTGGATGCACATCTTCATCCATCAAATTTTAATTTGGATGCTGCTTGCATTTTAGAAAGTTACAAACTAATATCATGATGCAGTGCAATACTAGCTCACTTTATCTTAAAAGAGAGGTTGCACATATTTGGTGTGCTTGGATGTGCTCTTTGTATGGTGGGATCTACAACTATTGTATTGCATGCTCCCCATGAAAGAGTTATTCACTCTGTTAAGGAAGTGTGGCAACTTGCTACAGAACCAGGTAATTTTCAATTCCTGATTGCTTGGTTTCAACATACCTTTTACCATTGGATCTTGTAAAAAATCAAAGTAGGAGGATACAAGATGAGTGGGATGCATATTAAGGTCTTAATAGATTCAAGGCATTCATTTATGGTTAATTAAGCTGACATTGAGTTCTTTCCTTCATCAACAGGCTTTCTAATCTACATGTGTATAGTTGTGGTTGTGGTTTGCATCCTTATTTTCTATTGTGCTCCACGATATGGGACAACCTATCTGGTTATATATGTTGGAATATGCTCTCTCACAGGCTCAATTACGGTATATCGATTATGTCTTCAATGCTCATATGTGTCATGTAGGTCATAATAAAACTACCCTCTACAAACCTAATGTTGTTCCTTGTTGCATAGGTTATGAGTGTGAAAGCAGTGTCAATAGCTATGAAGTTAACATTGGAAGGCAACAACCAATTCATTTACTTTCAGACCTGGTTCTTTACGATTATTGTGATAGGATGTTGTCTTTTGCAGATTAACTACTTGAACAAGGTAAACTTTGACTTACATATATTAGTACATTTACTGTGGCAGTCATTGTAATATGTCTTCCAAAGTACAACTTAAAATAATTATGTCAAAGTTAAATATATTTTAAAATTTTTTTATTGGTTATTGGAGTGCTAATGTTGAAAACACTATTTTGATTTTTCTATGCCCTTACTTAGTGATTAGGTGGTAAGTTGTAATGCTTAAGAAATTTAACTATGCTTTTGCAATCTTTGGGACTTCTACCATCATCTAAATGACCTTGTTTGTATTTCTAATCTAGTTCGTGGCATTTGGAAAAGTTATAAACAAAAGATTTTGTATTGCTTTTCTTAATTGGCATTGAACTATTTCATTCCCTTTCGTTGCCGTTATAGATGAATAATACTTAACATGCAATATTTTAGAAATGGCATGCCATATTTTTTACATTAGGGATTTCCAACTAACTTACAATCGAACTTCATTTTAGCATTCGCTTTAGACTTTCTTTAATAATTAAAACCAATAATGTTAGAATCATTGAAATAAGTGTGCTGAGTTTGTATTTTAAGACAATATGCTTATTAGTTACCAATTATGTTGTAAAGCTATTTTATTTATTTTGGAAACATGTTTCAGGCTTTGGACACCTTTAACACTGCAGTTGTATCACCAATTTACTATGTCATGTTTACATCATTCACCATCTTTGCCAGCATAATCATGTTTAAGGTAACTAATTGCCTTCTATGATTCCATATATATATATATATATATACTTTTGTTTAAAATGTACCTGATGATACTACTAAATTATGCAATCATATAGTAGTTTAAAACAATAACAACAACAAAAGCCTGTTTCCACTAGATGGAATCAGCTACATGAATCAAAAGATACCATTGCGTTTTGTCAAAAAACCAATTCTTCAAAAATGCTATTTAGGATCTAAATCATTTTTAAAAATTTTACTTGGAGTTTTTATTAGTTTTCCTCTACTTAGTTTTAACTATTGGCGATCACTTATCTTTTCAACTCTTCATATAGACATATGGCTGTTTATGGAGATAAAACACAAACTACCTCTTTTCTTGTTGTGCAGGGAAAAGTAAAACTGAAATGAGAAATATGAAAGTGTGAGAAACTTAGTCATCCATAGTATTGGACCAATCATAACTAGAATTTAACTTGCATAATAATATCAATTTGAATACAAGTTTTGAAGAACTTGGACAACTAAGATCTATTAAATCAGACAAACTGAATTTAAGATTATATCATATAATTTTGGCAAATTATTGCTGAGCCAGCGATCTGCTCCTAGTTTTAAGTTTTGGTTGCCAATGGCATATGATTCATTCTTGTTTGGTAGTTGTTTGAAAACATATTCCTTAAAATGTCCTCTTCGTGTAATTTCATATTTTGTTTTAAAAGCATGCATGTTTTGTTTGCTAGTACTTAGTAGCTGAAAATTCTAAGACAAAGTTTATTTTGTTTATTTGTTATTGTTCTATTTTATTATTTAAAGGACTTTTTTTAAGAAAAAACAAAAATGACATTTAATTCTCACAGTATATTACATGGCATGATTAACATCTACAATTGTCAATTGATCAATTATCATTATTGCTATTGTCTTGTTAGACTCATTTGTATTATTATGAGTCTTAAATAATATCTTCGTCGGAAGTTTGCAAATGGATTGCAAGTCATTATCTGCTTTATTTATCTGCTACAGGAATGGGACACACAAGATGCATCTCAAATTGCTACCGAGGTTTGTGGCTTTATCACAATTTTATCTGGGACCTTTCTTCTTCACAAAACCAAAGATATGGGAAATAGACCCATAGAGTCTCCTGTTTTTGTAAGTACTCCACAAAATGTTAGTAGTCACTCAGGGACTTAATAGCCATGCTTCTTTTTTTTTTTTTTAACTCTGGTCTGGACGACAATGGTATTTCACCTATGTTTCCAAGCTAAGCAGACAGCTTTCAACAATGGAGATTAAGCTATGGTATTTCAAGGAGAAGAAGGTCAGTGAATGAGTGGTATTGGCTGGTTCTAACAAGCTTGTAATGTCAGAAAAGGCAAAACAGAAATATGAAGCGTGAAAGATTACAAGAAGCATTTCAGAAAATATTAGAGAATAATGCTGCAATTTAGTTTCTTGTAATTTTTTTATTTTATTTTCCCTTTAACAATTTCTATGAGCAGATGATTTTTATGTGAAATTGTGAATAGCTAATACTCTGAAGTTGGTCTTCATTAATATGAGATCACAATCCTTGAAATCCATTTGAAGGTCCTATTATGGTCTTCATATACTTCAATGTTGTCCTGATTCACGCGTAAAGGAAAAAATTGAAATATTCATTTGAGCGA

>GLYMA.05G168200

TTGCATTTGTTTTGATCCGTGGGTGTGGGTCGATAATTAAAGCGTTATTCGTCCTCAACATAAAAACAAACAGGGAGTGTGCGGCAGAGGAGTGTCTCGTTTATGGAGGATCAAAGCGGAGAAGGTGAAGGAGAAGAAGAAGAACAACAACAACCTTCACTCTTCTATTATTATTATTATTATTATTATTCAATTTAATCATTTAATAATAATAATAATAATTTTGTTCACCAATTATTATTATTCTACATCAGCATGGGTAAGGGTCCCTTTTCCTTCCGCCGCTCCGCCTCTCGCCGCCGACCCAAGAAGACCGCCGCCCCGCCTCCTCCTCCGTCTCCGCCGCAGCCTCCCTACGCGGCCGGAATCGCCACCTCACCCGACGACAACAACAACCGTCTCATTGCTGCCGGCGCCGGCAGCAGCGCCTTGACGAAGGCGAAGAAGAAGACCGGCGGCGCCCGCTTGTGGATGAGGTTTGACCGGTCGGGCCGGTCAGAACTGGTGGAGTTGGAGAAGAACGCCATCATCCGCCACGCAGCGATTCCCGCTAGAGACCTGAGAATCTTGGGCCCCGTCTTCTCCCACTCCTCCAACATCCTCGGTATTCCAATTTCCCCCTCTATTTTCCTTTTTCAATTTCATCGTGTTCTCAAAATTAGGGTTTCTCTAAATGAATTGAATCACGTGTCTTAAATTGTGTTTGTTTTGGTAACTTGACTGTGGTTCTGAGTTTTCTTTTCTTTTCTTGGACGGTGTTATGACTATGATGAAGAATGAAGAATAGAATTATAATGTGTCTTTTTTTATAAAAAAAATTGCAGCTAGAGAGAAAGCAATGGTGGTTAATTTGGAGTTTATAAAGGCAATCGTGACTGCTGAAGAAGTGTTATTGCTTGATCCTCTTCGGCAGGAGGTTCTTCCCTTTGTTGAGCAACTCAGGCAACAGCTTCCTGGGAAAAGTCAACCTAAACTTCTCGGCGGCGTGGAGGAACAGGAAGGCGAAATGCAGGTCTCTAATGGCAGGCAATGGTTGCCTATGCCGGAGGCGGCTGATGGTTTGCAGTCAGAGCTTCCGTTTGAGTTTCAAGTTCTGGAGATTGCTTTGGAGGCTGTGTGTACTTATCTGGACTCGAATGTGGCGGACCTTGAGCGAGGTGCTTACCCTGTGTTGGATGAATTGGCTCGGAATGTTAGCACCAAGAATCTTGAACATGTGAGGAGTTTGAAGAGTAATCTTACGCGGTTGCTGGCAAGAGTGCAGAAGGTATGTTTTTGCATAAGTACCATGTGGTTCTTCCTCTGATTGTTGCTATAACTAAGTTGATGTGGATAGTTATTTTGTTACTTATAGATGACTGTGGAAACTGGAAATTGAGAATAGGAGGATTAACCTTTGGGAAAACAGATGTACCAAGTCCAATTTCGTTTTAATTAATGTAGTTTGTTGGACTGGTCAAGCCACCAGTTAATAGCCCATGGGATGTGCAAGCTAATAAAAGTGCCAACAGATTCTTGTAGTTATTGTGCATTGTGATGATTGAGGATTTTTCAGAGAAATAACATGTAATTGGACAGCAATCTGCATCCAATTCTGCAGTTGTTGAATAAAGAATGAAAGGCTATCTCTTGCCAAACTCTTTTAATTGTTGAATTTCTTTTGTTACATTGTTCAATAGTCATTTCTTATGCTCTTAAGTAGAGGGTTTTGTTCACTGTTTTATATATAGAAAATTTATTGCAGGCGGTTTTACCTTGTATGTGACGATGCATTCCTTCATCTTTGAGATATCATACTTAAAGTGTATGACCCTCTTTCACAGGTGCGAGATGAAATTGAACATCTGTTAGATGACAACGAAGATATGGCACAACTATATTTGACGAGGAAGTGGTTACAGAATCAACAATTTGAGGAGGCTCATTTGGGTGCCACAACCTCAAATAACTTTCCTAATACCTCACGTTCTGTTCGTCGACTTGGTTCTAACAGAAGTGAAAGTCTTGTGACCTGCCATTATGAGGATGATAACAATGTGGAGGACTTGGAGATGTTGCTTGATGCATATTTCATGCAGTTGGATGGAACTCGTAACAAAATATTATCTGTAAGTATTCTAACAACCATACTCTGGTCAAATCATGTGTCTGTCAAATTTTGAAGGATGAAACTGTCGTTTTCTACTTGGATTCTATCATCTATCTAATTTTTATCACACACTTGTGCTTTAAGACGAGAAATTTCATATATTTCATTTGCATACGTGAATCAACCACATAAACTTATTTGTGACTTGTGATACTTACCAGAGATATAATATTGCATTATTTTACTTTTCTCTTGTCTTGAGGATGGAGAGAAGCTCAATTGTTTGCTTGTTTCAATGCAACACTACTAGGAGCTTATCCTTAAGAATTCCCAAGCTCCAATAAATTAATTTAAGCTCACCCTCTGTAGGATACTGGTAATGTATTTTGTCCAAGTTTGTTGCAATTCTGGATTTACAAAATATATTATTGCCTGCCCTGTTGATTTAAAAACAAATGGTTGTAACCTACTGTTGATTTTCATTTCTTGTTTATAACAAAATGGTAGAAAGTCTATATTGTTAATGTTGAGGGGCGAGGAAGTTAGTCTGTGTTTCTTAGACCAAGATGTTGGGCTTTAGGGAGCATTTGTGTTTGTTGAAATGTCAGCTGATTTTGGTTATTGTTTCTGGAATGCCAAATATTGTGAGAATTTGACTGGAATCTTGAATGCTGTTTCAATTTATTGTTCTTTAGAACTGTTAAGGGTCTTACTTCTTTATGTTTACTGATTTGCAGGTTAGGGAGTATATTGACGACACTGAAGACTATGTCAACATTCAACTTGATAACCACCGAAATGAACTTATTCAGCTGCAGTTGACGTTGACTATTGCATCATTTGCTATTGCTATTGAAACTATGATTGCTGGTGCATTTGGTATGAACATTCCTTGTAACTTGTATCACATTGATGGAGTATTTTGGCCCTTCGTTTGGATCACGTCTGCAGCTTGCGTATTGCTTTTCTTGCTTATTTTAGCATATGCAAGATGGAAGAAGTTGCTGGGATCATAAGGCAAGGCTGTTGTGACAATTGGCAATTTGAATCTCCGCCGCGAGTGATCTGAAGAACAGACACAAACGTGTCACCGGAGAATAATTGTAATAGTTGATGTATAAGATGTTCCAACATTGCTTTTAGCATCATGTGTTTTATAGCCGATATTATTCACACCATCATTTTTAATTTACCAACTTTATGGTGTCGCGTGCATGCGTGTGTGTTGGCCATTTTGTTCTGGGTCCGTGTGTATTTCGGCTTGTTCAATTTATATGAGCATGACAAATGAAATTGCATAAGGAAAAATGACACTGTGGGGGCTTGATTTATATGAATAGCCAACCACATTGTACAAGAGGGTTCACTTTGTAAGCAGACAAATCGACAACTATCAAGAAAATTGGGATCTTCTCAATTCTAATGAGGAATTTTTGTACTTTAGGTAGAGTAGTTGTTGTATGTCTGTCTCAAATTTCAATACTTCTAGTCTGCATTGGATTTTTGCAAACATTGCAATAATCTCTAATATATATTTTACACTAACGGTATATGAGT

>GLYMA.05G196600

GACAAACTTTATCTAGAGATGCGACCAAAGCCAAGGCCCGTTCCGACAGACAAGAGCGACAGAATTTTAATTGATTGCAAAATAGAAAAAAAAGAAAGTGAAGCGTGTTAGTTAGTTAGTAGTTGGTGCGCTGCGTTGTGAGTTCGAAAGTGAATCTAGGAGAAGAAGGAGTAAACAACACAACCAACAAACATCTTCGATCTTATTCTAGGGTTTCAATTGTGATGTGAATCTAATGAATGTAACGGGAAAAGACTGTAGATAGATGAGCTCCAGCAATTTGACGGGGTTTGTGTTGGCCGTGCTTTCCAGCGCTTTCATTGGCTCCAGCTTCATCATCAAGAAAAAGGGTCTCCAACTCGCCAGTGCCAATGGCCCACGTGCCAGTCAGCCTCTCTCTGCTCTCACCCAACTTTTTCTTTTCTTTCTTTAAAAAAAAAATACATTTTTCTGGGAAATAATGTTTCATTCGTGGTCGTGTTTTGCATTGTGGTTTTTAGGTGTTGGTGGCTATGGCTACTTGCTTCAACCTCTCTGGTGGGTCGGAATGATTACCAGTATGCAACATTTTCTCTTTTCTTTGCTTTTTTACCCTTTACTTCACCCATTTCCAGATTTTTTTTTGTTGCTGATTTTGCTTTTTTGTTGTAGTGATTGTTGGAGAGATTGCTAATTTTGTAGCCTACATTTATGCCCCTGCTGTTCTTGTTACTCCACTCGGTGCTTTGAGCATTATTGTTAGGTAAATATTTATTTCCATTTTATACTATACTAAATACTATAGCTGTTTCCATTTCATGTTTTAGTCCCTATACAGTTTCTTTGTAGTGATTTTGTACTGCTACAAAGTCTTCTATATTAGTGAGAGGGACCAAAATTTTGTCAGGCATGCATGTGCAGGGACTAAAACTTATAATTTTCTCGTATAAAAACTAAAACTTAAAAATGGGGACAACTATAGGAACCACGTGAATAGTTTAACCTTATTATTAAGGAGCTGAGAATTGCTATGGATATGGGCAGTGCTGTCTTGGCACATTTTCTCTTGAAGGAGAAGCTGCAGAAAATGGGCATGTTGGGGTGTCTTCTGTGCATTGTGGGATCAACTGTTATTGTGTTGCATGCACCCGAAGAGAAATCTCTTAGTTCTGTACAAGAAATATGGGAATTGGCCATTCAACCTGGTATTCTGTCAATGTTTCGTTGATTATCCATTATGAAAAAAATGCCTAATGGTATTTTCTTGATTATTGCTTTCTGCTTTTTGCATCCAGCATTCCTCTCGTACACTGCCTCAGCAATTGCCGTGACATTGTTCTTGGTTTTGTATTGTGCTCCCCGTCATGGCCAGACTAATATTTTGGTTTATACTGGAATATGCTCGATAGTTGGGTCCTTGACAGTAAGTGACTCTACCCAGTTAACCTTGTCATATGTGATATTTTTATCATTTTGGATATTTCTGTACTTTCTAGCTGTTGACTGAGTTGTATACCATAGTCCACCATTTGTTTTATCTTTCTGATAAAATACTAATTTCTCAGGTCATGAGTGTAAAAGCAGTTGGCATTGCGATAAAACTTACACTGGAAGGTGCAAACCAGGCTTTCCACTTTCAGGCATGGGTTTTTGCAATGGTTTCTGTCACCTGCATCATTGTCCAACTAAATTACCTTAATATGGTCAGTACTATTTTTTTTTTCTGTGCATAATTTTTTGCATTACACATTAACTAGCTGTTAAAACCAGGCGTGCTTTCTTCAGATACTTTGTTGCATTCTGTTGCTTTGTTCTCTGTGTTTTACATGTATAACATGTCCTACTGAAGACAAGTTAGGCTGTTTGTGTTTGTTGAAACCTGTGATTGATGAAAACTATGTGAACTTTGTTTTCTTGTTTAATATCCTCTTAATTGTCTTCATTTTGGAGGATACACTCATTCATGTTTGCTGACCATTTATAAAATCTTGATTTTAAAATTATTTATTTTAATGATTTTCTTTGCCTTGATGCATTTGGCTACATATTTTATATGGTCTAATTGTCTAACCAAGAATTTGATGAATTCTAGGACAAGGTTTAAAATTGAGCTTTAGTTTTCATCTCCTATGCTATTGTTTGCAATCATACCTTATATTTTTTTATACAATTTTCTCTAGTAAGTAATTGATTGATTGATTGATTGAAAGAGACAGGTGCATTTTCCATAATGCAATCAATGAATCTGTTCATCCCACCACTTAGTTGATCTGTCTAGACTTATAACTTTAACACCAGTTCATTTATGAATTGCATGATTCAAAGCCACAATTTTATCAGTGATAAATAATGACGCCAGTTCATTTATGTATTGCATGGTTCAAAGCCATTTATGATTTATCACATGATATGAATGGCTTGTGGAGTTGTATGGAATCATTCAATTCTAACAACTATGGTTCAGGTAATTTTCAATTTGGAGCAGAACTCTTTTTCGAGTAACTAAGGTTGAGATTAACTTTGAATAATAAATACTGTGGTTAGGAGTGTATATGCCCAAAACACTACTTATTTTTTAACTTGTCTGTTGAGTATATGAATAAAATTTTGACGTCCAGAATCATTTGATTTTGAAATAAGATTGTTTGTCCTTCCTTTAGAAGAAAAGATCTAGCTCGTGTTGGGTTCCTGTTATATGTCTCATCTGATTATATGTAGTATGGGGAATTATTTTAGTTTGGAGACTTTGATCATACTTTTCTTGGCTTCTTAACTTTTTCTTTGATCACTAACTATAATCATGCTTGTATTGTGTTTCCTTGCAATTCTTCTGACTCCATGTGCCCATTGATTGTTCCTTTCTAAATTAAATTGCATATCCAATTTTTTAGAAACTCTTTGAAGGTTGTGAAATAATTTAGAAGCCAGAGATGTAAATCCCTTGTCCAAATAATATCTATGTGATGCTTGAGCTGAAGCCTACCACCTGCATTTTGATTTACTGGAGTGTTTCTGTTCCTTTTAGTTGTCATAATTTTCTTGTTATTTAGTCTTCAATCCTGGCTTCTATGGTGACCAACTGTTCCATGGTTGCAGGCATTGGATAATTTTAACACAGCAGTTGTTTCTCCAATCTATTATGCATTGTTCACTTCTTTTACAATATTGGCCAGCGCAATCATGTTTAAGGTTTATACTTTTCAGAAACATAATGAGAAAATTTGTGTGTGGAAGGAGACATTTTATGGTTCAAGTATTTTTTGTAATCCTGCCTCCTCTGCTTTTCTTATTTAGGACTATTCTGGTCAAAGTATAAGCAGTATTGCATCAGAGCTATGTGGTTTCATCACTATTTTATCTGGAACGACTATATTGCACAGTACAAGAGAGCCAGATCCTCCAGTCGTTGCAGGTAATACCTCTTGCACTTTAGTGTGGAGGACTTGGATGATAGAAGCTCATCATTATTCCAATCTTATGTTTACGTGGATAAGGGCTTTTCTTATGTTGTTCAATATTCTAATCAGTATATTGTACATTTGTCCTAATCCTGAATTCAATATCTGGATAGGGGTCGCATCAGATAGTGTAGGATTTCATAGCATAATAGGATATGTTTACTCTTGGCACTGAGATTGGAAATTATTTGCTACTTTCTAGTAATCTTAAGTTGAGATAACTAATGCTGTTTCCCAAAAAGTACATACACTATCTTATTGTTCTTCACATTCAGATTTATATACACCATTGTCTCCAAAAGTGTCATGGTATATCCAAGGCAACAGCGAACCCTGGAAACAGGAGGAGGATGTGTCACCCTTGAATTTAATTGCGATTATACGGCAAGACCATTTCAAGTGACAAAGGAGGCTAAACTGCATGAGGTAGATCTATCATGTGCTATCCTTCCATTAAGCTAGATATAGCACCTGCATTTTTCATATTCCTTAAGCTTGTTTATATTAAGCAACACTGTGCAGGCAGAGAGGAGACAGTGCTTCCTTTTTTGGGGGGAAATCTAGGTTTTTGTCTACTGGTAGCACTTGAAACGAAGGCTTGAGTGCTATCACCTAAGAATTAAAATGGTCCTTTGACCATGTTCATGTTTCTAAGGAGTGAGTTATAGTCTAGCTTGCTTCCTTTTTCTCAACTCTCTTGTATTTCTTCAATGTATTTCCCTATTGGTTCTTCTCACACCACCCTGTCTTTTTTATTTCTTATTATTTTTTTTGTTAAATGAAGAATGTTTCAGTGTTCTAGTGTTTTGCAGGCTGTGGAAGCTAATAATGTATATCGATCCGTGTAGTTTCAACTTTCAATTATTTAATTAATTAGTACTTGAAAATTATTGGTAGGATCTAT

>GLYMA.06G005000

GTAGTTTAATTAGCTCTCAACTTCAAAATTACAAGCTTAAGAGATTTGAATTCCTCGACTACTGGATTGGATTAGATATAAATAGTGTTGATGATAGAATACAAAAAGGTAATTATTTGAAAATATTGAAATTTGTGAATTGTAGCGTATGTAAGAGAGAAATGAGGTGACAGAGGCAGCATGAATGAATTGTCGCGAAGCAGAGTATGAGTATGAGAGGAAGGTTCCGCGGAGGAAATGAGAATGATGAAAAAGTGGTGAAGTGTGTAGAGAGCGTTAGAGTGATAGTGATGGGGATTGCAGAGAACTCAAAGGGTTTGGTTCTTGCTGTGGCATCGGGTGTGTTCATAGGAGCAAGCTTCGTCCTCAAAAAGAAAGGTCTTAAGCAGGCTGCTACCCACGGCACTCGCGCAGGAGTTGGCGGCTATTCTTATCTACTCCAGCCACTTTGGTGGGCTGGCATGCTTACAAGTAACCAACTTAACTCATTTTCTTCCCAATTTTTACCTTCTTTAAACGAACTCAATATTCTCGCAGTGCTTATTGGTGAGGTTGCAAATTTTGTCGCTTATATATATGCTCCTGCACTTCTCGTTACTCCCCTTGGTGCACTAAGTATTATTGTCAGGTATTATATTAACAACACTTCTCATCAATCTTAGTTTGACTTTCTTTTCAAACTCACTCAATCCCCATCTTTATCTATCGGGATATATAGTGCTGTTTTGGCTCACTTCTTGCTCAAGGAAAAGCTTCAGAAGATGGGCATTTTGGGATGCGTCTTCTGCATTGTGGGTTCAGTTCTCATTGTTATTCATGCACCTCAAGAGCATGCTTTAAATTCTGTCCAAGAAATATGGGATCTCGCCACTCAACCATGTTTGTTTTTGCCTTGTTTTTATTAACTTCATTTCCGGTCTATGATATGAAGTCGCGTTTCTCATCATCTCTTTTTTTCATTTCTACAGTATTTCTAGTTTATGTGGCGGCAGCAGTTTCGGTAGTTTTAGCCTTGGTTTTGCATTTTGAACCTCGCTACGGACAGACAAATATGCTGGTTTACTTGGGAATTTGCTCATTAATTGGCTCACTTTTGGTACTTTTTGTTTTCGCCAATCACTTTGATGGAAGCTTCATATTTATATTCTACTTCACAAACTAACTAAACTGTTCAGGTTATGAGCACAAAAGCCATAGGGATCGCAATCAAGCTTACATTAGAGGGAACAAGTCAATTAACATATCCTCAAACTTGGTTTTTTCTTACCGTCACTGTCATATGTATCATTACACAGTTGAATTACCTGAACAAGGTGAGTGCATAGTTATCAAATTTAGCCTCATGCAAAACATAAATTATTCTTCATCAATGCATTGAAGGTCACCTTTTCCTATATTTGTTTAAGCACTGCACAAATTGATGTCTGCTTTCTTTGTTCAGTACTGGTTGAGGTAGAAGCTTGTGTTGATTGACTGACTATCCATGTACATTGTGCTGATGTGCATGCTATATATGATCTGCAGATGCATCATCATCTTTCCAAAACCTTTTCTGAAGAGATTTTGGTAGTGCAATTTAAGGAGTTATTATAGCCAAAGTATGCAGGGCAATAGAATCATGTCATGGCAATTACAATTGTAACTGGATCCTGGATCCTTATATACTAGACACAATTGAATAGGAAATTGCATGTTCATGCCTTCGTTTTAAAACAAACCATCCCCATGGGATACATCAATATCTCAAGAACAAGGAATGAAGCATGTTAGTATGCTGATTTGAAAAATAGCTAACCTTGAATAATTTTAAATTAGTCATGAAAAATATGCTCCTAGATCCTAGGAGATCTTCTATTGCCCAAACCAAAGTTCCATTAGCACACTAGCTAATTGCACTCCTGCGTAAACTCTTGAGTTATAACTTCTTTACTCATAAGTATTAATAATAACTGTCTACATCTATATCAAAGGCCTTAGCCCTTGAAACTTGGCTTCTAGAGAGAATTAAAACCATGCACTTAATTATAATGGTCAAGACTGTAAAGCTCTGGCTTGCCAGTCAGAAACCTTTCCCCTCTAAACCTTAGATAGATAACTATAAGAAGCTATGCAAGATTGTTGGTGACTGGTAATAGTGCATGTCAGTTCCCAATGCCGTTCTTGTTGAAATTTCAGAGAATAAGGTTGTTGCGAGCTCCAAATTTTCATAGTAGTTCTAAAGCATTTTCTATCAAGTATATTATCAATTGGGCTACTTGACTAGTTAATTACATTTAACCTATGGTTTAAAATTATTGAGAGTGCAAAATGACACGTCGTGGTTGATAAGAAACTATAAATGTAGTTGGCATCAGATCAATCTTTATGAATTTTCATATTTTTGTGCATGAAAAAATTGAGTTCTTGCAATTGATACAAACTTAAATATACAACCCGTTCAAATTAATCCTTAGAAGTATGAAGCGAAATCTTTTAAATGGTGCTCTGCTAGTCTTTCAGCATGTGTCTTTGATCTAAAGTTTTATTATTTCAATACCAAGTTACAAACAGGTTAACAGAATTCTACTGTTGTTTTTACTGCCCTAATATGACTTTGCCTATCAGCCATCTAAGGTCTGTCTTAGTTTCCTTCATCTTGTAACTAATCTTGAACATTTTTGCACTATGGAACTATGTTTCAGGCACTGGATACATTCAACACAGCAATTGTCTCTCCCGTATATTATGTCATGTTCACAACTCTGACCATTATTGCTAGCGTGATAATGTTTAAGGTATCATGACATATTCATATTCTTGAATTCATTTGGTGCAATATCGAAGGTCTTGTTTTTTTGAAAGCTTGACTTTTTGGTTGCTTTAATTTTCTGTGCAGGATTGGTCTGATCAGAGTGCAGGCAGCATAGCCTCTGAGATATGTGGATTTGTAATTGTTCTTTCTGGAACAATCTTATTGCATGCGACAAGAGAACAAGAACAATCCAACAAGCAAGGTAATTAATACATGTTACTATCAATCAATTGCGCATGTCTAATTACCAATTATTTGTTTGAAATGTGTCCAAGGCGAAATCCCTTGAATTATGGATTCGTGATTGTAGCTTCTATTTACTACCTAATGACTACCGAATGCCCCTCTCCCTTTATTTCCTGTAATAAAATTATCAATTGTGCATAGACTCTCCTGTGGCAGACAGTGATATTCTATTTGATGCAGGGTCCTTAACATGGTACATTGGTGAGGACTTGGTAAAGAGCATCGAAGATGGACACCTGAACCTGTTACATGGTTCAGATTATGTTGAAAAGTGACTTTTCCCGTGGTTGACGTAAGAGAACCGGTTCTCCTAGTACAGTGGCATGAACCTAGTGGATTCATGATTTGAAACATTGCAGTGTGGATTGCTTGTGATGCTATAGTTTTTCTGACTATTTGCAGTCTATTGTATACAACTACCATAGGAACCATCTTATACAATGTGTGATTCGCTAATCACGATTCAAGCAATCTTTTTCAAACTGAAATGGTGATTGCTACCACATTGCAACACTGGATAATCATTAGAGTGATGGTATGCTTTATCATCCTTGATAGCCAGTGCAATGTGTTTCTTTTCTGCTTTTTTTGTAGGACGTTAGCTCGTACTTTTCATTCAGTTACCTGATTCTATTGATTCTTTTATTGATCAGGTATTAATATGTTGATTTCGGTTAAAGAATATTTAA

>GLYMA.06G053100

TTTTTCTTTATCAGGTCTCTGAAACCCGTTTAAAGAATGATAGTATTGAATAAATAATTAATCACAATTAACAAATCCTAGACACAAAACAAAAAGTGGGGCGGGCACGCAGAGCAGAGGGAGGAAGAAAGGGTCGAAGAGAATGTGGGAATCAATTGTTTTGACGGTGGTTGCCACTGCCGGCAACAACATCGGCAAAATCCTTCAGAAGAAGGGCACTGTCATTCTTCCCCCTCTCTCTTTCAAGCTCAAGGCATGTTTCTCTTTTCTTAGTTCCGCTTTTTTTTTTTTTTTTTTTTCTTTTCTTTTCCATTTTCATATGCCCTTGATTTGGTTCCCGTTTACCATTTCTGCGGTTGTATATTTTGATATTTTTTGGAACGGTGAAATTGGGTACATGCGCGTGCATATGTATTAACGTGGGATTTTGGGTGGAGGAAAATTGGGTAAAGATCCAATTTTTCGTTGAAGCATCAGTAATTCACTGTTTGGTTAAGGATTAACATTGCTATTTGTTTTTTTACAATTGTGTTTGCAGGTCATAAGGGCATATGCTTTGAACAAAACCTGGCTCATAGGTTTTGTAATGGATATATTTGGGGCACTGTTGATGTTAAGGGCATTAGCTCTTGCTCCTGTAAGTGCTCTAGCTATATCTATAATAGAAAAATAATGCAGAAAAAACTATAGTTTGAATTTGAAGGGATATTTCTTTATCATTAGCAGGATCTTATAGTCTTTTGCCAAGATTTTTGTCATGTGTAATTTGGTTGTTTTTCTGATTTAGGTCTCCGTCATCCAACCAGTTTCTGGCTGTGGATTGGCAATTCTCTCAGTCTTTTCTCATTTTTATCTCAAGGAAGTTATGAATATTGTTGATTGGGTGGGCATTACCTTGGCAGGTTTTGGCACAATAGGTAATGCAAGTTTTAGATTGCCAAAGTAGAGTGTTCAGTTTAATGCTTGTATTTCCTTTAACACAGCAAGGAACATGGCTGTTGAAGTAATTATAAAATGTCTATATTCTGTTTCTATTGGTCTGTCCCTTTCTTTGTGTTGTGTTATCATGTGACACTAAGTCACTGACAGAAAAATCTTGTGCATTTATTAACAATTCTTTTTGTTGATTATGATATACTAACAGAATTTCTAGGTTTTTATATAGGTCAGAATTTGAATCGAAACCTAAAAGTTATGGTGTGACCATTTTATTTTTCTTTATGGTGCAGTATGCACTTGTTTTTTGTAGCAATGGATAGAGATTTCATGGAATTTTCCTTTTTTTAATCTAGCAGCTTATAAAATTAACTTTTGGAATCAGTTACCAACATCATGTATCAATCTGTTGAAATATGATTTATGTTCATTTCTCCAATTTGTTAACTATTGTATGCTCTTTTAGGAGTTGGTGCTGGAGGTGAGGAGCAAGAGGCTGCTGCTCTATCTATATTTCACATACCGTGGCTGGCATTTGTTGTTTTCATCTTGTTTGTAGGTACCCTGTCTTATGTTTTGGTCCATCAATTACCAATTTTATTTGAAGTCAATATGAAGACCTTACTGTGGTCTCTTGGAACTAATTATGCAGAGTTATGATTTCTTTTTTTGCAAACCTGATTGCATGATTACAAGACTCATAGTTTTGATACATTATTTTATTAATAGTTATAGTATTTTTATCGGATTAACTTTTCTTTAAAGAGTGTGGAGTCATTGATGCAACAAAGTAACATTACTATCTTATAAAATAGTTATTGTTCCTAATTGTTAGAAACAATTGTACAGATAATGCTTAATGGATGGCTTCGCATATTCAAACGCAATCGAAGAGAACAAGAGATGGTAATTCTTGCTTTAATCATGATAAGAAGCCTTTCTTACATGTTTATACCACTTATGGTAATTCTTTGGTTGCTTGACATTTTTAAGTGATGCAGATGGAATATGATGTTGTTGAGGAAATTATTTACGGCTTAGAATCTGGAATTTTGTTTGGGTACTGTTACTTCCCTACTATATTTATATATTGTGGTCTCGGATGGACACATTTCACTTCCTTCATTTGCCTCAACTCTTTAATATTGTCATGAAGATTATGACAAATAAGTGTTTTACCCTTAATGATATTACCTGTACTTGTCAATGTTTTTTTTTCTTTTAAATAAATAATGTTTTGGAGAGAATAATGCAGCTAGAAGGATAACAAGTCATGTAGAATAGAAAAGTAAAAGGAAAAAAATGCTACAAAACTGTCCTTTCTATCTGTATAGTGTATACAAATCAAGAACTGGAAGTTTCTTCAAAATAATTATCAGCAGATTACCATAGTCAGAGAGATCATAGATACAACTCTATTCCATACATATGTAAACAGATGACTCAATGTGATCATTTCTTTCCATTTATAACTAACATGATCTGTTTTCTTGTTAATTATCAGCTTCTTAAAAATAGTCAATTTTATATAACTGCTTACAAAAGTTTATTTGTGAAGCCTAACCCTTAGTTTTTAGATTTGTCAGTTTTTATTGTGTGTCATTACTTTCTGTTGGCTATGAACAAAACTTGCAGATAATTAGATTATAAGATACACCATCCGGTCTTTTATATAAGAAACAAATAATTAATTCATCAAGACCAATAAAAATAAGTTAGTTTATCTTAATGAATAATGTTAGAATTTAAATTTCATTCCAAAAATACTCATAATTAAATTATTTTAGTAGTGGTAATATTTAATGATGCTACACATATTTCAAGGGATAATATTTTTTTCACCAATGAGTGTATCTTATATGGTTAACAGGTCAATAAATGCATCCACTTGCATGGAGAATGAGTGACCATCTCGTTAGTTGCTTTCATAATAAATTAGGGTATAAAAGAGAATTAACAATTAACACATCTAAAAGTAGGTCTATGTACTCATTTGTTTTTTATATAAAGGACCCGAAAGAGTATTATTTACACACAGAGAACGCAAGTTGATCATAAGTCCTTACTCTTAATTGTGTTATTATGAAAATGCAGGATGGCATCTGTAATATCAAAGATGGGGTTTCTGTTCTTGGAGCAAGGCTTCCCCAAGCTGTTGGTTCCTATTTGCATCATCATAAGTGTCTGTTCAAGTGGTACAGGCTTTTACTACCAGGTATTCTGAAGGCGTCTTTCATGCCATTTACTTTTGTGTGAACTGTTACATACTTTAAGCCTAAGGGGCATTTCATTGTATGGATGGTGGGAAGGTGGGATTTCAGCCAAACTGGCTTGATATTTGTCCAAGTTCTGCTTTGTGAATTTAAGATACACTAACATCTTCTGTATTTACTTCCCCAACATGCAGACACGTGGTCTAAAACATGGGCGGGCTATTGTAGTTTCTACATGTGCCGCAGTGGCATCAATTTTGACTGGTGTACTTGCTGGGATGCTTGCTTTGGGTGAGCGACTCCCTTCGGCCCCAAAAGCTCGCTTGTTACTTCTTCTTGGATGGTAATAATAAATGTTATGCTATTATTTCTTTGTGCATTTACATCCCTTGCTTTATAAAGCTGATCTACGCAGAAATGAACTCCACTCTTCTATACTTCATTACATCTGCAAATAGTTACATTTCATTTTCGAGCATTTTCTGTTCTGCTTCTAATCTTTGATGCAACTCTTTTTCTGATTTGGTTGAGTATATATATATATATATATATCACCAATGTTATGGTAATTCTCGAACAAGATAGACACCTTCATGTTCTTGTGTCAGGAATATGTTAATCGGAAATATTTTGCTGCAGGCTACTTATAATTGTTGGCGTGATTTTACTTGTTGGTTCTACAAAGCTAGTGAGATTCTTTCGATTTTCTTCACACCGCTTTAAGAATTATGGCCCTAGAAGATCTGGAACTTCCCGTGTTAGGGAACCTAGCCCAACTGCTGTCATTCAAGCAGCGACCTTAAATCATTTACTCTCATCATCTTCCAAAGAAAAAGCTTGATTTGACTTGAGCTGGAAAATGATCCTTCATCCATTCTCAGGAATCACCCTCACCCCTTAAAGAAGAAGACCCCTTTTGTAAGGTTAGGTTGTTTGTGTACAGTGGTTGCTTTATCAGAGGGCATGAATTTTTAGGGATCATTCCTTCTGGCTCACGTATTTTACGTGAAAAATCATCACCTTTTCTGATGCCACTTGTATAATTCATGAACTTCCAATATTTCTCAATATTGTATATGAAGATGGTATAGCCAGATGCAGAATGCTAGCATATCATGGAAACAAATTGCAGGGTGCAGTGGAGAACGATTTATTCCTAATTTCCTATGCTAATGTAGTAGCAGTGTTCTTTGAGATTAGGTTTGACTTCCGTTACCGGGTAATAGGACAAGGCTGTACTTTATCCTCTGGAAACTATCATCGTGGCAAAAGTGCTGATTCCATCAAAATTTAGGCCACAAACCCTCCGTTGAAGGGAATTATAATGATGATTTGATCCTGATTGTTAGATGAGGGAGAGTTTACTATTTGACATTTCTAAAGTGAGAGGGTATATTTATTTCCTTTTAATTTAAAGTTAAGATTGTGAGGTATTTTAATCAAAATCAAGAGGTAGATATATTCTTACCTTTTCACTTTACACATTCTTT

>GLYMA.06G159100

TTCTCTCTATGTCACTCTCCCTCCAACTCCAAATGCGACCAAAACCAAAACCACCCGTCAATTCCAACTCATTGACCAAACCAAACAGTAAAATAACTAATAAGTAACAACACAAAGCGCCCCGCCAGAAAAACAAAACTTGACTTTGGAGTTCATTCATTCATGCAATCCAAATTCTAATCTAGGGTTCCAAATCACTCTCCGTTGCGACCATGTACTCCACCAATTTGATCGGTTTCATTCTGGCCGTCGTCTCCAGCGCCTTCATCGGCTCCAGCTTCATCATCAAGAAAAAAGGCCTCCAACGTGCCAGTCTCAACGGCTCACGTGCCAGTTACCTCTCTCTTCTCCCTCACTCTCACGTGCCGTTTTCCTCCTCTTCCTAACAATTCTTCGTTTCAGGTGGCGGAGGCTACGGTTACTTGCTGCAACCTCTTTGGTGGCTCGGAATGGTTACCAGTTAGTACAGTACCTAACTACTTTTATTCACACTTAATTGCCTTTTTAGTCCTTAATTCTCGTAATTGTGCGATTTTACTTTCTCGGGTTCAATTATGCAATTGAGTTCCTCAATTTTTTTATTTTTTTTTTACCATTTACAGTCCATTCATCGTGTTGCTTCATTCAATATCTAACGAAATTAGCTAACTTAAGAGACCGATTGTAAACAAGAGAAAACTCGAGTGACTCGAAGACAGTAGTTTCAGGTGTTTACAATAACTCACGAGTTAGACTCCCTCACACTTGATTTACGCTATTAGAATACCGGGACCAAAATCATGATGAACTAAAAATATAATTAAGTATTTTCTTATTTATTTCACTTTCTTATTGCTTCTGAGTTGGAGCCTCTTTTTTTGTTACAGTGATTGTCGGAGAGATAGCGAATTTCGTGGCGTACGTTTATGCCCCCGCGGTGCTTGTCACGCCGCTTGGTGCTTTGAGTATTATTGTTAGGTAATTGCCAATTTTTTATTCTTTTTTTTTAATATTATTTTTCTACTAAATTAACTGTTTTGTGAATCTGGAAATGGCTAGTGCTGTGTTGGCGCATTTCATGTTGAACGAGAAGCTGCAGAAAATGGGCATGCTGGGGTGTCTTCTGTGCATTGTGGGGTCCACTGTGATTGTGCTCCATGCACCTCAAGAGAAGCCTCTTAGTTCTGTAGAAGAAATTTGGCAGTTAGCACTTCAACCGGGTATGCTGTTGCTTGAGGTTTTATTGTTTGTATCTGCAATCAAAATGCGAATTTTGTTTTGTTATGCACGCCTGATGTTTCTGCTTCCTGTTTTGGCATCCAGCATTCTTGTTGTACACTGCCTCGACCATCGCTGTAGCTTTCTTTTTGATATTGTATTGTGCTCCTCGCTTTGGCCAGACTAATATTTTAGTTTATATTGGAATATGCTCCATAATTGGATCCTTGACTGTAAGTGGCTCCTCTTTCTACCCAATTCATCTTTGGAGTACTTATAGTTGACTAAACTCAATACCAGAGGTCATCTTTTGTTTTGCACTTCTGATAGAATATCTTGTTTCCCAGGTCATGAGCATAAAAGCCATTGGCATTGCTATAAGACTTACAATTGAGGGTGCCGATCAGTTTGTTCAGTTTCAGACATGGATTTTTACGATGGTTGCTATTTCCTGCATCATTACGCAGTTAAATTATCTTAATATGGTCTGTATCACTCTTAACTTTTATGTGAGCATAATGTTTTGTATTTGAAAAAAAAGAAATTAGAGCCTGGGCTTATCTTTTCTTTAGAAGAAATGATATACATATTCTGTGTTGGCTTTCTACTCTGTATGTCATCCTGCTTGTTTTTCGTGTGAGGAAGCATTTTAGTTTGCAGAAGAAGGGGATCATACTTTTGAAGGCTTTGGGCCCTGCTTGTTATCAACATGTCTGTTTTGATGTTTTCACCTAAAGTAATTGCATATCATTATATCAATCAATAAAACATTTTGTAGGCTATGAGCTGATCTAGAAGTTCAAGATGAGCATCTACTTCAAATAACATTCATGTAATGCATGATCTGGGCCACCAGAACTGATAATCTTAAAGTCCTGACTCCTATTTACATGATTGTTGCTTCCTTGCCTTTCAGTTGTCATCATTTGCTTGTTAGTTGATCTTCAAACTGGGCTTCTGTGCTGACCAACTCTTGCATGACTGCAGGCATTGGATACTTTTAACACAGCAGTTGTTTCTCCGATCTACTATGCCTTGTTCACATCTTTTACAATATTAGCTAGTGCAATCATGTTTAAGGTTGGTATTTTACTGAAACAAAATGAAAAATGCATCTAAAGAGACATCTTAACGATCAAGTGCTCAACTATCTGTTGTAATCCTGCCTCCTCTGCTTTTATTATTTAGGACTATTCTGGTCAAAGTATAAGCAGTATTGCATCAGAGTTATGTGGTTTCATTACTGTTTTATCTGGTACAACTGTATTACACAGTACAAGAGAGCCAGATCCTCCAGTCAATACAGGTATTGAATCATCCTCTTTGTTGAGGAGCCCTTTGTTGATGTAAGGTCCTCATTATATGTGCCTTTGTATTGAGCTCCAATCAAAGACTTTATCAGTATTGGGTAGCTTTACCTGTAATTAGTTTGTTTACACCAAAGGTCCTCATATTTTCTTGTCGACTTCTTGTTCAATCTAACATGCATGAGTAGCCATGGCCACAGAATAGATTTCTTTTTTCATATAAGCTATTAGAGTAACAAGCACGGTTGTCAAAATTGAGGTCTTATTTTGGATTGGCAAGAGAGGTGAAACACATACATTGTGAGACTGTAACAAGGATTGTAAGATCTTACCTAAAATACAAAATTGTGTGTGTAACGAAAACAATTAAACTTAAATACAGACTTTTTTTATAAAAAAAAAATGCCTGCAAAAAACACCTGGAGATTCAAGGAAAGTTAAAGTAGAAAATGTTTGGGTTGAGGGCAGATAGAAAACATTGACAGACTAAGCTTTCGTAACATAATTTGGATATGTTTTGACAGTGAGATTGGAATTTATTTGCTATTTTCTAGCTATGTTAAGTTGAGAAGCTAATGCTCTTTTCAGCACTGCATATTTTATTATTTTCCCCCTCCCTTCAGAACAGATTGGGAAATACTGCCTAATTATTCTACACGTGCAGATTTGTATAGTCCCTTGTCTCCAAAAGTATCGTGGTATATCCAAGGCAATGGCGAACCTTGGAAACAGAAAGAAGAAGATGGGCCACCCTTTAATTTAATTACAGTTATCCGGCAAGACCATTTCAAGTGATACAGAATTCTACTGGTGGAGAAAGAAGTTAGATCTGTCCGTTGCTTGATGTAGCAGGGTTATGTACATTAAGCAACAAAATGTGGCAGTTATACGGTTTGGCTCTGACCCCATCTATTTGATTTAGCTAGATTTAAAAGACTATATCATCACATTGTGCAGACAGAGACGAGATATTGCTTTATTTTTTGGGGGGTTTTGTTCATTGGTATCATTTGAAATGAAAGCTTGATTGCTATCATCTAAAATGCCAGGGGTGATGTATCTTCGAGTTTGCATCATCATCATCTAGTTTTGCATTATTTTCCCCCTCTTGTATTCCTTTCCCCAATTGTATTTTTTGTGGTCTATCCCCCCTTCCCCCTTCCTTTGATTTATCTCTGAACATTGAGAAATCTAATCTATGTATATTGATCGATGTAACTTTAGTTTAATAGAAGGATGTGGATATGTATATGTATGGATATCTATAATATTCTACTTCTACAACTGGATTCATCTTTGCTTGGAGACCATTAAGGTTCTGAAGCACAACAAAACTACTCTGGCTTAAATTGATTGG

>GLYMA.06G208700

ATGGGTAAAAAGTAATTTATTAAGAGATTTTGAGTTCATTTCTTTAAACTTTTAAAACGGTTTTAAAATATATATTATCTATTGTATCCTTTATATATATATATATATATATATATATATATATATATATTATATGATAAATAAATATTTTTATATAACTAAAATTTATTATGAATGAATTTTTTAATATATATATACTATATTTTTAATTTACCATAAAAATAAATGCCATTTTTTATTATTGAAATTTCTTTTAAAAATAAAGATGAAAATAGTAAATTGAAAGAGATAAATAAATAGTTAAAAAAAGTATAAAAAGGAAAAGTGGTTTGAAATAATCAATATATAAATCTTTTGGACTAATAATTAAACTAAATAAAATTTGAAATTAAAATTAAGAAGTATATTTAAAAGAAGCAAATATTAAAAAAATAAAATTAAATGAATATATCCAATATAATATATAGATGAAATTCTGTCTTATCTTATACTCCAGACAAGTTACAATAATCTCAAACCACAACATCTATCAGAATTTGTTTCTACTTGACAACATGATTATGCTTCTTTCCATTTTTGCCACGACACAAGAACATAATTTAACGACTAACCAAGAACATAATCTTTCTACTGCAGCATTTCTCTTTTATGCAGCTTTGGTAATAACAGCTATTTTTATCCTTATCTTCCACTTCATTCCTCTCTATGGCCAGACACACATAATGGTTTATATCGGTGTTTGTTCCCTTGTAGGTTCTATAACGGTATGTCTCATTATGTAGTCATTGATGACTTTCTAATAGTATTTGTCTCCCCTTGTTATCCCCCCCCCCCTGTATTTGTATTGTAAGGTTTTCCTTTGACAGGTTATGAGTGTTAAGGCTCTTGGAATTGTCATAAAGTTAACACTGTCTGGGATGAATCAGCTAATTTACCCTCAAACTTGGGCATTCACTCTAGTTGTAATTGTTTGTGTTCTTACCCAAATGAATTATTTAAATAAGGTACAATAGTTTTTCTTGTTAGTTCCCTGAACTATTTCTTGTTAGACTCTCGAGTTTGACAAACTTGAGTTTAGGTATCTGGATGGTTTATTATGAGAACAAGTTCATTTTCTCTTTGGCTTTAAACTTGGATATGCTGGATTAGGATAAAACTATATGTTTCGTTGGTGTTGGATGTCCATTTGTCATGATTCTGCTACTTACTAGTATTATGCAATTACTTTTACATATTGTCACCATTTTTTTTTGGGGGGGGGGGGGGGGGGGAGTTGATGAATAGTTTTTTCTGCTTTATAATTCACGTGATAATATTTTTTTCATTTCTGCAATCAGGCACTGGATACTTTTAATACGGCAGTGGTATCTCCCATATATTATGTTATGTTCACAACATTTACCATTGTGGCAAGTGTTATTATGTTTAAGGTAAGTTTTGTTACCTAA

>GLYMA.08G126600

AAATATGAAGTATGCGAGAGAGAGGAGTGTCTTGTTCTCGTTTATGGAGGATCAAAGCGGAGAAGCTGAAGGAGAAGAACAACAAATACAACCTTCACTCTTTCATTATTTAATAACAATTATTATTCTCAATGGGCAAGACTCCTTTCTCCTTTCGTCGCTCCGCCTCTCGCCGCCGCCCCAAGAAGACCGCCGCCCCGCCCCCTCCTCCCTCTCCGCCGCAACATCCCTACGCGGCCGGATTCGCCGCCTCACCCGACGACAACAACCGTCTCATCGCCGCCGCCGCCGGCAGCAGCGCCTTGACGAAGGCGAAGAAGAAGACCGGCAACGTCCGCTTGTGGATGAGGTTTGACCGGTCCGGCCGGTCAGAACTGGTGGAGTTGGAGAAGAACGCCATCGTCCGCCACGCAGCGATTCCCGCTAGAGACTTGCGAATTTTGGGCCCCGTCTTCTCCCACTCCTCCAACATCCTCGGTATTCCAATCCCCCCCCCCCCCCCCCCTCCGTTTTATCCTTTTTCAATTTCATCGTGTTCTCAAAATTAGGGTTTCTCTAAATGAATTGAATCACGTGTTTTAAATGTTCACCATCAGTATTATGATTATGGTGAAGAATAGAATTATATGTGTCTTTTTAATTATTATTTTTTGCAGCTAGAGAGAAAGCAATGGTGGTTAATTTGGAGTTTATAAAGGCAATTGTGACTGCTGAAGAAGTGTTATTGCTTGATCCTCTTCGGCAGGAGGTTCTTCCCTTTGTTGAGCAACTCAGGCAACAGCTTCCTGGCAAAAGTCAACCTAAACTTCTCGGCGGCACGGAAGAGCAGGAAGGCGAAATGCATGTCTCTAATGGAAGACAATGGTTGCCTACACCGGAGGCAGCCGATGGTTTGCAGTCTGAGCTTCCGTTTGAGTTTCAAGTTCTGGAGATTGCTTTGGAGGCTGTGTGCACTTATCTGGACTCGAATGTGGCGGACCTTGAGCGAGGTGCTTACCCTGTGTTGGATGAATTGGCTAGGAATGTTAGCACCAAGAATCTTGAGCATGTGCGGAGTTTAAAGAGTAATCTTACGCGGTTGCTGGCACGAGTGCAGAAGGTATGTTTTTGCACAAGTACCGTGTGGTTCTTCCTCTGATGTGGATAGTTAATTCGTTACTTATAGATGACTGTGGAAAATTGAAATTGAGAATAGAAGGATTAACCTTTGGGAAAAGAGATGTACTAGTCTGATTTCGTTCTAACTGATGTAGTTTGTTGGACTGGTCAAGACACCAGTTAATAGCCCATGAGATGTGCAAGCTAATAAAAATGCCAACAGATTCTTTTACAGTTATTGTGCATTGAGATGGTTGAAGATTTTTCAGAGAAGTAACATGTCATTGGACTGCAATCTGCATCCAATTCTGCAGTCTTTGAATAAAGAATGAAAAGTTATTTCTTGCCTAACTCTGTAATTGTTGAGTTTCCTTTGTTACATTGTTCAATAGTCATTTATTATGCTCTTAGATTGAGGGTTTTGTTCACTATTATAGATCGAAAAATTATTGCAGGCTATTTTACCTTGTATGCGATGATGCATCCCTCATCTTTGAGATATCATACTTAAGAAGTCTATGACCCTCTTTCACAGGTGCGAGATGAAATTGAACATCTGTTAGATGACAATGAAGATATGGCACAACTATATTTGACAAGGAAGTGGTTGCAGAATCAACAATTTGAGGAGGCTCATTTGGGTGCCACAACCTCAAATAACTTTCCTAATACCTCACGTTCTGTTCGTCAACTTGGTTCTATCAGAAGTGAAAGTCTTGTGACCAGCCATTATGAGGATGATAACAATGTGGAGGACTTGGAGATGTTGCTTGATGCATATTTCATGCAGTTGGATGGAACTCGTAACAAAATATTATCAGTATGTATTCTAACACCATATTCTGGTCAAATAATGTGTCTGTCAAATTTTGAAGATTGAAACTGTCATTTTCTATTTGGATTCTATCGTCTATCTAATTTATATCACACTCTTGTGCTTTTAGATGATAAATTTCATATATTTCATTTGCATACGTGACTCAACCACATAAACTTTGTTGTGACTTGTGATACTTATGTGGTCCTTTTGTTCAATTATGGGTAACATTTTTTATTTAACAACTAACATAACAATACAAGAACTGCAATTCTCTGCGTAGGGATGTTGGTTTTCATTTCTTGATTATAACTAAATGGTAGAAAGTCTATAATTTTAATGTTGAGGGGGAAGGAAGTTAGTATATGTTTCATGGACCAAAATATTGGGCTTTGGGGAGCATTTGTGTTGTTGAAATGTCAGCTGATTTTGGTTATTGTTTCTGGAATGCCAAATATTGTGATAATTTGACTGAAATTTTGAATGCTGTTTTAATTTATTGTTCTTTAGAAGTGGTAAGGGTCTTACTTCTTTATGGTTGCTGGTTTGCAGGTTAGGGAGTATATTGACGACACTGAAGACTATGTCAACATCCAACTTGATAACCACCGAAATGAACTTATTCAGCTGCAGTTGACGTTGACTATTGCATCATTTGCTATTGCTATTGAAACTTTGATTGCTGGTGCATTTGGTATGAACATTCCTTGTAACTTATATAACATTGATGGAGTATTTTGGCCCTTCGTTTGGACCACGTCTGCGGCTTGCGTATTACTTTTCTTGCTTATTTTAGCATATGCAAGATGGAAGAAGTTGCTGGGATCATAAGGCAAGGCTGTTGTGTGACAATTGGCAATTTGAATCTCCGCAGCGAGTGATCTGAAGAACAGACACAAATGTGTCACCGGAGAAGTGGCTTAGGAAGCAACTATCTTAATTTCTGACAAAAATTGTAATAGTTGATGTATAGGATGTTCCAACATTGCTTTTAGCATCATATATTTTATAGCCGATACTATTCACAGCATCTTTTTTAATTTACAAAGTTTATGGTGTCATGTGCATGCGTGTGGGTTGGCCATTTTGTTTTTGGTCCGTGTGTGCATGGCAATCGAAGTTGCACAAGGAAAAAGGACATTGTGGGGGCTTGATTTATATGAATAGCCAACCACATCGTACAAGAGGGTTCACTTTTTAAGCAGACAAATCGACAACTATCAAGAAATTTGGGATCTTCTCAATTCTAATGAGGAAAATTTTTGTACTTTTGGTAGAGTAGTCGTTGTATATGTCTGTCTCAATACTTCTAGTCTGCATTGAATTTTTACAAAACATTACAATAATCTCTAATATTATGCATGAAATAAATTGTTAATATTTTT

>GLYMA.09G019600

ATGAGTGATACAGAAGGTCAATCACATTCCTCGGGAATAACGAAGAAACTAGATAGCCATAAAACTTATCATGGTAGAGATCCCAATAATGGAAATGACCTTTGGAAAGATGGACTTATTTGTGCTTTTGAATACATTCGAGGACAAAACAGATCGGCTAAATTGAGTTCCTCCTCATCAAAGATCACAGACGGAATGCATGGCCAACATTCAAAGATGCATCATGTCCCTTCGGATGATAAAAAGAAGCTCTCAGATCCTTCATCTGTAAATGTCTCGAGGGAGAGTTTGTTTGGTGGCTCTGATGACGACAAGGAGAGCCAGACCCCTAAGGCTGGCCAATCTAAAAAGTATGAGGGTGGTCATTGGGTACCAATTGGATGGGCAAGAATTTCAGAACTTGTCCAAGCAGTTCAGGTTGATGCTGAGTGGTCTTCTCATCAATTTGAATTTGAGTATTCTAAAGATGATTTTACTGTAGCAGATTTGGCAGCTCCCTATTGGGAGCATCCCACTGGGCCTATATGGTGGTGCCATGCTTCTGCAGGTCACCCCACTTGGCTCAGCAATGCTCAATGGCTACATCCTGCTGTTAGTTTAGCTCTGAGAGACGAAAGTAGACTTATAAGTGAGCGGATGAAACATCTTTTCTATGAGGTACATTGCGTTTTAAACTTTTGTAATTGCTAGTCTTAACTAATATTATCATTTGGCAATCTATATTTGGATCTATGTTTGCTAAAACACATTTACATGCACCACATCAAGCATACATATGAGAGTGTGTATATGCAATAAGATTTTAAATGAAAATTTTCAATTGTGTTATGTTTTTGTTGATGGCTTAAATGCTTGTAGGATTGTGGTTTCTTTGTCATATGCTCTAGGATTAGGGTTTTCTAGGATCACTTAAGTGTGGAAGGAAAACAGGTAAATTTTGATTTCTAGTTGATGGGTTTTAGTACAAACTAGAACAAAGTCCGTGTGGCAGCACAGCAGAGACCTACAAATCCCAAGTCGTTGTCTTGTTGTCGTTAGGTTTTCAGGAAAATTATAAGCTTTTAGGGACCTGTAGATAGCAGTTGTCAAGCTTGACACCTAGCACTAAGTTGGAATGACTTCTTTGCTGTGCTGCCACATGGACTTAGATTGACATCTTCATGAATGGGAAACACAGCATCAAGAGCTTGCCACCTAGCACTAGGTTGGATAATTTTAATAAGGCAAGAAACTTTTAGGGACATGATATGGGGATTTTTTGAGGAGTTTGCCAGGAAAATAGCAACAGGTAAGGCTTGTAAATAAAAAAAAACAAAAAAAGTAAATGAGAAGAGTACAAAAATAAACCTAGGATTCCTTAGGCATTATGCCTTGGGCTTGTTTGTGTTAGTGTCACACAGCCAGTAGATAGCAGTTTGTCTGAAAGAAAGCAATATAGCTAATTTAAGTAGACATCCAATGTATCTCATGCTTGCATTGATCTTAGTGCACTCTTTTTTCCAACTGCATTTTATGTAAACTTCAAGTCATGTTACTTTGGCTTGACATTTTTATTTAATTTGTCAGGTTATTCCGTAAATCTATATTTGGGGCAGCAGATGAGATTGAATTGAAGTTTATGAACAGGTTCTTAAATGTGCAATTTGTGTAAATTCAAGTCATTCCAGTTTTATAAATTGTCTTGCACTAAGTTGACAATTTTCCCTTTTATTAGGAGAAACCATGAAGATTTGAATCTTTTCATCCTAATCTTAAATCTAGAAATTAGAAAGTTATCAACACAGGTATGTTCATTCTTGCCAAGGAGAAGATATCTTTAGACAACTTCATGTAATTATTTTCACTGAGCTTTATGTAAAACAACTAACATAATACAAATATAGCATTTCATGAATGCTTACATATCTCATGTCACAATCTTCCTCCTCCTTACAGATTTAAATTTGGAACAAGAATCCAGAAGTTTGCCAGACTTGACATAACTACAATTTCTCATACATAAGTTTTTATTGCTTTTCCTTTCTAAAAGCTGGATTTAAGAGTCATTGAGTTAAGGGCTGCTACTTCACAGCTTGAGCTTTTAGTACTTTTAGTTATCAACAATGTCAATGAAGTTTAATTGTTCTTTGTGTCCTTCAAACATGTAAAGATACATTTTAATAATAATAACATTCCATAACCTATTTATTTGTCTATTTTAATATTTCACGTTTGAAAATAGCTTTGAAAAGCCCAAGCAATACCTAAGTGAGCCAAATTCTCTACCTTGGGTTGATTTTTTTTTAATGAAATTAATACTGGCCAAATCCATTACACAGAAGCTCTTCACAACTCGTGTTTCATTGCAGGTTATCAGGGTTAAGTGGTCACTCCATGCAAGAAATGAGATTGTCTTTGAGCTTCTTCAACATCTAAAAGGAAATGGAGCAAGAAACCTGTTAGAGGGAATAAAAAAGAGCACAAGAGAAATGATTGAGGAGCAAGAAGCTGTTCACGGCCGTCTGTTTACCATTCAGAACTTTCATTTAGAATGCTTTGCTTTTTTTTTTTTTACTTTCTGATAGTAGCTAGAACAACTTTATATTGCTCACCCTAGATTAACATGATAATTGTCTTGCATCCGCTGAATATCTATGCAGGACAGAAGCCTTCGAGTAACCCATAATTTAGCTGTATTTGGTGGCGTTGGCGTTGTCCTCACCATCATTACCGGATTATTTGGGATCAACGTTGATGGGATACCTGGGGCAGAACATACTCCGTATGCATTCGGTGTTTTCACGGCCATCCTTGTCTTTCTGGGAGTAGTGCTGATCGCAGTTGGCATGGTTTACCTTGGGCTGAAAAACCCTGTTGCTGAGGGACAGGTTGAAGTTAGGAAGCTTGAGCTGCAAGAATTGGTGAAGATGTTTCAGCATGAAGCAGAGACTCATGCTCAAGTCCGGAAAAATATTTCTCCCAAAAACTTACCTCCTACTGCTGGTGATGGTTTCCGCAGTGATGCAGATTATCTTGTCATACAATAG

>GLYMA.10G180200

CTTATTTCTTCACCCTCGCCCTTGCCTGTGCCTCCCCCCCTTCTAAACCTCACATAATTAATTCACATTAATTAAAGAGACACACATACGTAAATCAACACAACACAAACCCAGAATCAAACGTAGTAACTCTGTTGGGTTTTTTCCTTTCTCCGGGAAACAGATAGAGAGAAAGAAGGAAAAAGTTCCTTTTTGTTGTATCGTATGTTTGTTTCTTCTGGGAATCTTATTCCTCAGAAAAACAAAGTAAAAAAAAAAAAAAAACATATTTTGAGGCGTGAGTCGGATACCCAATTGGCGTGTTGATGAAATGCGCGAAGGGGGTGTGAGCAATTTGGCTTGTGGTATTGTGGATGTGAATCGAGTTTGAAAGTTGAAATTGTGGGTTGGGACATGTGATTGGAATTGGGGGAGAGGGAACGGGTAAGGGAATGGCTCGTGATGGGAGTGTCGTCCCTGCGGACCCGCAGGCAATGGCGGTTGTGAAGAAGAAGACGCAGTCTTCGAGGAGTTGGATTCTGTTTGACGCCACTGGGCAAGGCACGTTGCTCGACATGGACAAATATGCCATCATGCATAGGGTTCAGATTCATGCGCGTGATCTCAGAATCCTTGATCCCTTGCTCTCTTACCCCTCTACCATTCTTGGTCGTGAGAAGGCCATTGTTCTTAACTTGGAGGTCAGGTCCATCATCATCATCATATATTTACTTTTTATTGTCACAATTTGTTTTCTGGTTTTGTTTTTTGATTTTGTTTTGTTTCTTTTGATTCTGTGTTTTTCAGCATATCAAGGCAATTATCACCGCTGAAGAGGTTAGTTGATCACCTTTTTTTATCCTTCAATATGTTATGTGTGACTGCCTCCATCTCCGAAGGAGCGGGATTTGAAACATTATTATGGCTTCCTGTTTCTGGGAAAGAACATGAATAAGAACATTATGAAATTAGTATGGGGACACAAGCTTTGGATTTTTGTTTTGGGCTACTCATAATGAAAAGCTTAGACTGATTGAGAAATTAGGAAAGGAGGAATGGCAGATGGGGGTGGGGGGTTTCTCTTAGCATGTTTTTTTGGGCACTGAAAAGTTTAGGTGAGTTGGGTCTCGGGGCTCACTAGGCAAACCTCAAGCTCAAAGAGTGCTTACAAACTACCCAGCAACAAAGTCCTCACATCGGCAGTGGGAATGGAACCCATGTCCTTTTGGGATGAGTCCGTTCTCTTAGCATTTTATCTTGCTATTGCATTAAATGGCACAACTAGTCTGAGGATGGATTGAATTCCGGCTATGGAATTTTGTTTTCATTATCAAGTTTATTGAATAAAACGGGTAGGAAGACAATGAACTACATGTTGGATCAGGACCATAACCGTCTTGTACAAGAATATACCTACATGTCATTAATTTGTTTCTGACTTTAGTTATGATGGCTGGCAAAGGACATATTTTCATATAACTAGAATATTGTAGAGCCAGCTTCAATAGGCTAATTAATTAACCACGCTCTCTTACCTAGTTTGTATATTTGTGAATTCCTGTATCCAACCTTTGGTCCTTTTTAGAATGCATGATTGTTTCTAGAGATTGAGATGGCATTTGCTTCCACATCCTGAACTCCTGAATGTATCTAAATTGTGGGATAGGTATTGCTGAGAGATCCAACAGATGAAAATGTGATCCCTGTTGTTGCGGAACTGCAAAGGCGGTTGCCTCGATTAGGTGCTGGTCTTAAACAGGAAGGAGATGGTAAAGAGTATCTTGGTGGCCAAAATGATGCTGAAGCAGCTGAAGAAGACGGTACGCTACATACTTACATTTAATGGTGAACCATAAGTAATGAACAAACTCATTGATTGCATCTGTTCCTCTTATAAATCTATCTTTGTATTTGCATTATCTTTTGCTCTTGTTATCTGAGTGATCTAAATTATGCAATGTAGAGTCACCCTTTGAATTCCGGGCCCTGGAGGTTGCTTTAGAAGCCATTTGTAGTTTTCTTGCTGCACGTACATCAGAATTGGAGATGGCTGCTTATCCTGCATTAGATGAACTTACCTCCAAGGTACATATATTTTTGAATTCTTTCTCTTCTTGTTTTGGTTTTAATCTGAGATCCCCTGGACCATACCCTTTCCTCAGACACAGAATAAGCTAACAAATAAGAAGCTTCTTGAAAGTGGCTGAGGGAAAACTTCTTTCATTCATCTCAATATATTATCCTTTTGATTTTTTATACTTATCTCACTGAGGTGTTTATTCTAATTATGTCAAGAATGTTTTTGTTGGCGGGCTTGAAATGTGGATGCTCTAGTTCGATTTTGCTCTTGTTTCCTTTCTTCCTTTTGTTTCTTAACTTTTTTGGTTCAGTCATGAACTTTAAAATTATTGTAATTGTAATCTGCAGATTAGTAGTCGAAATTTGGACAGAGTTCGAAAACTGAAGAGTGCAATGACAAGGCTGACTGCTAGGGTTCAAAAGGTAAGTCAAGTTTTACTATGCTAGGGAAAAATTTGCACATGGAAAAAAACTATTTTCTTCTATGTAAATATATTCTCCACCCTCTGCCTCTCAATGCTGTTGATATTTTGGACATCCTTACACTTTGAAGGGTTATTCATTTTTTATGATGCATAAATGCATGAGAAATTCCTTATTAAAAATATGCCTTTATATAGTTTGCTAGAGTTTGAAAAATTGCAACGTGTATTTTCAAGATCAAAAGAGATTCAATTTCAAGAAAGATTCACTGTTGTGGGTCCCTGCCTTCAAAAAATGTTCTTCTGATAATGACTTATTGGCATTCTCTTTATTGTAATTATTTTATTTTTCTTCTTAATAGAATTAGTTTAATGTGACTGTAAACTGCCTTGATCAGTTTTAAGTAGAAAACAACATATATCCTTTGTCCCTCTCCCATCACAAGTTCTTGTTATCTGGTACAGGTCAGAGATGAGCTTGAACAATTGCTGGATGATGATGATGATATGGCTGACCTATACCTGTCAAGAAAGGCTGGTTCAGCATCACCAGTTAGTGGATCAGGTGCTGCTAATTGGTTTGCTGCCTCTCCTACCATAGGATCAAAGATATCTAGAGCAAGTTTAGCAACAGTTCGTTTAGAAGAAAATGATGTGGAAGAGCTTGAAATGTTACTCGAGGTTATAAACTTTATTTGCTTTAAAAATAGAAGGAAAGCATCTTCATTTTTTTAATGAGGAAAAAATGCTATAAAAGGTCTTTGTTCATTTTGCAGGCTTATTTCAGTGAAATCGACCACACATTGAACAAATTAACCACAGTAAGTATCATCTCTTTGTGTATGTGAACACATGTGTGATAGGGAGCTGTAAATCTTATTTTCCGGATATGACAGATACATGCCAGATATAAAGTATTTTGGAAGATTGTTTCTTTGTAAATATGGAGAAAAGTTTTTTCTCTCGTTAATTTAAAACCTATGGTCTGTTTACTATTCTAATTCTTAGAAAGTGAATTCAAGGCCTAGCCTTTTGGGTAGAGACTTAGTTTCAGACTAGTATGCAAGTTAAAGTTTGACCAAATTGGTACTCCGAATGCTTAACACTTGTGGTGAAATTTATGGTGAACAAAGGGGATATGCATTTTGCTCTCTTTCTTCTTCCATCACCTGCTGTTTTAAAGTTAAACTTTGTGGTTTATATTCAAAATCTTGTTTTGGAAGTCTTTTATGCAATTTTTCTGAAAAATTAGTTATAAGAATTTTATGAGATGATATACTCTTACTTTGTTGGATTCATTCTCGTCTGAGATTGAGTGATTTATGCTATTTATCCTTTTGGATCAAATAGACCATACTTGGATCTTTGCTGTACCACTTTTGCCTTTGTAATTAAATATTATGATAATGGTGATTATATGTCTAATTCATGCTGTCAAATCCCTGAATTTATTTGTGGTGGGATGTGTTAAGAGGATCTACTAACGCCCATTAGATTAGGATTTTCTGGAAAGGGATTCAAAAGTCAAAACTAGCCTGTTATTTACATATTCAGGTTTACCTGAACTCTGAATTTGCTGTACTAGATAGTTTTACTTTTACCTAATTACTTTTCTTTTTCCTTTCATTTAAAGCTGCGAGAGTACATTGATGATACCGAAGATTATATTAATATTCAAGTAAGTAGCTAGCAATTTACTGATAATATATCTATTGGTATCTGGTGAATGACATGAAATTTAAGTGAAGTTTTTTTCATTTTGCAGCTTGACAACCATCGTAATCAGCTGATTCAGGTGCCTTTTCACGTTTCTGCACCTGGCATGTTGCATGTCATCTATCGGTATTCTATAATGTTTTTTTTTTTTCACAAGTTCGCTAAATGGTCTTCAACTCTTGTGCAGTTGGAGCTCTTTCTTAGCTCAGGAACTGTTTGTCTATCTTTCTACTCTTTGGTGGCTGCTATATTTGGCATGAATATCCCATATACTTGGAACGATAACCATGGTTACATGTTCAAATGGGTATGCAAACTATTACTGTTTATGCATGCGCATGTGTAAACACATATATGTAGCTGCAAAATTTTGGACTTGATATTATGAATTAAGAATCAAAATACCCCATGCTTTCTTGCAGGTAGTTATTGTCTCAGGAGTATTTTCTGCGGTGATGTTTCTCATTATTACAGCCTATGCTCGCAAGAAGGGGTTAATAGGATCGTGAAAATCAATCTACTGAAAAAAGGGGTGAATAGGACTCCTACATATAACAGTCTAATAATGAATTTATGTTGAAGAGGTTTTTATTGTAGATGGTTCAACATCATGTTGAGTTATTGGACATGCTTCATGTTGAGTTATTGGATCTGCCATTTCCTCACAGCTATTCTGTTCTCTGGATTCAGTCATTATAACAGATTGCAAATACAGTCTACAGATTCAAAGTTGATTCTTGATTTTGAATAATTTCAAAGGCCTTGATACTTAATTTAATTATAATTAAGTTGAATATGCTAGTCTTTACATACTTTTTTTCTACTATTTCTCTTCTGTTCACTTCTATGTAAACAAGGATAGGATAAGTGCCACCGAACTCCAAAGCATTTCATCAATTAGATACAAAATTTAATTACATTGAA

>GLYMA.11G105300

ATGGTTTCGGTAGTTTTGCCCTTGATCATACACTTTGAACCTCACTATGGACAGACTAACATGCTGGTCTACTTGGGAATTTGTTCATTAGTTGGCTCACTTACGGTAATTATGTCTGTCTGTCTGTGATGAAGTTTCATCTTGATATTGCAACGTTTGGACTAACTGTATACTTGCTACTTTGTACATGAATTTTTCAATTTTTCAGGTTGTGAGCATAAAGGCCATTGGAATTGCAATAAAGCTTACACTTGATGGAATAAGTCAAATAGTTTATCCTCAGACTTGGTTTTTTCTTACCGTGGCCATAATCTGTGTCATTACACAGTTGAATTACCTTAATAGGGTGAGTGGGTGGTGAAACTTTGCCAGTTTGGCCTGCATTTGATGCATGATTTATGGTTTCTTATTCCAGTGCTGTTCCATGGGGCAGGGGCAAACCCTCTTGGCTTTTGTTATTATAGTTGTTTTTAAGAGGAAAACCAATCAAAGGGTTGGTCTTCTAGTTTTGGTAAATAGAGCAATAGAGCGTAGCTTTTTGAATTCGATTAAACTAGAATCAATATAGAACTCATACTCTCTATAAGGCAATGGCTTGCAAGTGACAAGCCCTTCATGCACAACTATAAACAGCTAGTCATATAGATAACAAAACAAGGCCCTACTTGATCTGTTGATTACATTTGCAGCTATGGTGAGAAGTAATTGTTATTGCTAAATAATGCACTGCAGTTGATACAATTCTAGGAAAGCATTAATATAGTTGATAGTCATGGACCAAGCATGCTGAATTCTCATATTGAAATACGTATGAACTGGAAAAAAATTTAATTATGAACTTGATACAGATCTTCAAACATGATCCATTGAAAGTTATACAGAGAAGTATAAAACACAATGTTGTGAACAGCCCTGTCCTCTGGATGTAATTAGACTTAGTCTCGGGAGGTTTTCTTTCCACATCCTTGATCCAAACATTTTTTTATACTTGCAAATAGAATTCCAGTAGTGTTTTCTCAACTGATACGTTTCTTTGGGTTCCCACTGGTATCAACTTTTCTTATCATCCGTCTATGATGTATCTGTAAGACTACAAGTATAACTCCCTTTTGCTTTTGACAAATCTTGAGCTTATTTTACTATTAAACTATTGCAGGCTCTGGATACATTCAATGCCACAATTGTTTCGCCTGTATATTATGTAATGTTCACCACTCTTACTATTATTGCCACTGCAATAATGTTTAAGGTATGATAATATACTCATTTTTTTTATTCCTTAGGTGATATCTCAAACTTTTCTTTTTGCTGAACAATAGGATATGACAAACTTGACTGTTTACTTTCAGGATTGGTCCCGGTCAGGATATCAGCAGCATAGCCTCTGAGATATGTGGATTCATCACTGTTCTTACAGGAACAATCATATTGCACATGACTAGAGAACAGGAAGAATCCAATATGCAAAGTACAGAATAACATGTTGCTTTTAATCTGATTCATGCCAGTTTCCATTTGTTTGCTAATGCATTTATAGACTCTTACAACTCATAGGGATTTGTCTATAGCAATTAAACCTGATGAAGTGATGGTATCTTATCTATTTGATGCAGAGACCTTTACATGGTTTATAGGTGAGGATTTGATGAAGGATGTTGAGAATGAACACCTGATTCTTATACACGATTCGGATTACCTAGAACGTTGA

>GLYMA.11G255400

CTGGGACGCATAGGTAACGAATTTACCGTTACCTGTGGCGTCGCGTCTCAGATCCCTCTCTCTTTGGAGAGAACGCCCCAACAATCTAAATCTGCATCGCATTATCATCATCATCATCATCAATACTTCATTTATGGGGTTTTATTAGCTACAATACTTGTTTGCTGCAGGCTGCCAACGAAAAGGTGGTAATCATTCCCTTCTCTTTGTTTTAATCATCTCTTGGCCATTTAATTGATTTCTTTCTAATCTTTATGCATGCATCAAATTGATTAAGGTCAAGGCATTTTGGATCAGACATCTTGGCCCTGCAAGCTTGATGCAACACAACAAATATATATATCATGGGGAAAACACACGACAACGTAGTTGGGCTTATATTGGCCATCTCTTCCACTGTTTTCATCGGTTCTAGCTTTATAATTAAAAAAATGGGTCTTAAAAAAGCTGCCGACCACGGCAACAGAGCAGGTTCAACACCTCTCATATATATCATCTGGGTTATTCACGTATTAAACTTTTTTTTATTTTATTTTAACATATATAACAATTTTTCATTGATTTGATCAGCCACGGGAGGGCATTCGTATCTGTATGAGCCGTGGTGGTGGGCTGGAATGATTTCAAGTGAGTGATTTTCAATTTTCTAATTCTAATTTATATATTAACGTAGTGGTTTCTGCATCAATCAACGTATGCTATATATATATTTATATATATATATATATATCTTTCTGTTTTCTCTAGGCCTTAATTTTCATAGTGCAGAATAATTCACACATACAGCTCATAGAATTTTGTGACGTAAACGTCCGGATCCTTGTCTCTATTTCCACTGATATTGAGAAAACTTCTTCAAGACATATATTACCTAAAATCACATCATGCATTTTGTAGTGATCGCTGGGGAAATAGCCAATTTTGCAGCTTATGCGTTCGCTCCTGCAATCCTTGTAACTCCTTTGGGAGCTTTGAGCATCATTTTCAGGTAAGCAACACACACATATATATACACATATGTATGTATAGCCTTGTTCGTTCACAACACAAACATATGTGCATGCAATTAAGTTAAGAAAGAGGAAACAGTTACAGGGTGGATGATATATATATATATATATATTTATGATGCAGTTCAGTGTTGGCTCACTTCATATTAAAAGAGAAATTGCACATTTTTGGCGTGCTTGGGTGTGCTCTGTGTGTGGTGGGATCTACGTCTATTGTTTTGCATGCACCGAAAGAGAAAGACATTCATTCTGTCAAGGAAGTGTGGGAACTTGCTACAGGACCAGGTAATAAGCCTTCATTAGTTGCATGCATGACATTCATATCTGGTTATTAATTAATTAATGAGCCTAAGCTTAATTGATTTCTTTTAAATTTTCCTATGATCGAAATCGAACAGGTTTTATTGTCTACATTTGCGCTATAGTGATATTAGTTTGCGTCCTTCATTTCCGTTTTGTGCGAAGCCATGGGCAGACTCATATGATGGTGTATCTCGGAATATGTTCTCCCACCGGCTCCATTACGGTATTAATCTAATGTTCAATGCTTAATTATACGTACGCACGCACGAGTATATCATAAACTTAATTAATGCTGATATCCTACCTAGTTTAATTTAATTTTGCATATATAGGTTATGGGTGTCAAAGCAGTGGGAATCGCTTTGAAGCTTACATTTGAAGGGACGAATCAATTTGTTTACTTTGAGACCTGGATATTTACAGTGGTTGTGATAGGATGTTGCCTTTTGCAGATTAACTACTTGAACAAGGTATGCTTTAATCTGTGCCATTATAAATGTTCAAAATTAAATTAATCTTTCAACTACGGGCTGTCATCCCACAGAGTAATTGTTTCGTTTCAATTTTTACTATAATGATCGGAGCACTAGTGTGAAAAAGTCAAATATTTCTCATAATGGGGATATATATATAATATCATGCATGTAGCATCTCGTCAACAGACACCTTAATTCTCATAATGTTATATGAATAGACTACCATTAATCCTTAGGAGATTTAGGTGTTATTATTTGTCCTAAAAGTTTTTACCGTGTCATTAATTTTTTTAGTTTTCCTATGATGAGCTAACTGCATATATATGCATTTCGTGATCTAGTAGCTAGGTACCATTTGGAAAGTTTACAAACTACATATATAAGACACTAGAACGATTTAGTACTGAATCTACTGTGCGCCCTGTGAGCCTACAAATTAAACACGTTATTCTTAAGGATGAACAGTTGATTTGGAAAACGTGTTTGCAGGCTTTGGACGCCTTTAGCACTGCTGTGGTGTCACCAGTTTACTACGTGATGTTCACATCATTTACAATCGTGGCCAGCATTATCACGTTTAAGGTGACTATACGGCTTGTTATTTACTACCTTCAATCATGCAATAGTTAATAATGACTTGATGGTGGATGCATGTATATGCAGGAATGGGCGAAGCAGGATTCAACGCAGATTGCTACTGAGTTGTGTGGTTTTGTGACAATATTATCTGGGACCTTCCTCCTTCACAGAACTAAGGATATGGGAAATAAACCCTCCGACGCCTCCGTTCATTCAAGTCCTGAAGATAACAATAGTAATACTAAGACACCTCTAAGCAACCAAATTTGAATCTATATAGCTTCTCTCCACAAGCATTATTTTGCTTCTTAACAAGCATGTAATTTCACACATTTTTTTTATAAAAAAATCAAAGATATGCGATGAAAATATGGAAGATTAAAGGAAACATGCCTATATATATGTGGTTTTGCCCAATAACTGCGTACTCCCCCATGCCCCTGCACAAATCACAGTTGCATATCATGTAAATCGACGGATGATATGTCATCCCCATTTTGTACTGTAGTTAATAGAAGAGACCCAATAAATGAAGCAAACGTTGCT

>GLYMA.12G030100

AATTGCAGAGTGTGAACAAAAAAAAAGAAGAAAGAACTGACAGCTAATGAGAGGAGAGAGAAAGAAGGTGAAAAATGAAAATATCCGTGTAGCGTGTGAAGGGATGAGCCACGTCATCAAGGAAATTGATTTTAAAGGGTCTTGTTCTTCCATCTTCAGCGACCAACACCTGTGCAGCAATTCTCTGTCACCAAATTTGATCTTTCTTTCTTTCTTTCTTTGGCGGGAATGACAGAAACGGGGGTTTCTGATAATTTCAAGGGCTTAATACTGGCGATGGGTTCTAGTGCATTCATCGGTTCCAGTTTCATCTTGAAGAAGAAGGGTCTTAAGCGTGCTGCTGCACGCGGTACTCGTGCAGGTCGTTTCTATTCATTGTCAATCACACAAATTCCCAATTAATACTTTTATTTTGTGACCAAGCAATTGCATTGTATAGTCTTTGGTATTTTAGATCAGATCCATGAGATGCTATGGTAGCTTCTGATCCCGTTTTTGATAATTTTGATTTTTGAATTTTTCAGGAGTTGGTGGCTATACTTATCTACTAGAGCCGCTTTGGTGGGCTGGCATGGTGACAAGTAAGTGTGGCACCTTGATTTCTTCTCGCTCTCTCTCTCTCTCTCTCTTTATCCAAGGTTGTTGATTTGAATTTTGAAATGAAACTATTTTGGATCTTGAACTATTTGCTTACAGTGATTATTGGGGAGATTGCAAACTTTGTTGCTTATATCTATGCTCCGGCAGTTCTGGTTACTCCCCTTGGTGCACTTAGTATTATTGTCAGGTACTCTAGCTTTAATTAAAATTTTGATTGTTTTGATGAATTTTGTGTTTGTGTGGATTGTAGAACTAACATATCCGGTATGTGTTGCTAGTGCTGTTTTGTCTCACTTCTTGTTGAAGGAACGGCTTCCGAAGATGGGGGTATTGGGGTGTGTATCCTGCATTGTGGGATCAATTGTTATTGTCATCCATGCACCACAAGAGCAAACTCCGAGTTCTGTCCAAGAAATATGGGATCTGGCCACTCAACCAGGTTGGATTTTAACCTGTTTTAATTTACCCTTGAAGTTAATTCCTATATTCCGGTCTATTACCTGATGTTTTCATCCCCCCCTTTTGTTTTTCAACAGCATTTCTGTTTTATGTGATGGCAACAGTTTCGGTAGTTTTGGCCTTGATCGTACACTTTGAACCTCGCTATGGACAGACTAATATGCTGGTCTACTTGGGAATTTGTTCATTAGTTGGCTCACTTACGGTAATTATGTTATGTCTATCTGTGATGAAGTTTCATATTGATATTGCAACACTTGAACTAACTGTGTATACTTGCTACTTTGTACATGAAATTTTCAAAATTTCAGGTTGTGAGCATAAAGGCCATTGGAATTGCAATAAAGCTTACACTTGATGGAATAAGTCAAATAGCTTATCCTCAGACTTGGTTTTTTCTTACCGTGGCCACAATCTGTGTCATTACGCAGTTGAATTACCTTAATAGGGTGAGTGAGTGGTGAAACTTTGCTAGTTTGGCCTGCATTTGATGCATGATTAATGGTTTCTTATTCTAGTGCAGTTCCATGGGGCTGGGGCAAAGCCTCTTGGCTTTTGTTATTATAGTTGTTTTTTAAGAGGAAAATCAATCAAAAGGTTGGTCTTCTGGTTTTGGTAACTAGAGCAGTAGAGCCGAGCCTTTTTGAATTAGATTAAACTAGAATCAATATAGAACTCATACTCTCTATAAGGTGATGGTTTGCAAGTGACAAGTCCTTCATGCACAACTACAAACACCTAGTCATGTAGATAACAAAACAAGGCCCTGCATGAATTCTCACATTGAAATATGTATGAACTGGAAAAAAAATTGAATTTTGAACTTGATACAGATCTTGAATCATGACCCATTGAAAGTTATACATAGAAGTATAAAACACAATATTGTGAATAGCCCTGTCCTCTGGATGTAATTGTACTTAGTCTTGGGAGGTTTTCTTTCCACATCCTTGATCCAAACATTTTTTTTATACTGACTTGCAAATAGAATTTCAGTAATGTTTTCTCAACTGATACGTTTCTTGGGGTTCTCACTGATATCAACTTCTCTTATCATCCATCTATTATATAACTCCCTTTTGCTTTTGACAAATCTTGAGCTTATTTTACTATTCAAATATTTCAGGCTCTGGATACATTCAATGCCACAATTGTTTCTCCTGTATATTATGTAATGTTCACAACTCTTACTATTATTGCCAGTGCAATAATGTTTAAGGTATGATAATAGATTCATATTTCTTTGATTCATTTGGTGATATCTCAAACTTTTCTTTTTGCTGAAAATAGGATATGACAAGCTTGACTATTTACTTTCAGGATTGGTCCGGTCAGGATGTCAGCAGCATAGCCTCTGAGATATGTGGATTCATCACTGTTCTTACAGGAACAATCATATTGCACATGACTAGAGAACAGGAAGAATCTAATATGCAAAGTACAGAATAACATGTTACTTTCAATCTGATTCATGCCAGTTTCCATTTGTTTGCTCATGCATTTATAGACTCTTACAACTCGTAGGGTATAGCTATTAAACCTGATGAAGTGATGGTATCTTCTCTATTTGATGCAGAGACCTCAACATGGTTTATAGGTGAGGATTTGATGAAGGGTGTTGAGAATGAACACCTGATCCGTATACATGATTCAGATTACCTCGAACGTTGAATTTTCCTTGGAGGTTAGGTTGATGTTCGAGAACATTTTCCATCTGTGCTGATACAATGGCGTGAGCCTGCCCATGGTCACATGATGTTTTGCAGTGTGGATTGCTTGTCCTTGAGCTTAATGAATTCAAGCATCCATGGTGCTATAGCTTTCTAACCATTTCTCCGCATGCTATTTGCATGCAGCTGTTGACTGGAGCCATCTCAGATTTCAAACGGAAATGAGATTGCTGTCTCATTACAACAAATGGAATTTTTGGAGCGATCTTATGCCCTGTTCTATTGTCCATCTTAGCCAGTGTAACTTTTCTTAACACACACACACACGTATCTTTTGGTAGGAAGTGAGCAATTGTTTATACAGTTCCGTCTGTATTTCTCATTTAATTAACTATTAGATTTATAGTTTATTTTCTTGAAATAGGTAAATGATAGATCCGTTGACTTCCGCGGAAAATGTTGAGGATATTTATTCTCCGATCTTCTGCAGTAAAAAGTGAATTATTATCTATTTTTTTATCCTCCAGACCTGAGGATATTAGCAAGCAAAGATGACTTGATACAGTTATTCGCATCAAAGAACCTGCTAGTGGAGTAGTGGTAGCGAGTTTAGCGTACTTTTGCATGGCATCTATCTTTGTTCTATCCTCATTCATTCCTCACTTATTTATAATACAATTCGTTAAATTGTTGCTTCGATTCGAAGCG

>GLYMA.12G168000

AAAAAAAGGAGAAAGAAAAAAGGAGGTGAGGAGGAAGACAAGAGAGTATTGGGAATTTGGCAAACGAGAGAGAGTAATTCCCAATCAAGTAAAGATTTAGATTAGCAGGAATGAAGAAGAATGGAGTATAGTAGTCATCAACCTTGTTAGCAGTCTCAGTCACACTGTAAATTTTTGGTTACTTATCTCTCAAAATCTCAAATGCATATGCAATTGCAAAGCATTTCCGATCCTGAGGGAGAGAACTTTTGTGACAGCTGTATCCACTCCGATTGTTGATTTTTTATTTTCTTTCTCGCGGCAAGATCTCACTCTGCACCTAGGGTTAGGGTTTTGGCATGGCGACTTCTTCTTCTTCTTCTTCGACTTCGAGCTGGCGCGAGGGCATGTCCTCCGACAACATAAAGGGACTGTGTCTCGCTCTCTCCTCTAGCTTCTTCATCGGTGCCAGCTTCATTGTCAAAAAGAAGGGTTTGAAGAAGGCCGGTGCTAGTGGAATCAGGGCCGGTATCGTTCATCCTTCACTCTCGATTTCTTTTGAATTAGTACTCGTTTCTCGTGCTTCGCTGATCTCCCGAGGAGTTTTTATTCACTTGTATTGAGTTTATTTTCCGTGTTTGGTTAAGTTTTCTTGGGATCTGGAGCTTTTTTTCTTTACTCTCTTGGGCCGCGAGTTCATCTGTCTCAAATCTCTCAAGTTTAAACTCGATGCGTTGCTTTGCCTGGCTTTTTTTTTTATATCTTCTTGCCCAGAAATTGTTGTAGAATAAGCTTATTTGGTCTCGGCGTGAGGAGGAAGGGAACACTTTTATGTTCTCGATTCTCATCCTTAAGATTTTCATCCAACTTTTTTTTGGCAATTTTAACAATTCTTTTCTTTTGCTCCTTGGTATGGAATGATGATAGATCTATATTATTCTAGCCGCGTCTTTATTGCCACGAAGAAAAATGGAGTTTGTGTGACTTGAGATCTTGCTTTAAATTTTAGAAATCGGTGGATAAACTGTCTGCTAGTTTCATGTTTTCATGTTGTAGCAACTCATCACTAGGGAAATCTTTTATTCTTCTCCTATAGAGAAATTATATTATGTTTTAATGAAATGGATGCTTAACTCTAAACTATATGGGTCTCTTTTCTTTGATTTATTTAATTTACTCTACAAAATAGTTGTTGCCTAATGTTTCTGTATCTCTAGCATTTGCTCTTTCTGTTAGGATGGATATAATATAAGTCAGTGCACTTGTATTATCGTGTGCTGTTAATAGTTTTTATTTTTTTGGTTCATTGTCTCCCTGATGGATCATGTGCAATAGTGACTGACTATCCAAAATTATATCTGACAGGAAGTGGAGGTTATTCTTACTTGTATGAGCCGCTTTGGTGGGTGGGAATGATAACAAGTGAGTAATACATATTATTCTGTTTGGTTTTGGAATGACAAATGAATTGAATGTATTGTTGATTTTTCCTTTCCCAAGTGTATCTCTTGAGCTCCTGCCTTTTTTTTTATATATATTTTACAGTTATTTTACAAGTGCAGAAAGTAATGTAGTTACTATACTTCTGTAGACTATAGATTGAGTTCTATGTAGCACCTTAACAAATCCAAAGAGGGGCACTCATCTGTATGTTTTCCCCTTCATCCATGTATAATCCATCTTTCTGCTACTTATTATCCAAATTTATGCATTGCACAAAATAAAGAATAAATAAAAAAAAAGTTGCTAAAGCCTGTACCTTCATCCTAACTTGTTCTTTTATATCCTTTCTTTTGCTTCACATATTTTCCAAGGGAATATCTCAGCATGGACACAAAGGGCATATAAAGATGCTATGCTATGAGTTGCATATACTATTAAGTGGATTAAAATTTCTATTATAATTTTCTAACACATTTGCAATTTCAACAGTGATTGTTGGGGAGATTGCCAATTTTGCAGCTTATGCATTTGCCCCAGCTATATTGGTCACCCCTCTTGGTGCTCTTAGCATTATTATCAGGCATGATAAAAATTTAGCTTCAAATTGATGCTTGACTCATGGTAAATTCTGATTTGTGATGTTTGCTTGCAGTGCTGCTCTTGCTCATATTATTTTACGGGAGAGGCTACATATTTTTGGAATTCTTGGTTGCGTTTTGTGTGTCGTGGGATCTACAACAATTGTTTTGCATGCTCCTCAAGAACGGGAAATTGAATCTGTTTCAGAAGTGTGGGATCTTGCTATGGAACCAGGTAGCCATGTTCCTACTTGATATATCACTGTCTTTACTCAATAGATATCGTATTTAACGACTAACCAAGAACATAATCTTTCTACTGCAGCATTTCTCTTTTATGCAGCTTTGGTAATAACAGCTACTTTTATCCTTATCTTCCACTTCATTCCTCTCTATGGCCAGACACACATAATGGTTTATATCGGTGTTTGTTCCCTTGTAGGTTCTCTAACGGTATGTCTCATTATGTAGTCATTGATGACTTTCTAATAGTATTTGTCTCCCCTTGTTATTTTCCCCCCTGTATTTGTATTGTAAGGTTTTCCTTTGACAGGTTATGAGTGTTAAGGCTCTTGGAATTGTCATAAAGTTAACACTGTCGGGGATGAATCAGCTAATTTACCCTCAAACTTGGGCATTCACTCTAGTTGTAATTGTTTGTGTTCTTACCCAAATGAATTATTTAAATAAGGTACAGTAGTTTTTCTTGTTAGTTCCCTGAACTTTTTATTTATTTGATGTACTATTATGTACTTGTTTTTGTCAACACTCAACAAGTTTTGTAATGTACTTCACTACTTATTTTGGTCAACAGGTATCATGTACATGAAAATAGTTTGATTGAATTTCTGTTACCATTCAGTGAAATCTGTGAAAGTATCATGTATTTATGCTCACAATTTTTTTAAATGTATTTTGATTTTTAAACATTTTATGTATGTAATCTTTTCTTGCTTGTGTTGAGAAGAATATAAAATTGTCTAAGTGTTGGTTATTTATCTTCTTGTTGGACTTATGCATGATATGTTTCTTGAACCATAATTCTTTTTATTGTGTAAATCTTGTAACTTTAAACATATTACCATCTGATATAACCTTGTGATCTTTAAGTAAACTACTGTGATTGATTGAAAATTTTATCCCTTGGTTTAAAGATAAAGAACTTGATCGGAAAATTGACAGCTAAATGAGGAAGGTCAACAAGGAAAACAGAAGTTTGACTCTCGTTTGTCAGTTGGCCAAGATTTCCCTGTCTTTTAATTTTTTTGGGAAGAAGATATGAGAAAGCTACTGTGATGAATTTTGTAATTTAAATCCAATGGGGTGTTTGGTAGTTTTTTTTTTTTAAAGTTTCCTAGGAATCTTAGGTTGGGAATGTTAAATTCCTATGTCTGGTATAAATATTTTAAAAATCATTCCCAGGAATTGTTTTTTAATTAAATTTCCCACTAAAACTTTCCCATGGGAGGGGTTGGGAATCCAAGATTCCTACGAAGGGTGAAAGTTTTTTGTATAATTATGTCTTTCATTCCTGGGAATATCTTAGACAAACCGAACATATTTATGACATTCCCAGGAAATATAATTCCTTGGAGACATGATTCTCGGGGATGAAAAAAGACTTTGTATACCAAATATCCCCCCTAAGGGAAATGTTAGCAACACTATTTATTATTGGCTGATGTTTATTAAAAATCTCAAATATGTGTGGGTCGTATTCCATATTTAATGAGCCCCCACTCATAATTTTGTAGTTTCCAATAAATTTTAATCAATAATAAAGAATGCGTTGAAAGAGTTGTGTTGCTAGCACTCCTCTTAAATCTAAATGCACATAAAGGCAACTTTGGACATGGATGTCAAACTCAAGTTAGCTCTTAGACTCCTATGATTTTACTGGTCTAGGTAGAGAAAACGAGTTAACTTGCTATCAAACACTTTTAATTTATATATGATTTGGTCATTTTAATGGTTTGATCTTTTGTAGTACTAATGTACTATCACACTTTATTGTTGTTATTTGTTACTTGCTATATTATGAAGTTGAAATGTTGATTTATTGTTGTGTTAAAAGTTTAATGTTATGCAAATATGTCATTAATAGTTTTTTTTATATTATTTTTTATATTAGGTAAAACTTTTACGAGTTTACGAATCGAGTCTACAAATGTTGCCCGGGTTACGTAGACTCTCGAGTTTGACAAACTTGGGTTTAGGTATCTGGATGGTTTATTATGTGAACAAGTTCATTTTCTCTTTGGCTTTAAACTTGGATATGCTGGATTAGGATAAAGCTATATGTTTCCTTGGTGCTGGATGTCGATTTGTCATGATTCTGCTACTTACTAGTATTAGGCAATTACTTTTACATACTGTCACCATTTTTTTTTTTGGGGGGGGGGGGGGGTTGATGAATAGTTTTTTCTGCTTTATAATTCATGTGATAATATTTTTTTCATTTCTGCAATCAGGCACTGGATACTTTTAATACGGCAGTGGTATCTCCCATATATTATGTTATGTTCACTACATTTACCATTGTGGCTAGTGTTATTATGTTTAAGGTAAGTTTTGTTACCTAACTGGTATAGTTAGACACTGCTTCATTACTCTAATTTTAAGATTGTATAAATTGTGTTAAAATAATCCGGATTGATTGTATATTGTATAATCTGTGTTTGTTAAGGCAATGCTTAATATCATTTTGTTTTGGTGTTGCCACTAGATCTTCAATGATACTTGCATGCTGGTTGTTGCCTATAACTATGTGAACCTAATTTTATTTGCTAGTCAAGATTCTCTTGGTAGGTCCCTGCATATACCTGTGTAAAGAACTGCTTGCAAATGAAGTGTGAGAGGTGAATGCTTTTATGAATAAACGAGTGTCTGCTGAGGCATTTACGTCTTGATGTGAAAATTGTTTTCTCGTAAATTTCAATAGGGTGTGAAACTTCTTTTGCTCTTTCCCTTAACAAGCATATGGAGATGCAAATCTTTAATTTTTGTCTCACTATTTGTCTTGAATCTGATGTGCAAATCATCAGTTTAATTGTTGCTGTTAAAAAAAAAAATCAGTTTAATTGTTACTTTCACATTCAATGTCTGTCCCCTCTTTGCCATATCTTAGGGTTGACCTTTGATTGAGAATTGATTTTGATGTCAGGACTGGGATAGACAAAGTCCAACACAAGTTATCACAGAAATATGTGGGTTTGTGACCATTCTATCAGGAACTTTTCTTCTTCACAAAACTAAGGATATGGCTGATGGTATGATCTAGTGATCCTACAGTAGCAATTTCATGTAACTTTAGATTACACTCTCGTGCTCACATGCACTTTCTTCAGACATGACTTTGTTAATATTTTCTTCATGTGCAGGTTTACAACCATCTTTATCTGTTAGACTTCCTAAGCATTCAGAAGAGGATGGCTTTGATGGTGGTGAAGGCATTCCTCTTAGACGGCAAGAAGCCATGAGATCGCCATGATATATTCTTTGCATCACATCTTGCTTTTCTAAATGAGCTTGAGGCGCTGGGGAGGGATTTTGCCAAGGTCGTCTGTAATTTACTGATTATTTCCCTCCCCATCATATTTTTTACCTGATACAGTTGGAGTCGAAGGATGTGAGTTTCCTATCACTGTTCTTTTGGTCTCCCGATGATTATGCTTTCTGATCTTGCCTAGCATGCCCCTCTACACTCCATAGCGGAGACAAAGGCCAATGTAATTTTCTGCACATTGATTATATATAGTGTACCTCAGTTAAATGAAAATATTCATCATCAAATTTTGAACTGGACAGCATTGCAATGTTATTATCAGTGTCTGCTGCCATTTGAACTCGAGCTGACCTCAATATTTTGTGCTTGTTGGAGCTAAATATAAAAATTAGGATGTGTAGATTCCAGGTCAAATGCATCGTTAAATACGGTGGATTGTTTTAAATTTTAGTCGCTTTGTAGATTTAGCACTACGCATGGTCTTGGCATCTTTCTGGGAAATATATGGTATAACAAAATCCATAGGCCCTAGTCTTTTCCTTTCCTCACTTGATAAATATCTTAGCGTGCAT

>GLYMA.13G368400

TTTTTTTGCGATAGTGTTAATATGAATAGATAGGTTTTGGTATTTTGAGATTTGAACATACGTGCGTATAGTGAGAAGAATGAGGCGAAAGGGTGTGGGAACGACGGGAGTGAAGAGTTGGATGGTGGTGTCGGAGACAGGGCATGCGCGGCTTGAAGATGTTGGAAAACACTCCATAATGCGGCGAACTGGGTTGCCGGCGCGTGACCTTAGGGTCCTCGATCCCGTTCTCTCGTACCCTTCTTCCATCCTAGGACGCGAAAGAGCTATTGTTGTCAACTTGGAACACGTCAAAGCCATCATCACTGCCTCCGAGGTCCTCCTCATCAACTCCTCCAACCCTTTCTTTCTTTCCTTCCTTCAAGATCTTCACATCCGTCTTTCCAATCTAAATCCCTCCTCGGTACGTACGTCTTTGTTCCCTTCTTCATTTTTTTTTTATCCTCAAGTGTTACTCCTTTAATTTTGTTCGAAATGTTAGTTTGGAGAATTCAAATTCACCACTTCTCTCCTCTTTCTTCTTCTTTTATGTTAAATTAATAAGATTTTGTGTGAGTCCACAAAGGAAAAAAATGTTTGATTAACAAAGTTTCTTTTATTTTAAACAATATCTAAATTAAAATTTCATCTTGATATAATTTTTTGATGTATAATAACGACACAGAAAGTTAGAAACCTAATATCTCATGTAGATTACTCACCACTAGATCGATCCTAATCGGTTCTTCTCGATATAATATTTATGTATGAAAAAATTACTAATAGTTTTAATGGTGCGAATGTTTAGGGTTTGGAGATGATTTCCTCGTAAAAATATTTGTAGTAGATGATGATCATGCACATTGTTTACGGTGGCAGGGAAAGTAATATTTACTGATCTAAACTTTTCATTACACAGATGAGCAATGACATGGATGGTGGTTACGAAGAAAAACCTCTAGCGAATGATTCCCGAAACGGTTCACCTGTGAGAATACCTGAGGACTCTGATGCCGACTTTCTTGTAAGAGCAGATAGCCTTAAGAGCAGTGCAGAGACTGGAACAGGAACAGGAACAGGAACACCAGCTCCTAAGCCGTTACCTTTTGAGTTTAAAGTACTTGAGGCATGTATTGAATCTGCTTGCAGGTGCCTTGAATCTGAGGTATGTACTAACTTAGAACTGTATTTACTCTATCTCAGATTAGGTTGTTGGTTGAATTTTCCATTGGGTAAAATTTCTTCAATGCATACTGTACACCTCCGACCCTCTGGGTCAGACTCTTTCAGATGATCTCCTCAGTTTTGATCTCTTCGTATTTATGTACTTGTGCAAGACTGTATACTCTTCCGGTTAAAGATCCATTGATTTGAATGTCGAAGGATCTAACCTAAATACGATATTATTATATAATTCATACTAAACCTAGAAGGAGGGGGACTACATTCTCATACTTAAATGTTTTTGGACCAGGTCTCATTTCTTCCTCGGAGACATGACTTAGCTACATACATATTTTTTTTATATATATTTATTACTAGTTTGAGGGCTTGTCCTGATGCAGCGGTAAAGTTGTGCCTTGGTGACTTGTTGGTCATGGGTTCAAATCTGGAAACAGTCTCTTTGCATATGCAAGGGTAAGACTGCGTACAACATCCCTCCCCCATACCTTCCTTCCCCCATACCTTCGCATAGCGAAGAGCCTCTGGACAATGGGGTACGAAGAAGAGATATTTATTAGTAGTTTCCCTTTCTTTAACCTGATGTTTTCTTTGTTTTTTTTATATATTTGACAAGTCCAGACGCAAGTACATTCTATGTATATGCTTATTATAAGCTCTATCTGTGACTTGTGTCTATGTTAGACTTCAACACTGGAGGTAGAGGCTTACCCAGCTTTAGATGAATTGACCTCTCAACTTAGCACACTCAATCTTGAACGTGTTAGACAAATCAAGAGTCGTTTGGTTGCACTCTCCGGTCGTGTGCAGAAGGTATATAATATTCATCATCCAACACCTGTCACCATGCATCAGGATTTGTAACTACTTTACATAAGAATTTTTGGAAAATGAAATTAATACACCCTCTGATAATATTTTATAGTACGTAATCAGCAGTGTGCAAATTGATTTGAGCATTAGCAACTAACCATACCATTTCATTCTTCTTTCATGGATGCCAACTATAGTAACACTAAGCAAGTCTTAAAGAACTTGTAACATTTTGTTTTCACCGTGGCGATGTGCAACTTCTGCAAATGCTGGTCGTTTTGTATTGCATTTTGCAAAAGCACTTGTGAGGTTCTTGTTTCATTGCCCAGTTTTGTTCTTAATTCAATTTAGTCACATAAATTGTCCACTATATGCAAATGGGTTTGCAAGTTAATTTGGAAGAAATGCTAGGAGAACATTTGGTTGACTCTGTATTGTTGTAAAGAATATTTCGGGTAGGAACATGAAGTCTATATTTTTTTTATCAGCAACATAAAGTCTATATTGTGAACATGAAAATGTGAAATCCAAAAAGAAGCAAGGTGATTAGATTTGTGGATTCTTTTAGTATGGATTTACTGTATGAGTATATTTTTGCACTAATATGGAGTATGCCTAGGGATATATTTTGATTAATTGTTTAATGGAGCTACGTTGTTTTCAATAAAATAAATCTAGACTAATTCTAGTCTTGGATTTTACAGGTAGCAGATGAACTGGAACATTTGTTGGACGATGATAATGACATGGCTGAAATGTACTTGACGGACAAGCTTAATGCTCGTTTATGTGATCAAACATCATTAAAAGAAGGCTACAATTCCGAATTTGAAGATAATGATCAAAGGTAAAAAGTAAAAACCATCAATGTTTGTTTATTTAATTACAGCTATAGTCTGTATATTTATAAAAGCTGTCCAGCAGCATCTTGTTATATGTGGGTTATTGGATGCTGGACAGTAGAATTATTTATTTTTTTATTATAAAAAATTCTCACTTTAATTTTTTAAAAGTTGTGCAATAGTACATGCCCACACCTAGTCTAGACTTCAGCTTAGCAATTACACGTGTCATGCTCACAAATCACAATTAATGGTTGCTTTTGTCTTTAGTGATGAATCAAATTCAGAAAAGTATGACAGATTCCTTTGTCCTAAACTTGACGTTGAGGAATTGGAGATGCTTCTAGAAGCATATTTTGAGCAGACAAATGGAATCTTACAAAGATTGACTAGTGTAAGTATCGATCTATTGAACTCTTTTATCCTTTCAAACACTAACGAGAATGATTATTGTAGGTTTTCAGAAAAAGAGAAACATTTGTGGAAGAGAAACACTTACTATGTACTTTGCTGCATAGAAAGGAAAGAAATAATTAAGATTTAGAATGATGGAAAATCAGTAAGAGAAAAATAGAATGAATGCAAACAAGTGAGCAATAGATTAAATTTAGGAGTGTTAAGTTTAATAAGAGAAATAGAAAAAGAAATAAGAACATGAGTGTGTTTCATAAAGCACTTTAGTAGCTTATAAGCTACACTTTTGACTAATTTATAAGTTTATTTGACTAAACTTAATTAAAAGCAATTGTTTCTTATTCTGGGTAGAAAATTGATTTGTGTATCTGAGAGACATGAAAATCGATTTTCATGCTGAGAGAAAGAAAATTAATTGTCATGATGGATGTTGGACATTTGAAAATCAATTGTTGAATGGATAATATTCTATTATTGCTTTTAAAATTTAATTATTTTTCATTATATACACTTGTGATAAATGATTATCAACAAAAACAATTGATTAATTACTTTTGGTGAGAAAGGTACTCCATTCGTTTTTATGCAAATTTGTAAAGAAATAAAAAGAAATAGGATTCATATTTTTTTTCATTTCTTTCTTCTCTACTAAACCCATGTTAATATTTTTTCTTCTTTCTATTTTCTTACTATTTCTCTTCTACTAAACGAATTAACGAAGCAAATTTAAAACATAAATTTCTTATCTAAAATACTCACTGATGTATGTGTTATTTGATTAAGTTGAGTGAGTACGTGGATGACACGGAGGACTACATCAACATAATGTTGGATGATAAGCAAAACGAGCTTCTGCAGGCAGCAATAATATTCGACACCATAAACATGATACTTAATGCCGGTATTGTGGTGGTAGGATTGTTTGGCATGAATATTCAGATTGACCTCTTCAATGGTCAACCTCGTCAATTTTGGGCTACAACAGGGGGTACATTTGGAGGATGTCTACTTCTATTCCTTGTATGTTTATGGTGGGGCAAGAAAAGATATTTTCTCTCTCACTAGAGGCCAATTA

>GLYMA.14G033700

GGGGCCCTCACTGGTGGTGTAGAATAATAAGGTTGTGGTGGTACACACTATATCTTTTCCTGCTTTCCATGCCACAATCATGAAACAGACAATGATGATGATCTGAGGAACCGTGTTTGTCTCGTGTCTCATCCATGAAGAGAAGGAGAAGAAGGGATCCAAAATCAGAAGAAAGTTACGAATTTTCGAGATGGGTCTGTCCAAGGAGAATCTGAAAGGTCTCATACTAGCTTTGGTGTCAAGTGGGTTCATTGGGGCAAGTTTTATCATTAAAAAGCAAGGCCTTAGAAGAGCTGCAGCAGTTTATGGTGTCAGGGCTGGTGAGGGGATTTCTCCTTTTTCTTTTTTCTATTAGAATTTCTATTTGGAAGCTTGTTTTTGGTTATGCATGTCTTTGTAATTTTTTTGTCATTTTGTAGGTGTTGGTGGGTATTATTATCTCTTGGAGCCATTATGGTGGGTGGGAATGATCACAAGTAAGCTCTAAGTGCTGTTTTAATTTTGTTTCTATGCTTTTAGTATTGTCATTTTTGTGTTTTTTGGGGGTAGAATCTAGATTTTCTTGTACCTTAATGGTTTTATTTTGTCTTCATAATTGGTTTTGATTTTATCCAATCTTGTATGGAATTAATTGGATTTTATTTTTAATAGAATTTTTGAATCTCATAAGTTAGGATGAATTAATTTCGTTCTGCATTGTATAGTGGATCCTGGATTTGATGTTTCAGAAGCTTTATTATTTGCTAGATTTCTTTATTATACACTCACATATGGTAGATCAGTTGTAGTGAAGTTGAAGGTTGATTAAAATATGCAAATTTTAGTTTATCGGTGATTTTTTATTTATCTGATTTCTTGCAGAATCTGTTTCTGTATGGTTTATTGTACAGTGATTGCAGGAGAGGTTGCAAACTTTGTTGCATATGCGTTTGCTCCTGCAGTCCTAGTTACCCCTCTTGGTGCACTAAGTATTATTGTGAGGTATTCCCTTAATTTTCATTGTTGATTGAGAATTAAGGTGGTGCTAGCTTCTAACATTGGCGACATAACATGGTTGCTTCTTCAATGATGTTTGCATGGTATCCTCTAGTGCTGTTTTGGCTGACATTATTCTGAAAGAGAAGCTACACAATCTTGGGATTTTAGGCTGTATAATGTGCATTGCTGGTAGTATCATTATTTTTATTCATGCTCCTAAGGAACAACCTATTACATCTGTTCTGGAAATATGGAATATGGCTACTCAACCAGGTTAGAATACACTGTCTTTTTTCTTTTTCTTTTTCAATGCAATTCATTGTAGTTCCTTGATAACAAAGATATCCTCCCTGCTTGCAGCTTTTCTGGCATATGTGGGCTCAGTAATAGTATTGGTTTTCATTCTGGTCTTCCATTTTGCACCAAGATGCGGGCATACGAATGTGCTAGTTTTTACTGGCATTTGTTCATTGATGGGTTCCCTCTCTGTAAGTAAATTAATTAACTGTTATCTCTCCTGCTAAGTCTTCTGTTTGATAATTGATCTTAACTTCAAAGTTTAGTTTACTCAAAGTTATTAACTTTAAAACATATACAATCTAAGGGACACGATTTCATGTCAAAATAATTTGGTTTTCACTATTCATTCCTATTTTTTAATAATGAATTTCATTTCATAAATAAAGTTTAAGCTTCCATATAATTTTTTATATATATTTTAGTTGTATGGTTATATGAGACTATGGTCTAAAGTTTTCTCCCTTTCCCCAATTACATGGATAATTATTTCTGTCATAATAACCAATGATATTTACTGAAAAATTGTAGTAACGCTGCAATAATATGGTGGCTGATCTAAATTGAATTGCTATAATTTTGCAGGTGATGAGTGTTAAAGCCCTTGGAACTTCTTTGAAATTAACTTTTGAAGGGAAAAATCAGTTAATCTACCCAGAGACATGGTTTTTTATGTTAGTTGTGGCTATATGTGTCATCATGCAAATGAATTATCTTAATAAGGTTTGTTACATGATCTTAAAATTAGCATGCTGGGCTTGTCGTACATATATTTTTATCTTAATAAGGTTTGTTAGTGAATCCGGAAACAGCCTCTTTGCATATGCAAGTTGTGTTTCATCTACTCTATCTTGATCCATATATTCCATAGGAACTGTTTATTTTTGTGAGCGGGGGGTGATAAAAGAAAGATTTATTCTAAAGAGGAGGAAAAAATACTAGCGAGGAGGATATCATTGGGCCTAAGAGGCTAAAAAATGAAAAATGCAACTAAAAACTTCGTACCCCATTGCCCAGAGGCTCTTCGCTATGCGAAGGTATGGGGGAGGGATGTTGTACGCAGCCTTACCCTTGCATATGCAAAGAGGCTGTTTCCGGATTCGAACCCATGACCAACAAGTCACCAAGGCACAACTTTACCGCTGCACCAGGGCTCGCCCTCAAAAAAATGAAAAATGCAACAAAGCTGCTAATTTCTTTATATATGAAGGAGACTCCCTTACAAACTATATGGGTATGGTTGTGGCAAGGAAGTGAAATTACGCCCATGAATAAGGATATGAGAGATTATGGAGAAAAAATAAACAAGCTTCATAGTGTAAGGAGCTAGTCTCCAGCTATTACCCTAGCTTTCTTTTTCATTTTCTTGGGTTGCCACTCTGTAACAGAGGTTTATACCTCTGATATAGTTCTAATTGAGTATCAACATTAGTTTTACCATCCTTATTCTGGTTCCTGACAACATTAGTTTATTCTTCTAAACGATTGTACTGTAAAACTAGTTTTGAACAGAGCTTAATGGCATTGATTGATTAATTATCCCAATACCTAATGGGATAAGGCTTGGCTGCTGTTGTAGGCATAAATTTCATAGGAGTATCTTTGGGGTGACTCAAGTTTTGAGATTTTGGAAAAGTCAGGAAAATAAGTTTATTTACTTGGACCTTTGGCATATATTATAATATTGCTAAATTTGGAATGAAGCAAAGCAAAGAAGCTCAGATTTGAGGCTTCTGTTTTGTTGTGACTTATATATGAGTTTTTATTTTTTATTTTTATCATTTTGTAGTGTTTGGTGTTTTTGAGGAAAATAGAGGCGGTCTTGTTGCCAGACTCTTCCTTTGAATTGGAATGCAGGGAAGTTTAGCTACAACTTCATATACAATTAAGGAAGATGACAACTTAATATAAAACAGAATGAAAAATCTTACGAAATGAAATTCTGCTAAGCTTTTTAAAAGATCTAGAATTCTTTTCATATCTCTTTTTAATATTTCTTTTGTTTAAGTTAATCTTATCAGACAGTAAGAGGGGAATTACATTCCATGGGAGGACTGTTTCTGATAAGATCATCAGTAATGGCTCCTATGATTTTACTAGTTGCATGTTAAATCCTCCATACAACATACAGTATTACAACATGCGAATTCCTATGGAATGTAAATTCAAGATAGAGCAAACCTCAGAACTCAGAACTCAAGATGAGTACCCGACACTAAATCTCAGAACTAAGAGATTACATAATACAACAAAAGTCAAATCAAGATACAACAAAGTAAATTGTTTTACATCATGCAACTAGTAAAGGTTTGGTGTTTGGTACTCTATCATTGCTTACTGATAATCTTTAATCTTATCAGAAATTGTCTTCCACTATCAATTGATTGTAAGTTCTCTATACAACATGATTTTACTTTTGTAGTATTATGTAATCTCTTCTGAGTTCTGAGGTTTACTTTAGGTACTCTATCATTGCTTGTACAAAAGAGATCTGCATATTAGTAAGCATGTGCACCTGAACATTAGCCTGGTTAGGTGGTTACTTTTCTGTTTCTTTACTTTTGTTGGTTTCTTAATCTGTTTGAATGTGCTATCCCTTGATGTAATGACTTGGTCTTTTAGATGTTTAAAATGAAAGAATTGTTTAATTGTCGAAAATACTGGCATTCATATAGCTAAACTAGCGGTATTGAGAACTCCTCACAATGACATAGTGATCCATTCTATTTTGTGAAGTTTATTCTTCTTTTTTCTTTATCAAGTCTTGTTCAAAATGATGTCTGAGTTATATTCAATGGAAAAAGAAATCAATTCTAGAGAATTCAGATTTTCTCTGTGCATTATATACAAAGTTCTTATATATGAAATTTTGGATCAATTATTCTTCACTGACATGATTGCAATATTTCAGGCTCTTGACACCTTCAACACAGCAATTGTATCTCCTATATACTATGTCATGTTCACAACACTTACAATACTAGCCAGTGTAATAATGTTTAAGGTATAATCAACTAGTTTTGGCATTTAATGCTAAATGCATAAGATGTTTCTGTTGTCTACGAATTGTACAAGAACCTATTTACATTTAAAAATAATGCTTACAGGATTGGGATGGCCAAAGTGGTGGAACTATTGTGTCAGAAATATGTGGCTTCATCGTTGTACTCTCTGGAACAATAATGTTGCATGCGACTAAGGACTTCGAGAGAAGCTCTTCTTTTAGAGGTGTGATAAATTTGATCATAGCACTTAATTCTGCTGCCACTGACTATCCAGATGAATGTATGGAACCATAATTGATATTCTTTCATATTTGCATTTGTTGAAACAATTGCAGGCAGTGCTCCTTCATCGCCTACGCTATCTGCCCGACTTTTTACCGGAAATGGGGACTCATTACTTAAGCAAGATGAGGAAAATGGATCTCCCGAGAGTAATATGTGCTCAAGAAGGCAAGAGTTGTATTAGATGGTTAAAGATCTTAATTCATTTAATTGTAGGGGGAAGGAGAACCCTTCCCTGGAGAATCTGAATTAGACCATAGAATGATGGGAGATTTGTGAAGCCATTCTTTTATCTTGAGCATCTTAGGAATTTGTCCTCATAGATTTTAGTTTTGACTTTGCAAGACGGTTCTGGAACCAATGCTTTGCAGGGTACATAATGTACAGCGTGTCCACAGTTTCTTTTGTCACGAATGGTTTGTTCACTTGTCAAAAGTAGCTAATATGCTTCAAATAGTTAGATTGAGATTAGTTGTATTTCTGAGGCTCTCTTTACATGAACCAGAAAAGAAGTGAGTGGGGGATAGAGATTATTAGTTTGTGAAAATCATATGCATGGTGATTTTGCTTTGTTTTTCAATTTTATTTATTTGTTTTCTTTGTCAATTTGGCTTGGCATAGAGGATTCATTTGTGGTTTGTAAGTAATTAGATATGTACAGAAAGCTAAGAGACTTCTCTCTAAATTTTAGTTTGCTATGAATAAGAAATATGTTTTCATTCTTACATT

>GLYMA.14G097400

TTGAAAGTAACAGTCTAACAACGGATCGTGAAGGAGACCAAACTCGCAGAACAGAGAGAGAGTTAGCACTTAAGAGTGCAACAAAGAGGAAGAGAAGTTGAAACATTTTCAGACAGAGGCATGTGGGAATCTGTTGTGTTGACGGTAGCTGCCACCGCCGGCAACAACATCGGAAAGATCCTTCAGAAGAAGGGCACTATCATTCTTCCACCTCTCTCTTTCAAACTCAAGGCATGTCTAAAATTCTACTTTTTGTGTTTTTTTTTTTTAAATCTGTATGATTTTGTTTTCACTAAAGATCTCTCCTTTGTTTATTATATTTTCATTTGAAACATGTGGGTGTGTATGAAATTTGTAGTCATTATTAGTTTCTATAGCTGTGTTTATTTGGTGTCAATTTGAAATTGGACATTTTCTATGCATGTGTATGATTTGAGTGAGAATATGAGGATGGAGTAATACCAAGATCACTTTTTTTTTTTTTGCATTTAAGGTTATGTGGGCATGAAGTGACTTAGTGATTATAGATCTCATTGTGTTGGCAGGTCATAAGGTCTTATGCTTTAAACAAAACCTGGGTGGTAGGTTTTCTAATGGATATATTAGGGGCATTATTGATGTTAAGGGCATTGTCTCTGGCTCCAGTAAGTGCTATATCCTAATCCTATGTTGTGAAGTAAAAAAAATGGCACTCAATTTGAAGGGTATTCTTAACCCTTTGTATGCTCTTCTAGTGTTTTGCTTAGGTTTTTGACATTGCTATGGTGGCTTTCTTATGTCAAATGTATTATAGGTGTCTGTCATCCAACCAGTTTCTGGCTGTGGACTAGCAATTCTTTCAATCTTTTCTCATTTTTATCTCAAGGAAGTCATGAATGCTGTTGATTGGGTTGGCATTACATTAGCAGGTTTTGGCACAATAGGTAAAGTAAGTTTTTATTTACCATAGAGCATTTTGTTTACCTGATTCTGCTTCCTTTAACACCATGGGGAGCAAGGTTGTTGGGGGAGTGGGAGGGGGGAGAGTAATTATTAGAGGAAAAAAAAGTGTGTTTAAATCAGAAAAAAATATGTAATTTTTTTATAATTCATTTTGGTGATTATGTTTTAGTGATGGATTTTCTAGCCTTTTGTATAGTGCAGAATTCAGATCTACAGATAATGCAAGTAACTGTTGCCAATTTATTTGTTTGTACACTATTGTATTACACTCCAGTGTCTGAGGACCTGAGCAATGAACCAAATTTTTCTGGAGTTTTCCTCTTTTTTATTATTTGGCTGCTGAGAAAACCAAGTTTGTGATGAAATGTCATTCTACTAACATATTTTATTTTGTATCTATGTGATGCTAACTGGTTAAATATTATATGTTTTCTATGTTTAGGAGTTGGTGCTGGAGGTGAGGAGCAAGAGGTGGTTGCTCTATCTATTTTTCACATTCCAGGGCTGGCATTTGTTGTTTTCATCTTGTTTGTAGGTACCTTGTCTTACAAATCAACTTATTTTATAGTCAAGTTGCCTTTATCAATTGATGAGATATATTTAGCATTCATAAGTTTGGCATCATGGCAAGATGTTTGTATCACTTTATTTTGATATAGTAACAAGTTTGTGAGGAAATACTTGAAATCAATGTGAAAGAAGACATAGTGGTATTCTAGTATTAATTGGAAGCCTGTGCATTGTGTGGCAAACAAACATTTCCTTGTGTTTGAGTCTAGTGTGTATAAATGACAAATGTCATGCTTAATAGTGGCATATAATCCATGCATGAAGTTGTATAAATTTCTAAAATCACATTTCTTATTTGTACGAGAAAAACCTTGTCTGATTAATTGATAATCTGTTTTTACCATTATTTTCATGAGATATTATTTTTCCATCACTCTGGTGTATTTAAAATGCAAACTGATGTCACCCTTAATGATGACATATAATCAATGTGTAAAGTTATACATGTCTTTAAAATTAAATTATTATTTGATTATGTGGTAATTAATTTTTCCTAGTCATTTTGTTAAAGTTATCCCTTGGTTCTTGAATCTATGCATATCATTCATCACTAATTTCCTTTTCAAGTCAATATGAAGGTGTTTTGCTGGTCTCCTATCACTTAAGGCATTTACTGGTAGTATTATTAGAAGACTATTGGTTACAATACTTATGGTATTTTTGTATAGGCTTTAAGACTTCTGTATGATTGATGGAGTTCACCAATTGCTAATGCAATATATTAGTGTTACTATATTCCAGACATTGTTTATATTTTGAGAAAATAGGCTATGCACTGACAGTGTAAAATAGTTTTACATTGTCATCCAATCACAACCTACCATGTATGGTAATTTTGTCGACTTTTATGATAATTATCCCTAAAAGTCATATCAATGGTGATCTATGATTGGTTAACATTGTAAAATTGTTTTACACTGTCAGTGCATGACCATTAAACTCTTATATTTTAATTGTTTGAAACTCTTGTACAGATACTTCTTAGTGGATGGCTTCGAATATGCAAGTGCCAACGAAGAGAACAAGAGATGGTATTCATGTTATATCCATGCTGTAACACTTTAGATTTACATGCTTATTTTCATTGAAAACACCCATGCATGGTGATTTAACGAGATATGTTGTTTTTTGTTCTTCTATCACTTGACATTTTTAAGTTATACAGGTGGAATATGATGTTGTTGAGGAAGTCATTTATGGCTTGGAATCTGGAATTTTGTTTGGGTACTTTTGCTTCCATCTCCACTCTTTTTATGTATTCTAATTGTGTTCAGATATATCTCAATTTTTCATGTACCTCAACTCCTTAATATCTGGATGAAGATTCTGATAATTAAATTAACAGCTGTCTGCTACAAGATAATTAATCATTGCATGGTTTGGAGTTTAGATATATTATTTTCTCGTGTATTTTAGTTTCTTTTTTCGGATAAAAAAAGGTTTCAGAGCGCAAAGAGAATAAGGCAGATAGGAGGATAAGTTATTATCATTTGTAGGGGATTGCTAACCTCTACCCACTGGTGTTTGACATTTTCACATTTTATTTTGGACTAAAAACTTTTTTTTTTTTATCGTAAAAAAGCTTTAGATTATGGACAATAGTGTCCTTATTTTTTGTTACCCCAACCACATTTTTTCTTTTGTGTCTTCCATTAAGTTTCAAATAATTCAAATTTTAAAGGCTGCCACAGTCACCATGCCCATAGTTATTTCCAAACAGAGATGCAACCAGTTCAGGCAACTTAACATGGCATCCAATTCAGCCAACTGGACATGGCAAATGAAGCCTTTGAAAAAATGTTACCAGAAACTTCTTTGGTGTTAATCATCATCATCTTAGCCGATCATTGCTTTCTATTAGCTTGTGATTTTAACATTTTTTCTTGGAGGCTAAACCGTAACTAAATTCTATTGGCAATGAACAAAACTCGCGTATTGCAAATATTAGTTGCATGCCAGAGAACAGAACATGATTGTACATCACTCTTAATTTTGTTTACACCTAAATGCAGTATGTCATCTGTAATATCGAAGATGGGATTTCTATTCCTAGAGCAAGGTTTTCCCAAGCTGTTGGTTCCTATGTGCATCATGATTAGTGTGTGTTGTAGTGGCACTGGCTTTTACTACCAGGTATTCTGAAGCTCACTTTTACATGTCTACTATTATGAGAACTATTACACCCTTTAAGCATGGAGGGCCATTTAAGCTGTTTCTGTCAAACTGCCTAGATACTTGTCCAAGTTCTACGCTTCCAATTTCAGAGACGATAACCTCTTTAAATAATCATATTATCTTCCCTGCTCCTTCAGACACGCGGTCTAAAGCATGGAAGGGCTATTGTAGTTTCCACATGTGCCGCTGTGGCATCAATTTTGACTGGTGTTCTTGCTGGGATGCTTGCTTTGGGTGAACGACTTCCTTCGGAACCAAAAGCTCGCTTGGCACTTCTTCTTGGATGGTAATATAAGATGTTATGATTTGTGTTTTACAGCTTTTTTTTTTCTTTTGAACAAAGGAAAAATCTAAATTAAACTTCAACCCTTTCATTTTTTCTCTTCACACATAGTAAACATGCACACATAGAAAAATGAAAGAAGACTCAGGAGTAGCAAGGTATTTGTGCCTTCTGGAACAGGATGCAAGTTGAACTTTTAGATTGATAGAGTCCATAATATTTATGGAGAAATGAGAAATATCCATCCAAGAAAATCTCTATTTGGATCCAATTTCAAGGTACAAGACTTGAGTCCCACATGGCAAGTATGGAATTTATATAAGGCCTTAGGCTCTCCTACTACTATGACTAGTTTGTATGGTGTGATTCATTTAAGGTTTTTATAAATTTGTATCAAGTCTTTTCATCATGTTTTGCACTTGTAAATAAGCAGGGCGTGGTAGAATGTTTGGATCCAATTGCAAAGTATGGAACTTGAGTCTTGGATTGGAAGTATGGAATTCTGGTGTGGGATTTATAAGGCCTTGGGCTCTCCAATTACAACTGCTAGCTTTTGTGGTGTAATTTTCCTAGGGTTCTTATCAAACTCTTAACCATGTCAGAAATCAAGAACGAAAACTGTTGGTTCTCATCTACGTTGATATTACCAATGGATTGGAAGTTAAAAGATTTCACACCCTTTGCTTTCCAAGGTTCCTTGCATCAAACAATGCAGAAACAAACAGCAGCAGTTCAATACTTCTTTCCATCTGGAAGAAGCTGCATCGTATTTAGTTGTCTGTTACAAACATTTGGTGGCTTTCATCTAACCTGTTGCACCAGTCTTTATATTGGTTGATGAATATCTTTAACTTGAGAACAATAATTGAAAATATAGATTGAGACTATTTTGGAAACACCGTATCTAGTCATTCTTGTTTCTCAAACAAAATGGAAATCTTCATGTTCTTATACATATAAAACTTTATGCTGCAGGCTACTTATTATAGTTGGTGTGATTTTACTTGTTGGTTCAACACGGCTAGTGAGATTCCTTTCTTGTTCTTCACAGCGAAAAAGAAGCAATGTGGATAAGAATTTTGACCTTAGAAGAGCCACTTCTTCCCGTGTGAGGGAAACAAGTCCAAGTGCTGTCATTCAAGCAGCAACATTAAATCATTTACTATCATCATCTTCCAAAGAAAAAGCTTGAGCTGGCATGAGCTGGATCATGTTCCTTCATCCTTCCCAGGATTTCTAAGCATTATTAAGTTGCTTTCTAATCCATCTGCCAAGGCCAGGGAGTATTCTGTATAGCATAGAGGCTGCTTCATCAGAGGGCATGGCATTTTGGTGATCATTCATTGCTTCTGGCTCACATTTTGGATGAAATATTTGTTAACTTCTGATGCAACTTGTAAAAACTATTCATGAACTTACACTACTAGTGAGTGTACCCGTGCACTGCACGGGTTGATAAAGAAATAAATTTTTTGTTAAAATGAATTAATTTGTATAAAAAAATAGACCTTTAATATCCACATTATAAGATTCTATCCATG

>GLYMA.15G125900

CCTCTCTCAGCATAGAATAGACAAATGAAGTCCGACTAAATTAAATGCATCCAATCAAGTGACTTTGGACATGAAACCTCAAAAAAGTTAAAAAAAAAACAAAGGAAGAAGAATAGATGCTCCAATTCAAATGATCTGCTTATAATTGGTATAATTGGTTGGAGAGTCGTAAATTATTGTAAATTTTCTGAAATTATCTTCGATTCCTCCTATTAAAAAAAAAAAAAAGAATAGATGCTTGAGCACTTAACAAGTTAAATTAGAGTCAAATAAAGCAACAAAGTGCTTCGATTGATTCTATAACGTGAAAATAACGAATTTATGAGTAGTAGTAGATAGTGTAGTGTGTTTGTTTATATATCGCAATTCGCACATCATTGTCAGGGTATCGATTCGCTCGGGTTGTGCGCGGGCTGCGTGTTTTTTCTTTTATTAATTTATTAATATAAGGATTGGATTTGGGATTGAAGTTGAAAGACACCACTCTCCTCTGCCTCTGGTAATTTCCTTTTTATTCCTCAAATTGTTTCTTATTTACGGTTTCCTTGTTCAGTTTCTTTGGTTGTTATATACATAAAATTTTATTTGTTGTTTGAGTTTTGATTTGAACTATATTTTTTTTCCAATGCTTAAGCACGGTTGGTTTCACAATGCAAATGATTACTAAGTATTCAATTCTTTTAGGATCGGTGAATGGTAATGATATCAATGTCCGAGTCTTCTGAAATCTGAATTAATAGAATTTAGTTGTGGTCTTTATGATATTAATAAACCTGCTATGCGCTTTTATATATGCACCTTAGATATACATGCTGCTGAATTGAATTCTAACTTGGAAAGTATCCGAGTGATAACTGACATCTTGCATCGTTACAATTATGCTTATTGTTGAGTTCTTAGAGCCATTAGGAACACTGGAAAAAAAAACCCAGTGAGCCAACCTCATTTGAAGGCTTGTTATAATTATTCATCACCCAAACTTGTTTTCTCTTTTAAACAAGAAAATAGATTGCATGACAGGTTTATTGCTTTTGATGCGGTTTGATGATTGATTGGTTAGATTTAGACCATTATTGAAAGACTTTCTTTCATAATCTCTTACTTTAACTAACTACCACAGCAAAATGAATGAACAGTGAGGTTTAAATTATTTTCATTCACCTTTTCTGCCTTTTTTTTTTTTTAATCTTTTTTATTTCAGTAAGAATATCTGTACTGTCCTATTGTAAGATTAATACCTTTTCCCTTCAAATTGTAGCATTGATTTGTGATAACAGATAAGTCAGGGAAGGTTGATGTTGATTGTCAAATCGTGTCCTGAAATCCGTAGTTTTTCAAATGCCATAGCAGTGTAGCTTTCCTCATATAAGAGTGGCTTTTCTGGTGAAGAGCTAACTTAAAAGCCCTATGTTGGCTTCATTAAGAGCAGAAAAATGAGTGATACAGAAGGTAAATCACATTCCTCGGGAATCACGAGGAAACTAGATAGCCATAAAACTTATCACGGTAGAGATCCCAATCACGGAAATAACCTTTGGAAAGATGGACTTATTTGTGCTTTTGAATACATTAAAGGACAAAACAGATCGGTTAAATCGAGCTCCTCATCAAAGATCACAGACAGACTACATGTTAATGGCCAACATTCAAAGATGCATGTCCCTTCAGATGACAAAAAGAAGCTCTCAGATCCTTCATCTGTAAATGTCTCAAGGGACAGTTTGTTTGGTGGCTCAGATGACGACAAGGAGGGCCAGGCCCATAAGGCTGGGCAATCTAAAAAGTATGAGGGAGGTCATTGGGTACCAATTGGATGGGCAAGAATTTCAGAACTTGTCCAAGCAGTTCAGGTTGATGCTGACTGGTCTTCTCATCAATTGGAATTTGAGGATTCTGAAGATGATTTTACAGTAGCAGATTTGGCAGCTCCTTATTGGGAGCATCCGGCTGGGCCTATATGGTGGTGCCATGTTTTTGCAGGTCACCCCACTGTTGAGGCTTGGCTCAGCAATGCTCAATGGCTACACCCTGCTGTTAGTTTAGCCTTGAGAGACGAAAGTAGGCTTATAAGTGAGCGAATGAAACACCTTCTCTATGAGGTACGTTGCATTTTAAACTATTGTTATTGTCAGTCTTAAGTCTTAACAAATATTATCATTTGGCAATCTAGATTTGGATATATGTTTGCTAAAACACATTTACATGCACCACATCAAGCATACATATGAGAGTGTGTGTATATGCAATAAGATTTTAAATTAAAATTTTCAATTGTATTATGTTTTTGTTGATGGCATAAATGCTTGTAGGATTGTGGCTTCTTTGTGATGTATTCACCCTGATGATAAGCAACTAAAAAAGCAAGTGACTTTTTGGATTTTTCTTCTCTTCCTTAGTTTGCTGGTCTTAAGAGAAAAACATCTATAGGTTTGGAATTTAACAGATTTGGATTCGAGTTTTCTAGGATAAATTAAGCGTGGAAGGAAAACCAATGGAAATTGGATTTCAGTTGATGGGTTTTAGTACAAACTAGAACAAAGTCCGTGTGGGCAGCACAACAGAGACCAACAAATCTCAAGTTGTTGTCGTTAGTTTTTCAGGAAAATCATTAAGAAATCATTGAGTGGGAAAACACAGCATCAAGAGCTTGACACCTAGCACTAAGTTGGATAATTTTAATATAGCAAGAAACTTTTCGGGATGTGATATGGTGATTTTTTGAGGAGAAAAATAAATTAAGAAGACAAAAAAAATATGAGAAAAATACAAAAATAAACCTAGGATTCCTTAGGAATTAAACCTTGGGCTTGTTTGAGTTAGTGCCACACAACCAGTAGATAGCAGTTCATCTGAAAGAAAGCAATACAGCTATTTAAGTAGACATCCAATGACTTGTACCGCCTCCCAAAGAAAAGAAAACCTTCAATGACATACCCCACAATGAATTTTCCTCAGAAACCAATCTAACTCGATATTGATTGGACTTCTTACTTGTAGGTCCCAGTCAGAGTTGCAGGAGGGCTGTTATTTGAGCTCTTGGGACAATCCGCAGGTGATCCTCTTGTTGAAGAAGATGACATTCCAATTGTTCTTAGGTCTTGGCAATCTCAAAACTTCCTTGTAACTGTAATGCATATAAAAGGATCAGTATCAAGGATAAATGTTCTGGGTATAACAGAAGTTCAGGTTGTTCTGTCTCTCTGGCCTCAAGTTACACAATAATATTCTTTTAGAGAGATGTAATTCATTCAGTGCTTCACTATGGTTAGGAGCTTCTTTCTGCTGGAGGGTATAATATGCCGAGAACAGTGCATGAAGTTATAGCACTACTTGCTTGTCGTCTCTCACGGTGGGATGATAGGTAATGACAGCATTTTCCTTCATATTTGATGCTGGTAATAGTTAATTTTGGAAAAGAAATAGTCAAAAGTAGGTTAGGTCTTGCTTCTAGTTATATAACTTCAGAATTTAGATGTCTGAGGGTGAGTTAAGTTTGTAAATATTAAGACATTGCATGTTCTGAAAGAAAAAGCTATCAAGCATATGAAAAATATGATTGACTTTGTTGCTAGAAAATGAAAGGAATATATTTTGTCTATCACTTCTATCTCTGTTATGCACGAAAGACAGGAAAAATGTGGATTGTCAGGTTAGGTTTTGTTAAGTTCTGCTGTTAAATTTTATATTTTAGATTTATTGACTTTTACCACCTCGTACTAGATTATATGGGCATGTTGTTTCCTTAGGAGTGTCCAGTAGATAAGTAGCTTGCGAAGATGGCCAATGTACCTCATGCTTGCAGATCTTTGTACACTTTCTTTTTCAACTGCATTTTATGTAAACTTCAAGTCATGTTCGGATTGACATTTTTATTTAATTTGTCAGGTTATTCCGTAAATCTATATTTGGGGCGGCAGATGAGATTGAATTGAAGTTTATGAACAGGTTCTTAAATGTGCAATCTGTGTAACTTCAAGTCATTCCAGTTTTATAAATTGTCTTGCACTAAGTTGACAGTTTTCCCTTTTATTAGGAGAAACCATGAAGATTTGAATCTTTTCATCTTAATCTTAAATCAAGAAATCAGAAAGTTATCAACACAGGTATATTCATTCTTGCTAAGCAGAAGATATATCTTCAGACAACTTCATGATTTTTTGATTTGGATAGAAATGTGATTATTTTAACTCAGTAATAGGGGTTAAAATGAGGGTTGAGGTTTTCAAATGGTTGGATGTTTGCAGTTTACAGGTTGATCTTTCCGTTGCAGGTTAAGTAGTGCTTGTAACCTATAAAATTATACTTGATATTAGATTATGTAGAGTATACTGGATCTGTATGTCTAAACTAGACGACACTAGGGTATATTTATACTGGTGAATATCGATTACAGCAATAACTGCAAGTACTTATCTCCTGAAAGTTCCGCAATTGGAGAAATAATTTACTTTATCATATATATTAGTACCTAGGTTTTCTATTTTTCTAACAAGGACAATGTTAACAAGTGCTCTTATAGCACTGGTTAAGGAATTAAAAGTTAAAACTCTTCACGGAAACATGTTCCCAACACTCTCCCCCCATGATGATTTCAATGCAATCTCACCGTGATTTCTTGCAAAAACTTTCTATTAATTGATTTCTTGACCAATGCTCGGCACTCATTAGCAGAGTCAGGACCTTTCTAAGAATTATTTTGGAAGAGATTAGGAATCATTTTAGACATTTGAATTTAGTTTTGTCGTTACATCTCTTATATAAAGTGAGCAAGGGAAAGAGAGATGTGAGTTGCAGATCTGTTTGCTGTAGTTGTTAGTAAACTAAATTTTAATTTCCAGAGATGTGCATGTGCAGCTGGTCTAAAGTTATCAAGCTTCAAAAGGCTGGAAATGAACCAAACAAGCTTGAGAGTAGTGTGAAACTTGCCTTGTTAATTAGCCAAATGAACTTAATTCATAGACCAATTAAGCTGAACTCAGGACAAATACTGCTATACTCTTTTGGTTCATGAACTCTCTCTCACACACACTTTGTATATATAGTATTTCATATTTACGATAGTATAATTGATTAGTTAGTTGAATTGAGTCAAGTTTATGAGCTATGGCTTTTGTTCATTTGGTTCATATTGTCTCATTTTTTATGTAATTCAAACTGATAAACTGAGCCAAAGTATTAGAGAAGTTGATTTGAGCCAAATTTGAGCTAAAAAAATGGTTCATATCATACTCAAGTTTAGTTTCATGTGGAACTATTTCTTATATAGCCAAGCTGAACTGAGCCCTTTTCAAACCCGAGCCCACTTGTCTCTGAACTTCATGTAAAACAACTAACATAATACAAACATATGATTTCTTTTAGTTGTCAACAATGTCAATGAAATTTAATAGTGCTTTTTGTTCTACAAACATGGAAAGATACATTTTAATAATAGTGACATTCCATAACATATTTATTTGTGTAATTTTAATCTTTCACTTTTGAAAATAGCTTCGAAAAGCACAAGCAATACCGAAGTGAACCAAATTCTCCACCTTAGATTGATTTTATTTGTGTAATTTTAATCTTTCACTTTTGAAAATAGCTTCGAAAAGCACAAGCAATACCAAAGTGAACCAAATTCTCCACCTTAGATTGATTTTATTTTTTTAATGACAATTCATACTGGCCAAATCCATTATACAGTAGCTTCTTACTACTCTTGCTTCATTGCAGGTTATCAGAGTGAAGTGGTCACTCCATGCAAGAGATGAGATTGTCTTTGAGCTTCTCCAGCATCTAAAAGGAAATGGAGCAAGAACCTTGTTAGAGGGAATAAAAAAGAGCACAAGAGAAATGATTGAGGAGCAAGAAGCTGTTCGTGGCCGCCTGTTTACCATTCAAGATGTTATGCAAAGCACTGTTCGAGCTTGGTTGCAGGTATCAATACTTCAGGACTTTCTTTAGAATGCTTTTTTCTTTTTTACTTTCAGACAGTAGCTAGAACAACTTTATATTGCTCACCCTAGATCAACATGATAATTGTCTTGAATCCGCTGAATCTCTATCCAGGATAGAAGCCTTCGAGTAACCCATAATTTAGCTGTATTTGGTGGTGTTGGCGTTGTCCTCACCATCATTACTGGGTTATTTGGGATCAACGTTGATGGGATACCTGGGGCAGAACAGACACCTTATGCATTTGGTGTTTTCACGGCCATCCTCGTCGTTCTGGGAGTAGTGCTGATAGCAGTTGGCATGGTTTACCTTGGGCTGAAAAACCCCGTTGTTGAGGAACAGGTTGAAGTAAGGAAACTTGAGCTGCAAGAATTGGTGAAGATGTTTCAGCATGAAGCAGAAACTCATGCTCAAATGCGGAAAAATATTTCTCCCAAGAACTTACCTCCTACTGCTGGTGATGCTTTCCGTAGTGATGCGGATTATCTTGTAATACAGTAGAAATACTATCATACTAATACTTTAATTGTCCAAGGATGCTTTAACATCAATTACTTTTGGTGTTAACTGTTGTTCCAAGTCTGAAGTTAAAAGCATGAATTGAAAAAGCATTTCTCTTTGCTTATTCATGAACTTTGGTGAATGTGGATTGTGGAGGAACTCACAACCGTGGTTGGATGGATGGGGGACGTCAGTGATGATAAGCTCATACTTTCTTTTGGACATTCGTAGGCCTCAGTCTCTAACAAAGCCGAAATTCTTCTAGGCTTTGCTCTATACAAGTTCTTCATTGAATAAATTAGAAGTTTTATATGAAATGAATATTTGTATGCCTCGTATGCAAATTGTTCATTTAGCTGAATATTTGTATGCCTTGTATGCAATTGTTCATTTAGCTGAATTTATAAACGTCATGTCAACTTAGTGTATATAGAAGATTTTTTTTTTTTTTTTTTTTTGTAATAACAAAGTGAAAGATGTGATACTACTCATACTGTGATACTACTCATACTAGTGAGACTTTAGACATTCTAGTTCTACTAAGGATTTGTATCGTTTTGCTGCCTTTGTTTTTTTAGTAAAATAAGATTACC

>GLYMA.16G003900

TTTTTCTTTTGACGAGATAGTGTGATATTCATAATAATCGTACGATAGATAAATAAAGGAGAAAGAAAAAGGAGGTTTGAGAGACTAGAAGAGGATTGGGATTTGGCAAGAGATTTAGATTAGCATGGAATCAAGAATGGAGTGTAGTAGTCATCAACAACCTCAGTCACTGTAAATTTTTTTGGTTATTATTTATCTCTCTCAAATGCATATGCAATCGCAATCAATTGCAAAGCATTTCGGATCCAGAGGGAGAGACCCTTTGTGACAGCCTGTATCCTCTCCATCGTCTGATAGATTCTTCATTTTTTTTCCATTTCTCGCGGCAAGATCTCACACTCTGCACCTAGGGTTAGGGTTTTGGTTCTTGCGGCATGGCGACTTCTTCGAGTTCTTCGAGCTGGCGCGAGGGCATGTCCTCCGACAACATAAAGGGACTGTGCCTCGCTCTCTCTTCTAGCTTCTTCATCGGCGCCAGCTTCATTGTCAAAAAGAAGGGTTTGAAGAAGGCCGGTGCTAGTGGAATCAGGGCCGGTATCGTTCATTCTTCGCCACCGTCGATCACTTTTTTGTTTTTACTTTGTATTCAGTTCATTTTCCGTGTTTGCTTTAATTTTTCTAGGGATCTGGACGTTTTTTCTTTTACTTCTCTTGGCCACGAGTTTTTCAACTGTCTCAAATCTCTCAAGTTTACACTCGATGCGTTGCTTTGCCGGCCTTTTTTTTTCTCTGTTATATCTTCTCGTCGCATTCCACAAGACAAATAGTTGTAGAATAAGCATATTAGGTCTCGGCATGAGGCGGAAGGGAACATTTTTGTGTTCTCGATTCTCATCCTTAAGATTTTCACCGAACTGTTTTTTTGGCAATTTTAACTATCCTTTTCTTGTGCTCCTTGGTATGGAATGATGAGAGATCTATACAGTTTGTGTGACTTGAGATCTTGCTTTAAATGTTAGAAATTTGGTGGATAAACTGTCTGCTAGTTTAATTTTTTCTTTTTGTAGCAACTCATCAGTAGGGAAATCTTTTCTTTGATTTATTATAAATTAGATGGATGCTTAACTCTAATGATTCGGCCTCTGATTGGCTTGTTTACTTATATGGGTCTCTTTTCTTTAATTTATTTTATTTAATCTACAAAATAGTTGTTGCCTAATGTTAATAGTTTTTATTTTTTGATTCATTGTCTCCCTGATTGATCATGTGCAATAGTGACTAACCATCCAAAATTATATCTGACAGGAAGTGGAGGTTATTCTTACTTGTATGAGCCGCTTTGGTGGGTGGGAATGATAACAAGTGAGTTATACACGATTCTGTTTGGTTTTGGAATGACTAATGAATTGAATGTATCGTGGATTTTTCCTTTTCCAAGTGCATCCCTTGTGCTCCTGCCTCTTTTTTTTTATATAAATATTTTATTTAGTTTATGGTTATTTTACAAGTGCAGAAAGTAATGTAGTTACTATACTTCTGTAGACTATAGATTGAGTTCTATGTTGAACCTTAACAAATCCAAAGAGGGGCATTCATGTGTATGTTCTCCCCTTCATTCCTGTATAATCCATCTTTCTGCTACTTATATCCAAATTTATGCATTGCACAAAATCAAAATAAAAAATAAGAAAAAGGTTTCTAAAGCTTATACCATCCTAACCTTTTCTTTTCTTTCCTTTTTGCTTCACACATTTTCCAAGGGAATACTTCAGCATGGACACAAAAAAGGGCGTATAAAGATGCTAACAAACTTTCCACATACAATTTATTGATATGAGTTGCGTATAATATTTAAGTGGATTAAAATTTCTATTCTTATTTCTAACACATTTGCAATTTCAACAGTGATTGTTGGAGAGATTGCCAATTTTGCAGCTTATGCATTTGCCCCAGCTATATTGGTCACCCCTCTTGGTGCTCTTAGCATTATTATCAGGCATGATAAAAAAATTTAGCTTTAAATTGATGCTTGACTCTTGGTAAATTTTGATTTTTGACGTTTGCTTGCAGTGCTGCTCTTGCTCATATTATTTTACGGGAGAGGCTACATATTTTTGGAATTCTCGGTTGTGTTTTGTGTGTTGTGGGATCTACGACAATTGTTTTGCATGCACCTCAAGAACGGGAAATTGAATCTGTTTCAGAAGTGTGGGATCTTGCTATGGAACCAGGTAGTCATGTTCCTACTTGATATATCAGTGTCTGTAATCAATAGGTATTGTATTGAACAACTAACCAACAACATAATCTTTTTTCTGCAGCATTTCTCTTTTATGCAGCTATGGTTATAACAGCTACTTTTATCCTTATCTTCCACTTCATTCCTCTCTATGGCCAGACACACATAATGGTTTATATTGGTGTTTGTTCCCTTGTAGGTTCTCTAACGGTATGTCTCGTTATGTAGTTGTTGATGACTTTCTAATAGTATTTGTTCTCCCCTTGTTATTTTCCCCCCTGTATTTGTATTGTAAGGTATTCCTTTGACAGGTTATGAGTGTTAAGGCTCTTGGAATTGTCATAAAGTTAACACTGTCTGGGATGAATCAGCTAATTTACCCTCAGACTTGGGCATTCACTCTAGTTGTACTTGTTTGTGTTCTTACCCAAATGAATTATTTAAATAAGGTACAGTAGTTTTTCTTGTTAGTTCCCTGAACTTTGTTTTATTTGATGCACTAGTATGCACTTGTTTTTGTAAACATCCGACAAGTTTTGTAATGTACTTCACTACTTTTGGTCAACAAGTATCATGTAACATGAAAATAGTTTGATTGAATTTCCATTACCATTCAGTGAAATCTGTGAAAGTATCATGTATTTATGCTCACAACTTTTTTAAATGCATTTTTTTTTTATTTTGAACATTTTATGTATGTAATCTTTTCTTGCTTGTGTTGAGAAGAATATAAAATTGTCCAAGTGCTGGTTACTTATCTTCTTGTCAGACTTATGCATGATATGTTTCTTGAACCATAATTCTTTTTATTGCTCAAATCTTTTAACTTCAAACATATATTACCATCACATATGACCTTGTGATCTTTAAGTAAACTACCGGGATTGATTTAGAATTTTATCCCTTGGTTTAAAGATAAAGAACTGATTTGGTCATAATGTCATATCCACTCTTTAACGAATAAATAGTATCAATCAAATATGTAAAATGTTTCTTTTTACATAATAAAAGACAGCCCCCAAAACTTGATAGGAAAATTGACCGCTAAATGAGGAAGAAGATCAACAAGGAAAACAGAAGTTTGACTCTCGTTTGTCAGTTGGCCAAGAATTCCCCATCTTTTAATTTTTTTGGGAAATCCCTCAAACTAAGTTCTAAGTTACATCTATGTGGCATGCAGATTGTTGGAAATTCAAAATTGCTCTTCATTTGAAGTGAATCCACATTTGTGCAGGTTGAGTAGTTTTGGCCCAACACAAGAAACTGTGGCTTTTATGAGTGAGAATATAACATGTATCTAACTTTGTTTCAGGAGAAATATAATAAAAATGAGTGGTTGATTTTAAAGTAACTTTATTTTGGGGTGGGGCGCATAAGATGGAATTTTTTTATTAGTACAAAGTTAAAGTGAGGAAAACATTATTCTGGAAAGTAGAAATGATGAATACCGTATCTATTACTCATTGTGGTGAAGATATTTTGCTGTTCTGCTATAAAGATGTTTTTCTGTAATTTTGCATGTTTGGCATTATTATTTAGAGCAAATTACATTCGCACCCGCCTGAGTTTATCTCAAATTACACATAACACTACTCTTTTTATTCCTACCCTAATCCCCCTTTAAGTTTGAAAACATTACATTAATACTCCCTTATGTTTGAAAACATTACACTAACACCCTTGGACCCTCTCATGTTACACTAACACCCTTTACAAGGAAGGTGACTGTAATGGTTAAAAAAGCAGGGGGTGTTTGTGTAATTACAAGAAATCTCAGGGCTGGTTAGTGTAATTTACTCTATTATTTATGATAGAGTGCAACAATCTCTTAGCCCATGATTTTTCTTGAATCAGTAAATTAGTCAATTGAAATTGATATGTTAATCGATATTCAAATGCCTTGCAGGTGGGTGTTTGGTAGGGGAATTTTTTTTTAAGTTTCCCGTGGGAGGGATTGGAAATCCAAGATTCCTATGAAGGGTGAAAGTTTTTTGTATAATTATGTCTTTATATTTATGATATTCCCAGGAAATATAATTCCTTGGAGACATGATTTTCGGGGATGAAAAAAGACTTTTTGGGTCATATTCCTAATTTATTGAAAATCACAAATTTGTGGGTCATATTCCTAATTTATTGAAAATCACAAATTTGTGGGTCATATTCCTTATTTAATAAGCCCCCCACTCGAAATTTTGTAGTTTCCAATACATTTTAATCAATAATAAAAGAATGTGTTGAAATAATTGTGTTGCTAGCACTCCTCTTAAATCTAAATGAACATAAAGGCAACTTTGGACATAGTTGTCAAACTTGAATTAGCTCTTAGACTACTACGATTTTACTGGTCTAGGTAGAGAAAACAAGTTAACTCACTATCAAACACTTAAATGGATAAACTCAGGTGAATTTGTTAAACTCGTGTAAATTCGCATAAAGTTGAGTCGAGAAGTGAGTCATTGAGTTTGAAATCAAATGTAATTTTTGCCCCTCATTGACCACTCAGTGATAGTGTTTAACCAAGGCTCAAATGATTTGAAGGAATTAATTCTTTTTTTTGAATGTTTTCACTTTAGTTACTTATGATTGTATGAATTTATGTATGATTTGGTCATTTAAATGGTTTGATCTTTTGTAGTACTGTACTATCATACTTTATTATTGTTATTTGTTACTTACTTACTTTGCTTTATTATGAAGTTGAAATGTTGATTTATTGTTGTGTTAAAAGTTTAATGTTGAGCAAACATGCCATTAATTGGTTTTTTAATATTATTTTTTATATTACGTAACACATTTACGAGTATACGAATCGAGTCTACAAATGTTCCCCGAGTTTATGTAGACTCTCATGTTTGACAAACTCGGGTTTAGGTATCTGGGTGGTTTATTATGAGAACAAGTTCATTTTCTCTATGGCTTCAAACTTGAATTTGCAGGATTAGGATAAAACTATATGTTTCCTTGGTGCTGGATGTCGATTTGTCATGATTCTGCTACTTACTAATATTATGCATTTACTTTTTACATATTGTCACCTCCATTTATATTTTTGGGATTGATGAAAAGTTCCTTCTTTTTTATAATTCATGTGATAATATATTTTTTTCATTTTTGCAATCAGGCACTGGATACTTTTAATACGGCAGTGGTATCTCCTATATATTATGTTATGTTCACAACATTTACCATTGTGGCTAGTGTTATTATGTTTAAGGTAAGTTTTGTTACCTAACTGGTATAGTTAGACACTGCCTGCTTAATTGCTTTAATTTTAAGATTGTATATTGTATAATCTGTGTTTGTCAAGGAAATGCTTGATATAATTTTGTTTTGGTGTTTCCACAAAATCTTTTCAATGATACTTGTATGCTGGTTGTTGCCTATAACTATGTCAACCTATTTTTATTTGCTAGTCAAGATTCTCTTGGTAGGTCCTGCATATACCTCTGTAATGAAGTGTGAGAAGTGAATGTTTTTGTGAATAAACCAGTGTCTGCTGAGACATTTACATCTAGATGTGAAAACTGTTTGCGAGTACATTCAATAGGGTGTGAAACTTCTTTTGCTCTTTCCCTTAACAACCATATGGAGATGCAAATCTTTAATTTTCGTCTCACTATGTCTTGAATCTGATGTGCAAATCATCAGCTTAATGGTTACTTGCACATTCAATGTCTGTCCCCTCTTTGCCATATTTTGAAGGTTGACCATTGATTGATAATTGAATTTGATGCCAGGACTGGGATAGACAAAGTCCAACACAAGTTATCACAGAAATATGTGGGTTTGTGACCATTCTATCAGGAACTTTTCTTCTTCACAAAACTAAGGATATGGCTGATGGTATGATCTAGTGATCCTACTATAGTAGCAATTTCATGTAACTTTAGATTACACTCTCGTGCTCACATGCACTTTTCGTGTAGGGCCTTGAAGTCTTCAGATATGACTTTGTTAATATATCTTCATGTGCAGGTTTACAAACATCTTTATCTATTAGACTTCCTAAGCATTCAGAAGAGGATGGCTTTGATGGTGGTGAAGGCATTCCTCTTAGACGGCAAGAATCCATGAGATTGCCATGATATATTCTTTGCATCACATCTTGCATTTCTAAATGAGCTTGAGGCGCTGGGGAGGGATTTTGCCAAG

>GLYMA.16G149500

CTCATTCTCTCTCCTCTTTTCCTTGCACTCTCATCTTTTGCTTCTCGAATCTATGACAAATCCTCCGTTCATTTTGGACCAAGAAAAGAAAAGTCTCACACTCCTTCTTATTTTTTCTTCTTCATAATAATATTAATAATACCGTTTTTTGTTTTTATCCACAATTTCTGTTTTTATAGTTTTTTTTCAAAACCCTTTTTCATAGTATTTCCCTTCCCTCTGAATCTACCTTTCTTTCTTTATACAACCAACTCACGCAAGCGTCCAACCTCGCAAATTTCGATTTTGCTGTAGCAATAGAAGAGGGGAATTGATTGATTGTCTCTCAGGTAATCCAGTACCTTTTTCAAGCTTTCACGGTTGTTCCCATTTGACCCTTCCCCATACAATGCATTTTGGGTTTTAATTTCCCTTCTTTGTTTTTTTTTTTTTTGCAATTTGTAGTTGTATCGGTCTTGAGGGTTCAATTTCCTGGCAAGCATAGCAGATCTGGGTTCTTCAAGGTTAGAAAGCTGACTCTCTCTTTATTTTTTTTCCTTTGATTGTTGTTTTATTTTGTCTTAATTAAATGTTGAAAGATCAGAAGACTGGAAGAGTTACATTGTGCTGTTTATTCCCCCCAATAGATTGGATTTTGTGACCCCTTATTAAAATTATCTTAAAAAACATGATTTTTAGTGCACAAATTAGTCTTACTTTTTATGGTTTGAATTTGAGCCAATTGGGTTTGTGTTTTCATATTGAATTTTTTCTATTTCTTGCTATACCCCATTTATCAGCTTCATTAATTTTACTCATAGATCTCCAAAGTTTTAGTAGTTAATAACATTGTTGAAGGTGTGAACTTTGATTTGATGGGTTTGATTTGTGTTTGGTGGTGACACAGGAGTATTGCTGTCCTGACACTTGGTGTGTAGAAGAAGGGCTATCTTCCTTTCTACCTGTGTTTTGCGTTGTGTACAAGAGTTTGAGAACTAACATCTGATAGACGGCCATTGGAATTTTCTTCTGCTGCATGTGGTGTCTCCTTTGCAGTGCCACATCATTTCATTCATGGGGGAGTGGATTGTTGGAGCTTTCATCAACCTCTTTGGTAGTATTGCAATAAACTTTGGGACCAATCTTCTCAAATTAGGGCATAATGAGGTATGCCAAATTCTTTTTTCTGGTTGGAAGTTGGAACCGTTTGTGAAGTACATTTTTATATATATGTTTCTCTCTCTTTTTTTGTAATGTGGTTCAAAAATTGGCTTTGCTGTTGTATCCCTTTTCAGATTTCAGCTGACATGATAAATCTTGACTTAACTAATGGGTTAATGTATAATTTTTGAACTCGTCTGTATCTTGGTACATAATTGCTTTGGATATTGTTTGCATGGTTTTTTGGAATTTGGATTAGTGACTTGTGCTATTAAATTGAAGCACTGAAACTTCCTTATACATATTACATCTCAATGTCAATATTAACTTGCAGTCTATACTATTTCTTGAAATTTGCAACTTCTAGTATCAAATTATTTGATAAAACTGAACAGGTTAGGCTTCTTTTTTTCTTTTTTCTTGCAGAGAGAAAGACATTTACTTGGAAGTGATGGGGTAAATGGAAAGATGAATCTGAAGCCTATTATATACTTCCAAAGTTGGAGAATTGGTATATTCTAATTGCATTTATATATGGGTTTTGCACATTTTATCTAGGTGGCTGTTTCCTGATTTTTTCTTTCATTGCATAATTCTTGATATGCAGGCATTGTATTTTTCTTTCTTGGAAATTGCCTTAATTTCATTTCCTTTGGGTATGCTGCTCAGGTATGTTTCCTGCAATTTGATTGGAATTTTATGTTCAATGATTTATGGTGTAATTTTATGATAATATGTAGTAGTTTTGCAGTTTTTTTTTTGTTTGGTTGGTATATATTTAGTGAAGTATGCTATTTCTTATGATTGATTTGATGGATGTATTTTCATTTGTTCTGATTACTTATTTTTCTTTGTGCTGTGCAGTCACTTCTTGCAGCACTAGGATCTGTTCAGTTTGTATCTAACATTGCCTTCGCTTACTTTGTCTTGAACAAAATGGTGACAGTAAAGTATGCTAAATTTGAACTGCGATTACTGTTTCATATTTGTTTGTTTTCACTAACTCTGTTCTAGCTGTTAATACTTGTAGCTTAATTGATGGTCTTTTATCAGGGTACTGGTTGCAACAGCTTTCATTGTTCTTGGGAATGTTTTTCTAGTCGCTTTTGGCAATCACCAATCACCTGGTATGTAGCATTTTGCCCAGTTCACTTAAATCTTTGCTTCTAGTCTTCCTTGCAGAAACAATGTTAATTAAGCTGATAGATGTGCATCAGTACAGTATCAAGTATTATCTGCAATCAAATGACTAAACTGCTAATTTGGTGAACTGCAACTTAAGTAGCTAGGCTTTTATATGTTAGAGATAGGAGGGAGGATTTTGTAATGCCTCTGCTATAAACTTAATTTGATTTATTGTTTAATATGTTCTTTATGCTTACTGAATATTATGATTCATTGCTGTTAAACAGTTCAATCATTGATTTCAATTTTGCAAATGCAAACTAATATGTGGTTAACTACCTGTGTTTCAGTTTATACGCCAGAGCAGTTGACAGAGAAATATACCAATATTGCATTCCTTCTATACCTTCTAGCTTTGATCTCTATTGTTGCCTTGCATCACTCCATCTACAAGTAATTAGTAGGATTTAATTATTTGAGTCTAATCAAACTATTTTTCTTCTTTATGATTAATATGTGGAATATGCAGGCGGGGAGAACTTCTGTTTGCAGTATCAGGACATGACCTCAGACCCTATTGGAGCATGCTACTGCCCTTTTCATATGCTGTAGTTTCGGGGGCTGTAGGTTCATGCTCAGTCTTGTTTGCTAAATCGCTGTAAGTCAGCTACTTCAACATGTTGCCATGCCTCATGATACAATAGCCATTGTGCCTAATGTCAACTTTTTGGCAGTTCTAACCTATTACGACTGGCTATGTCCAATGGTTATCAGTTGCACAGCTGGTTCACATATTCCATGCTTCTTTTATTTCTTAGTACTGCTGGATTTTGGGTAAGGCTTTTTCTTTGGTAAAGATTCTTTGTTGGGCTTTATCAATTTGTGCTGATGTTTTGATGGGGTAACTTTTTGAAGTTAATTTATGACAAATGTGAATGCTAACCCGGTCTTTTCCAATTGTGCAGATGACCAGGTTGAATGAAGGACTGTCATTGTTTGATGCAATTCTTATTGTTCCCATGTTTCAGATAACATGGACTTTCTTCTCAATCTGTACAGGATTTATCTATTTTCAAGAATATCAGGTACTTTCTCTCTCACTTAATATGCACAGGTAGCCTTTGCTTTGCATGGGCACTCTAAGAAGATTTAATCATATAATATTGAAGAAAACTAATTAAATACTCCTACTTTGATGGAATGGAGTCACTTGATTATATATTTCAGAAAATCAAATTTGTTACATAATTTAGAAACACCATGGGGCATGGGTTCAACATACAAAACCAGACCTAGCCTAGATAATGTAAGTCAAACTCAGATAAATGACTTAATCTTGCTTATTCTTCTGCTGGACCTGGCTAAAAAACTTGGAAAAAGGATTTGACACCCCATTCCACTACTGGTCCTGTCAAATCAGGGATGGGTTTTGTAATCAGGGGGGGATTAGCATATAGATATTGTGTAGTTGGGTGCCAAGCTTACAGTCAGATCTATGGTTACTTAATTCTTGCTGTATAGCTTGTATTAGGACACCATAGTTCTGCATAATATGGTGATAGGTTGTATATACAACAGTACCCCTTGTACTGATATTATTACTATATATATTATAATCTTACTTTTGCTGATAAAAAAAAATGTACATTTCCCATGATACCGAGATGTGGTATATTGTAGTGTAACTGCTTGTTAGGTTCATTCTCTGGTGACGTCTATCAACTGGTTTTTCTGCAAGGTCTTAACCTACTGAAATTTTCCATTCTGGTTATAAAAGTTTCTCTCTCAAACCCAAAATTAATCTTCTGTTTTTACCAGTGGAGCTTTGAGCCATGGGTCATTGCTTACTGCTGGGGAACAATGTGAACTTCTAAGTTCTAATATTGCGGCAGAGAATGCAGAAAATCTAAAATTAAATAAAATAGAAATTAATGAGGAGGATATTGCAAGGTAGTTAAATTCGAGATTTTATTTAGGATTGGAAAGGGAGTTAAGATCACCAATCATGGGATCTTAACACTGATTGTAAGATCCTATAAAATTCATACATTCATACACACAGATAATAGCATAAACAAGCAAAGAGGACATTTCAGTTTAAGTTCAGCAACTTCAAATCAGAAACACTGAAAAATAGATGCCAGGGAGCCAGATACAGAAGAGAATTAGAGAACAGAGAACCCAGCAGTCAGCAAAAGACAGATGTAAAAGAGAACCACCCAGCAAACTCAGCAAACATAAGTGCAGAAGAGAAGAGGAACAATGAAGGAGAAGAAGATAGCATGCAGCAGTGGTTTCAGCTTTAGAATGACTGAAAATGGTGGTAAATTTTGCCAATTTCAGATTCAGAACTACCTGGATATTTCATGTTGCAAAATAGGTTGCAGGTCAAAAACCTCACATTGGACTATATTTCAGGTGTGGTGAGGTTGCAAGATCCCAGCAATCCTATGCGAACTTGCCCTGTGATCTCAGCCAATCCTACCAAATTGCGACTGAGTGGGATCATGGTTGCTCATGGCAGTGAAGATTATAAAAATCATACAATATGGAAAATAAAAATTGTAAAATAGGAATACAATCTTAGTACCATGGGGATAAGATGATGAACTATATTCCGATCAACAGATTAAGCCCATTGTAAAATAGTGTAAAGTCTAAACTACTAAGTTAGCCAATGAGTAAAAGATGCGAGAAACACAAACTGTGACAAACATTTGTCAAATATACGAAGTGTATAAAACTGAAAGATACTCAAAATAGGCCAAATGGTAAAAATAAGAGCATGCATCTTTTTATTTCCATTGCTGGTAAAAAAGAATCTCATTTACTGTACTACAGACAAACAATACCCTAGAAGAGTTGTCCTTGAGAGCTATATATATTGTCACAATAAGATTGAAAATATGCATAAAAGTTGCAGTAGCATATAATCAGCCAGAGATATGCATACACAAGGAAATGGAAGGGATACAAGTAAGCTGCCTCTGTAAACAAACCCGTATTCTACATTCTTGACTTCTTGAATGGTATCCCTAGGGCTGGGTGCTGGATCAAATCAAATTCACCAGGAAGGAAATTTCCCTTGCTTTCTTGCTGTCATTTGCACAAAAATGTCTAAGCAGCTTTCTAGAGATATGAAGCTGCATATCTAGTGGGAGCTGTCCAGCACATCTCCAAATTTGTAATCACAATCTAGCATAGCAATTTAGTAGTGTAACTCTCTTGTCCAAGATGAAGGAATTTATAGACCAATTAAAGAGCAAAGGCATTTTAGATTTAGAAATTCCTTGTAAGACCATTTGTCTCCATCCAATAAATTTAAAATGTTCAATATGAACAAAATACAAAATGTTCAAACAGAAAATAGAAATATTGTAGAGCATAACAAAATTGATAGAAACTGAACTACATAGGATGACAAAATAAATGTATATTTCAAAATTCAAAATATGCCAAGTGTCTAACTGAAGGAGACTAATGTTAGATAGAGGAGAATAGCTGGGATGAAATGACTCTACTCTCAAGAAAATATCTCATCTTTCACATATAGGGATTGATTAACTAGGATCATAGATTTGAAAATTTTGGGCAAGTGCAAAAATATGGATTGTTGCTGAACCATGCCCGATGAATCCAACATTGTTTCATTCTTTTATTATCAGATTCAGTGTGTTATTTGGTTTCAATGATGCCCTTGTTTTGCCTCACCTACCTTTCCCCTAAACTCAACTTTGATTTGGACAGTGCTAGTTTTTTATTATAATTTTTTGAATATGTTAGAACCAGGTGCTTTTATTATTTTTACTTTTTCTTTTTAATTTAATAGGGGTAAAAATTGTAAATATAAATTTGACTATTTTCTTGAAATTTCTACCAAATGGTGAGATATGCAGCTAATAATTGCTGCGCTTGCCTTCATTAATTTATTTGATCTTTTTGTCTATAGTAAGTAGACAGTCATCCAGCACCTTCTAGAATCTAGAAGTAAATGTTTCTCAGGCTTTGATGCTTGTATCTCTAGTATTTTTTGTTTTATTTTTGTATGGTGTGAATGTCTGAATCATTACATTGATGAATGCTGAATGTGTTTTGATAATGAGTTTAGGGGTGATGTAATACTACCTTCCTGAACGCAGGTATTTGATGCATTAAGGACAACGATGTTTATACTTGGAATGATGTGTGTGTTTATTGGCATTTCTTTGCTGGCACCTGATGAATCAAAAGGTACTATAATTTGATGATTATTTTATGGTTTTATAATTCAAGGTAAGCATTGCAGAGGATAATACGAAACTTTGTTTTGAGAAGTTTCAGGTCCTGAGACTAAAGATAGTTCTTTGGATTCCATGGTGTCTTCTGCCATGTCAACAGAAACTAGCAGGTACTGTATGAACCCTTTGTATGACTTACATAAAGTGAAGAACTTGTAGCCACTATAGTTTACTATATGAAACATCACTGAAGGGAATCAAAATGATTGAAGTACTAGTTCATTGTATCATCTCATTTTAACTTTTGCTTTGACCTTCTAAACCAGCCATAGTTCTCTTAAATTTTGTTGACAGAATTATATATGACTGTATTTTCTTGTATTTCTTTTCCTTTATTTCTTCATGAGTGTTACAGTAACATACACATGTTAATGTTGGCGACTTTCACTGATGCCTATACTGTTTATCTATGATAGGCTGGTAGTGTCTCCCGAAGAAGCACAAAACAAAGACTCAAGATCATTTGTCAAAGCAATACTAATAAAGGTTACAGATTTGTTGGTAAAGGCAAAGGTATTATTCCTAGTTGCATTAACATTCTTTGCGAATGGTGCCACTTGTTATGATGATAAATTGACAATTCAAGCTTGCTTCTTTCTATCTCTTTGCAGACTTCTTGTGCATTGTCTCTTGGTTTTGGGGAGGATACCATCAACACATCATCGGTTCTTGTGATGCCAATGATGTCATCAAGAATGACTGGATTCAGAGGAAATGGGCTTGAAAGAGCAAGAATATTGTCCATGAGAAATGGTTGGAGCAAGATTCCAATGGATGAAGATGCTGGCAAATTGCTTGAAACTAGTTCAGTTGTTCCTCCTAGCCCTTAGCAGTGGTTGTTAGTGTCATAGGATGTCCCACGGTATCATTTTTTACCCCTTGATTTCCCATTTTTTTTAATGATTGTTGGAGTAGCTGAGATATAAAACCGTCATAATTCGATTTATTTGCTTAGCATTCATGTAATTGGGAAAAAGAGGATAAAAAAATTGTTGTATATTGTATATACATATTGTAAATTTTTGCTTGCATAATTGAAATTTAAAAGGAGAAGAAAATTCGATCT

>GLYMA.17G227100

CGAGTATCCGAAGTTCTTCATTTCTAATTATGAAATTTAGTATTTAAGCTCATCAAATCCAACCACTAGTTTATATACTAATTGATATTAGTGTTAATATATTACACTAATAAGAAATAATTGGTAGCTTCTTAGGTAGCATAACAATTGAAACTTGAAAGTAACAGTCTAACAACAGATCGTTAAGGAGACCAAACCAACAGAACAGAGTAACAGAGAGTTAGCATTGCAACAAAGAGAAAGAAAAGTTGAAACATTTTCAGACAGTGGCATGTGGGAATCTATTCTGTTAACGGTGGCTGCCACCGCTGGCAACAACATCGGAAAGATCCTTCAGAAGAAGGGCACTATCATTCTTCCACCTCTCTCTTTCAAACTCAAGGCATGTCTAAAGTTCTATTTTTAAAATTTGATTTTGTTTTCACTAAAGATCTCACCTTTTTTGGTAATATTTTCATTTGAAACATGTGGGTGTCTATGAATTTTGTAGTCATTATTACTTTCTATAGCTGTGTTTATTTGGTATCAATGTGAAATTGGACATTTTCTCCTTTTTTGGTAATATTTTCATTTGAAACATGTGGGTGTGTATGAAATTTGTAGTCGTTATTAGTTTCTATAACTGTGTTTATTTGGTGTCAATGTGAAATTGGACATTTTCTATGCATGTGTATGATTTGAGTGAGAATATGAGGATGGAATAAATACCAAGATCACTTTTTTTTTTTTTTTTGTATTTAAGGTTATGCGGGTATGAAGTGACTATGTGATTATTGATCTCATTGTATTTGCAGGTCATAAGGTCTTATGCTTTAAACAAAACCTGGGTGGTAGGTTTTCTAATAGATATATTTGGGGCATTATTGATGTTAAGGGCATTGTCTCTGGCTCCAGTAAGTGCTATATGTTATGAAGTGAAAAAAAATCGCACTCAATTTGAAGGGATTTCTTAACCCTTAGCATGTTCTTCTAGTGTTTTGCTTAGGTATTTGACGTTGTTATGGTGGCTTTCTTATGTCTAATGTATAATAGGTCTCTGTCATCCAACCAGTTTCTGGCTGTGGACTAGCAATTCTTTCAATCTTTTCTCATTTTTATCTCAAGGAAGTCATGAATGCTGTTGATTGGGTTGGCATTACATTAGCAGGTTTTGGCACAATAGGTAAAGTAAGTTTTTATTTGCCGTAGAGCATTTTGTTTACCTGATTCTGCTTCCTTTAACACCACGGGGAGCAAGGTTGTCGGGGGAGAGGAAGGGGGGAAGTGTAATTATTAGAAGAAGAAAAAAGCATGTTAAATCAGAAAAAAGATGTAATTTTTTTATAATTCATTTTTATGATTATGTTTTAGTGATGAATCTTCTAGCTTTTCGTATAGTTCAGAATTAGATCTACATATAATACAAGTAGCTGTGATCAATTTATTTGTTTGTATACTGTTGTATTACACTCCAGTGTCTGAGGACCTTAGCAATGAACCAATTTTTTTCTGGAGTATTCCTCTTTTTTATTATTTGGCTGCTGACAAAACCAAGTTTGCAATGAAAGGTTATTCTACTAACATATTTTATATTGTATCTGGGTGATGCTAACTGGTTAAATATTATATGTTTTCTATGTTTAGGAGTTGGTGCTGGAGGTGAGGAGCAAGAGGTGGTTGCTCTATCTATTTTTCACATACCAGGGCTGGCATTTATTGTTTTCATCTTGTTTGTAGGTACCTTGTCTTACAAATCAACTTATTTTATAGTCAAGTTGCCTTTATGAATTGATCAGAGATATTTAGCATTCATAAGTTTGGCATCATGGCAAGATGTTTGTATCACTTTATTTTTGAGATATAGTGACAAGTTTGAGAGGAAATACTTGAAATCAATGTGAAAAAAGACATAGTGGTATTCTAGTATTAATTGGAAGCCTATGCATGGTGTGGCTGCCAAACGTATATTTGGCTTTGAGTCTAGTGTGTATAAATGCCAAATGTCATGCTTAATAGTGGCTTATAATTCATGCATGAAGTTGTATAAGTTTCTAAAATCACATTTCTTATTTGTCCAAGAAAAACCTCGTCTGATTCATTGATAATCTGGTTTTACCATTATTTTCATGAAATGTTATTTTCCCATCTATCTAGTGCATTTAAAATGCAAACTGATGTCACCCTTAATGATTACATATAATCAATGTGTAAAGTTATACGTGTCTTTAAAATTAAATTATTCTTTGATTGTGTAGTAATCAATTTTTCCTAGTCATTTTGTTAAAGTTATCCCTTGGTTCTTGAATCTATGCATATCATTCATCACTAACTTCCTTTTCAAGTCAATATGAAGGCATTTTGCTGGTCTCTTATCACTTAAGGCATTTTGCTGGTAGCATTATTAGAAGACTATTGGTTACAATACTTACTGATGGTATTTTCGTATAGGCTTTAAGACTTCTCTGTGAGTGATTGAGTTCACCAATTGCCAGTGCAATATACTATATTCCAAACATTGTTTATATTTTGAGAAAATAGGCTATGCACTAACAGACAGTGTAAAACAGTTTTATACTGTCATCCAATCACAACCAACCATGTATGGTAAGTTTGTTGACTTTTATGATAACTAGCCCTAAAAGTCATATCAATGGTGATTGGTGATTGGTTACACTGTTGGTACATATGATCATTAAACTCTATATTTTAATTGTTTGAAACTCTTGTGCAGATACTTCTTAGTGGATGGCTTCGAATATGCAAGCGCCAACGAAGAGAACAAGAGATGGTATTCATGTTATATCCATGCTGTAACACTTTAGATTTACATGCTTATTTTAATTGAGAACACCCATGCATGGTGATTTAATGAGCTGTGTTGTTTTCTGTACTTCTATCACTTGACTTTTTTAAGTTATACAGATGGAATATGACGTCGTTGAGGAAGTCATCTATGGCTTTGAATCTGGTATTTTGTTTGGGTACTTTTGCTTCCCTCTCCACTTTTTTTATGTATTCTATTCATAATCAGATACATCTAATTTTTCATATACAGCAACTCCTTAATATCAGGATGAAGATTCTGACAGTTAAATTAACAGTTGTCTGCTACAAGATAATTAATCATTGCATGGTTTGGAGTTTAGTAATATTATTTCTCGTGTGTTCTGGTAGTTTCTTTTTTTGGATAAAAAATGGTTCTCGGAGTGAAAAGAGAATAACAACATTAGGAGGATAAGTTATTATCATATGTAGGGGACTGCTAACCTCTACACACTTGTGTTTGACATTTTCACATTTTATTTTGGACTAAAACTTAATTTTTTTATTGTTAAAAAAATTTTAATTATGGGCAATAGTGTCCTTATTTTTTGCTACCCCAACCACATTTTTTCTTTCATGTCTTCCATTTAGTTTCAAATAATTTAAAGGCTGCCACAGTCACCATGCCATATTATATTTCCAAACAGAGATGCAACCAGTTCAGGCAACTTAACGTGGCAACCAATTCAGCCAACTGGACATGGCAAATGAAGCCTTTAAAAAAATATTACCAGCAACTTCTTTGGCATTAATCGTCATCATCATCTTAGTTTTGACTATTTTACTGTAGCTACTAATGCAGTTATGCCAAGTCTGAAGCCATTCATATAGCCTATCATTGCTTTCTATTAGCTTATGATTTTAACATTTTTTCTTGGAGGCTAAACCGTAACTAAATTCTATTGGCAATGAACAAAACTTGCATATTACAAATATTACTTGCATGACAGAGAACAGAACATGATTATACATCACTTTTAGTTTTGTTTACACCTAAATGCAGGATGTCATCTGTAATATCAAAGATGGGATTTCTATTCCTAGAGCAAGGCTTTCCCAAGCTGTTGGTTCCTATGTGCATCATGATCAGCGTGTGTTGTAGTGGCACAGGCATTTACTACCAGGTATTCTGAAGCTCACTTTTATATGTCTACTATTATGAGAACTATTACACCCTTTAAGCATGGAGAGCATTTAACCTGTTTCTGTCAAACTGCCTAGATACTTGTCCATGCTCTACGCTTCCAATTTAAGAGACAATAACCCCTTTAAATAATCATTATCTTCCCTGCTCCTTCAGACACGTGGTCTAAAGCATGGGAGGGCTATTGTAGTTTCCACATGTGCAGCTGTGGCCTCAATTTTGACTGGTGTTCTTGCTGGGATGCTTGCTTTGGGTGAACGACTTCCTTCGGAGCCAAAAGCTCGCTTGGCACTTCTTCTTGGATGGTAATATAAGATGTTATGTTTTGTGTTTTACAGTTTTGTTTTGTTTTTTGTTTTAACAAAGGAAAAAATCCAAATTAAACTTCAACCCTTCTGTTTTTTCTCTTCACACATAATAAACATGCATGCATACAAAAATGAAAGAAGACTCAGGAGTGGCAAGGTATTCATGGCCTTCTGGAACAGAAGACAAGTTGAACTTTTAGATTTATAGAGTCCATCATTCATGGAGAATTGAGAAGTATCCATCTGAGAAAATCTCTATTTGGATCCAATTTCAAGGTATGGGTCTAGAGTCCCACGGAGCTAGTATGGGATTTTGGCATGGGATTTGTAAGGCCTTGGACTCTCCAACAATAATGACTAGTTTGTATGGTGTTATACTCTTAAGTTTCTTATCAATTGGTACCAAAGCTTTTGATCATGTTTTGCACTTGCAAATAAGTGGAATGTTAGGATCCAATTGCAAAGTATGGAACTTGAGTCTCGCATTGGAAGTATGTTCTCTGGTGTGGGATTTGTAAGGCCTTGGGCTCTTCAACTACAACAAGCTTTGGTGGTGTGATTCTCCCAAGGTTCTTATCAATCTGTTCCCCATTTCAGAAATCAAGAATGATAACTGTTGGTTTTCATCTACATTGATCTTACCAATGGATTGGAAGTTAAAAGATTTCACATTCATTGCTTTCCAAGGTTCCTTACAACAAACAATGCAGAAACAACAGCTTTAACTAGAGAATCATAATTGAAAATACTTATTTCCCTCTGGAAGAAGCTACATCATGTTTAGTTGCCTGTTACAAACATTTGGTGGCTTTCATCTAATTTGTTGTGGTAGTCTTTATTTTAACTAGAGAATCATAATTGAAAATATAGATTGAGACTATTTTGGAAAAGACCCTGTCTAGTAATTCTTTGTTTCTCAAACAAAATGAAAATCTTCATTTTCTTATACATATGAAAACATTATGCTGCAGGCTACTTATTATTGTTGGTGTGATTTTACTTGTTGGTTCTACACGGTTAGTAAGATTCCTTTCTTGTTCTTCACGACAAAAAAGAAGCAATGTGGAGAAGAATTTTGGCCTTAGAGGAGCCACTTCTTCCCGTGTGAGAGAACCAAGTCCAAGTGCTGTCATTCAAGCAGCAACATTAAATCATTTACTATCATCATCTTCCAAAGAAAAAGCTTGAGTTGACATGAGCTGGATAATGTTCCTTCATCCCCAGGATTTCTAAGCATTAAGTTGCTTTCTAATCCGTCTGCCAAGGCCAATTCTGTATATTATAGTGGCGCTTCATCAGAGGGCATGACATTTTGGTGATCATTCATTGCTTCTGGCTCACACATTTTGGATGAAATGTTTGTTAACTTCAGATGCCACCTTGTAAAAACTATTCATGAACTTACACTATTTTTGCTTTCAAGGATTGTATATGACAGTGATGATAGCATACCAACATACAAAATTTGTTTTACTTGCACTTAAACAAATTGGTAAACAGTTTATTCGTATGCAGTTGGGATCTTTGCCTATAGCTGTGGCTTTTGTT

>GLYMA.18G091200

ATGATTATGCTTCTTTCCATTTTTGCCACGACACAAGAACATAATTTAACGACTAACCAAGAACATAATTTTTCTACTGCAGCATTTCTGTTTTATGCAGCTTTGGTAATAACAGTTACTTTTATCCTTATCTTCCACTTCATTCCTCTCTATGGCCAGACACACATAATGGTTTATATCGGTGTTTATTCCCTTATAGGTTCTATAACGGTATGTCTCATTATGTAGTCATTGATGACTTTCTAATAGTATTTGTATCCCCTTGTTATTCCCCCCTGTATTTGTATTGTAAGGTTTTCCTTTGACAGGTTATGAGTGTTAAGGCTCTTGGAATTGTCATAAAGTTAACAATGTCTGGGATGAATCAGCTAATTTACCCTCAAACTTGGGCATTCTCTCTAGTTGTAATTGTTTGTGTTCTTACCCAAATGAATTATTTAAATAAGGTACAGTAGTTTTCCTTGGCCAATCCAAACTATTTCTTGTTAGACTCTCGAGTTTGACAAACTTGAGTTTAGGTATTTGGATGGTTTATTATGTGAACAAGTTCATTTTCTCTTTGGCTTTAAACTTGGATATGCTGGATTAGGATAAAACTATATGTTTAGTTGGTGTTGGATGTTCATTTGTCATGATTATGCTACTTACTAGTATTATGCAATTACTTTTACATATTGTCACCATTTTTTTGGGGGGTGGGGGGGAGGGTTGATGAATAGTTTTTTCTGCTTTATAATTCATGTGATAATATTTTTTTTCATTTCTACAATCAGGCAGTGGATACTTTTAATGCGGCAGTGGTATCTCCCATATATTATGTTATGTTCACAGCATTTACCATTGTGGCTAGTGTTATTATGTTTAAGGTGAGTTTTGTTACCTAACTAGTATAGTTAGACACTTCATTACTCTAATTTTAAGATTGTATAAATTGTGTTAAAATAATTCGGATTGATTGTATATTGTATAATCTGTGTTTGTTAAGGCAATGCTTAATATCATTTTGTTTTGGTGTTGCCACAAGATCTTCAATGATACTTGTATGCTGCTTGTTGCCAATATTTATCTAGTACCTAATTTTATTTGCTAGTCAAAGTATCATGTATTTATGCAAATGAACTGCATATACCTGTGTAATGAACTGCTTGCAAATGAAGTGTGAGAGGTGAATGCTTTTATGAAAATAGTTTGATTACCATTCAGTGAAATCTATCAAAGTATCATGTAGTTATGCTCACAATTTTTTTAAATGTATTTTGATTTTTAAACATTTTATGTAAGTAATCTTTTCTTGCTGGTGTTGAGAAGAATATAAAATTGTTTGTTCTAGCATTTAAGAACTAAGTGATCTTTATTTTATTTTTATCTGATAAACACTCGGTGATCCTTATATCAACTTTTAATCAATGTCCTTGTTTGTTCAGGGATTTATTGCATATGCCCTTAATTATGGACTCATTACATGGTGA

>GLYMA.20G210300

ATTTCTTCACCCTCGCCGTTGCCTGCGCCTCCCTCTTATAAACCTCACATAATTAAATTCACATTAAAGAGAGACACAAACAAACTTAGTAACTCTGTTGGTTTTTTCCCTTTTCCGGGAAAGGTTGATAGAAAGGAAAGAAGAAGAATAAGTTCCTTTTTGTCGTATCGTATGTTTGTTTCTTCTGGGAATCAGATTCCTCAGCGTGGTGTCGGAACAAAAAAAAAAATATTTTGAGGCGAGACTCGGATACCCAATTGGCGTATTGATGAAATGCGTGAAGGGGGTGGGGAAAATTTTGCTTCTGGTGTTGTGGGTGTGAATCGAGTTTGAAGTTGAATTGTGGGTTGGTTGGGGCATGTGATTGGAATTGGGGAGAGGGAAAGGGTAAGGGGATGGCTCGGGGGGATGGGAGCGTCGTCCCTACGGACCCGCAGACAATGGCGGTTGTGAAGAAGAAGACGCAGTCTTCGAGGAGTTGGATTCTGTTTGACGCCACAGGGCAAGGCTCCTTGCTCGACGTCGACAAATATGCCATCATGCATAGGGTTCATATTCATGCGCGTGATCTCAGAATCCTTGATCCCTTGCTCTCTTACCCCTCCACCATTCTCGGTCGTGAGAAGGCCATTGTTCTTAACTTGGAGGTATTGGTATATTTTTTTTCCCTATTGTTTTTTATTTTATTTTTGTTTTTTTAATTTTGTTTTGCTTCTTCTGATTCTGTGTTTCAGCATATTAAGGCAATTATCACCGCTGAAGAGGTTAGTTAGTCACCTTTTTATCCTTAAATATGTTACGTGTCTGCTTCCATCTCTGAAGGAGCGGGATTAGAAACATTATGGCTTCCTATTTCTGGGAAAGAAAGTGAATAAGAACATCATGAAATTAGTATGTGGACACAAGCTTTGAATTTTTATTTTTGGCTATTCATAATTGAAAAGCTTAAACTGATTGAGAAATTAGGATTAGTGGAATGGTGGTAGATGGGGCGTTCTCTTAGCATATTATCCTACTATTGCATTAAATGGCACGACTAGTCTTAGGATGGATTGAATTTCGGCTCTGGAATTTTGTTTTCATTGTCAAGTTTATTGAATAAAATGGGTAGGAAGACAATGAACTACATGTTGGATCAGGACCATAACCGTCTTGTACAAGAATATACCTACATGATTTTGTTTCTGACTTTAGTTATGATGCCTGGCAAAGGACATATTTTCATATAACTAGAATATTGTAGAGGCAGCTTCAGTAGGCTAATCAACTATGCTCTCTGACCTAGTTTGTATATTTGTGAATTCCTGTATCCAACCTTTCGTCCTTTGTAGAATGCAAAATTTTTTCTAGAGATTGAGATGGCATTTGCTTCCACATCCTGAATTCCTGATTGTATCTAAATTGTGGGATAGGTATTGCTGAGAGATCCAACAGATGAAAATGTGATCCCTGTTGTTGAGGAACTGCAAAGGCGGTTGCCTCAATTGAGTGCCACCGGTCTTCAACAGCAAGGAGATGGTAAAGAGTATCTTGGTGGCCAAAATGATGCTGAAGCCGCTGAAGAAGATGGTACGTTACATTAAATGATGAACCATAAGTAATGAACAAACTTATTGATTGCATCTGTTCCTGTTATAAATCTATCTTTGTATTTGCATTATCTCTTGCTCTTGTTATCTGAGTGATCTAAATTACACAAATGTAGAGTCACCCTTTGAATTCCGGGCCCTGGAGGTTGCTTTAGAAGCCATTTGTAGTTTTCTTGCTGCACGTACAACAGAATTGGAGATGGCTGCTTATCCTGCATTAGATGAACTTACTTCCAAGGTACATATATTTTTTGATTATTTCTCTTCTTGTTTTGGTTTTAATCTGAGATCCCCTGGCCCATACCCTTTCCTCAGGTACAGAATAAGCTAACAAATAAGAAGCTTCTTAAAAGTGACTGAGGGAAAACTTCTTTCCTTTATCTCAATATATTATCCTTTTGATTTTTTATACTGATCATCACTGAGGTGTTTATTCTAATTATGTCAAGAAGGTTTTTGTTGGCAGGCTTGAAATGTGGATGCACTAGTTCCATGTTGCTCTAGTTTCCTTTCTTCCTTTTATTTATTGACTTTTTTGGTTCAGTCATGAACTTTAAAATTATTGTAATTGTAATCTGCAGATTAGTAGTCGTAATTTGGACAGAGTTCGTAAACTGAAGAGTGCAATGACAAGGCTGACTGCTAGGGTTCAAAAGGTATGTCAAGTTTTACTATGCCAGGGAAAAATTTGCACATGGAAAAAAACTATTTTCTTCTATGTAAATATATTCTCCGCCCTCTGCCTCTCAATGCTGTTGATATTTTGGACATCCTTACACTTTGAAGGGTTATTCATTTTTTATGCTGCATAAATGCATGAGAAAAAAAATATGCCTTTACATAGTTTGCCAGAGTTTGAAAAATTGGAATGCGTATTTTCAAGATCAAAAGAGAATCAATTTCAAGAAAGATTCACACTGGTGTAAGTGGGTCCCTGCCTTTTTAAAAAAAAATGTTCTTCCGAGAATGACTTATGGGCATTCTCTTTATTGTAATTATTTTATTTTTCTTCTTAATAGAAGTTTAATGTGAATGTAAACTGCCTTGATCAGTTTTAAATAGAAAACAACATATATCCATTTTCCCTCTCCCATCACAAGTTCTTGTTATCTGGTACAGGTCAGAGATGAGCTTGAACAATTGCTGGATGATGATGATGATATGGCTGACCTGTACCTGTCAAGAAAGGCTGGTTCAGCATCACCAGTTAGTGGATCAGGTGCTGCAAATTGGTTTGCTGCCTCCCCCACCATAGGATCAAAGATATCTAGAGCAAGTAGAGCAAGTTTAGCAACAGTTCGTTTAGATGAAAATGATGTGGAAGAGCTTGAAATGTTACTTGAGGTTATTAACATTATTTGCTTTAAAGAATAGAAGGAAAAGATCTTCATTTTTTTATGAGGAAAAAATTGCTATAAATGGTGTTTGTTCATTTTGCAGGCTTATTTCAGTGAAATCGACCACACATTGAACAAATTAACCACAGTAAGTATCATCTCTTTGTGTATGTGGACACATGTGCAATAGGGAGCTGTAAATCTTAATTTTTGGATATGGCAGATACATGCCAGATATAAAGTATTTTGGAAGATTGTTTCTTTGCAGATATGGAGAAATTTTTTTTCTCTCATTAATTTAGAACCTATGATCTGTTTACTAATCTAATTATTAGACAGTGAATTCAAGGCCTAGCCTTTTGGGTAGAGACTTTTAGTTTCAGACTAGTATGCAAGTTAAAGTTTGACCAATTTGGTACTCTGAATGCTTACAATTGTGAAATTAATGATGAACAAAGGGGATATGCATTTTGCTCTCTTCCTTCTTCCATCCCCTGCTGTTTTAAAGTTAAACTTTGTGGTTTGTATTAAAAATCTTGTTTGTAAGTGTTTTATGCATTTTTTCTTAAAAATTAGTTATAAGAATTTTGTGAGATGATATACTCTTACTTTGTTGGATTCCTTCTCGTCTCAGATTGAGTGATTTATGCTATTTATCCTTTTGGATCAAATAGACCATACTTGAATCTTTGCTGTACCACTTTTGCCTTTGTAATTAAATATGATGAAAATGGTGATTCTATGTCTAATTCAGGCTATCAAATCCCTGAATTTATTTGTGGTGGGATGTGTTAAGAGGATCTACTGATGCCCATTAGATTAGGCTTTTCTAGAAAGGGATTCAAAACTAGTGTACTCTTCTGACATTTGCTTGGCTTCTTTAAAAGTCTGTGTTATTTACTTAGATAGGTTTACTTGAACTCTGAATTTGCTATACTAGATAGTTTTACTTTTACCTAATTACTTTTCTTTTTCTTTTCATTTAAAGCTGCGAGAGTACATTGATGATACTGAAGATTATATTAATATTCAAGTAAGTAGCTAGCAATTTACTGATAATATATCAATTGGTATCTGGTGAATGACATGAAATTCAAGTGAAGTTTTTTCCATATTGCAGCTTGACAACCATCGTAATCAGCTGATTCAGGTGCTTTCACGTTTCTGCACCTTGCATGTTGCATGTCATCTATCAGTATTCTATAATGTATTTTTTTTTTACGAGTTCGCTTAATGGTCTTCAACTCTTGTGCAGTTAGAGCTCTTTCTTAGCTCTGGAACTGTTTGTCTATCTTTCTACTCTTTGGTGGCGGCTATATTTGGCATGAATATCCCATATACTTGGAACGAAAACCATGGTTACATGTTCAAATGGGTATGCAAACTATTACTGTTTATGCATGCACATGTGTGCTCACATATATGTTGCTGCAAAATTTTGGACTTGATATTATGAATAAAGAATAAAAATACTCCATGTTTTCTTGCAGGTAGTTATTGTCTCGGGAGTATTTTCTGCTGTGATGTTTCTCATGATTACAGCCTATGCTCGCAAAAAGGGGTTAGTAGGATCGTGAAAATCAATCTACTGAAAAAGGGGTGCATAGGACTCCTACACATATAACAGTCTAATGAATTATGTCGAAAAGTTTCATATTGGAGATGGGTCAACATCATGTTGAGTTATTGGACATGCTTCAGTAACAATAACTGTTAAAGGGTCTAGTCCTTAAGTTGATACATATTATTGAGGGCTGTTAGCCTTTGGGATCTGCCATTTCCTCAGCTATTTTGTTCTCTGGATTCAGTCATTATAACAGATTGCAAATACAGTCTACAGATTCAACGTTAATTCTTGATTTTGAATACTTTCAAGGCCTTGATATGTAATTAAATTTATAATTAAGTTGAATATGCTAATCTTAACATACTTT
