# Supplementary material for: Genome-wide identification and functional characterization of magnesium transporter (MGT) gene family in soybean (Glycine max L.) and their expression profiles in response to aphid infestation, dehydration, and salt stresses
Source: PLoS One. 2025 Aug 29;20(8):e0330440. doi: 10.1371/journal.pone.0330440 (PMC12396710; doi:10.1371/journal.pone.0330440)
Supplement: S4 Data — (S4 Data.DOCX) [file pone.0330440.s004.docx]

>GLYMA.02G068000

MGEWIVGAFINLFGSIAINFGTNLLKLGHNERERHLLGSDGVNGKMNLKPIIYFQSWRIGIVFFFLGNCLNFISFGYAAQSLLAALGSVQFVSNIAFAYFVLNKMVTVKVLVATAFIVLGNVFLVAFGNHQSPVYTPEQLTEKYTNISFLLYLLALISIVALHHSIYKRGELLLGVSGHDLRPYWSMLLPFSYAVVSGAVGSCSVLFAKSLSNLLRLALSNGYQLHSWFTYSMLLLFLSTAGFWMTRLNEGLSLFDAILIVPMFQIAWTLFSICTGFIYFQEYQVFDALRTTMFMLGMMCVFIGISLLAPDESKVSGPETKDSSLDSMVSSAISTEANRLVVSPEEAQNKDTRSLVKAILIKITDLLVKAKTTCALSLGFGEDTINASSVLVMPMMSSRMTGFRGNGLERARILSMRNGWRKIPMDEDAGKLLETSSVVPPSP

>GLYMA.02G117100

MDETQDHYYSSSLPESSLSHDGGGRSYFNGQINRGTAISGLKKRGHGSRSWIKIGQDGNFQTVTLDKATIMRYCSLPSRDLRLLDPMFIYPSTILGREKAIVVNLEQIRCIITADEVILMNSLDGSVGQYRLELCNRLQNEKADDLPFEFRALELALELTCTSLDAQVNELEMEIYPVLDELASSISTLNLERVRRFKGHLLALTQRVQKVRDEIEHLMDDDGDMAEMCLTEKKRRSDTCTFNDCFQTRASGRLISKSAPASPERTISGVQMLQRAFSSIGNSSKHGSSMGSSDNGERIEPLEMLLEAYFIVIDNTLNTILSLKEYIDDTEDFINIKLGNIQNQLIQFELLLTAATLVAAVFAAVAGVFGMNFETTVFDYPSGFHWVLVITGIACIALYFALLFYFRYKKVLAA

>GLYMA.02G280800

MGLSKENLKGLILALVSSGFIGASFIIKKQGLRRAAAVSGVRAGVGGYYYLLEPLWWVGMITMIVGEVANFVAYAFAPAVLVTPLGALSIIVSAVLADIILKEKLHNLGILGCIMCIAGSIIIVIHAPKEQPITSVLEIWNMATQPAFLAYVGSVIVLVFILVFHFAPRCGHTNVLVFTGICSLMGSLSVMSVKALGTSLKLTFEGKNQLIYPETWFFMLVVAICVIMQMNYLNKALDTFNTAIVSPIYYVMFTTLTILASVIMFKDWDGQSGGTIVSEICGFIIVLSGTIMLHATKDFERSSSFRGSDPLSPTLSARLFTGNGDSLLKQDEENGSPESNMRSRRQELY

>GLYMA.02G285600

MVVEALVPLLESNMQAMNEDYSASFTSKMKKEGNHKTYSSTRDSNNNMQQGGELWTNGLICAFEFMRGNGPTKKKDYCLGRIGNSLNESDSHGDDFHLYCKEDLPRRYWRPIGWDRISELVQAVHSGDAQPFDFTDDESDVPVADVATPYWERPVGPTWWCHLDAADPFVTAWFGSSRWLHPAISIALQEESRLISDRMKHLLYEVPVRVAGGLLFELLGQSAGDPFAEEDDIPVVLRAWQAQNFLVTALHVKGSASNINVLGILEVQELLAAGGAKNPCSIHEVVAHLASRLARWDDRLFRKHIFGAADEVELMFMNRRSHEDLHLFTIILNQEIRRLSTQVIRVKWSLHAREEIVFELLKQLRGNAARALLEGVMKSTRQMIGEQEAVRGRLFIIQDVTQSTVRAWLQDRSLTVTHNLGIFGGCGLVLSIITGLFGINVDGIPGSSGTPYAFLLFTMILFVLGVVLIGIGLLYLGLKKPIIEENVALRKQELQELVRMFQHEAETHAQVRKTVPHKAQTAAVRPPNGANHRFIMSKLCSH

>GLYMA.03G159400

MALASSVVELQPSSVKKKTAVSRSWILLDHYGKGTVLDADKYAIMRLVQIHARDLRILDPLLSYPSTILGREKVIVLNLEHIKAIITADEVLLRDPMDDDVVPIVEELRRRLPQVSAAEQGQGKEEACAQDGEGGEENEFPFEIRALEALFEAICSFLDARTRELETSAYPALDELISKISSRNLDRVRKLKCAMTRLTIRVQKIRDELESLLDDDDDMADLYLSRKLDASSSPTSSSDAPYWLYGSPNTGSKRHKSSRVSGTTVQRENDVEELEMLLEAYFMQIDGTLNKLATLREYIDDTEDYINIQLDNHRNQLIQLELFISVGTVCMSLYSLVAAIFGMNIPYTWKAPGHEHVFKWVVIFGGMVCASLFLSIVSYARRKGLVGS

>GLYMA.04G005200

MGIAENSKGLVLAVASGVFIGASFVLKKKGLKQAATHGTRAGVGGYSYLLQPLWWAGMLTMLIGEVANFVAYIYAPALLVTPLGALSIIVSAVLAHFLLKEKLQKMGILGCVFCIVGSVLIVIHAPQEHALNSVQEIWDLATQPLFLVYVAAAVSVVLALILHFEPRYGQTNMLVYLGICSLIGSLLVMSTKAIGIAIKLTLEGTSQLTYPQTWFFLTVTVICIITQLNYLNKALDTFNTAIVSPVYYVMFTTLTIIASVIMFKDWSEQSAGSIASEICGFVIVLSGTILLHATREQEQSNKQGSLTWYIGEDLVKRIEDGHLNLLHGSDYVEK

>GLYMA.05G153000

MGASSDNVTGFVLAVCSSVFIGSSFIIKKMGLKKAGATGKRAGAGGHAYLYEPWWWFGMISMIVGEVANFAAYAFAPALLVTPLGALSIIFSAILAHFILKERLHIFGVLGCALCMVGSTTIVLHAPHERVIHSVKEVWQLATEPGFLIYMCIVVVVVCILIFYCAPRYGTTYLVIYVGICSLTGSITVMSVKAVSIAMKLTLEGNNQFIYFQTWFFTIIVIGCCLLQINYLNKALDTFNTAVVSPIYYVMFTSFTIFASIIMFKEWDTQDASQIATEVCGFITILSGTFLLHKTKDMGNRPIESPVFVSTPQNVSSHSGT

>GLYMA.05G168200

MGKGPFSFRRSASRRRPKKTAAPPPPPSPPQPPYAAGIATSPDDNNNRLIAAGAGSSALTKAKKKTGGARLWMRFDRSGRSELVELEKNAIIRHAAIPARDLRILGPVFSHSSNILAREKAMVVNLEFIKAIVTAEEVLLLDPLRQEVLPFVEQLRQQLPGKSQPKLLGGVEEQEGEMQVSNGRQWLPMPEAADGLQSELPFEFQVLEIALEAVCTYLDSNVADLERGAYPVLDELARNVSTKNLEHVRSLKSNLTRLLARVQKVRDEIEHLLDDNEDMAQLYLTRKWLQNQQFEEAHLGATTSNNFPNTSRSVRRLGSNRSESLVTCHYEDDNNVEDLEMLLDAYFMQLDGTRNKILSVREYIDDTEDYVNIQLDNHRNELIQLQLTLTIASFAIAIETMIAGAFGMNIPCNLYHIDGVFWPFVWITSAACVLLFLLILAYARWKKLLGS

>GLYMA.05G196600

MSSSNLTGFVLAVLSSAFIGSSFIIKKKGLQLASANGPRASVGGYGYLLQPLWWVGMITMIVGEIANFVAYIYAPAVLVTPLGALSIIVSAVLAHFLLKEKLQKMGMLGCLLCIVGSTVIVLHAPEEKSLSSVQEIWELAIQPAFLSYTASAIAVTLFLVLYCAPRHGQTNILVYTGICSIVGSLTVMSVKAVGIAIKLTLEGANQAFHFQAWVFAMVSVTCIIVQLNYLNMALDNFNTAVVSPIYYALFTSFTILASAIMFKDYSGQSISSIASELCGFITILSGTTILHSTREPDPPVVADLYTPLSPKVSWYIQGNSEPWKQEEDVSPLNLIAIIRQDHFK

>GLYMA.06G005000

MSMRGRFRGGNENDEKVVKCVESVRVIVMGIAENSKGLVLAVASGVFIGASFVLKKKGLKQAATHGTRAGVGGYSYLLQPLWWAGMLTMLIGEVANFVAYIYAPALLVTPLGALSIIVSAVLAHFLLKEKLQKMGILGCVFCIVGSVLIVIHAPQEHALNSVQEIWDLATQPLFLVYVAAAVSVVLALVLHFEPRYGQTNMLVYLGICSLIGSLLVMSTKAIGIAIKLTLEGTSQLTYPQTWFFLTVTVICIITQLNYLNKALDTFNTAIVSPVYYVMFTTLTIIASVIMFKDWSDQSAGSIASEICGFVIVLSGTILLHATREQEQSNKQGSLTWYIGEDLVKSIEDGHLNLLHGSDYVEK

>GLYMA.06G053100

MWESIVLTVVATAGNNIGKILQKKGTVILPPLSFKLKVIRAYALNKTWLIGFVMDIFGALLMLRALALAPVSVIQPVSGCGLAILSVFSHFYLKEVMNIVDWVGITLAGFGTIGVGAGGEEQEAAALSIFHIPWLAFVVFILFIMLNGWLRIFKRNRREQEMMEYDVVEEIIYGLESGILFGMASVISKMGFLFLEQGFPKLLVPICIIISVCSSGTGFYYQTRGLKHGRAIVVSTCAAVASILTGVLAGMLALGERLPSAPKARLLLLLGWLLIIVGVILLVGSTKLVRFFRFSSHRFKNYGPRRSGTSRVREPSPTAVIQAATLNHLLSSSSKEKA

>GLYMA.06G159100

MYSTNLIGFILAVVSSAFIGSSFIIKKKGLQRASLNGSRASGGGYGYLLQPLWWLGMVTMIVGEIANFVAYVYAPAVLVTPLGALSIIVSAVLAHFMLNEKLQKMGMLGCLLCIVGSTVIVLHAPQEKPLSSVEEIWQLALQPAFLLYTASTIAVAFFLILYCAPRFGQTNILVYIGICSIIGSLTVMSIKAIGIAIRLTIEGADQFVQFQTWIFTMVAISCIITQLNYLNMALDTFNTAVVSPIYYALFTSFTILASAIMFKDYSGQSISSIASELCGFITVLSGTTVLHSTREPDPPVNTDLYSPLSPKVSWYIQGNGEPWKQKEEDGPPFNLITVIRQDHFK

>GLYMA.06G208700

MAFLFYAALVITAIFILIFHFIPLYGQTHIMVYIGVCSLVGSITVMSVKALGIVIKLTLSGMNQLIYPQTWAFTLVVIVCVLTQMNYLNKALDTFNTAVVSPIYYVMFTTFTIVASVIMFKVSFVT

>GLYMA.08G126600

MGKTPFSFRRSASRRRPKKTAAPPPPPSPPQHPYAAGFAASPDDNNRLIAAAAGSSALTKAKKKTGNVRLWMRFDRSGRSELVELEKNAIVRHAAIPARDLRILGPVFSHSSNILAREKAMVVNLEFIKAIVTAEEVLLLDPLRQEVLPFVEQLRQQLPGKSQPKLLGGTEEQEGEMHVSNGRQWLPTPEAADGLQSELPFEFQVLEIALEAVCTYLDSNVADLERGAYPVLDELARNVSTKNLEHVRSLKSNLTRLLARVQKVRDEIEHLLDDNEDMAQLYLTRKWLQNQQFEEAHLGATTSNNFPNTSRSVRQLGSIRSESLVTSHYEDDNNVEDLEMLLDAYFMQLDGTRNKILSVREYIDDTEDYVNIQLDNHRNELIQLQLTLTIASFAIAIETLIAGAFGMNIPCNLYNIDGVFWPFVWTTSAACVLLFLLILAYARWKKLLGS

>GLYMA.09G019600

MSDTEGQSHSSGITKKLDSHKTYHGRDPNNGNDLWKDGLICAFEYIRGQNRSAKLSSSSSKITDGMHGQHSKMHHVPSDDKKKLSDPSSVNVSRESLFGGSDDDKESQTPKAGQSKKYEGGHWVPIGWARISELVQAVQVDAEWSSHQFEFEYSKDDFTVADLAAPYWEHPTGPIWWCHASAGHPTWLSNAQWLHPAVSLALRDESRLISERMKHLFYETSNVSHACIDLSALFFPTAFYVNFKLFRKSIFGAADEIELKFMNRRNHEDLNLFILILNLEIRKLSTQVIRVKWSLHARNEIVFELLQHLKGNGARNLLEGIKKSTREMIEEQEADRSLRVTHNLAVFGGVGVVLTIITGLFGINVDGIPGAEHTPYAFGVFTAILVFLGVVLIAVGMVYLGLKNPVAEGQVEVRKLELQELVKMFQHEAETHAQVRKNISPKNLPPTAGDGFRSDADYLVIQ

>GLYMA.10G180200

MARDGSVVPADPQAMAVVKKKTQSSRSWILFDATGQGTLLDMDKYAIMHRVQIHARDLRILDPLLSYPSTILGREKAIVLNLEHIKAIITAEEVLLRDPTDENVIPVVAELQRRLPRLGAGLKQEGDGKEYLGGQNDAEAAEEDESPFEFRALEVALEAICSFLAARTSELEMAAYPALDELTSKISSRNLDRVRKLKSAMTRLTARVQKVRDELEQLLDDDDDMADLYLSRKAGSASPVSGSGAANWFAASPTIGSKISRASLATVRLEENDVEELEMLLEAYFSEIDHTLNKLTTLREYIDDTEDYINIQLDNHRNQLIQLELFLSSGTVCLSFYSLVAAIFGMNIPYTWNDNHGYMFKWVVIVSGVFSAVMFLIITAYARKKGLIGS

>GLYMA.11G105300

MVSVVLPLIIHFEPHYGQTNMLVYLGICSLVGSLTVVSIKAIGIAIKLTLDGISQIVYPQTWFFLTVAIICVITQLNYLNRALDTFNATIVSPVYYVMFTTLTIIATAIMIGPGQDISSIASEICGFITVLTGTIILHMTREQEESNMQKTFTWFIGEDLMKDVENEHLILIHDSDYLER

>GLYMA.11G255400

MGKTHDNVVGLILAISSTVFIGSSFIIKKMGLKKAADHGNRAATGGHSYLYEPWWWAGMISMIAGEIANFAAYAFAPAILVTPLGALSIIFSSVLAHFILKEKLHIFGVLGCALCVVGSTSIVLHAPKEKDIHSVKEVWELATGPGFIVYICAIVILVCVLHFRFVRSHGQTHMMVYLGICSPTGSITVMGVKAVGIALKLTFEGTNQFVYFETWIFTVVVIGCCLLQINYLNKALDAFSTAVVSPVYYVMFTSFTIVASIITFKEWAKQDSTQIATELCGFVTILSGTFLLHRTKDMGNKPSDASVHSSPEDNNSNTKTPLSNQI

>GLYMA.12G030100

MRGERKKVKNENIRVACEGMSHRPTPVQQFSVTKFDLSFFLSLAGMTETGVSDNFKGLILAMGSSAFIGSSFILKKKGLKRAAARGTRAGVGGYTYLLEPLWWAGMVTMIIGEIANFVAYIYAPAVLVTPLGALSIIVSAVLSHFLLKERLPKMGVLGCVSCIVGSIVIVIHAPQEQTPSSVQEIWDLATQPVSVVLALIVHFEPRYGQTNMLVYLGICSLVGSLTVVSIKAIGIAIKLTLDGISQIAYPQTWFFLTVATICVITQLNYLNRALDTFNATIVSPVYYVMFTTLTIIASAIMFKDWSGQDVSSIASEICGFITVLTGTIILHMTREQEESNMQKTSTWFIGEDLMKGVENEHLIRIHDSDYLER

>GLYMA.12G168000

MATSSSSSSTSSWREGMSSDNIKGLCLALSSSFFIGASFIVKKKGLKKAGASGIRAGSGGYSYLYEPLWWVGMITMIVGEIANFAAYAFAPAILVTPLGALSIIISAALAHIILRERLHIFGILGCVLCVVGSTTIVLHAPQEREIESVSEVWDLAMEPAFLFYAALVITATFILIFHFIPLYGQTHIMVYIGVCSLVGSLTVMSVKALGIVIKLTLSGMNQLIYPQTWAFTLVVIVCVLTQMNYLNKALDTFNTAVVSPIYYVMFTTFTIVASVIMFKDWDRQSPTQVITEICGFVTILSGTFLLHKTKDMADGLQPSLSVRLPKHSEEDGFDGGEGIPLRRQEAMRSP

>GLYMA.13G368400

MRRKGVGTTGVKSWMVVSETGHARLEDVGKHSIMRRTGLPARDLRVLDPVLSYPSSILGRERAIVVNLEHVKAIITASEVLLINSSNPFFLSFLQDLHIRLSNLNPSSMSNDMDGGYEEKPLANDSRNGSPVRIPEDSDADFLVRADSLKSSAETGTGTGTGTPAPKPLPFEFKVLEACIESACRCLESETSTLEVEAYPALDELTSQLSTLNLERVRQIKSRLVALSGRVQKVADELEHLLDDDNDMAEMYLTDKLNARLCDQTSLKEGYNSEFEDNDQSDESNSEKYDRFLCPKLDVEELEMLLEAYFEQTNGILQRLTSLSEYVDDTEDYINIMLDDKQNELLQAAIIFDTINMILNAGIVVVGLFGMNIQIDLFNGQPRQFWATTGGTFGGCLLLFLVCLWWGKKRYFLSH

>GLYMA.14G033700

MGLSKENLKGLILALVSSGFIGASFIIKKQGLRRAAAVYGVRAGVGGYYYLLEPLWWVGMITMIAGEVANFVAYAFAPAVLVTPLGALSIIVSAVLADIILKEKLHNLGILGCIMCIAGSIIIFIHAPKEQPITSVLEIWNMATQPAFLAYVGSVIVLVFILVFHFAPRCGHTNVLVFTGICSLMGSLSVMSVKALGTSLKLTFEGKNQLIYPETWFFMLVVAICVIMQMNYLNKALDTFNTAIVSPIYYVMFTTLTILASVIMFKDWDGQSGGTIVSEICGFIVVLSGTIMLHATKDFERSSSFRGSAPSSPTLSARLFTGNGDSLLKQDEENGSPESNMCSRRQELY

>GLYMA.14G097400

MWESVVLTVAATAGNNIGKILQKKGTIILPPLSFKLKISLCWQVIRSYALNKTWVVGFLMDILGALLMLRALSLAPVSVIQPVSGCGLAILSIFSHFYLKEVMNAVDWVGITLAGFGTIGVGAGGEEQEVVALSIFHIPGLAFVVFILFILLSGWLRICKCQRREQEMVEYDVVEEVIYGLESGILFGMSSVISKMGFLFLEQGFPKLLVPMCIMISVCCSGTGFYYQTRGLKHGRAIVVSTCAAVASILTGVLAGMLALGERLPSEPKARLALLLGWLLIIVGVILLVGSTRLVRFLSCSSQRKRSNVDKNFDLRRATSSRVRETSPSAVIQAATLNHLLSSSSKEKA

>GLYMA.15G125900

MSDTEGKSHSSGITRKLDSHKTYHGRDPNHGNNLWKDGLICAFEYIKGQNRSVKSSSSSKITDRLHVNGQHSKMHVPSDDKKKLSDPSSVNVSRDSLFGGSDDDKEGQAHKAGQSKKYEGGHWVPIGWARISELVQAVQVDADWSSHQLEFEDSEDDFTVADLAAPYWEHPAGPIWWCHVFAGHPTVEAWLSNAQWLHPAVSLALRDESRLISERMKHLLYEVPVRVAGGLLFELLGQSAGDPLVEEDDIPIVLRSWQSQNFLVTVMHIKGSVSRINVLGITEVQELLSAGGYNMPRTVHEVIALLACRLSRWDDRLFRKSIFGAADEIELKFMNRRNHEDLNLFILILNQEIRKLSTQVIRVKWSLHARDEIVFELLQHLKGNGARTLLEGIKKSTREMIEEQEAVRGRLFTIQDVMQSTVRAWLQDRSLRVTHNLAVFGGVGVVLTIITGLFGINVDGIPGAEQTPYAFGVFTAILVVLGVVLIAVGMVYLGLKNPVVEEQVEVRKLELQELVKMFQHEAETHAQMRKNISPKNLPPTAGDAFRSDADYLVIQ

>GLYMA.16G003900

MATSSSSSSWREGMSSDNIKGLCLALSSSFFIGASFIVKKKGLKKAGASGIRAGSGGYSYLYEPLWWVGMITMIVGEIANFAAYAFAPAILVTPLGALSIIISAALAHIILRERLHIFGILGCVLCVVGSTTIVLHAPQEREIESVSEVWDLAMEPAFLFYAAMVITATFILIFHFIPLYGQTHIMVYIGVCSLVGSLTVMSVKALGIVIKLTLSGMNQLIYPQTWAFTLVVLVCVLTQMNYLNKALDTFNTAVVSPIYYVMFTTFTIVASVIMFKDWDRQSPTQVITEICGFVTILSGTFLLHKTKDMADGLQTSLSIRLPKHSEEDGFDGGEGIPLRRQESMRLP

>GLYMA.16G149500

MGEWIVGAFINLFGSIAINFGTNLLKLGHNERERHLLGSDGVNGKMNLKPIIYFQSWRIGIVFFFLGNCLNFISFGYAAQSLLAALGSVQFVSNIAFAYFVLNKMVTVKVLVATAFIVLGNVFLVAFGNHQSPVYTPEQLTEKYTNIAFLLYLLALISIVALHHSIYKRGELLFAVSGHDLRPYWSMLLPFSYAVVSGAVGSCSVLFAKSLSNLLRLAMSNGYQLHSWFTYSMLLLFLSTAGFWMTRLNEGLSLFDAILIVPMFQITWTFFSICTGFIYFQEYQVFDALRTTMFILGMMCVFIGISLLAPDESKVSGPETKDSSLDSMVSSAMSTETSRLVVSPEEAQNKDSRSFVKAILIKVTDLLVKAKTSCALSLGFGEDTINTSSVLVMPMMSSRMTGFRGNGLERARILSMRNGWSKIPMDEDAGKLLETSSVVPPSP

>GLYMA.17G227100

MWESILLTVAATAGNNIGKILQKKGTIILPPLSFKLKVIRSYALNKTWVVGFLIDIFGALLMLRALSLAPVSVIQPVSGCGLAILSIFSHFYLKEVMNAVDWVGITLAGFGTIGVGAGGEEQEVVALSIFHIPGLAFIVFILFILLSGWLRICKRQRREQEMMEYDVVEEVIYGFESGILFGMSSVISKMGFLFLEQGFPKLLVPMCIMISVCCSGTGIYYQTRGLKHGRAIVVSTCAAVASILTGVLAGMLALGERLPSEPKARLALLLGWLLIIVGVILLVGSTRLVRFLSCSSRQKRSNVEKNFGLRGATSSRVREPSPSAVIQAATLNHLLSSSSKEKA

>GLYMA.18G091200

MIMLLSIFATTQEHNLTTNQEHNFSTAAFLFYAALVITVTFILIFHFIPLYGQTHIMVYIGVYSLIGSITVMSVKALGIVIKLTMSGMNQLIYPQTWAFSLVVIVCVLTQMNYLNKAVDTFNAAVVSPIYYVMFTAFTIVASVIMFKGFIAYALNYGLITW

>GLYMA.20G210300

MARGDGSVVPTDPQTMAVVKKKTQSSRSWILFDATGQGSLLDVDKYAIMHRVHIHARDLRILDPLLSYPSTILGREKAIVLNLEHIKAIITAEEVLLRDPTDENVIPVVEELQRRLPQLSATGLQQQGDGKEYLGGQNDAEAAEEDESPFEFRALEVALEAICSFLAARTTELEMAAYPALDELTSKISSRNLDRVRKLKSAMTRLTARVQKVRDELEQLLDDDDDMADLYLSRKAGSASPVSGSGAANWFAASPTIGSKISRASRASLATVRLDENDVEELEMLLEAYFSEIDHTLNKLTTLREYIDDTEDYINIQLDNHRNQLIQLELFLSSGTVCLSFYSLVAAIFGMNIPYTWNENHGYMFKWVVIVSGVFSAVMFLMITAYARKKGLVGS
