# Supplementary material for: Genome-wide identification and functional characterization of magnesium transporter (MGT) gene family in soybean (Glycine max L.) and their expression profiles in response to aphid infestation, dehydration, and salt stresses
Source: PLoS One. 2025 Aug 29;20(8):e0330440. doi: 10.1371/journal.pone.0330440 (PMC12396710; doi:10.1371/journal.pone.0330440)
Supplement: S5 Data — (S5 Data.DOCX) [file pone.0330440.s005.docx]

| Group | Number of PP2C genes | Gene ID |
| --- | --- | --- |
| A1 | 07 | *GLYMA.02G117100, GLYMA.03G159400, GLYMA.05G168200, GLYMA.08G126600, GLYMA.10G180200, GLYMA.13G368400, GLYMA.20G210300* |
| B1 | 03 | *GLYMA.02G285600, GLYMA.09G019600, GLYMA.15G125900* |
| C1 | 11 | *GLYMA.02G280800, GLYMA.04G005200, GLYMA.05G196600, GLYMA.06G005000, GLYMA.06G159100, GLYMA.06G053100, GLYMA.11G105300, GLYMA.12G030100, GLYMA.14G033700, GLYMA.14G097400, GLYMA.17G227100* |
| C2 | 08 | *GLYMA.02G068000, GLYMA.05G153000, GLYMA.06G208700, GLYMA.11G255400, GLYMA.12G168000, GLYMA.16G003900, GLYMA.16G149500, GLYMA.18G091200* |

**S5 Data.** Distribution of *GmMGT* genes among groups based on phylogenetic analysis with Arabidopsis, rice, and chickpea *MGT* genes.
