# Supplementary material for: Genome-wide identification and functional characterization of magnesium transporter (MGT) gene family in soybean (Glycine max L.) and their expression profiles in response to aphid infestation, dehydration, and salt stresses
Source: PLoS One. 2025 Aug 29;20(8):e0330440. doi: 10.1371/journal.pone.0330440 (PMC12396710; doi:10.1371/journal.pone.0330440)
Supplement: S6 Data — (S6 Data.DOCX) [file pone.0330440.s006.docx]

**S6 Data.** *In silico* predicted the number of introns and exons in *GmMGT* genes.

| **Group** | **Source accession** | **Intron** | **Exon** |
| --- | --- | --- | --- |
| A1 | *GLYMA.02G117100* | 5 | 6 |
|  | *GLYMA.03G159400* | 9 | 10 |
|  | *GLYMA.05G168200* | 3 | 4 |
|  | *GLYMA.08G126600* | 3 | 4 |
|  | *GLYMA.10G180200* | 10 | 11 |
|  | *GLYMA.13G368400* | 5 | 6 |
|  | *GLYMA.20G210300* | 10 | 11 |
| B1 | *GLYMA.02G285600* | 6 | 7 |
|  | *GLYMA.09G019600* | 5 | 6 |
|  | *GLYMA.15G125900* | 6 | 7 |
| C1 | *GLYMA.02G280800* | 8 | 9 |
|  | *GLYMA.04G005200* | 7 | 8 |
|  | *GLYMA.05G196600* | 8 | 9 |
|  | *GLYMA.06G005000* | 7 | 8 |
|  | *GLYMA.06G159100* | 8 | 9 |
|  | *GLYMA.06G053100* | 8 | 9 |
|  | *GLYMA.11G105300* | 4 | 5 |
|  | *GLYMA.12G030100* | 9 | 10 |
|  | *GLYMA.14G033700* | 8 | 9 |
|  | *GLYMA.14G097400* | 8 | 9 |
|  | *GLYMA.17G227100* | 8 | 9 |
| C2 | *GLYMA.02G068000* | 12 | 13 |
|  | *GLYMA.05G153000* | 7 | 8 |
|  | *GLYMA.06G208700* | 2 | 3 |
|  | *GLYMA.11G255400* | 7 | 8 |
|  | *GLYMA.12G168000* | 8 | 9 |
|  | *GLYMA.16G003900* | 8 | 9 |
|  | *GLYMA.16G149500* | 12 | 13 |
|  | *GLYMA.18G091200* | 3 | 4 |
