# Supplementary material for: Genome-wide identification and functional characterization of magnesium transporter (MGT) gene family in soybean (Glycine max L.) and their expression profiles in response to aphid infestation, dehydration, and salt stresses
Source: PLoS One. 2025 Aug 29;20(8):e0330440. doi: 10.1371/journal.pone.0330440 (PMC12396710; doi:10.1371/journal.pone.0330440)
Supplement: S11 Data — (S11 Data.DOCX) [file pone.0330440.s011.docx]

>GLYMA.02G068000

ATGTATCAATAATTAAATAATTAAAAAAAATCATATTAATGATAAAAATAAGATAAATTTATTATTATCAACTAAATTAATTATTTTTCACAAATGAAAAATAATATGTTAACACTTACAAGTTAATTTCAACGCCAAGTGTATAGTGAAATCTTTTAATCCTCTCATCTAAACTAAAAATAGAAATTAGCTGTAATAGTTGCTACTCAAGTTTCAAGTCTCAAGTTTCAAATTGTTTATCTTGCCTTTGAATGAAGGATTTTAAATAAGGTAATTCATTTTTTAAGTGAATTTGAAATACTTTTATCATAATTTAGTTGTTTGGATACAAAATTTTAAAGAATTTACATTTACATCAATTTTTAAAAGAATTTTAATAAACTAAAACAGTAGGAATTAAAATTTCACTTAATTGGTAAAAATTTCAAATTGTCTCCTCTCATGCACATACCTTCGTTCTCTCTCTCTTCCACACGAAAATCATGGGCAATGTCATCAGATACACTTTCGCACCGGAGGTAATTGCATGAACGAGGATCCAAATCCAAAGCGGTGAAGTGAATGTTGCGGCGGCGAGAACTCGTGACACAGGTGTGAAGACCGTGAGGATGACGTAGATGAAGATAGCAGTGACGTTGGGGCGCTTCTAGTTGTTGTCGCAACAGAGGGCTACCAGAAATGTTGGCAGTGCAGTAGACAAGGACAAAGATCATGCCATCGAAGGCCTGTGCAATGCCGAGAAGGCCAATGCCGTCACATGGTCCGGTTTTTTTTTTGTGACCAATGACGATGGCGATGGTGCCGTAGAGGAAGAGAAGTGTGACGACAAACTCGACAATGAGGGCTTTGTAGAAGGACTAGAGCTTAATCTTAGCCAAGTCAATGAGGGGCGCTAATGGAGGGTCTACATAATCCTTCCTCTACTGGCTCATTTCCTTCGACATTCTCTCTCTCTCTCTCTCTCTCTCTCTCGTAGGAACAACAGATGGAATGAGAATGGATGAGGTAGGAGAGAAAACCATAGAAATATCTCCCCATAAACAACACCTCTAAATATAAGTCAAATCAAATCAAATTTCAAAGTTTTCGTTGAGTAAAAGGATGATCCGGTCCTTTTCTGGCCAGTGGTCTCTCATCTCAACCTAAATGTTTCCACTGTCGTCTTTATCCGGCCAAACACATACATGTCATAACTCACAACGTACAACTATTGCTTTGAAACAAAAAGAAAAAGCAATGCTATAATTCATACCCCAATGCAAAGTCATCCCTTTTCAATAAAATTATTTTAATTATTTAAATATTTTAAAAGACTATTATTTATTTTTATTTAATATTGAAATTTTAATTTTTAAATAAATATTTTAAAATGAAATTTTGAATTTCATAAAATTGAATTACTTTATCCAAACTAAAAAATTAAAATACAAAAGATTGAATTATTTTATCCAAACAAAATATTTATAAAATAAAAATAATTTAAATCAAGACAATTAAAATATTATATATTTAAATTTTTTAAATATTTTCAAATCTTTCATCCAAACACAAGACTAATCTTTTTTTTTTTCTCCTCTCTCTCCTCTTTTCTTTGCTTCTCGAATCTATGACAAATCCTCCGTTCATTTTGGACCAAGAAAAGAAAAGTCCCACACTCCTTCTTATTTTTTTTTAATAATAATAAAAATATTCAAATCTCTGATTTAATGACCCACGTCGATTAGCCATTATTTATAAAAGTTACCTTTTTTTAGATTAGATAAAAGTTACCTAAATTATTATTTATAAGATAACTCCCCATGATTTAAATCGGAGATATTCCAGTAAAAAAAATCAGATATATAAGTTTAATTGTTTTTGTTTAAAATTATCATATTATCCTTGTTTTTTAGGGGGGAATTTTTGGTTTTCTTATTTATTTATTTATTTATGTATACTACTTTGTTATACCGTTTTTGGTTTTTATCCACAATTTCTGTTTTTATAGTTTTTTTTTTCTT

>GLYMA.02G117100

CAAAAAATATATAAAAATTAGCATTGAAAATTAGTTTGCATGAGCATGATTGTAGGGTAAATAAAGAGCAAAAAAGAAGTTGAGACCTCTTCATCACACACCATGTATGTGCTAATTAATTCTTTATTCTTAATAATATTAAGAGTGTCCCACAAGCGCAAAACTCAGACCTTTATGATGGTATCCTTTGACGTAGAAGAAACTTAATTCAACAAAATATATCTATCAAAAGATATAAAAAAAATGTTTAATCAAATTTGTTAATTTTTAAAAGTAGAAAATTTCTGTACTTACTTCTTCATAGTTTTGTTGCAATTGGCATAATTTCTGGTGTGTGGACTAATGATAATAACCTGCAGCTTGAGGCGACTATGCAATTCAGTTTCGAAAGTTACTTTCAATTGGTTTGTGATTGGTGTCTTAGTGTTTAGGGCCAAGCTACATTGGACCACCTTAATAATGGTCCATTGTGGCCATAATAGTGGCCTTTTGATTGTGTACATTGGAACACTAGCACAAATGTGTTCCAAACCCCACACATAAAGGTCACAAACAAGATCCAAAAATCCGTGCATTTAAGCACTTCCATTATTGCATGCTCCTCACCCACTATCTCAGATTCTCAGCACTTTCATTTTCCTTCTCCGGACTCCGAAGCATCAATTCTTCATCCTCTGCCTCTCCATGTCATTCCCCTGTCCGAACCAGCCCTTCAAGTACGAGTACGGGATGAGGAAAAACCCATACACCAACAGATAAAGACTTTGAAGTACTTTACCTCTTTGTTGTCGCTGGCGGCAACAAGAGGAATCTTGTGAAGAAAAAAACAATCGAGTGAGGGAAACCACAAAGGAAACAAAGGGTAGTGAATATCACCGTGCTCAAGCCTACAAAGCCTTTCAAAATCTTCCTTCTGCTTGGAACCCTAGCTCTGATTACCTACCCATTTTAATAATTGGGGTTGGAGGAATAAAGGGTGTGGAAGGTTTAGTTTTTCTATTTTTAATTAGTGTTTCATTTGAGTACACCGAAAATCGTGGCGACATACAATGTCACAGGGAATAGTTGTTAGGAAGAAGGAAAAAGACAATATTATTGTTCAGATGGTACACTATAACACTGTAAGCATCATTAATAGTCCACAATTGATCATTTATTAAGGTGGTCCACTTTAATTTTGGCCTAATTTTCCAGGATTTCTTATGATCAAATATTTTTTTTCCAATAAAGTTTTAACTTAATGTGTTACAAAATAAATGAGGAGCTTATAAACTACTAAATATATACTTATAAAAAAATATAAAATATATTTTTTATCCTTTGAAATTTTTAAAGTTCGATTTTTTTAATTTTTCTAAAAAAAAGTTTTAAATTTTTTTGTCCATTTTTCACCTCTCTAATTTTGAAAATTTTGTTTCATTTTTAAGAGTTATCTAAACTTTTTTTCATTTTTCTATAAAACTCTATCTAAAATATTTTAAAGGATTAAAGGTATTTTAAATTTAAAAGGATGAAAAATAGATTTCAAAATTAAAAGGACAAAAAAGTTTAGAGAGACTAAAGTCGATCTTTAAAATTTTTAAAAAACCAAAAACATATACTTGTAAAAAATATGCTATCAAAGGATTTAAGTTAGACAACCTTTTCTACAGAAAAACTACTAGTTTAAGGCTCTCATTGGATTGGTCAATAGAAATAGCATATGATTCATGAGTCTGTATGATATTTGTTTTCCCAAATTTCTTGTCCTTGTCAATTTTTAAAAAATTACATTTTTTTACTATCATCTAATTAATACCTTTATTTTTAACATATATATGTATATTTAATGCCACAAAAATATGTATATTTAATTATTTCACTTTATCATTTTTTTACAAAATATACTTTATCTGATAATTATATGAAGAAGCTAGTTAATCAAATAATTATACTAAATACTTCTAATTTAATTAACTAATTTATTGACTTCAGTTATGAACCCCTTCAGGAATAGGACC

>GLYMA.02G280800

CATGAACTTAATATGGGTAATAAGTTATAGCAAGTAAGGTTAAAAATCCCATATTCAATTTGAAATTTTAAAATGAGGCCCAAACTCACTAAAAGTTTGGTTTGTAACGTTTTTTCCACTTTTGAATCAAAACACAATCTGATCAAGCTAATGCTTAAAATTTGAAGTCCAAATCTTGCATTTCTTTAGCAAAATCCATGTGAAGTTAATTGACTACCACCTTTAGTGAAGATTAAATGAGAAGGGAATAAGATTGAATGGGAAGTGAAGAGTGAAAAATATATATTAAAATAGACAACACTTTTAAAAACTTAAAAATAAAAAACGTATTTTCAAATGAGTTAAAAAGGGTTTTGAAATTCATTTAAAAACTCCTTCAACCCTGTTTTATCCAAACAAACTTTATTTTCAAATTTTTGAAAGCAACAAACTAAAAACTCACAGCCACTTTAAATGCAACCTTAATTTTGTCTGTGTATTAATATTTGTTGTCTATCAAATGTTAATAGTATATTTTTTATTTTTTTAAAATTCTTAAAATTCATTTCCCCTTAATTTAAACAATTTATGTTTTCATTTAAATTAAAAAGAAAAACTTAAATAATGCACCTAATTCTTGGTTTATGGAGCATTTTGAAATATTTTCTTAATTGGTTGCCGACATTGGGCTACAATGGGTCATGTTAGATGCCCACATGTGATGTGAGTACTTACTGAATACTGAGTAAGACCAACACAAAGCTATTAGCAATGTCATATGTGATAGCCTCACATCAAGAAATCCTCACCAACCACCTAATACGAAACACGTGTCCACGTTACATTTGGCTACCATGGAACGATCCCCATAAGATGCTTCTTCAACACCACACCACACGGGAGCTTCAAGTGCACATGCACTAAGTCAACACGCATACTCACCTTAATTTTCCCGACACCATCACAAAAAAATTCAACACGCGGTACTATTTGTATTATTTTTTAACTTTGTTTTTTTTTTTTTATAAATAAAATGCCACAAGTTGTAAATTTTTATAAGATAAAGTTAGTGGATTATTTTAGGCAGTTTTATTTTGATTATGTGTGTAGGACGACAACCTATTAGACCAGGTTGTAACGTTCCCACTTGTTATTTTAAACCAATTTTGAATAGTATTAATTTTTTAAGTAAAACTTTATTTTTAATTGAAAAATATTATATCTAAAATATACTTAGGTTTTATCCTTTTCTCTTTCAAATTAGCCTCATCGAATTTTAATTTTAATTAGAACAACTTTTATTCTTTATGAAATGTTAAAATTTAAGCATATATAAAAAAATATTATTAAGAAATTTTTATTCATTACTTTGAGTAGCTAACTAGTTTCTCATGTTTTTTTTTTACAGTAACTAATTTCTTATGTTAGTAATGAAACATCCCGTGCAAAACCAAAATATAAATGTTAATTAATGCTTAATGAAGATAAAAAATATTAAAGTATATATTTCAAATTAAAGATACAAAAAATATTTAAAAAATAAATGTTTTTTACTAAATTAATTAACTTTTTCATTAAATAGGAAATATTATAATTATAAGCATTAAACAATGCCACATATTTTTATTGGTGTATTGATGAAATAAAAAAAAATTACAAGACTGAAAAGTCAGCATTAAACAATGCCATATACGTTATCACAAATCAAGGATTCTACTGTATTATAATACTCCTCAATTAGGTAACATTGTAAAAAGACTTCAATTGACCATATCAGGGCAAAGAGAATTCAATTAAAGGTTGCGTTAAAACTTTAAAAACTTGAATGCTTCCCTCGGAGAAAAAAAACGATTGGATTAGAACTTGCATGTTTCCTTTAAAGTCAGACCTTATATTCATTAATATGTCCGCAATAAATGCGAAAATTCTGAGCATACTACTATTAAAAAATGGTTAAAAATAACAAGTGGGGCCCTCACAGGTGGTGTAGAATAATAAGGTTGTGGTGGTACACACTCTAT

>GLYMA.02G285600

AGGTGATAATAATCTTAGAAAAAAAAAATAAATCTAATCAATTCGATATATCTAGATTGTAGTTTTAAAATGCAAGTCAAATTTTTAATTAAAATTAAGTAAAAGTATTTCAAAGAATCCACGTTGGCTGATTTCCATTTGGAAGGTCTCCCTTAGAGATGCTAGAAAGGAATCCACGTTGAATGCTAGGTCACACGCTTTTTACTGTGTATTCAACATTTGAGATGCTATACTAGCATCTAATTTTGAGGTCTTCCCATATACTTTTAGTCTACTGTCTAAGACACCTGATTAGTGTTGGGTCCTAACATTTACATCAAGATTGCTTTTTCTAGTGTCAACAAAATTGGACTTTTTATGGTTGCAACCTGAAAAAAAAAAATTGAGAATTAAAACTAATGAAAATTGCATAAAAAATAATTTTTATGAACAATACAAAAAGAATATAATTATTAGATTTTAATTTTCTTATGACTAATAGCGAGACTTAATTTTCATGTAATTTTTATATTTTTTTATTCAAACTTAGTCGTTTCTAATAATTTTGTACATGAATCTAATAAGACATATTTTATGTATTGAATCAATAAAAAAAGATTCAAAATCATATAAGAAGATTCAAAATCGTATAAGAAAATAGTTTAAACTAATAAATATTAATTACTATTTATTAATTTTTATTAGTGAAAATAATCAAAATTGTTGAAACTATTTTAAGGGTTTACTTGAGAATAAATTAAATTGTTGAAGAAGGATCACATGTATATGACATGGCATGATCAAAATTAAATAAAAAACATGATTCTTTTGCCAAAACATTCTTAAATTTTATATAAAGCAAACAACAAACAAACTATATAGAATAAACTGGTATCACGTGACACCAGATTGCTTGAAAAACTCACCTATTTGTATGTTCCCCCATAACAGTGGCTTCTCCAGGTTCTGACATAAACTTTAAGTATTTGACAATATTGAAAGATAAAGCTACAGTGAAACCTACGTCAATATACATGTAACATTAAATAGAAAGGGGTGATATTAACTATTAAGGAATAAAATAAGATTACCATTAGAAGTTGCACGTAATGTGATCCTTTCTGCACAACACCAGCTTGTTCACTCTCAGTCTTGCCTTCTCCACTTGACCAAAGATCGTATTCCTTACCACTTGAAGTTAAACTTAAAACATTATTCTCATCGAATCATAAAAAGGCTTTTTCCATCAAATTGTTGTACGTAGTTTGCTTTTAGAACCACTATTTTAAATTAATTATGCGAGTTATTATTTGAATTATTTAAGTTATTTAATTATTTGAGTTACTATATTTACATTATTTCAGTTTCCAACCTAACTCACCATAGCTAACAAAAAATAGTAGAACAAGTACGAAAGAAAACATCAAATTAAAGATAAAAGAATTCTTATCTCTATCCACCGTAATCATTTTTAGCAAAATAAAATCAATCACAATCACTATTGTAAATCTTGACAATCTCTACTAATTAATTACTAGAATTTATTTTCAGCTTATTAACTAAATAGTTCGTTTGATTGTCAGTATAATAAGATAAAATATAATATGAGAGTAGAATAAGAATAAAATATGATAACGTAAAAAAGAAATATATGATAAGAGTACAATAAATTTTAATCTAATCAAATATTTGGTTTGCGACAGGCAACAAATCAATAATAAAGTAATATAGAAAAGATAAAATTATTGTTTCATTAAAATTACATATATTATGGAATATGAAATTATTAAAAAAATTATAACTAAATTATAGAAAATATATTTTGTTGGAGTAATATTCTTCAAAATATAAACAAATTATATAAGTTTTTAAAATTATAATTATTATTCAAATAATTTTCTTTAAAAATATTTGAATAAGTAATGATTTAATTTTTAATATTTTAATTAAGAATATAATTTAATTATTTTTCAAAATTATTCTCTCCCTATATATAAATTATTTTTCGTATATCAATGGCA

>GLYMA.03G159400

AAAGATAGACCCAACATTTGAGACATAAGGTTCTACTTTTCAAATCTTAAATGTACGTACTCATTTACTTTCAATGCATTTTTCTTTACATTTCTTAGTTCAATTCTTATTTTCAGTCTCTTTTTATTTTATTTTTTGACTGGAAGTCTCTTTTTTATTTTCAATTTGCACTTATAATGTATATTTAAACACAAACTTAGAGTTAAAGAGAAGTTAATTGCCCTTCCCAAAATGTTTTTTTTATGAAAAATATTATAGACATTTTTTTCCACGTAAATACATACTCATGCACGTGACAACAACAAACTTCTCCATATTTGTATGAAATTGTTATATTTTAATTCAAGAAATACTACCAACATACTCTTGAATAATTTTTTTTTATTAATACTCTTATTTTATTAAATTTACATGGATCGTCTCATTAAATATGGGAATTTTATTCTCTATTTAGTAGATCTCATTTTAAAACTTTTTTGGGGGATATATATCTCATTTTCAAACTAATTGAATCCATATACTTTTAAGCAATAAAAAATGGGTTAAAAAGAGAATGTTAAAAAGTGACGAGCTCTTTGTTGTTCAAAAGTCACGGAAATTTGGAAAAGAAGACAAGAAGAATTCCTGCCTAATGGCTATGAAGTTAAACTCGAGCAAGGCTTATGACAAATTGAAACGAGTGTTTCAGATGAGTATATTGATGAGATATTTGAGCCTATATAAGGGGTCTCAGTGCAGGTAGTGTTGACTCGTATGTCCATGGCATTGTCAATTTCTATTTGGGCCATTGGCAATTTTCTGGTTTTAATAACTGTGACTTTTCTCCTGTCTATTGCAATATGGTTGCGGATATTCTTGTATAATTTGCCATGAGTCTTGATTTGGAAATTTGGTTAGAAACTCTGATAGCTAGTTTGATTGAGAAGATGAAAATAAAGAAAACGAAAATAGAGAAGAAATATTTGACCTAAAATAGAGTGTAAAAAATGTAAAAATCATATGAAACTCATCATATTTCTTCTCTATTTTTTTCATCTATTTCTCTATCAAATACACTCTAAGTTTCATTATTTTTATTTGGATTTCTGAGTTAAGTGATCTTTCAGTTTCTCATTTTCTTCTTAAAAAGTTGTATTTTATATATTTGTCGCATTTAACATGTTAAATAATGATATTTATTTTCATGAGTTATATAAAAATACAATTCTACCCATAAATATAATTATAAAGTACAATTCACTATTTTTTAAAAATAAAACCATCGAGCATGTGCACTAATCAATACAATTTTGTTTTAATAAAAAATTTCAATTACTTTGAATATACTATTATTGCAAAGAAAAGTTTTGTTGGCTATACTAATCACACTTGAAAAATATTCATAGCTATAATAAAAAAAGTAATAATCATGCGTTCGTTAACAGGTGGAATACTAGAAGTAGGTAGACCATTTTTTAAAGAAAAGAACTAGAAGTAGCCAACTTTACTGTGTGTAATTTAACGCACTCAGAATTTTAAGAAAGTACAGATATTTTTACAGGCGATTCCATAATCTTTTCTTTGGTTACATCTATCAATTACAATCTACCACAGTCAAAGAAATTTTAACATATTATCTTTCATTATGTTTGGGCACCTTTTTACAACTTAAAAACAAAAGGCAATATACAAAAAAAATGAAAAGAAAAGGCCGAAACCAACCAATAATCGGACCACCCTCGTGCAAATAACAAATTGACGGATGCAAACTCAAGAGAAAAAACGTGCGACCAAGAATTACCTCCAACGTTGTACGTTGTTCCTTCATTCCTCCTAAAGTTCAAACCTAAACGTGTGTTAAGCATAATTGTGTTCCTTATCAAGATATAATCTGAGTTTGAATGATTAAAGAAAAAAAAAATGTCAAAAATTCAATTAACAGTAACAATTAATATTTATGGATAAAAAATAAAAAACAAAAAATTGTGTCCATTTTATGTTTAACCTCCTCTCTACTACTG

>GLYMA.04G005200

CTTGTAATTAAATATATTCCTCATAAAACATCTATGTATCCATGGCATCCTTCAACTTTCTTACCAGGTGAACCTTTGCATATTTACTTGATCTGATTTGTTTTATTGAAGTAATTTACTTGATGTGATATTATAACAAGTGTGAGGAACACACTTTAATAAGCACACTCTTTTAAATATTTTTTTAAAATTATTAATTAAAATATATTAAGATTCATGAATTAATCCTATTCATAATTTTATAATTCCAAAAAAAAAGTAATAGTTATAGATTAATATTTTAAATCCATATGTTAGGGTTTTTTTTTTATAAATTAGTATTCGTTGTATAAAACTAAATAAACTATTATTTGTTATCTAAATTGTCAATAATACTAAAAGAGCATTAGCAACATACTCAAAAATACTCATTCTAACACATTTTTTTATCAATCAAAATTTCTTGACCTACTTTCTATAGCAAGTTAAATCTCTTTGCCTCACTTTATTAGGCTGGTTGATGGCAGGTTAAGATAGATTAACCCGCTTTTTATGGGTGTTTATGGGTTTGATTTACTTCATATTTGGGAAAAATAAAATTCAAATAAATTAATTTTAATTGTTTGGGTTTTAGATTTTTTTTTTTTTTTTCCTAACGTAAACCAAATTAACCCTATGTTGAATGGGTGGTTCGGTTCATGTTATTGAACCGGGTATCCAACAAAAAGAAAGAGAAAATCTGTTATATGATTTTGTCTCAAATTTAATAAATAATATTGTCATAAAAAATGTAAATAATATATTTTAGTGTAACCGGATTACATGGTTACACCAATTAAATAATAACATAAATTATAAAAAAATAACATATGAATAAAAATAAAAATAACAATAATATATAATTGATTTGGTTTAGATAGTGAATTTTTTTCAGAGTGAAGTGGACACGTAAACATTCCTACCTCTTTTTCACTTCTACTTTTCTCTTTTAATTTAATTTTAGGGACTTCTTGGGGCATTTGGGCCGGAGTATAACATGAAAGTCTTGATCCAGAGGGGTTTGGGATTCTGCTATAAATCGAGTTTTTTTTTTTTTTTTTAAGAATAAAGTCGAGTTGTTCTGGGTCTGGGTAATTTATCTAACTTTAGCTGTACATAAACTTAACAGAAGACGTCAAATGAGGAGGTTTCCTTCAAAATTATTATACTAAGTGCAGCATAAATGCACAATGTATGCTCCCCGAAACATGAAACTAACTACTACTTGAGTATCATTGGGATTACGACTCACTCTCCGTCTGTTTGGATCAACCTCGGGAACGCAATTATATTTTAATTATCACGTGAAATCAATTGAAAAATGCAAAAATTATACAATATTTTTTAGTGTTAATCTATTGTTTCACAACGCGTAAATCAAATACTCACAACTCTACTTTTTTTCCTTCTTTTATCCTTTTGGAACATTGACGTGCTTGGATACTCTTGGTAATTTTAAGAGTTTTGAAGTGAAAGTCTTACGGCTAAGTGCATGATAAAAAAAAAATTCATTGTCTTCCATTTCAGGGGAATGCTTTTGTTTTTTTTTTTTTTTTTTCTAAAGATAGTCTTAAGAATTGAAAGAAAGAAGTTGTATTATATTCAAAATTATAAGCTTAGAGATTTGAATTCCTTTCTCTTGAACTAATACATGTTTGAATTTTAGTTTTGCCTTCCAAAAAAAAAGATGTTTGAATTTTACTATAATATTACATAGCAATCTTGTACAAATTTGTTGAATAATATGAAGATTTCATATTTAAAATGATGTTCATGCTATTTGAAACCCTGCAATTGCATAAATTAGAAACTTATAGGCAGAAAGGTATGATATGAATAGTGTTGTAATTAATTTTAAAAATATTGAGATAAATTTATGATTGTAGCGTACGTAAGTAAAGAAATAGAGAAATGAGGTTCCGCGAAGCAAGAATGAAGGCGTACGTTGTCGCGAAGCAGGAGAGGAAGGTTCCGCGGAGGAA

>GLYMA.05G153000

ATCATTGTTTAATGGTAATATTTGATGGAAGAGGCTAAAAATAATAATAAACATAATTGAGAAATTAAGATTGACCAAAAAATTATTGAGGAACTAAAAGTGAATTTTATGAAAATATAAAAGACTAAGACTAAAAAAAATTGAGAGACTAAGACTAGTTAAAAAAAATTAAAGGAATAAAAATATTTTTTGTAAACAGATGATAGACTAGAGGTATAGTTTTAAAAAAAAATTACACGTGAATGTCACGTTACTGGACAACAAAGAGCAAAAATGTTAACGTATAAATAGTTCGATAAAAAATTTGATAAGGACTACAAGCAAACATTTGGAAAGTTTAGAAATCAAAAAGATATTAAACCCTATATATGATTTATTTGAATTGAAGGTACTTTATTAAAAAAATACTCATAATTAAAATACAATCAAACATATAAAATAAGAGATACAGATTGATTCCTATGATATTAAACCAAAATAAGATAATATCAAAATGATATAAATTTATCAACATTTATACATTATCTTAATATACATTAGAGTTTTTCTATTTTAAAATAAGTTTTATAAAATATTTTTTAATAAAACAAAATAAACAATGTTTGATGTAGATTTTATTATATAAGTTTGGATGATAAGTTTTGTAATAATAAATTTATATGTTAATAATATTTATTATATTTTAAATTTTGCTACTATAAAGTATGTGTTTATATATAATGCATTAATAATATTATATATTACATTAATAATTGTGTTAAAGTATTTGATTTATTTCTTTTAACTACTTTTAAATATATTTATGTAAAACTAATAATAATAATTGAAAATAAGCATATTTTTATATATGTAGTCAAAATTTAAAATAATTCATAATAATAAATTTTATAATCATTGAATAATCTATCTTTTCTTATTTTTTTCAGCCTTTCCATTCTATAGCTCGAAAGTTGTATACTCTCATGTTTTCATTAGGCCATCAATAATCAATCTTTTATATTCTATCTAACTTCATCATCTTAATGCACTTCAGACTTAGGACTATGTATTTTTTAGTTGTCATTGATTTAGTTTATTTCAGGATCCATAAATTCAACTATGAACCGACTCAATCCAAACATGATCTAAATATCTTATTGTCATGTTTGATTATGTTATGTCTTTATCTAATTATATCTTATCATATCTAATTAGTCCTTATGGGAACAATTATATATTATATTTATTAAATATTAATGTTATTTATATATTATATTTAATAAATACAACAATAAATATTATATAAGGAAATTCCTACGGAAAAAAATGTTTCTATAAATTGGTTTTTTAAACAATAATAATAATAATATCATATATATAGGTAATGATGACTTTTTATTTTAATAATATATTTTTAATATATATATATATATATATAAAATATGAAAGTTCATGCATTGAATAAAAGTGTAATATATTTAAATACCATTACAACTAATTATATACAAATAAATTTAATAACTGTTATAACTAATACTATATGAGAAAAAATACTTACATTTCAATACTATAATAATAACACTAAATATTATATTAATTAATATTTATTCACAAGTTTAATTCATACACATGCGTAAGCACGCATGCACATACTTACCCACGTCCACGTGTGTATATATATGATACATTATTTGATCATATATTTACATATATAATAAAATTATTGACTTTTGTATTAATTTAATTATTAGGAATGATTTTTAATCAATTGACTCACAGTTAAAAATAATTTCTTCATATATATATGTGTGATTGTGAAACATGAATTTTCCATTATATACTTTTAATGTTATTATTAAGATAATACATGACTATTAGTATTGATTATTCATTAACAAATACAATTCTTATAATATTCACTATTATTATTACATGCTACTGACTCTCAAAAAAATAAAAGCCACACACAACTCAGCACAAAATTGTAATAAATATTACCAATACAAAAGGTGTCAGAAAAC

>GLYMA.05G168200

CCTATATAAGCCTTTGATACAAATATATAAGAAAAGAAAAGATGAATCTTAATGCTACTAGATTCCTAACAATATGATAAATACCTAAATAAAAGGAAAGATAATGATATAAGAAAAGATAAGATCAATTCTAATATCACTAATTAATATATTCTTAAATTAATGCCATAAACATCATAAATATACATACACTATCTTTATTAAAAAGGAAAAAACAAAATATTAATATTCTTAATTTTTCTACTAAATCTTAATTAATTCTACAAGCATATACTTTTTGTTATATTTTTATTTTGAACTCTATATAAATTTCTTAGGACGTTAGATTTTAATACTAAAACTCTGACATTTCCTTTCGAACATGGATCCCTTTTTAACCCATAATTACACCACACTTCTACATCACTAAGCATGTCATATAATGGTCAAATTGAGTAATTCCTTTACTTGACTAAAATCTTAATCAGACTGTGAACTAAATGCATATTGTATATTTAAAATTGCTACATGTCAATTCAACTAAAAATGTGATCAAGACTTTAAAGATAAAAGAAGAAAATAGAAAAGTGGGAGATTAATTTTCTAAGGATTGATTAAGGAGTTTAACAACCCATTAAAGTAAGTGAAAACTTTGAAAACATTAAAGAAAACTTGAGAAATTTTCTTAAGGTAGAAAAAATTGTTCAAAAAATAAAATTTGTGCCCCGTAAATGTAAAAAACTATCCTATTTAAATTAAGAAAATTTGTGTTTTTAGTTCTCGTATATTTTTTAGTCAAAAAATTATCGTAATTCCTATGCTTTTAAACCGTTGAATTTAATCCTTGAACTCTTGAAAATACAACTATAGTAGTCCATATTTGCTAATTTCATTAGTGTGACCATGATGACATACAAACAATTGTTATTTGTGGCAGTCATAAAGATGTAATTCTCCCAAAGAAAAATAATACTCATAATATATTTTTAAAATTTTTGTTAAATACATTTTTAGTCCTTTTATTATTATTATTATTATTATTATTATTATTATTATTATTATTAAAATTTTGTTTTTTTATTATAAATTTAGTCCATCCACTATTGTAATGTAAAATTTTAATTCATTTATCAGTTTAATATTTAATTAAAATACAGACACGACACTAAAATATTAACACAAATACAGTACCACAGTAGTTTTTTCTCATGATTCTTGTGAGTTTTGAATAATTCTTAAGATTTTTAGTCCTTTTATTATTATTAAAATTTTGTTTTTTATTATAAATTTAGTCCATCCACTATTTTAATGTATAATTTTAATTCATTTATCAGTTTTGAATATTTAATTAAAATACAGACACGACACTAAAATATTAACACAAATACAGTACCTATCATCTCTTTGCTATTTTTATTTTAAAAATATTAAAAACAATTACCAATATAAGACCAAAATATTTGTAGGATAGATATGTAAAAAAAACAATTATTTTGTTTTGTCCGTATACAAATCATAAATCATTATTAATATTATTTTTTTATAAATATTATAAACTTTATTAAATGAATCATACATGATAACTTGAAATTTTTTTTGCTTAAAGTGACTTTTAGTTCATATATTTCATAATTATTTCTTTTTATACGTTATGTTTTAAAATTTTAATTATAATTATTTATACTTTCAAAATATATCATTTTAATTCTTTTTTAATTTTTGATTAGAAATGTTTTATTAAATTTTAAATTTTAAATTATTTGATGTCATGACGAAAAATTAAAAAGAAAAATAAAAAATTATACATTTAAAAAATATAAAGGAATAAAATAAAAAATAAAAAATTTAATATATTAAAATAAAATAACTGTAAAATATAATAAATCAAAAGTGATATTTTTTAAGAATTTTAATATAAAGATGGTTGTTTTCTGATGTATAAAAAAATACAAATAACGGTAATAGAGAGATAAATATGCAGTTACTAGTAAATAAATAAAAAAATCCACACCCGTTGACTT

>GLYMA.05G196600

TAATTACATGGCTAAAACAAAGTATTCTCATGAGCATTCATCGTTTATCATTGTCACAATTTGTAAGGACAGAAATGAAATAATATTTTTTCACGTAGGAAATAAATTTCAAATCATAATCAGGTATAAGGACAATTTTTTTAATTTAGCATAGGTATTCAAATACATGTTGAAGTCAAATCATACTCCATCAATCAGGTAGTTAGGCCGACTGACTCAGACTATGTTTGATTTATGGTGGAAGCTGAAAAAATTTACACATTTTATTTATTATTCATATTTTTTTACATTTTATTTTAATTTTAAAAAAATATTTTTATTTTCTGAATCAAACACATTTTTACGGAGTTATGTTATGAGTCTTGTTTTGTTTGACTATAATAAATGGCAAGAAATTATTTGAAAAAAAAATAATCTCGCGGACAAATATATCACATTAAAACATATATATATATATATATATATATATATATATATATATATATATATATATAAATCAAATTAAAATAAGTGTCAAGTCAAAAGTTTTAATTCAATCTACTAACTAGTAACTATTCTCATAATGATAAACAGAAATGTATATTTTTCCAGTAGGCAATAAAAAAAATGTGTTAAAGAAAAATATATGTCAAAGACTGCATGTGTACTGGCATTTCTCAACTAGGTAATGCGACATGTGCATTGGATAATTATGTAGTTTCCACAAGTTCATATAAAAATTGTTCATTTTTATTCATAACAAAAATGAAAAGTAACATTCATGTTGATTATATAAAAAATAAATTATAAAAAATGGTTAGTAAATTTTTTCAAATAAGAAAATTTTCATATAATAAATTTATAATATGAATAGAAATTGATATTTACACAAATAAAAATTTGTATTATGCAGTAAAACTATAAATAATTATAAGTATAAAATATCATAAAAAAGAAAAGCATTAAAATTTTAATAGCATCATTGTTATTTTGATTTCTTTTAAAACATTGAATTGCATAAAAAAGTATAATCCTAAATAAAATAAATAGTACTATTGAATTCAATAAAGATATATATATATATATATATATATATATATATATATATATATATATATATATCAGAGGAAAGATTGATATCTCATTTGAAGACAGAAAAATTTTCTAAATTCAAAAGTCAATAAAAATATATTACTACATAAATTTAATATTAATATATATTATTAACATAAATAATTATACATTATTAATTAACTAAAAAAGACTTTAAATATATTTCCTTTGTCCCAAAATATTTGTTTTCCTAGGTTTCATTTACACAAATCAAGGAACGAAAACCAATAAAATGATGAAAGAAAGATATAATTTTACAAAATTATTTTTATTATCATTAATTTACTTATAAATTGCGTTGTCTTTTATTAATATTATAACAAATATAAGTTAAAAAATAATATTAATATTACATTGAAAAATTAAAATGATTATTAGTATGTTTGGGTCAACTTTTCTTAGAAAAAAGTTGTTTTCAAAATAAGTTAAATGTCTGGGATGGTAACTTAAATTTTTTAAAATATATATAAAAAAAGAAATTATTTTCATGAATCTGAAAATTTATAGCTTATGGTTGTTTTTTCACGTTTTTTATTTCTACTATTTTCATACTCTTTTACTTTAATTAGTAGAATTTAATTTTTTTATTACATCCAAACAAAATTAATTATTTTTAAATCAATTTTGAACAAACTTACCCAAGCCTAAATTGAATCTAATTATAATCAATTATAACAATATTTGATTATTTAAATAATCTAATTTTTGTAGCTGCTCCAAGCACACCATTTTTATTCTTATAAAAGAATTATTTCGAGGTGAAGGAAATAATAGAATTTAGAATATATCACTACAAAAATTAAGAAATTTGAATATGATGAAAATTCAGCAATTAAATTCCTAATTCGTATGGAACAAAGTAATTGAGCTACTTTGGACAGTGAAGAATAAATGGAGAGGCTACTAAT

>GLYMA.06G005000

AAAATAGCTAAAATTTCTATTGGACAGAATTGTTTCCAGTAATCGAAAGAACAATCATTTGACTAATTGTGATTCATGAATTTCATAAGGGGAAATTTTCATCGGTGCAACTTGCTTGATTAAATCCTTGAATTTGTCCATGCTACATCCTGAAGGAATGTCAAATCTTATTGGATTTGTTCAGTTGAATGACTTGATCGGTTACAATGCTTCAACAAGGGGTAACGTTAGGGACATATGATTACAAATTTGTCTAAATCTATTAATTCCATATCCAAACACACAAAACATTTGTGGGTGTCATCGTTGGTTGAGGAGACGTACTTCAAGACCACACAACTTGGTGAATATCAGGCCACATCATGGCTGCCTGAGTAACTATAATTCTGTCAATGTTGATCCCATAAAATATGTGTTACCATTATTCACTTTACAACACATTTTGCATGTCTATGACAACTCCCTTGGGTTTACTGTCACACGAATTAATGTGACAAGAATATGAAGGAGATCAGTGAGATGCTGATCCAAAGGGAAAGAGAACAACAAAGGATCGCCCGATTTCAACTCGCATTTCTACTTAAAATGACGAAGACGAAAATGAATGGAAAAATAGAAAAAAAATGTGGAATTTGCCGGCAACATGACCATAGAAACAATTGTCCTAACATGCCTTCATCTTAAGTTTTTCTTTACCCAAATGTAATTGTTTAAGTATTTTATTTATAATATAGTATAATATTCTTCTATAAATAAATTGTTAAACAAAAAGTATTATCATTTTTTAAAAAAATCTAATGACATAAACAAACAAATTATATTTAGTACCATAATAATATTAAAATTTTTAATTTTTTTACTAAAACAAATATATTATTAGACAAAATTTAGATTTTAAAAAAATATTCACCTACAAAATATTTTATATAAATTAAATAATACAAAAAAATTAATTATATTTAATAATATTATTTTCTTATTAAACAAATTATTTTAATAAAAAAGTATTTTTAAAATCAATTAAAAATATATTTATATAATATAATATTTATATTATTTTAATAATTTAAATAAAAATAAAAATTTAAACATACAAATTAATTAAAAAAAAAGACCATACTGAAGTTGTGGTGGCCAAATCCGACTCCCCAAAACTACATGAAAGGGGTGTATATTAAAAAAAATTCAAAAATATGTGTATTTTAGTAAAACATAGGCGTGAGAAGGTGTATTTTGGAAAAAAAAAAAACCACTTTCATTTACATATTTACTTGATGTGATATATAACAAAAAAAGTGTAAGTAACACATTTTAACAAAAAAATAACATAATTAATTGACTAAATCATAAAACTCCATATATAATAATAAAAATGAAATAAAAAAAATTATTCAATATAAAGTTTGTGAAATGCAAAAAATAATTTAAGACGCCACATAATAATAAGTAAATTAAACATTAAAAAATAATAAATTAATTTGGTTTAGGTTGATTTAAATTTGAAAAGTGTAAATCCAACCCCCAAATCAATCAAAATTATATTTGAATTGTTTGATTCAGGTCAAACCTAAAACCAATCTAAGCCAATTTAGTTGGTTAAATTTGGATGATTGAGTTTGGTCGAATTGATCAGACAAATAAATATGCCTATGGTATTTTTAGGTATACTGTTCTCTATTAATTTAATTCCTGGGTGAACAATTTGGGCCGGAGTTGGTGTTAACATGAAAGTCTTGATCCAGAGGGATTTGGGGTTGGGCTATCAATCGAGTTTTTCTGGGTCTGGGTAATTGATCTAACTTTAGCTGTACTTGGTGTACATAAACTTAGCAGAAGACGTCAAATGAGGAGGTTTCCTTTAAAATTATTATACTAAGTGCAACATAATGTATGTTTTTCTGTCTGTACGCTCCCCATGAAACTAACTACTTGGGTATCAATTTACCCGGCGTGTCGTTGGGATTACAAGCTTAAGAGATTTGAAAGAAATAAGTTGTATC

>GLYMA.06G053100

TACGTTATATCTCGCAGTCTCGTGGCTTTGTGGCTTGAAGGTATCTTCTCAAACCATCAAAGTGTAAGCATAAAAAAACGCATCAAAGTGTGAGTTTGAACGAAGCATCAAAATAAAAGAAATTAGGAATTTACATGCCAATGTACTGTAATTAAGACAGTAAATAATAACAGGAAAAATATGTTTTTAGTTCACGTACTTTCACTTTCTCTTATTTTTAGTCTCTCAAGTTTTATATAATTCAATCTAGTTTTTATATTTTATTAATAATATGTTTTTAGTTCCCCCGTATGTATTACTGTGAGGAATTTTTTATTTTGTTAACTTAATAACAATGACATGAATTTTTTTTAAAAAGAAAATTATTAAAATGTCTCCCCCACATACATTGAATCCTAATTTTTAACTACTAGGTGTTCTTTTTCACCTTCATTATTCAATAGTTTGTGTTGCAAATTCATGAATTTGCACGGTTCCTTGACGATATCTATACATAGGAGTTGTGGTTCGAAATTCACAACTCTTTTCCACAACCAGACCAAAAGGAATGTGCGTATGAACTAAAAACATATTTATCCCAATAATAATTAACAAAAGAAGATCAATTAAAAATAGAATTGACAAATTAGGTTGATCAATCTAAACTTGTTCATTATGAGTTTATGGAAAATAACCTTAATTCGATTCCGGTGGAATACACTCTTAAAAGAGTTCTCACACTCTTAGAAAAAAACAATAAACTATAAGTATGATTAAACTTCAATCAAAAATTAATTTCATAATCAGTCAACAAAAGCTTGTGAAATACTTTACCATATGATTAGAGTTAGTATGGTCCTAATGATAACAAATAAAATAAAAAATCTAAACTTGTTAGTTGAAAGTTGAATTGGAGCTGTGTTAGGCCCATTTCTTTCTAGCTAAAAATTTATTGGGTCTAAGTTAGCCTTGTGTCATACTAGTGAACCACTAGACTAGTCCTAATTAAAAGTCAATAAAAAATAAAAGAATTTTTTAAATATGCAAAGTCCTATGTAACAAGCTAAGTACTGTAGGTGGCTACCCAAACCTGACCTTAATTAAAAATTGGTTGGGTCAATCTATTGGGTTGAGCTGAATTTGTTACCTCAAATTAAAAGTCATGTCATGTCAGAGATATTAAAAATTATAATACACAACTTAGAAAGTAAAGTTTTCACTTTTCTGATAACTTAAATTTTGTGATTTTAGCTATTTTTTCAAGAAAAAGGTTTTGTAAGTGTGTTTGATCCTTTTTTTTAATTTTATTGTCCTCAAAATAATAAAAATTGGGCTAAACATAACATTATACAAAAATACTTAAAAAAAACAAGTCACTTATTTTTAGAGTAAATTGTTAAAAACAAGTAGCTTATAAAATAGCCAATGAAAGAAACTCAAATTTAGCTTATCCGGAACTATTTTTCAAACGACCACGATTTGAATCCTATAAGAGTATTTATAATTAGTGTTTGACTCAATTTTTACTTAAGTTAAGCTAAAAATAAATTGGGTCAAACACACATCTAAAAGCCATTTCCCAACAAACTTATCAAAAACAAAATTTGATTTTGATTATAATAAAAATTGATTAAAACAGTAATAAATTATACTTGTTATAAATAATTTGAAATAATCTAATTTTGGCTGTTGTTCCAAGTGTCTTAATTAGACTTTCTCAATATTTTGTCTTTTTTTTAAAAATGTATTTCACATATTACTCATAATTGATTCAAAAAATTCTTATCCACTATCTCAGATTAACATCCTTTTAAGTTTTAACTGTATTTCTAAATGAAATTTTAATAATTTTTAAAAAAAATATCAATCAATAATTTATACATTTAAAGGTATATATTTATAATAGATTTTAATTTTAATCATTGTATAAGTTTTTACTCTGCGGGTTCATCAGAGAAATGATCCTTATACATTAAAAAATAACAGTGGTTTTAATTTTGTTATCACTCCTCTGAAAACCG

>GLYMA.06G159100

CTGTGAGTGATGACTAGGTCTGAGGCGAGTGAGAAAGAGTGAAGGTACGGGATTGACTCAAACCATGTGTCATGTTTGGGAATGGGCCAGGGCTAGGCCTTTAAGAGTTTTCCAGCGGCTACTCGTACTTGCAATCTGCAATGCCATTACGCGGACATAAATCCCTATTTTCGGTTCCTAGGTCGGGTTTTTGTTAGAAAATTGAAATTTCATCTTTTTCGCTCTAGTTTAGCTGCTCATGGTAAAATAAAATAATGGTTTATAAAAATGTTTTAATAGTGATTTCTTCTTGTTAGATGAGATAGTTAGGCATTTTCATGAGTCGTGTCGTGGTGTCTCGTTCATCCATTTAGTCAAAAGTTTATTTTGATAGGTTGAAACTTTAAAAATATAGTTTATTTAATTTATCAAAAAATTGTTGACATATATTTCAGTACTTTGATCTCATGGTGTGTGTTTGGTTTAGTCTTGCCAAATCCTTGAAGGATTGTTAAATCAAAAGTTATATATATTACAAAACATGTATGAAAATGTATATCGGTTAAATCAAACATGTCTTTGATCAAAGTCTTGGGATTCAATCCTTCACTTAGAGTATAATTTTTTTTTACTCCAAAATGAACTCACAAAGTGGAAAGAGATTAATTTGAGAGAATAATGCTCACACAACCAATCATATGGTTAATTTAGTTTATACCGATGAATTTGTCTTAAATTTTTAATTTTTTTTCAAAATTATTATTTTCATTAATATTTATTTTTCTTGTAATAGTTTATAATACATTACTTTTTATTCTTTTAACCAGGATAATTTATAATCTATAAATAATTAAAACAACAAATATAATAAATTGAGTTTTATAAAAAAATACTATTTTAAATTTAAATCTTATAAATAGAAATATAAATAATATTAATTTGACTTTTTTAATTTTATTTTATTTAATTAAAGCATTATTTTGTGATGTGACACTGTATGAACATTATCTTCATTATAATTATCTTTAAAAACATAATTAAGTAATTTTTTATTGATTAGTTGAACGTGTGAAATTTACACTGATTGCCTAATTAACTCACATTTAAATTCTTGCATTCAAAACCTAATTCATGTAAGTGAAGTGAATTTGACACATAAAATATGTATATTTAATTATTTATTCTTAAGTCGGGTGAATTGACGTAATAAATCTCACCATCCATATAATATCTATATATTCTTATTACTAAATCATTATAACTTTGTCATGTTTTAAAAAAAAGCTAAATCATTATTTTTGTTCTTTATCTTATTTATTTTTTTTAAAATGATTATTTGTCTTTTTAAAAAATTACTATGGTCATTTAGTTTATTTAAAAAGTTTAAAATAATTTTTATCATTTAAAAAGTTTACTTTAGTATATTATTTCATTTAAAAGGTTTAAAATAGTTCTTTAAATATTTAAATATATTTAACGTTGTCCTTCAACAATACTAAGATAAATAAATATTTAAATAATAAAATAATCAAAGTAAAATTAAAAAATAATATTTTGAACAAAATAAATAAAATAAATAATTAAAATGAAAATTTTAAAAAATAAATAAATAATTAAATTAAACTTCTCATTTCTATATATAAAATACAATTATCTAATTCATTATCAAGATTAAGAAAAATGATTAATTTAATTGATAATAATAACTTTTTCTTAAATTTATAATTTTTTAAAAATTATTTTTATTATTAATAGTTTTTCTCTTCTAATGATTTGTCAATATATTTTTTTAATAAAAAATATTTTTAATCTAAAAATAATGAATATAAAAGATATATTATAAATTGAGTGTTATAAAAATAAACGAAAAGTATTTTATTTTAAAGTTAATCTTATATATGTTAAAAGAGAGCATAATAACAGACCAAAATGATGATTTAGAAGTTTTTTTTTTCATGTCAGCGTAAAAAGAAAAAGGAAAGCAAAAATTCTAAACGAGGATTCACATGTTCACACTATCT

>GLYMA.06G208700

GGCACACAACTTCTCTTTATGATTTGGTTCACACAGCTGCCTTAGTTTCCCCCAATTACAAAAAACCCTAATGTTGTGAGTTACCATTTCCCAATTTTCCTACTTTCTGTTCCCTTTTTGCCACTTTGAAAAAAAATGCTATTTTGTTGTGGTATATTCATTGTAGTGTAGAGTGTTTGTGAAATGAAAACATCTACTTGTTTTTTGACTCATCAAGTTATCATTTTGTTTTTTGCAGTTTGGAGGCCTTCTAACAGCTGGTATCTTGGCTTTCTCTGGGACGTAAGTGTTATCTCTACCGTTGCTGTTTAAATGACTATGTTTGTATTGTACCATGGAATTCTCTTCCAAACTATTTGTTATACTTATTTTGGAACTAGAGTAACTTTGATTTATTTATTTGGGGGGGGGGGGTATAACAAAGTTCTCTTCCAAACTGCTGCTTACAAACAATTTTTAATTTGTAGAAATATAATATACTGATATCCACCAATAACCATGCACAGAAAAGAATTCCCAGATGTGCCCATAATTGGTCAAAGGCAAAAAGAGTAAGCAAGATGAGCAAATAGGCAAAGGATCGATAAATCTAACGTAAGTATTTTAGTTTATAACAATTTCTTAAGACATAAGAGAGTCATGTTATTCAAAGAATTTGTTGTAGTATATCATTATTAGTTATTGATAGTAGAAACCACGTAGTTGTGAGGGAAGTCTACACTGTGTCTGCCTCCATGTGCACCTTCCCACTTATGCAATAAATATTTTCAATGTCATTACATTTGTTTGCTTGTCTCTCAAATGTAATTATGTATGGCCCTATAAATCAGTATCACAAATGTTATCGGGTATGTTTAGAGATATAGATTAACAAAATAATATATATATCAGATTATATAATTTATCTGTAAATTTTATTATCATTTTTCTGTTGTTTAATCAATGAATTAAGAGTATTCGTCTAGGATATCCAACTACCATTAGTGGTAATAATTATGGTACAAATACAAACTATAAAACCATTTCACTAGCCTTAGTCTATGACATTCTACTCCTCTATGTGTCTCTCTCTCTTCCCTGATTGATTCATAACTTTGCCAGTTAGGCTCAGACCCAATCTGTATTAGTATTATATATCCTGGTCATAATTTTGATTGAAGCTAGCTAGCAACCTGGCCCTTTAGATCTTGGAGGAACAAATTGGCTCCACTCTTGTACCTTGGTTTCAGAAGGGTGCTCATGTTCTGTCTTGTTTTGTTTTTTTTTTTGTTTCTATTTGCTTCAGTGGTGTTTGCTCAAAACATCAACCAGAGAGCAAAGCCATGTGTAGTGGTGGCATAAGGGTCAACCCTCCAGTGAACCTATTATTCCTAAGGTCCATAAGGGTCAGATTGTTAAGATTAGAAAATTCATTTAGAAGATTCCCTTCAAGGTGGTTGTTGTTGACATCAAGCTTCAACGTTGAAGTTAACCTCCCAAGTGTCAAAGGCAAAGAGCCATATAATGAACTGCAACTTAACTCAAAGATTAATAACTAAAAGATTAATAACTCATTTAGCCCATAAAAAGCATCTGGAATTCTACCAGTAAGATAGTTTCCAACAAGAACAAGCTTCTTCAACTTCATGAGATTGCCAATGCTTGATGGTAATTCACCTGTTACATTGTTTTCTAATATTACTAGTGATTGAATGTTCTTGAGGACACCAAAACTTGAGGGGATGTCTCCAATGAGACCTCAAGTGATCTAAACTCCAGTGATTCTTGAGGGGATGTCTGCAATTTTGTATTATAATTTCTTATCAAAATCTTTGTGATTCATAAAGGAAGTTTTTCTATGTTTTAAAAAAATGAAAAATTAAAAGTTATTAATACAAATAATTTAATACTTGAATCTCTTGATCAATATTGAGTTCGATGTTTAGTTTAAACATAAAGTGATAACTGTATCCCACAAACAAATCAATCTTCTTGGTTATACTAAAAATAATATTTTTAAA

>GLYMA.08G126600

TCTCTTTATTTATTTAAAACAGAGAAGAGGTTTTCTTTGGTTTCTTTCTTTCTTTCAATAATGTTTTTCTTCTAATACAGCCTGTCAATTAATGTGAACTACTATATTTCAAGGATCAATCACTTTTCAAGGATAAATATCGTAAATCATGATATTTAAAAATATTCTTTTTTTCTAATTTAAAATTCCAAGTTACCCCACTACTCCCTTCTTGACAAATTACTATTTTTTTTTATTTTAAAATTACAAATTACCTCTCTGCTCTCTAATTAGCATTTGTAAACAGAGCATATCATTGAAAAAGCATAACATTTTTCTTAGTTTCTTTAGGTGTAGTCATCTATGTCATTGAAGATGCTCATGTATATTTTATCTTCACCGAGATATGGTTAAAGGACGAATATTACTGATTTCCATCCATGAGCTATTACTCGCTATAACTCATTCACGTATGTGAGAGAGCATAAAACTAGACAATTTAATTGTCATCAACCTTCTCTACATAATAGGTAAACCATTAGAGTTAACACTGTCAGAGTTAGCACTATCAAACAGTGTTGCACATTATTGTGACTTTCTCCCTTTTAATTGGAATACATTATATCTATTGTTGCAAAAAAACATGATGCTCATTGCAACCAAAAAATGAATAATTGGATACTCTGTCATGATAATATTTTATGTTCCATGTTTTAGTGTGCACATTTAAGAGATATACTCATATACAAGTGTACGTAGAAAGTGCAAAAAAAAATCCCAATACACCTTTACGATTCATTCACCATTGCAACAATCATGATTAGTTTTTCATTTATTTATTGATTCTGATGTTTTTCATGTGAAACCAGGCATATATTATTTTGTTATTAGTGTTGTGTTTTCTAAAATTCTCAAAGGAAAGATACCTTAGTTTCATTCCATGTGGGAATTCTAATCTGTCCTTCATTTTTTATTCAAAATTTATGCAAACCAATATGAAATTACAATAAATTTAGTTGCCTCAACAACCATGATTTGGAAAAGGGTGGACGATTCTAATATTTCCTTCTCTTTGTTCTGTTTTTTTCCATCTATTTAATCTTATATTATTTATTCATTTAAAACATAATTTTTCTTCTTCGAAATTTATGCAAACCAATATGAAATTACAAACAATTTAATTACCTCAACAACCATGTTTTGTAAAGAGTGAACACGCATTATAAGAATGGATTTAAGATAAGATTATCAAAGAAAATTGACTCAGTTTTTGCTTCATTTTGGTATTTTAATATTTCCTTCACTTATAATCATTGTTTTTATAACTTTTGTTTTATTCCTCCTCATCTATTTATGTTAAACATTTATTAACACCTCTCAATTTCATTTTTTTACATAAAATAAAAAATCTCTCAATTTTTTAATAATAAAATTCCAAACAAACCCACTACACACTATAAAATAAAACCACCGCACACTATTCTCTTCTGTTTATCTTTTATTAAACCACTGCTTTTATAAAATAAAAAAAACTATCAATTTTTTAAATTTTAAAAGTTTAAACTACCTCACTTTTCTCTTCAGTTTTTAAACTGTCCAATAATTTTAATTTTAAAACCTAACAATTAAAAAAATCAAAAAATTGTTCTGGGATTGATATTTACTAATTTCTTGACCAGATACAATAGTTTTTTTCCCAACTTTTAAAAGATCAACAATGTAAACTAACCAAAAAATTCAATGTAAACTAACCAAAAAATCCGATGTAAACTAAACTATTGTTATTTTAAAAAAAATATTTTTTTAATTTAAGATTTAAAAAATGCTACACATAAACGATGCAGCACATGTGTTTCCTAGTTGAATATAAAATTGGTTGTTTTCTTCAGATTGTAAAAAGAATAGAAATAAAAAAGCAGTTATTAATAACAGTTTATTTTATTTCCATTCCCGTTGACTTTTGAATTGGTTTTGATCCGTGGGTCGATAATTAAAGCGTTATTGGTCCTCAGCATATAAAG

>GLYMA.09G019600

CGCTCTATTGTGCTTTATGTTATATGCACATTAGAAAGTTCTCCTGAACAATCATGCACTATAATACCTGAATGAAAGGATGAGCATCGGTTGGATTCTGCTAAAAAAAATCAGATTTAGCAAATAAAAAAAGTAATAATTTTTGAAGTCCATGTCGGCGGTTTCAACTAAAAAACACTTGAGAACTCATTAGTTATAATTTTCAGATGGGTATTGCCATCCCGTTCGAAAACTAAAACAGCATTTTTCGGTAGTTTTAATGGGATGCTCATTGTTTTTTGCCTGTGTATTTTTGAAGTTGAAAGAAATGTGCTTTTAAGTTTTCTAATGTAACATTAATTGTTATTTTTTATTTATGTTTCTTATAATTTTAATGATACGAGTAACAAAAATTTAAAATAAATTAATGATAATAATGTTAATTTGGTAAAATTGGTATTTTTTCATTCATTTATTAATTTTTTAAAATTTATATAAATTAACCTAGGATGATAATTATAATGAAACAAGCATCTTAAAGTAAGTATTGTCTTAGTTTGTTTTAAATAGATAAAATTAATAAATAGATTAAAGAGAATAATAGTTTTATAAAATTAATTTTATTAAATAGATAAAAAATAATATTAATATTAGATTCAAAAATTAAAGTAATATTTATTAGAAGCATTAATGAAAAAATAATTAATGTTATATTGAAAACTTAAAGATAATATCATTTATCTATTTAATTAATGATTCATTTCCTCTTTCTATACAATATTTTTACTAAATAATGATTAAAAGTAATCTTGAAAAATATTAATATTTAATACATTTGTTTTATAAATGACATTTATTTTGCAATAATTTTTTTCTTCCAAATCGTCGTAGAGTAGTGAAATGTAAAAATAAAATAAAATTGTATAAACAAAAAGTAAAAATAGATACAGTGATGGTTTGTGACTTGAAGTCCCTCTCAGCATAATGCATCCATCCAATCCACAGTTTACATTAATCATAGAGTTGAAAGTGACTTTGGACAGCAAAGCTCAAAAAAGGAAAAAAACAAAAGAAGAAGAATAGATGCTTGAGCGCGTAACAAGTTAAATTGGAGTCAAATAAAACAACAAAGTGCTTCGATTAAGTTAAGATATGTGTGTGAAAATAACGGATTTATAACTTCATATGTATCGCACAACAAAGTGCTTTTATTAATTTATTAATATTCGTTTGAGGATTGGGTTTGGGATTGAAAGACACCACTCTCCACTGCCCCTGGTAATTTCCTTTTATTCCTCAAATCTTCCAATTATTATTTTTGTTAACAGTAATGATATCAATGCCCGAGTCTTCTGAAATATGACTTAATACAATTTAGTTGTGGTTTTTATAACTAATAAACATGCTGTGTGCTTTTATATATACTTCTGAGTTGAATTCTAACTTGGAAAGTATCTGAGTGATCCCTGAATTGCATCGTTACAATTATGCTTATTGTTGTGTTGTGTTCTTAGAGCCATTAGGAACACTGGAAAAAAAAAACCCAGTGAATCAACCTCATTTGAAGGTTCGTTGATTTTATTAATTACCCAAACTTGTTTTCTCTTTTAAACGAGAAAATAGATTGCATGACAGGTTTATTGCTTTTGATGCGATTTGATGTTTGACTGGTTAGATTTAGATCATTATTGAATGCTTTTCTTACATAGTCTCCTACTTTGATTAACTGCCACGGCAAAATAGATTGACAGTTATTATGTTTAAATTGTTTTCATTAATCTTTCTGTTTTTTTTCCCTCTTCTCACTTTATTAAGAATATCTGTACTATCCCATTCTAAAATTAATACCTTTTCCCATCAAATTGTAGCATTGATTTGTGATAAGTCGAGGAAGGTTGATGTTGATGTTCAAATCCTGGCTTGGAATCAGAAGTGTAACTTTCCTCATATAAGAGTGGTTTTTTTTTTTTGGTGAGGTGTTAACTTGAAAGCTCTATGTTGGCTTCATTAAGAGCAGAAAA

>GLYMA.10G180200

TTATCTATTTTTCGTTTTCATAAAATTATAATATGTTATTTTTGTACCTAAATCAAGTTTCATATATCCAATTTACAGAGAAAATTTCATCCTCATAAATATTAATAAATATATAAATTATATTTTAGATTTATAATAATAAATCATATTTTTAAACAATAATAATTTAAATTATTGAAAATTCCTAAAAAAATTGAAATATAATTTAAAAATAAAGATTAAAAAGATTGATGGAAAGAAATAACATAAAAAGTAAGGCTAAAAATAAAATGAGAATGATTTGATATGATTGGGAAATAAACTTTTTATGTTAATCATTAAATCAAATTAAATCTGATTTTTTTAAAAAAAATATACGTGAAAGAAAGAAGAAAAATTAACACTAATTAAATACTCAATATATAAAATTAAGATAAGACATGTGGTTATTGTAAAAAAAGGAAAAGGAAAAAGATAAGACATGTGGTTTAAGGTGAGAGAATATCTCTTAAAAATATTTTCGTTCAAAAATAATTTTTAAAGATGCTTTCATAGTTTCATTCAAGAATGATTATTCATATTTATAACATAGAGTAGATATATTTATAGCAACAAATCCAATATGTATTATGATTATAATTTTTTTCATAAAAGTAAAACTTATATAATTTGATGTGGCAATGACTACTAGACCTAGTGGCATGGGTACCTATTCATAACGGGTAGGGTAAATGACAACAAAACTTGAGAGATATATTACGATTTTCAATTCAAAATAAAACATTACACTCCTTAATTTTTAAGAGATAAATCTCATTCTTTAATAATTCACACACACAATAAATAAATCATGTTCCTTACAAAGATATATCATGTTTATTAATTCATTAAAAAAATAAGTTTCTTAATTGCAAAAAGATTATGTTTTGTCAAAGATCTTCAAATAAGATTTTAAATTTTACGATATTTGATAATCATGATCATGTTTTATAAAAAAATATTTGAATAAAATTTTAATTTAATTACTATCATATTTTTAATGTTTTTCATTTTCATATTAATTAAAATGTAGATAACAAAAATGATATGAATATTTAGATACTCAACAAGAAAATACATCAGTATTAAGAATTTTATCCATATAAATATATTTTTATCTTAATAGTCTGATTTATTAGAAAAAAATTAATCAAAAATTATTTTTATCAAAAATTAAATTTAGACAAGATGATCAACTTCAACACATTAATTACTTATATTTAATCTTTTAATTACATCCACGTGGATTTAATATAGAGTTTTCTGAAAAGAATAACTACCAGGAGATGCATTATAATATCTTATCTAAACTTTATTTATTTTTATGTTTAAATTTAAAATTTAAAAATATTTTATGTTAATATATTTTTTTGAACGATAATATTGTTAGGGATCTGGCTTATCTAATTTTCTTTTCCAAACTGTCCTGCCTATTCAAAATAATAGAAAAATCAAATTATGTTAATATTTAATTGAGAAACCAATTTCAGTGTACTAATGAAAAACGTATTTAATGAGTCAACTAATGAAAAAAGTATTTAATGAGTCAACTAATGGAAAAAGTGATGCAGTTGGTTGAAAAGAAAATAGTAATCAAGTAAGCATAATTCAACATTTATTTTTATTAATACGATAATACCCGTGAAAAGAGGATAAAGATTCCGAGAGTGAAGTCAGTACTAAAGTTTTTGATAAAATAATAAAGGAAGATAAAAAAAAAACCATGAAACGGGTAACCGTAACAAACACTTCAGCCAAAGTCAAGAAGAAGGATCCGCCGTACACTGTGTCAGGTGGATCAGCGAAGCACAGGATCACCCACTAACCCTACCACTTATTTCCTATTTTACCCCTACATCAATTTCGGTGGCGTCACCTCATTGGCCCACTCTGCAGGACCCCACCATAACAACCCTGAGCCGCCCCGTACTCTATTTACCGCGCACGTTCATATTTAAGTGCTTCCCTTTGTTGTCCTCATT

>GLYMA.11G105300

NNNNNNNNNNNNNNNNNNNNNNNNNNNNNNNNNNNNNNNNNNNNNNNNNNNNNNNNNNNNNNNNNNNNNNNNNNNNNNNNNNNNNNNNNNNNNNNNNNNNNNNNNNNNNNNNNNNNNNNNNNNNNNNNNNNNNNNNNNNNNNNNNNNNNNNNNNNNNNNNNNNNNNNNNNNNNNNNNNNNNNNNNNNNNNNNNNNNNNNNNNNNNNNNNNNNNNNNNNNNNNNNNNNNNNNNNNNNNNNNNNNNNNNNNNNNNNNNNNNNNNNNNNNNNNNNNNNNNNNNNNNNNNNNNNNNNNNNNNNNNNNNNNNNNNNNNNNNNNNNNNNNNNNNNNNNNNNNNNNNNNNNNNNNNNNNNNNNNNNNNNNNNNNNNNNNNNNNNNNNNNNNNNNNNNNNNNNNNNNNNNNNNNNNNNNNNNNNNNNNNNNNNNNNNNNNNNNNNNNNNNNNNNNNNNNNNNNNNNNNNNNNNNNNNNNNNNNNNNNNNNNNNNNNNNNNNNNNNNNNNNNNNNNNNNNNNNNNNNNNNNNNNNNNNNNNNNNNNNNNNNNNNNNNNNNNNNNNNNNNNNNNNNNNNNNNNNNNNNNNNNNNNNNNNNNNNNNNNNNNNNNNNNNNNNNNNNNNNNNNNNNNNNNNNNNNNNNNNNNNNNNNNNNNNNNNNNNNNNNNNNNNNNNNNNNNNNNNNNNNNNNNNNNNNNNNNNNNNNNNNNNNNNNNNNNNNNNNNNNNNNNNNNNNNNNNNNNNNNNNNNNNNNNNNNNNNNNNNNNNNNNNNNNNNNNNNNNNNNNNNNNNNNNNNNNNNNNNNNNNNNNNNNNNNNNNNNNNNNNNNNNNNNNNNNNNNNNNNNNNNNNNNNNNNNNNNNNNNNNNNNNNNNNNNNNNNNNNNNNNNNNNNNNNNNNNNNNNNNNNNNNNNNNNNNNNNNNNNNNNNNNNNNNNNNNNNNNNNNNNNNNNNNNNNNNNNNNNNNNNNNNNNNNNNNNNNNNNNNNNNNNNNNNNNNNNNNNNNNNNNNNNNNNNNNNNNNNNNNNNNNNNNNNNNNNNNNNNNNNNNNNNNNNNNNNNNNNNNNNNNNNNNNNNNNNNNNNNNNNNNNNNNNNNNNNNNNNNNNNNNNNNNNNNNNNNNNNNNNNNNNNNNNNNNNNNNNNNNNNNNNNNNNNNNNNNNNNNNNNNNNNNNNNNNNNNNNNNNNNNNNNNNNNNNNNNNNNNNNNNNNNNNNNNNNNNNNNNNNNNNNNNNNNNNNNNNNNNNNNNNNNNNNNNNNNNNNNNNNNNNNNNNNNNNNNNNNNNNNNNNNNNNNNNNNNNNNNNNNNNNNNNNNNNNNNNNNNNNNNNNNNNNNNNNNNNNNNNNNNNNNNNNNNNNNNNNNNNNNNNNNNNNNNNNNNNNNNNNNNNNNNNNNNNNNNNNNNNNNNNNNNNNNNNNNNNNNNNNNNNNNNNNNNNNNNNNNNNNNNNNNNNNNNNNNNNNNNNNNNNNNNNNNNNNNNNNNNNNNNNNNNNNNNNNNNNNNNNNNNNNNNNNNNNNNNNNNNNNNNNNNNNNNNNNNNNNNNNNNNNNNNNNNNNNNNNNNNNNNNNNNNNNNNNNNNNNNNNNNNNNNNNNNNNNNNNNNNNNNNNNNNNNNNNNNNNNNNNNNNNNNNNNNNNNNNNNNNNNNNNNNNNNNNNNNNNNNNNNNNNNNNNNNNNNNNNNNNNNNNNNNNNNNNNNNNNNNNNNNNNNNNNNNNNNNNNNNNNNNNNNNNNNNNNNNNNNNNNNNNNNNNNNNNNNNNNNNNNNNNNNNNNNNNNNNNNNNNNNNNNNNNNNNNNNNNNNNNNNNNNNNNNNNNNNNNNNNNNNNNNNNNNNNNNNNNNNNNNNNNNNNNNNNNNNNNNNNNNNNNNNNNNNNNNNNNNNNNNNNNNNNNNNNNNNNNNNNNNNNNNNNNNNNNNNNNNNNNNNNNNNNNNNNNNNNNNNNNNNNNNNNNNNNNNNNNNNNNNNNNNNNNNNNNNTTTCAACAGCATTTCTGTTTTTATGTGATGCA

>GLYMA.11G255400

ATTATGATAAAAAAAAATTACAGGAATTATGACAAAGCATTAACGGGTGTTTGGTAAGAGAGAAATTCTTTCATTCTCGAGAATCATGTTTCTTGAGTATGAATAAAATTTGTATGTTTGACCATTAAGAATTTTTAGATAAATTTTTTAAAAATAAAAATGTTATGAATATTTTTATTAATCATTTTAATTATTTATCACATTATGTAATTTAATAAAGAATAATATGATATTTTTATTGTAGTACAAGAAAAATAAACTTGGAAAAATAAAATTTTCACCTCTTATAAAAGTTTCCATGGGAATCTGATTAAAAAATATTTTTAGGAAATCTTCTTTCTTTAAGAGTTTTTTTTTAAATGCATGCCAAATATGAAAAATAAGTTTTTCAACCTATGATTCTTTAGAAATAAGTAAATGAAAAGGGTTTTTTTGGGGGGAAGGAAAAAGGGTCTTGCATGGTACATAATATGCTTTCTAGATATATAGCTAATGTAGTTATTATAATTTTCATTTTTTTTAAGATTGTAAGGTTTCGATTAATTTTTATTTTATAAGTAAGTTTTGTAAGAATAAATTTAAGATAACTTTATCTTAGATTTATTATTAGATTTCTAGGTGCTTATTTCTAGGCATGAAAATGTTGTTAGATTTATTCGCGCTCTAAGTGTTTAAGGAATGTGTTAAATGATTTATGTATGCAATAGACACATGAACCAATGGAATAGAGAGAATTAATTAGGTGGAGATAATTGCCATGGTAAAATAAATTTTTCTATAACATTGTTTAGAGTGTGTTGGACAAGTTTCTAAAAAATTATTTTGTTATTATATAAATTTATTTAATTAAAATGAAAATATTTAATAAATGACTTCATCTTATTAAATTATAGCTAAGTTTGATAGCGTTTGAGTTGGGAATTTATAGGTTAAAGTCGAGTCAAATTGATCTAAATTTTTTTAGACTATCGTTTTTAGAGTCTAACTTGACTTGATGGGCCATCATGCTAGTCCATTAGACTATGTCAATTTTGATAAATCTAATTATGATTTTTTATCATAATAATTTATGAGTAATTTGAATTCAATAAATTAATAACAAGTAAAAAAAATTAATGTCTACACTCTTTGTATGTATAGAATTTTTGAAATTTTATTTTACCTCTTGCATGCTTATAATAATTATTGAAACAAGAAATATATCAATGATGATAAATTAATTAAAAGATTTTTTATGAAAGAAATTAAAAGAATTAAAGTCAACAAAAATATTTTTTTCAGCAAACTAAAACTATATTAATGTGGGTATAAAGATACCCTAGGATACCCTAGCCGCATACAAAAAAAGTAGCCAAAACAGCCTTGCAATAGAATATTCCTCCAATGGTTACAAGATATTTAAAATGCACTATGCATGTAAATATTTAGTAACCATTAACTACCCTCATCAGTAATGAACTCTTTAAAACCCATACCACCCTCCTAAAAATAATGTACCTACCAAAGTCAATAGAAATATTAAAAAAAAAAGAATATGCTACATATATATATATTTTTAATTTTTATTAATAAATATTAATGGTTAATTTATTAATTTTTATTAGTAAAAAATTTAAACTTTTGACATATTAATTCCTCCCTTTTTTTTTACCTTTTATCATCAGGTCAATCTTCTATTTTTCATTTATGTTAAAGATAATTTAATGCTATATACAAAATTAATTATTTTTTGTAATTCTTATGAAGTTAAGACAAATTTAATACACAACCACACAATGCGGCTGGTACGTAGACCAGGTAAATAAAACAAATATTCACATGTTAATTAATGTTATATTTTGAATTTGAAGTAGTTGGCCGATAGACCAGTAATCAAATAAAAGTTGATAAAATTTGTTTTACGTGGAGTTTTTGTAATTTGCTAAAATAAAAATCAAAGTCACGTGATGAGGGTGGAGACTGGAATTATATAAATAGCTGGTTGTGTATGGCATTTGTTT

>GLYMA.12G030100

TACTCTGAAAGTTTTTAAAATAATACTCACTTATTAACAACAGTTAATGCATTGTTAACTTTTGTTGAATTACATGAGACATTAAAAAATTAATTAAATTATTAAAAAAATATTTGATAATATATAATATCTTGAAGTGGGATTCTACATTTTATCTAAAATTTTAAATGAACATCTATAATTTGACAAAAATGTCTCCACTATTAATAAATAAATGTTTATTAATTTTCTCAAATATAAACATATAAACCCTCAGCTAAAATATTTTAAAGTCAAAATTATTTATAAATTCAATAAAAACATATAAAATATTTAATTTATGCATGTGCTAATGCTATATCAACGATTCATAGGGATTGCTACTCTTGATCTATCTTTCCTTTTTTTTTTGGTTACATCAATCCATTTTTATATCATAATTTTAAGTATTTTATTATCGTGATAATAATGTTACTATAAGTTATCGTCTTTCTTCTAAGTCACGAGATTTCTCTTATATTAAGTATTTTACTAATGTTGTTTTAATATTTCTCAAATACTATTATTTATATTAATTTTAATCCACCATTATGTGGTGACTATAAATACAAATTCTTAAACAAGTGTACTTTTAGGAGCTGTTATTTTCATCGTTAAGTGATGAGACTATTTTTTTAATTAAGTATCTCATTTATTTTTAATTATTTTTTTTTTATTTCAAATATTGGTACTTAAATTTAATAAAACTCAATTTAAATTTTAATTAAATTTTAAATCATAACAAACTGTATTAAAATAAACAGAATCATTTTTAAGATTATTGTAATATTAGTCAATCTTAATTTAAATTTTATATTTTTAATTTTGAATATTCACACTCCATGTCAAATAAGTAAAGATGATACTTATAGGTTTAGTTTAGTTAATTGAGCAAACTCTTTATATCTGTTTATTGTTTTTCTCAACATATGTTTTTCCATTCAAAGATCAAATCCATTGTGATCATCATGTTGGTAATTTCATTATTGTTTAATAATAATAATAATCTTTTTTACTTTAGTGCTTTTTAATTTTTTTTAAAAATTAAAAAATGTTTATGCCGTGCAACCAAGAAAATGTAGCTATTATAAATAGGAAGCGTCACTGTTGAATAAAAAGATTATTATATATTTTTTTCTCTTTTTTAATAAGCTTCTCGTCACTTTTTCTTTATCATTTTTTTAATTTGATTTTAGTTCTTTAAAAAATTATTTGTTAAGTTCATGAGTATTTATTTAAATTTAATTTTAAATTTTCACACATTATATTAAAAAGTAAACACAACCAAGAAATTTAACTTAATTGTTTGATGGATAAGTTATTCGAAATTTTCGACGGATATAAAATATATAAATAAACCTAAAGATTATACATCAACCTGAAATATAAGTTATGATTCTTTCCAGGAGAACAACTCCTTTTATAAATAATTTTTGGCTTCAACGTTTAAGAATGATGTGAGACTTTATTATTTGAGATATTGTATATATTGTTTAAGTATTCAATGAACCCCACAATTTTGTTGTTATAAAAAATGTCTGGATGGATTTAGAAAAATATATTATTTATAAATAATATTAATCTTAATTTTTGAAAGATACAAGATTTTATGATTATTAAAATAATTTGACAGATTTATGTTAAAGTCAAGGAGTGCACTCTCATTTTCTAAAAATTATAAATAAAAGTATAAAAGTTCTTTTAACCAGAAAAATATAAATAAAATCTTATAATTATTATTTTATTTAATTTATATTTATATATTAAAATTTATTGATTTTAATTTTTATAATTTGCTCTCACATATTATTATTAGGAAATATAGATGATTATTTTTATTACTATTACTATTATTTTTGAACTTACGAAGGAATAATAGATTAGTTTTACGTCCATCAGATAATTATCCAAAAAAATCATGAATGGGATATAGGATAAGAATAAGAAAGAACATCACAATTTCCATTAGGGTTATCCTTGCA

>GLYMA.12G168000

TTTTCATGTTATAAAATTATAATTTTGCCTTGAAAATTTTAATCTTTCATTTCACCTAAATATGAAAACAAAGCTGTAGTATTTTTTTATTTTTTTTGAGGAAAAACACATCAGGGTTTGTCAATTAGATTAACAATTTTGTAAAGCACAATTGAATCTCTGTCCCAGACCAAACAATACAATATTGACTCCCGCAACCATCTTGTTTTCACCTTATTTATATTTCCATATATTGAAAACACTTCAACACATTTTGAATTCGATGCTTTCTAGTTTTCTCCAAACTTCAACTTTAATCATAACATTAGCTTGTTTAACCACTCACTTATGCCTAAATTAAAAACTCGATCTATTTGTTGGCCTCAATTTAAGTATTGTATCTTATCTATTATTGCTTGCTCCTTATTACAAATAGTTTTTCTTTGACAACTTACTATTCAATGCCTTTGTATCTTCTTCATATTTATCAATGATCCTAACTGAATTACACCACTGCCATGAACAAAATGGGAGGGGAGTGTTCATTGTCTGCATATGCAACCGTTTCTTGCATGTTTGTTTTTCCATCACATCCACTTCGATCCGCGTCTAAATAGAAGTTGATTTGTGTAGCTTTTGCGTTTATGTTGCTATTTGACAAGCTAATTTGTGTAGTTTTTGTGTTTATTTTATTATTAGATGCGGTATCAAACATGTTTTTAGTATTTTGAAAGGTTATGTAGCACTTGAATTATGAAAATGTCAAAAATATATTTTTGAGTACACAACCAATGATTTTTACATGGAGTAAATAAACATATTTTTATATGATTAAAAGCTATTTTACAAATTCAACAAATTTCGTTCCCATACTCTTCATACCAGTTGCAAAAATTACAGCTAAACCATACAGTTCAACAATAATAAATCCAATGCTCTACAACAATGGTGGATAGGTACAACACGTGTCAGGTGGGGTGCACTTAGATTGACCTAATCAATGTCTACATGTTTGTGAATTTTGCTATAACCATCATTTAAAAAAACATTTTTTTTAACCAGGCTTAACTCACAAAACTGTGTTTAGTATTAGTAATAAAAATGATAATTCATTATTACTTTATTTTTTTTACGGTTTTCTTACTTTATTTGTTGTATAATCCTTTAAAAATAAAGAATGAACGTGATGTTTCGTAATTACTAATAAAAATGAAATCAATAATGCAATTAAACCCATGAGAAAATACAGGTGCACCCAAAACTTGTTACCTTGTCCCTTTAGCCTTTAGCTTACTCGTTTAAGTGAAAAGTCTACTTCATTTCATTTATGGACGTTACTCAATCAAACATAAAAGTGAAGCCAAAAAACAGCATAGATTTTTTTTGTTGTTGAGAAAAAACAGCATAGATTTGATCGACAATTTTGGATCCTAAAATAAGAAAAGGCGGCAAATTTTCTTTTATTATCCCCATGGGCTTCAACAGCCGATTATAATTGTTTTTAACTCGAAATTAATCCGTAAAATTCGATGTAGATAAATTATTATTATTAATCTTTTTAAAATTACAACTAAGAGCATCTATGTACATAATTATGTAAATCAGTTGTTTATTCTAAATTTATGAGTCTTATAATATTACATAAATTTTAAAATTTTACACAAAAATTTAATTTCAATATCAAAGTTGATAGATAGAGTATTTAACTTGAATTAATTAAAAAATGATTCTTTAATTAATAAAAATAAATTAAGCAATAATTATTTATATAACTCATGCACCCTCAAATAATCTGCTTGAATTATTAAAAAACTTGATAAGTAAAATTGTTTAAATTTAAGTAAATCATGAAGAAAATAGAAAAAGGGTCTCCACGATAAAATAACAAAAAAAAAAAAAAAACGTTTGGTATTGGTTGTGCGAGAGATGATATGGCAACAATAAGATTATTCATATTACTACTTTTTTTGACGAGATAGTGATATTCATATTAATCGTACGATAGAGATAAAAACAAAAAA

>GLYMA.13G368400

TTAATATTCACTCTTTACACTTTCAGAAGTGTGAGCAATCAATCTTCTGATCCTTGTTATAGTTTTCTAATTAAATTTATTGGTTGTTATATTTTGAAAGACTCTCAAATCTTATTGGGCTTTAATGCGCAAATCTTGATTGATTTTCAAATTTTAATTTCTTTTGGCATTTCCTTCTTATTTAAGTTATTATTTGGGCTTTAAAAAAGTAATTGAAGTGATCTAGGTGTTTATGGAAAGCTTAAAGAAATGTCTCCGTTTCTCATGTAGAGGGTGTGAGAAAATTTAACATGGATCATGAATGAATTTGGACTTGAACTTTGAGCATTTCTGGAGTTGTAGAAATCAAAACAACATCAACTTGAAAATTCAACTGTCTTTTTAGAACCGTAGTCTACAGTGTATGCCATTACTTTCATTTTTTTATTTTTTTAATGATGATTTTGTATATAAAGTCTTTCTATACAAGGAGAATTATATGGGGGTTTGGGACTATCACATCACCTATGGTTTTAGCATGTAATTATGCAGCACGTGGATGAGAGATACAACAACACAGAAAATTAAAGCTGTTATCTTTTAACAAATTAAATCAAATTTAAAAATCCCCCTTATCAATTAATAGCAAATCTTGAGCAAATCATGTTTAAGATTGAAATCCAATTTAAAACTTCTAAAAATAACTGCTCCATCATCATCGTTCCGCAACGCTGAAAGATGACAGCCCCTGCCATTGTTGTACCAAGAAAGAACCACCAAAATTGGATTATTGCTGATGGTCATGGCAAGCAAGAACCAACAATTAATGTTTGGTACATGATGTCTGGAGGTTCAAACTTTTTCATCTTCAAAATTTGATGCTGAAGGAATAAATTGAAAGGTTGACTTTGTTTGGAGAATAAAAACCACCTTGAACTTTTGTTAGAATCCACCTTCAGCATCTTTCTGATTAATTAAGAGAATAAAAACCAGGAGTTGCATCAGTTCACAGTTTCTGATTATGTTTCCGGCGACATGGAAAAGAATACACATGATGATGGAGCAGTTTTTTTTTTTAAAAAAAAATTGATATCAATCTCTCTCTTTTTTTTATGGAAATATATCAATCACTTGAACAACTAGATTTGCATAAGATTTTCTGTTTTAATTGATTAAGAAGAATTTTAATTTTGATCTAATATAATTTAGTTGAAAGATAATATAATTAATTTGTATCTGCCTAATGTTGCATTTTTCATCCACATGTCGCACAATTATTGACTAGGACCATAGATGATACAGTGGACTAACATGTAATTTCTCCTATACAAGACTAATGCCGGGCTACATTAAGAATTTGTTTTATAAATTTGGTTTAATTCAACGCAAAAATTTCAATGACTAATTTATATATAACTTTTATGAAGACCCCGCTTAGCCCATGAGACCATTCGGAGGATCAAATAATTCATGATAATAGCATACATGTCTTACTTTGCTTTGCTTTGCTTTTGTGGAATTAATTTGTAATCGAGCTAACTAATATCTAGCGGGTCCATCCCTTGCACAGGAATTTATTCCCAACCCAATGGCTTGTTGTAAAATAATTAATAAATAGAAATGGTATAAGAAAAGTTGAAAATTTGCATTCCTACAGCTGTGGTCGTACCATCCATTTTGTACTATACCAATTTTGTAAATCCTTTTTTAATATTATCATTTAATAGATAGATATGACAGATTTGTTAATTTTAATAATTATCTTAAATGTTATATTTTTTTTCATTGGTTGATACTGGTGTAAAACAAATTACATTCAGATTCATTGAAATTAAACTCAGTTGATTAAATGATAAAAATATGCTGGATAAACTAATTTACTATTTTGCCCATATAAAAAGATAAATCACAGAAACCTGAATCTGGGTGGATGAATCTTATTAGTGAAATGAAATGAAAAATAAGAATAAAAATAGCCTAAAAGTAAAAAGGTTACGTTTATGTTTTTGTTTTTTGTTTTT

>GLYMA.14G033700

TGATATTTCTTATCTATCAAGTACCAAATGTTAATAATATATTTTTGAATTTTATTAGTTCTTAAAATTCATTTCCCCTTGACATCAAGAAATCCTCACCCACCACCTAATACGAAACAAGTGTCCATAGGATGTAAAATTTAACCCCATGTTACATTTGGCTACCATGGAACGATCCCCATAAGATGCTTCTTCAACATTTTCTATTGGCCAGCTCAGGAATGCAACACGTGTACCCTCTGTTCAATCCCCTGCACCACTCCACACGGGAGCTTCATGTGCACATGCACTGCACACTCCTTCAAGTCAACACGCATATTTACGTTAATTTTTTCGACACCATCATAAAAAAATGCAGCACGCGGTACTATTTGTATTATTTTTTAACTTTGTTCTTTTTTTATTTATTTATAAATAAATGCCACAACTTGTAATTTTTTATAGGATAAAATTAGTGGATTATTTTAGGCAGTTTTATTTTGATGATGTGTGTACAACCACGGCCTTGTTATATATATTAGACCAGGTTGTAAGGTCCCCCACTTGTTATTTTAAATCAATTTTCAATAGTATTAATTATTGAAGTAAAACTTTATTTTTAATTAAAAAATATTATATCTAAAATATACCTAGATTTTATCCTTTTCTCTTTCAAATTAGCCACATCGAATTTTACTTTTAACTAAAACAACTTTTATTCTTTATGAAATGTTAAATAAGTATATATATATATATATATATATATATATATATATATATATATATATATATATCATATTATTAAGAATTTTTTTTTTCATTACTTTGAGTTTCAGAAATTCACTAATTTCTTATGTAAGTAATGAAAAATCCTATGCAAACCCAAAACCAAAAGATAAATGTTAACTACTGTTTAAAGAAATGAACATAAAAAATATCAAAAAATATATTTCAAATTAAATGTACTAAAATTATTTTAAAAAATAATGTAATTTTACTACATTGATTAAATTTTTTCGTTAAGTAGGAAATATTATAAGTATTAAACAATGACATATATGCTATCACAAATCAAGGATTCTATTATTAATAATATTTATCATTGAATAATTAAAAATATTAAATAAAATACTCCCTCATCCATTCTTTTACAATTATCGGTTGTTTTACAAGAAAAAACAATAAATAAATAAAAAAGAATAATAGTTTTACAAAATTAACCTTATATAATGGTTAATTCATTGATAGATTTTATTGTCCATCATTGATATTAAATTAAAATATATTTTTGATCTCTAATAAATATTAAAATTTTGTGTTCATTTTTTAATAATATTTTTTTTCATCAAGTCTCTAATAAAATAATAATTTTATTTTTTATTTTTGACATTTTTTGTTAGTCCCTAATAACGAATTTTATTTTTGTTTTTTGATAAATTTCTTCATTTGTTATTAGTTCCTTTAAAACACTAATAATAAAAAAAAAATTATCAATAAAAAACAAACAATCATAAAATTCATTAATTTATTATAAACTAAAAAAAATGTCAATGATTACAAATAATTTTTTTTGTTTTATCATGGACTAAAAATGAAGACTTTTTTTAATAAGAAACAAACACAAAAGTTAAATATTTATCGGGAATTATAAACATGTTTTAGTTTTAATATTATAAAAAATATAAATAAATAAATATAATTAATATTACATAAAAAATTAAAATAATAATTATTTTAAGATAATTTATTTATATGACAATTATAATAAGATGATGAGAATACATTCAATATCCCAATTAGGTAACATTATTAAAAGAATTCACTTGACCATGTGAGGGCAAAGATAATTCAATTAAAGGTTGCATTAAAAACTTTGAAAACATGAATGTTCCCCCAAAGAAAAAAACGATTGGATTAAAACTTCATCTTTGATTCAAAGTCATTGTATTCATTAATGTCCGCAATAAATGCATAAATTCTGAGCATACTACTATAAAAAAAAAATGTTAAAAATAACAAAT

>GLYMA.14G097400

TCCTCACTTATGTATTTTATTTTGAGAACTTGAAATCTTTGACAATTTGATCTTCTTCCAACAATTTTAATTTACTGAGTATATGTTTAGAAGGACGTTAGGTGACCGCGTGTACGTAGTCTAATATAAAAACTATGCCAAAATATCAAAATCACACAAAAGTCGAAATGATATGCATCTAAAAAATGTAGACTATATGCATGTTTGGATCTTTGGCGATTTTATTGCAGCACGAACTATAAGACAACTTCAACGTAGAAACAAGAAAGAAAATATTTCCTGAAACGAAGGTTGATACTCACAATTCAAATTAAACAAGCTCTATATGAATAGATAAAAATCCTATTGTAGAAAATATAAATCAACCTAAGAGGCTAAGACAACCTGGAAACATGAATGGTTCATTCATTGACATTTGAAGTTGCTATGCTATGAATGTGAATCCACTCATCACTTATATTATTTAAACAGTCTGTCCTGTGTTTTTATAAGCATTTGGCACTTGTCATGTACCCCAGCAATAGTAGAAGCAAAACTAAGTAAATTATTAGAATCAATTAACTGCCAGGTGATGTGGGGACCACTGCACTAGGCACCACCAACCAGAAGAGGATATTACTTTTACATCAAGTTTCAAGTTTCGTATAACTAAAATTCTATGCCCTACTTGGTTTATGATTTTTGTAATTTTAAATATATATTTTAGTGTACATAACTTCTATTTAAATATATATTTTAGTTTCACATCAGGTTCAAGAAAAATTATGTTGTCTGACTATAGTACAGTCTATTGCATTTGGCTGTCTAGGAACAAGTTGATTTTTGAAGATTATCAATTTTCTATAATAGAGGTTATTAGCAAGATTAAATTTTTTATGTATAGACAAACGCACATGTTGTATTTGTTTTAGCATCTTGATATAAGCCTTCTTGTATTAGAGAGATTTTTTATTTTCTCTGCGTGGTATGCCCCGTTTATTGTTGTATCATTTTGGGTTTAATATAATTTACATTTTTCTCAAAAAAAAAATAAATATATTTTAGTGTACTAAAATTCTATGGTAAGGTTTTGATTATTTTCATTCGGATTTGTTCTGGACTCATGCAAAGGATTTAGGCCCAACCTAATAATATTACTAAGTTGAAGCACGAGTCAATAAGATCCAATGCACTATATATGGGTTGATATTTTCTATGTGATCCATTTTCAGAGAATTTCTTTTACGCCCTATATATCATGTAAAAACTACTATCAACTTTTTTGTTTTTTATTTATTTATTGAAAATGAGGCTAAAAGCGGGAAATAACTTTGACCCATATCAGCTCTCAAAACATAAGAAATATAATCAGGCGCAAAATCATAAACATGAAACGAAGAATCAACATATAAGGCTTTCTTTGCGAGTTAATCCGTAATTTGATTAGCTTTAATTTCCTTAGGACGTGGTTCCAAATAATAAACTATATAAATAAGTCTAAAGACTTTGAGTATATTCTTTAACTTTTACACTTTTTTGTGTACAAAAAAAAAATTATACTTTTTATCTCTTCATGCAAATAATGACTAAAATGTGGAAATCCTATTCGGGAGAAAATTTTGCTTAAATATTCATCTCCTTTTAGATTCGTGCTGAACATAAATGAAAATTTTTAAAATAAAAAATTATCACTGCCATCAAGCGTGAAAACTTGAAAATTGGTCCTTCAAAGCATAATTAAATAAAAATTATAATATTGAAAAATTAACTCAATTATATATAATTAATTTAAATAACCATTTAATATTCATGCATAAAATTTCATGTTAAAAATTACTACTAATTTTAAAATAACTGCTCGTACTTTTATACCTATTGTTTTGAATTTCTAATTATGAAATTTAGTATATTTAAGCTCATCAAATCCAACCACTAGTTGATATATTAATTAATATTAATGATAATATATTATACTAATAAGAAATAATTGGTAGCTTCTTAGGTAGCATAACAATTGAAAC

>GLYMA.15G125900

AGCTATACCTATATGCCATCAGCTTTTCAGCAGACTTTTGCTGGAAATAACACATATCCCCAATCTCTGGCAGCAGTGCTTCCACAGTATAAAAATAATGTTTCTGCCAGCAGTTTGCCTCAGTCTGCTGCTATTCCTCCTGGATATGGCTTTGGCAGTTCAACAAGCATTCCTGGAGGAAATTTTTCTCTGAATCCACCTGCTGCTCCTACTGGGACAACCATTGGATATGAGGATCTTATAAACTCCCAGTTTAAGGACAATAATCATATGATGTCACTACAGCAGGTACAAAGATCTTGATTTTTAAACTATTTACATTTAACTGGTTTTGTTTTCCTGTTACGATTCTTCTCTTAAAAGTTGCCAATTTGGAAATATCATATGATGATTCAAGTTGAAATGTGATCTGTTAAACTTTCTTTTATTTATTCTTGTTTACAAACCTCAAGTTCACTTTGTTACTATTATGTAACTCTGAATTGTCAGTTTTGGGGGAGGTGGGGTTGAGGAAAGAATATAACATGGAACCTTGTTTACTTTTTTTCTAATGCTGCTTATATGTAAAGCTGAAATGGTTTTATTTGGTGTTTTCTTCTAGAACGAGAATTCTCCCATGTGGGTTCAGGGCCCTGGCTCTCGAACAATGTCTGCAGTTCCTCCCAGCAACTATTATAATTTGCAGGGACAGAATCAACAACAACCAGGTGGATTTCGGCAAAGGCAGCAACAGCAGCAACAGCCTTCACAGCATTTTGGACCCCTTGGGTACCCTAATTTCTACCAATCTCAGACTGGCATTTCGCTGGAACATCAGCCACAAAATCCTCGGGAAGCATCCTTGGGTGGTCCGCAAAGCCAACCATCCAAGCAGTCCCAACAAATATGGCAAAACAGCTACTAATCTTAATCATCTCCCGGTTTTTCAGGGTATTTATGTGAGTTTGAAAGTGGCTAATCAGAGGCATTTACGGTCTAGTAAAAATCATTGTTTGGTCTCTAGAGACCTTGAGCCCATTGCCACGAGGTTGTATTCTACATAATTATGTTGGTCATGTTTAACGTAGGTACTAAGTGTGCTAATTTGGTTTACTCGGTACGTTTTTTGTTTTGTTTTCTAATATTTTCATCCTGATCATAGTGGAAGATCTCTTTTGGGTAACATTCTCACCTGTTATAACTGGTTGCGCTCAATTGTGCTGTGATATACACATTAGAAAGTTCTCCTGAACAATCATGCACGAACAATCATGCACTAAACCTGATTGTAGGGGGTGAGCATCGGTTGGATTCAGCTAAAACATTCAGAATTAACAAATCAAACAAGTAATAATTTAACGACATCATGCATCATTCATTGGGTTGTGGTAGCATCGGGTGCGTATGTGGGTTAATATTTAAGTCCATGTCGGCGGTAGAGTTTGACAATGTTTTTCAACTAAAAAAACACTTTTGATAACTCATCAGTTAGTTACATTGCAAAAATTTCAGTGGTTATTGGCCTCCCGTTCAAAAACTATAACAAAGCATTTCTATAATTTCATTTTAAAGCAATGACATCTTAATGCGTGTTCGTTGTGTTTTTGAATGTGCATTTGCACGACACTGCTCTGGTTCTCTATATTTGATAAATATAAATTTGATTTTTTAAATCGAAAAAAGTGCTTTAAAGCATAAAAATTAAGTATGAAGTTTTAATTTTTGTAATATATAATTAATAAAATTATGATTACAATATTCTTTTATTGCTTACTCATCAGCACATAATGCTTTTCTGATTTAACAAACATTTTTAAAATTGTTAAAACATAAATATTAATATTTTTTGTATTTAATATAGTAAATTGGATAATATTGACAATGCTCAAGTAAATAAAACATAAAGTAATAAATTGGATACAACTACAAGTCTACAACTAACTTCACTAGGTTGAAAATATATGCGGTCATTGTAGAAACAAAAAAGAATAGGTAGAGTGTTCAGTTTCTGACTTTATGTC

>GLYMA.16G003900

CGACTAAAGACTAACTAGTTTAAGTTCTTTTGTGTGTTGAGAGAGATTCCATCTTATTTAACTAATCAATAATACCATACCACATGAAAGTTCATATCAAAATATTCATTTTCCTAAACTTTGAGGGTCTGATTCTTACAAGACAACAATATGAAAGTTTATCAAGAGTAGTAATGAGTAGTTATCTCCCTTAAAACGATTTTCCCCTCTTCAAAAAATGATTCTTTTAATGTCGGAGGGTGGAATAAAATGAAAAAAAAAGTGAATTAAGATGAAAGTTTTGAATTAAAATATAATATAATAATATGGGTCTTATTTATTTCCCTTCAAAGGAATAGACTCTTTAGTTGAGAAAGGTTCTCTCTCCATCTTGGATTATTTTTTTTCCTCTTTATAAAAAAAGTTTATTTTTCTTCCACCCTTTATTTTCTTCCCTCACCATCACCACTTTTCTTCATCACCTTCACCCGACCTTTCTATGTTTGTCTATGCATGACACATTTCGCTTGATTGCTACCCTTTCCCATCTCATTTCTCCTTCCATAAAACATGTAAGAGTTGTCTATTGTAATTATGTCCAAACCATCGAAGTTACTTAGGCAGCATTGCACTGAAGTTAATTTGTGTAGCTTTTGTGTTTATTTTGCTATTTGATGAGCTGATTTGTGTAGCTTTTGCTATTAGACGCGATACCATACATGTGTTTTGGTCTTTTGAAAGATTATGTAGCACTTGAATTATAAAAATGTCAAAAATATATTTTAGTCGTTTTTTGATGTTCTAATTTATTTTTTAGATTTAATTTGATCTTTTAATTTTTTCAAATTCAATTTGATCATTTAATTCTTTAAATTGATAAAATTTGTGGTGGTTTAAAACTATTATAAAATATATATTTGTTGACACCGTGAGTTTATTTTAGACAACAATACCACATTAAATTAATTTTTAAAATTAGAAGACTAAATTTTAAAAAATTAAAAGATCAAATTAAACGTAAATAATAAATTATAAAAAAACTAATTTAACCATATATTATATTTTTTAGTACACAATCAATCATTTTTACATTTTTACATGGAGTAAATAAACATGTTTTTAGATGACTAAAAGCTTCCCCTAAAATAAAAGATGACTGAAAGCTATTTTACAAACTCAACAAATTTGGTTCCCATACTCTTCATACCATTTTCAAAAATTACAGCAAAGCCATCATACTCAACAATAATAAATCGAATGCACAACAATAATGGTGGATAGGTACAACACATGTCGGTTGGGGTGTACTTAGAGCTAATCAATGTCTACATGTTTGTGAATTTTGCTATAATCATCATTTAAAAAATAACATTTTTTGTAACTAGGCTTAACTCACAAAATTGTGTTTAGTATTAATAACAAAAATGGCAATTCATTATTAGTTTATTTGTTGTATGATCCTCTAAAAAAAATAAAAAATGAACGTGATATTTCGTAATTACTAGTAATAAAAATGAAAGCAATAGTGCAATTAAACCCATGAGAAAATACAGGTGCACCAAAAACTTGTTACCTTGTCCCTTTAGCCTTTAGCTGACTCATTTAAATCAAAAGTCCACTTCATTTCACCATGGACGTTACTCAATTAATTACTAAACATAAAAAGCGACGCCGAAAAACAACATAGATTTGATCGACAATTTTGGATCCTAAAATAAGTAAAGACGGCAAAACCATTATGCAGCCGATGATTGTTTTCAGTTTTCACTCGGAATTAATCCGTAAAAATCAATGGAGATAAATTATTATTATTAATAATCTTTTGAAAATAGAATTATGTGTTAATTTAATCAAACTCTATTGATGAATAGTTTATAAGCAAGTTCTTAATAAACCTTAAAACATTCTCTGACCTAATTAGGTCTCTCCAATAAAAAATAAACGTTTATATTTTTCGATGCGAAAATGAATGGTATTGGTTGTGCGAGAGATGACATCGCAACCATAATATATATATTTAT

>GLYMA.16G149500

ATCTCTTTCTCTATTTTATTTTATTTTTAAATGGAGAGGCACGGAAGGCATCTTTTTCTCCTAGTAATCAATAATGTAAAAGCTTTCCAGCGACTATAGCAATCAAGTGTAACATATGATATGGTACTGTGTTGGATGTTACCAAGGAGCTACAGTGAATTTAACGATTTAATCTCTTGAGAACTAGGTAAATACAAGGAAGGTGCTGAATTAATATGATGATTGTCTCAATATTCGGCTACATAAGATCTTGTGGGAATTTTCTTTTCTCTTGGATAAACTTCATGCTAATTTTTTTTATTAGTATAAAAGACTAACAAATGACGTACTTATTCATTGATCATAGAGGCTCTAATACCATGTTTAATTAGGGCCAATATTCAATTAAAACCTAAGTTGATAGTATCAGAAAAATGAGATTGGATACATGTTAGTTCAACTTGATAAAGTCAAACCTCATAATGAGTTCAAAGAAAAAGAAAAAATAAACTATAAAAAAGACTCATATTGATTAAGAAATTATAAAAGATATGAATGAGAGTGTTCAAGCTATATTTATTGAACTTATTAGGCCATCTCTAATAGATGACCTTAAAATAGGATCTTTAAGCTCAGAAAATTGTTCTTTCAAAATTTAGAGGTTAGATTTTTATAAAAGAATCTTAAAAAAAAATTTTAAAATTATTTTTTATAATGAACCTAATCATACACGAATTGTGATCAATATATAAACATTGAAATTTTAAATAATTTACACTATTTGCACAATAATTAAGGTGGTGCACAATCAGGATCAATATATAAATACTGCAATATTATGATATTTTATTATTTAATATTTTTTAAAATATTAAAATAAATTTTGCATTTTTAAATTATTTTTTTAAATTAAAAAATACAAATAACTATTTTTAAACATTTAAAATAAATTGTAAATAAATATTAGAAGGCGAATTTCTTATAAGACTTAAAAAATAGTTTAAGATTTTAAAATAAGATCTATTATTGAAATAAAAAATTAAAAAAAATTTAAAGTAATGTGATATTGCAAGATTTACTAAAAAAAATGATTAAGGTCTCAAAATATGATATTACAGTCTTAGGAATACTCTTAGGAATGCTCTTAGGGAGCCTAAGAATATAACTAACTAATATCCTCTAAAGTAATAGTGTTAGGAGAAACTCATTGACACATTTGACAAATCTTTAATAATGATGACCATATTTACTAAGTACATATACTATTGTCTAATTAGAGTTAAAATTTCACTAGATAATCACATAATTTTTATATATTTTTTTATTATCGGATTGTTTGATCAAGATAGGTCTTAAGAAGAATTAACTCATACACATACCCTTAACCTAAAGACATGGTATTTGGTGAGTTGTGAATCATCCATCGTCTCACCAAGTTATATATATATATATATGTTTAAGCAACACCTAAAAGTTTAAAGTTTGTGAATTATGAATTGTCCGACCAACATTACCTTACTAAGGTCAACTCTGACCGATCAAGAATAATAGATCGACCACAAGTAAGGATGATCCAACTCTAGTTATCTTAGATTATATGCACTTTACGATTTCATTAACGGTTCTAGAATCTCTGACTTTGAGACCTCCAAGTTCTAGACGTGAATAATAGACAATTTTAAAATATTTGACTTTGGAATCTTCGAGTTTTAAACATGAATAATTATACAAATTATTAAAATAAAATTTCAATTCCAAAAAGACTAATTTTTTTATTGAGAAGAGCAAAATACTAAATTAGTACCATATCTAAGTTTTTTCCTTGTAATATTTTCTTACAAGTTAATTTCAACGACGGTGTATAGTGAAATCTTTAATTCTTTCACCTAAACTAAAATTAGAAGACGGCGGTAATAGCGGTCGCTCAAGTCTCACGCTGTTTCGCTTTTAATGACCTCGACCCGACAACTTCCTCTCTCTTCCTTTCTCTCTCTCTCTTTTTTTTTTTTTTTTCCTTTTTT

>GLYMA.17G227100

CTAAGATTGAGTGTTATCGGTGTCACAGGTTTGGCCATTATCGTTCTGAATGTTATACAAAGCTGCCACGCGACAAAGAAAAGGAAGCGATTTCAAAGTTTGTTGAAGAGAATGAAGCTGAAACACTACTGTTCAAAATAGTGAGGAACCAAAATCAGATGTTTGACATGTAGATACAAGGTACAATAATCACATGAGTGGAAGTAAGTCCTCTTTTTTTCATCTAAATGAAGATTTTCATTCAACTGTTAGTTTTGGTGATTCTTCCTCTATAAAAGTAAAGGGTAAAGGTGATATTAAAAAAAAACAAAGAATGGTTTTGTGGAAACAATCTCTAATGTATTGTATGTACTTGACTTAAAAAGTAACTTGTTAAGTGCTAGTCAATTACAAGAGAAGGGATATGTCATTACTATTAAACATGGTGCTTGTGAAATTTATGATTCTACTAGAGGTGTTATTGTTGTTGTACAAATGAGTTCCAATAGGTTGTTTCCATTGAAGATCGAAACTATTCAGTCTTGCTTAGTGGCTGAAATCAAAGATCCTTCATGGTTATGGCATTTTCGTTATGGCCACTTGAGTTTTGGTGGATTAAAGACTCACAATCAGAAGAACATGGTGACAGGACTTCCTCCAATTGCAGCCCCTTCCTAAGTGTGTGAAGATTTTCTCGTTAGCAAACAACATTGTTCTCAATTTCCTAAAAACAAGTCATGGAAAGTAAAAGATGTTCTGGAGTTGGTACATTCAGATATATGCGGGCCAATAAGTCCATCTTCCAATAGAGGCAAAAGTTATTTAATTACCTTCATTGATGATTTTTTAAGGAAAACTTGGATTTATTTTTTGCAGGAAAAATCTGAAGCATTTACTGCATTCAAATTTTTTAAAGCTCGTGTTGAAAATGAGGCTGGAAAAAATATCAAGACCCTTCATATAGATCGTGGTGGTGAATATTGCTCAAATATTTTTGAAAATTTTTGTGTAGAGCATGGCCTACGAAGAGAACTCACAATAGCCTATACATCACAACAAAATGGTGCATCAGAGAGGAAGAATAGGACAATTCTTAACATGGTAAGGAGCTTAAGGACATGAGGAAGGGTTCCAAAGAATTTTTGACCAGAAGCCGTAAATTGGAGTGCTCATATTTTGAATAGAAGCCCAACATTTGTTGTTCAAAACATGACTCCTAAAGAAGCTTGGAGTGGGAGAAAACCATATGTGAATCACTTCAAAAAATTTGGATGCATTGCCTTTGCCCATGTTCCTGATCAGAAAAGAAAGAAACCTGATGACAAAGCTAAAAAATATGTGTTTATTGGTGTAAGTGAAACCTCCAAAGCTTACAAATTGTTTAATCCTAGTACAAAGAAAATTGTGACCAGCAAGGATGTTGTTTTTGATGAAGAAAACACTTGGGATTGGAATAGGCAGCAACCTACCCAAGTATTTCTTGATGAAAATGACGAGAAAGGACAAACTTCAGCCTCTGGTACATCTCAAAGAATTACTCCAACAATAGGTTCAAATTTTAATAAAACATTTAAAAAGTATCAGATACAAGAAACTATTGCCTTAAAAAAGGTTTTTTTATATACAAAAAATAGGTATTTTAAAACCCAAAATAAATATTTTTTTATACAGGTTCAGTATAAACTTCAGTTGTTAATGTATTTCATACATTATTAATATACTAACCAAATGAACAAATGTTGAATAAGAATTAGCAATATATTTGTGTTAATACCTATTCATTTTTTTAATAAGTTGATACTTATTCATTTGAATCAACAATCCTAATGGTTAATATAAGTGATATTAAATTTATATCTCTTAATTAAGTGATTTATTTCTTTATTAATTATTGAGTATCAAATAAATGATATTAAAAATAATATAACTTTGTAATATAATAATAAAAAAGTAGAAGAAAAAAAACACATTCATTTGATTTGATTGTAGACACATAAAATTCATAAATCAAACTCTTCTCT

>GLYMA.18G091200

TTCTGTTAGGATGAATATAATATAAGTCAGTGCACTTGTATTATCGTGTGTTGTTAATAGTTTTTATTTTTTTGGTTCATTGTCTCCCTGATGGATCATGTGCAATAGTGACTGACTATCCAAAATTATATCTGACAGGAAGTGGAGGTTATTCTTACTTGTATGAGCCGCTTTGGTGGGTGGGAATGATAACAAGTGAGTAATACATATTATTCTATTTGGTTTTGGAATGACAAATGAATTGAATGTATTGTTGATTTTTCCTTTCCCAAGTGTACCCCTTGAGCTCCTGCCTTTTTTTATATATATTTTATAGTTATTTTACAAGTGCAGAAAGTAATGTAGTTACTATACTTCTGTAGACTATAGATTGAGTTCTATGTAGAACCTTAACAAATCCAAAGAGGGGCACTCATCTGTATGTTTTCCCCTTCATCCATGTATAATCCATCTTTCTGCTACTTATTATCCAAATTTATGCATTGCACAAAGCTTGTACCTTCATCCTAACTTATTCTTTTATATCCTTTGTTTTGCTTCACATATTTTCCAAGGGAATATCTCAACATGGACACAAAGGGCATATAAAGATGTTATGCTATGAGTTGCATATACTATTAAGTGGATTAAAATTTCTATTATAATTTTGTAACACATTTGCAATTTCAACCGTGCTTATAGGATAAATAAATATTTTTATATAACTAAAATTTATTATGAATGAATTTTTTAATATATATACTATATTTTTAATTTACCATAAAAATAAATTCCATTTTTTATTATTGAAATTTCTTTTAAAAATAAACATGAAAATAGTAAATTGAAAGAGATAAATAAATAGTTAAAAAAAAGTATAAAAAGGAAAAGTGGTTTGAAATAATCGATATATAAATCTTTTGGAATATTAATTAAACCAAATAAAAATTGAAATTAAAATTAAGAAGTATATTTAAAAGAAGAAAATATTAAAAAAATAAAATTAAATGAATATATACAATATAATATATAGATGAAATTCTGTCTTTATCTTATACTCCGATCCAGACAAGTTACAATAATCTCAAACCGCAACATCTATCAGAATTTGTTTATACTTGACAACATGATTATGCTTCTTTCCATTTTTTCCATGACACAAGAATATAATTTAACGACTAACCAAGAACATAATCTTTCTACTGCAGCATTTCTCTTTTGTGCAACTTTGGTAATAACAGGTACTTTTATCCTTATCTTCCACTTCATTCCTCTCTATGTTTTAAAAAAAATGAAAAATTAAAAGTTATTAATACAAATAATTTAATAGTTGAATCTCTTGATCAATCTTGAGTTAGATGTTTAGTTTAAACATAAAGTGATAACTATATCCCACAAACAAATCAATCTTCTTGGTTATACTAAAAATAATATTTTTAAAATGGGTAAAAAGTAATTTATTTATGAAACGTATGCTTGCGTATTCGTCCATAGGTCAAATCGGATATGTAATTATTGGATAAGAGATTTTGATTTCATTTCTTTAAAGTTTTAAAATATATACCATCTATTGTATCCTTTATATATATATATAATAGGATAAATAAATATTTTTATATAACTAAAATTTATTATGAATGAATTTTTTAATATATATACTATATTTTTAAATTACCATAAAAATAAATGCCATTTTTTATTATTGAAATTTCTTTTAAAAATAAAGATGAAAATAGTAAATTGAAAGAGATAAATAAATAGTTAAAAAAAAGTATAAAAAGGAAAAGTGGTTTGAAATAATTGATATATAAATCTTTTGGACTATTAATTAAACCAAATAAAATTTGAAATTAAAATTAAGAAGTATATTTAAAAGAAGCAAATATTAAAAAAATAAAATTAAATGAATATATACAATATAATATATAGATGAAATTTTGTCTTATCTTATACTCCGATCCAGACAAGTTACAATAATCTCAAACCGCAACATCTATCAGAATTTGTTTCTACTTGACAAC

>GLYMA.20G210300

TAATTTTGTGTTTAGAAATTATGTTTTTAAAGTGAGTCTAGAACAAATTAATTTTAATGATAAAATGAAATTACTTTTGAAATATAAAAATAGTTTATGAAAATTTTGTAGGATTCATGTTTAAAAACTAAAACTAATTATATTCACTTTAAAACTTTTACTTCTTTTAAAATTAAATGTACAGAGAAATAATTTTCAGATTTAAAAAAAATACCAAATAAATTGAGTTTCTATTCCACTTAGGACGATATGATCCTTTTATGTCAAAGCAACTACAATCTTGAGCAGAGATAATTAATAATTGGATTCGGGATAACTATAGGAAATAGTAGTTTGTTAATAATGGAATTGATGACAGGGTTGCTTTCAGCATTTCTCAGATTACTTCCTGTATTCTTTTTGAAACACTTTTTTGATTTTTCAGATTAGTCCTTCTAGAAGTAAAAAAAAAAAGTCCAAAATATAATTTTACATTTTGAATTCATCATTCGAAAAGTAAAAGGGAGTGCAAGAAGCAATAGGCAAGCAATAAGGCCCAAATGGCAATCCGAGTTAGTGGGTTGAATGAATAATACATTAGCGAAGCCCCAAATGCATATTGTAGCAGTTCAGGGCTTGCTATCTTTGTCAACAGTTGCGCAGATTGCAATGAGAAAGAATTACTTTTCAGATAACGAGTGTTTAAAATAATAAGTCTAAAGACCCTATTGGTATGACATTTTTGTTTGATTTCAAAAGTCAGAGGGGCTTCTTAATATATATTTCTATAATTTCTGATGACATATGAGCAGGATAATAACACTAGACATAGTACAAACAGAAAGAATTAACGCAGCATTGGTCAGAAATGACCTCAATGAAGTTTTCTTTTGTCTCTGGGTTAACATCCAATCTAAATTAACTTCAAGACTGCAACTTGTTTCTATGACGATAGCTAAGTGATTAGTTAATACACTATTAGCTATAGAAAAATATATATTAAAATAACTTTATAGTTGTTAAACATGCACATATTAATATTATTCCTACAAAAGATCTAAGTCAGTTGGTAAAATAATATGTAAATTGTCGTAAATTTTTTTTTATCTAAATATTAACGGTACGACTGAAAGAGACTTTTGATAGGTAACTTTGTTATTCCGTATTTTTTTATTTGTAACCATTTATGTTTGATTGCCTTCAGAATGTTGTTTTGGGATTTAATTAATGCTATAAATGTTTCTACATTTTTTTATGCAGGTGAGAAAGCATGGCGTTAACGTGTTCTTTGATAAGCTTGGCATTGAGCATGGAAGAATTTTTGTTATTATTATTTCCTTCTGAAAAAATAAGTCTTAAAATAATAATTATTTTAATTTTTTAATATGACATTAATTACATTTTTATTTATATCTTTTATAATATTAATGCAAATACGTGGATTAATAAACGTGAAATTATTCACGTTTAATCAGAGTTTTCAGATTTGAGTGTTGGATGCACACATTTGTATTTATTTGAGAAAAAAAATTGTATCTCACAAATACTTTTTACTGTTTGAATGAGTAAGATATGATTATTATGATAAAATTCTCTCCGAGTTTTTAGTGATTAGTGATTTAATTAATGGAGAAAGTGATGTAATTGATCGAAAAGGGATACTAATAAAGTATGCATAATTCAACATTTATTAATACGATAATACCCGTGAAAAGAGGATAAAGACTCCGAAAGTGAAGTCACTGCTAACTAGTTTTTGATAAAATAATAAAGGAAAAAATCCATGAAACGGCTAAAGTCAAGAAGAAGGATCCGCAGTACAGTGTGTCAGATGGGTCGCCGAAGCACAGGATCAACCACTACCACTCATTTCCCATTTTACCCCTACGTCAATATCGGTGGCGTCACCTCATTGGCCCACTCTGTGGGCCCCCACCTAACAACCCTGAGCCGCCCCGTACTCTATTAACCGCGCACGTTCATATATTTTTGAAGTGTTTCCCTTTGTTGTCCTCATTCTT
