# Supplementary material for: Genome-wide identification and functional characterization of magnesium transporter (MGT) gene family in soybean (Glycine max L.) and their expression profiles in response to aphid infestation, dehydration, and salt stresses
Source: PLoS One. 2025 Aug 29;20(8):e0330440. doi: 10.1371/journal.pone.0330440 (PMC12396710; doi:10.1371/journal.pone.0330440)
Supplement: S13 Data — miRNA targeted prediction of GmMGTs. The miRNA data was downloaded from the plant micro RNA encyclopedia (http://pmiren.com/). (S13 Data.DOCX) [file pone.0330440.s013.docx]

**S13 Data.** miRNA targeted prediction of *GmMGTs*. The miRNA data was downloaded from the plant *micro* RNA encyclopedia (<http://pmiren.com/>).

| **miRNA ID** | **Target ID** | **Target length** | **Target start** | **Target end** | **miRNA_aligned_fragment** |
| --- | --- | --- | --- | --- | --- |
| Gma-miRN1258 | *GLYMA.11G255400* | 22 | 829 | 850 | UCACAAAAUUCCACAACUCCUC |
| Gma-miRN1270 | *GLYMA.02G280800* | 22 | 221 | 242 | AGGACUGCUGGAUCCAAUGCCU |
| Gma-miR10407 | *GLYMA.11G105300* | 24 | 74 | 97 | AGUUAACGGAUGAAUGAAUUUGUC |
| Gma-miR10407 | *GLYMA.12G030100* | 24 | 647 | 670 | AGUUAACGGAUGAAUGAAUUUGUC |
| Gma-miR10441 | *GLYMA.02G068000* | 24 | 341 | 364 | CAACCCUGAGAACAAUGAAAUCGU |
| Gma-miR10441 | *GLYMA.16G149500* | 24 | 341 | 364 | CAACCCUGAGAACAAUGAAAUCGU |
| Gma-miR159h | *GLYMA.06G159100* | 20 | 987 | 1006 | UUGGAUUGAAGGGAGCUCCA |
| Gma-miR169ab | *GLYMA.17G227100* | 21 | 504 | 524 | AAGCCAAGGAUGACUUGCAGG |
| Gma-miR169c | *GLYMA.17G227100* | 21 | 504 | 524 | AAGCCAAGGAUGACUUGCCGA |
| Gma-miR169q | *GLYMA.17G227100* | 21 | 504 | 524 | AAGCCAAGGAUGACUUGCCGA |
| Gma-miR4994 | *GLYMA.14G097400* | 21 | 287 | 307 | UGAUAUCCUUGAGCUAAUACA |
| Gma-miR4994 | *GLYMA.17G227100* | 21 | 269 | 289 | UGAUAUCCUUGAGCUAAUACA |
| Gma-miRN1265 | *GLYMA.15G125900* | 22 | 448 | 469 | UAUUUUCGGAGUCCUGGAGCCU |
| Gma-miRN1281 | *GLYMA.06G005000* | 21 | 557 | 577 | CAAAAUGCUAAUCUGAGGCUC |
| Gma-miRN1351 | *GLYMA.20G210300* | 22 | 729 | 750 | UUAUUUUGUAGCAGCUGAUGGG |
| Gma-miRN1354a | *GLYMA.14G097400* | 21 | 854 | 874 | UUGAAGCAACAAGCAAUGUCA |
| Gma-miRN1354b | *GLYMA.14G097400* | 21 | 854 | 874 | UUGAAGCAACAAGCAAUGUCA |
| Gma-miRN1354c | *GLYMA.14G097400* | 21 | 854 | 874 | UUGAAGCAACAAGCAAUGUCA |
| Gma-miR10188 | *GLYMA.17G227100* | 24 | 545 | 568 | UCUCUGAUUUUGCAGAUAAGGACU |
| Gma-miR10196 | *GLYMA.05G153000* | 22 | 535 | 556 | UGAUUGUGGGAGAGCAUUUCAU |
| Gma-miR10447d | *GLYMA.20G210300* | 22 | 368 | 389 | UUGUUUCCAUGUUGUUGAGUGA |
| Gma-miR1508a | *GLYMA.02G068000* | 21 | 179 | 198 | UAGAAAGGGAAAUAGCAGUUG |
| Gma-miR1508a | *GLYMA.16G149500* | 21 | 179 | 198 | UAGAAAGGGAAAUAGCAGUUG |
| Gma-miR1508b | *GLYMA.02G068000* | 21 | 179 | 198 | UAGAAAGGGAAAUAGCAGUUG |
| Gma-miR1508b | *GLYMA.16G149500* | 21 | 179 | 198 | UAGAAAGGGAAAUAGCAGUUG |
| Gma-miR1513a | *GLYMA.12G030100* | 21 | 749 | 769 | UGAGAGAAAGCCAUGACUUAC |
| Gma-miR1513a | *GLYMA.11G105300* | 21 | 176 | 196 | UGAGAGAAAGCCAUGACUUAC |
| Gma-miR169ab | *GLYMA.14G097400* | 21 | 522 | 542 | AAGCCAAGGAUGACUUGCAGG |
| Gma-miR169c | *GLYMA.14G097400* | 21 | 522 | 542 | AAGCCAAGGAUGACUUGCCGA |
| Gma-miR169p | *GLYMA.17G227100* | 21 | 505 | 525 | UGAGCCAAGGAUGACUUGCCG |
| Gma-miR169p | *GLYMA.14G097400* | 21 | 523 | 543 | UGAGCCAAGGAUGACUUGCCG |
| Gma-miR169q | *GLYMA.14G097400* | 21 | 522 | 542 | AAGCCAAGGAUGACUUGCCGA |
| Gma-miR169x | *GLYMA.10G180200* | 22 | 966 | 987 | UGAGCUAAGGAUGACUUGCCGG |
| Gma-miR171a | *GLYMA.13G368400* | 22 | 729 | 750 | UUGAGCCGUGCCAAUAUCACGA |
| Gma-miR171i | *GLYMA.13G368400* | 22 | 729 | 750 | UUGAGCCGUGCCAAUAUCACGA |
| Gma-miR2119a | *GLYMA.11G255400* | 21 | 234 | 254 | UCAAAGGGAGUUGUAGGGGAA |
| Gma-miR2119b | *GLYMA.11G255400* | 21 | 234 | 254 | UCAAAGGGAGUUGUAGGGGAA |
| Gma-miR395q | *GLYMA.13G368400* | 21 | 371 | 391 | CUGAAGUGUUUUGGGAAACUC |
| Gma-miR4345 | *GLYMA.14G033700* | 24 | 264 | 287 | AAGACGGAACUUACAAAGAUUGUU |
| Gma-miR4345 | *GLYMA.02G280800* | 24 | 264 | 287 | AAGACGGAACUUACAAAGAUUGUU |
| Gma-miR4374a | *GLYMA.02G280800* | 22 | 115 | 136 | CAACACCGUCUUUGAAGCCUGG |
| Gma-miR4374a | *GLYMA.14G033700* | 22 | 115 | 136 | CAACACCGUCUUUGAAGCCUGG |
| Gma-miR4374b | *GLYMA.02G280800* | 21 | 116 | 136 | CAACACCGUCUUUGAAGCCUG |
| Gma-miR4374b | *GLYMA.14G033700* | 21 | 116 | 136 | CAACACCGUCUUUGAAGCCUG |
| Gma-miR4411 | *GLYMA.02G285600* | 22 | 326 | 347 | UUAUUGUAACUAAUUUGUCGGU |
| Gma-miR4412 | *GLYMA.11G105300* | 21 | 338 | 358 | UGUUGCGGGUAUCUUUGCCUC |
| Gma-miR4994 | *GLYMA.06G053100* | 21 | 269 | 289 | UGAUAUCCUUGAGCUAAUACA |
| Gma-miR5780c | *GLYMA.02G068000* | 22 | 847 | 868 | UUAAUAUAUCAGGGACUUGGAA |
| Gma-miR5780c | *GLYMA.16G149500* | 22 | 847 | 868 | UUAAUAUAUCAGGGACUUGGAA |
| Gma-miR9730 | *GLYMA.15G125900* | 22 | 1444 | 1465 | CGAUUGCUGUCAUAACUGCUGC |
| Gma-miR9749 | *GLYMA.05G168200* | 21 | 1325 | 1345 | UUAGCUUCUUUCACCUUUCCC |
| Gma-miR9749 | *GLYMA.08G126600* | 21 | 1322 | 1342 | UUAGCUUCUUUCACCUUUCCC |
| Gma-miR9760 | *GLYMA.05G153000* | 21 | 746 | 766 | UGGAUGAUGUAGUUUUGAUUG |
| Gma-miRN1263 | *GLYMA.12G030100* | 20 | 110 | 129 | GAGUGAGAGAGAGAAAGAGA |
| Gma-miRN1264 | *GLYMA.06G208700* | 21 | 109 | 129 | UGUGGAAUCUUCAAUGGAGUC |
| Gma-miRN1270 | *GLYMA.14G033700* | 22 | 221 | 242 | AGGACUGCUGGAUCCAAUGCCU |
| Gma-miRN1284 | *GLYMA.06G053100* | 22 | 407 | 428 | AGCGAGGUGAUGAUAGCAAUUC |
| Gma-miRN1284 | *GLYMA.14G097400* | 22 | 425 | 446 | AGCGAGGUGAUGAUAGCAAUUC |
| Gma-miRN1284 | *GLYMA.14G097400* | 22 | 1015 | 1036 | AGCGAGGUGAUGAUAGCAAUUC |
| Gma-miRN1284 | *GLYMA.17G227100* | 22 | 997 | 1018 | AGCGAGGUGAUGAUAGCAAUUC |
| Gma-miRN1306 | *GLYMA.08G126600* | 20 | 345 | 364 | AUGUUGUUUUCUCUUUCGUG |
| Gma-miRN1306 | *GLYMA.05G168200* | 20 | 348 | 367 | AUGUUGUUUUCUCUUUCGUG |
| Gma-miRN1307 | *GLYMA.02G117100* | 20 | 923 | 942 | AUCAAUGAUGAAGAAAUGCA |
| Gma-miRN1324 | *GLYMA.10G180200* | 22 | 1106 | 1127 | AGGUACGUCGCCGAAGAAGCUA |
| Gma-miRN1350a | *GLYMA.06G053100* | 22 | 835 | 856 | UGGAACCGACGAGCAAGGACGA |
| Gma-miRN1350a | *GLYMA.17G227100* | 22 | 835 | 856 | UGGAACCGACGAGCAAGGACGA |
| Gma-miRN1350b | *GLYMA.17G227100* | 22 | 835 | 856 | UGGAACCGACGAGCAAGGACGA |
| Gma-miRN1350b | *GLYMA.06G053100* | 22 | 835 | 856 | UGGAACCGACGAGCAAGGACGA |
| Gma-miR10188 | *GLYMA.02G068000* | 24 | 151 | 174 | UCUCUGAUUUUGCAGAUAAGGACU |
| Gma-miR10188 | *GLYMA.14G097400* | 24 | 563 | 586 | UCUCUGAUUUUGCAGAUAAGGACU |
| Gma-miR10193d | *GLYMA.16G003900* | 21 | 556 | 576 | AAAAAAACCAAUGUUAACUGU |
| Gma-miR10193j | *GLYMA.16G003900* | 21 | 556 | 576 | AAAAAAACCAAUGUUAACUGU |
| Gma-miR10193k | *GLYMA.16G003900* | 21 | 556 | 576 | AAAAAAACCAAUGUUAACUGU |
| Gma-miR10193l | *GLYMA.16G003900* | 21 | 556 | 576 | AAAAAAACCAAUGUUAACUGU |
| Gma-miR10405a | *GLYMA.15G125900* | 21 | 1160 | 1180 | UUGUUUCUUAUAAAAAGGACC |
| Gma-miR10405b | *GLYMA.15G125900* | 21 | 1160 | 1180 | UUGUUUCUUAUAAAAAGGACC |
| Gma-miR10405c | *GLYMA.15G125900* | 21 | 1160 | 1180 | UUGUUUCUUAUAAAAAGGACC |
| Gma-miR10405d | *GLYMA.15G125900* | 21 | 1160 | 1180 | UUGUUUCUUAUAAAAAGGACC |
| Gma-miR10405e | *GLYMA.15G125900* | 21 | 1160 | 1180 | UUGUUUCUUAUAAAAAGGACC |
| Gma-miR10421a | *GLYMA.14G097400* | 22 | 101 | 122 | CAGAAUGAGACCUUGAGCGUGG |
| Gma-miR10421b | *GLYMA.14G097400* | 22 | 101 | 122 | CAGAAUGAGACCUUGAGCGUGG |
| Gma-miR10424f | *GLYMA.02G117100* | 21 | 630 | 650 | UUUUCUAAUUUAUCAGGGACU |
| Gma-miR10424g | *GLYMA.02G117100* | 21 | 630 | 650 | UUUUCUAAUUUAUCAGGGACU |
| Gma-miR10424h | *GLYMA.02G117100* | 21 | 630 | 650 | UUUUCUAAUUUAUCAGGGACU |
| Gma-miR10424i | *GLYMA.02G117100* | 21 | 630 | 650 | UUUUCUAAUUUAUCAGGGACU |
| Gma-miR10424j | *GLYMA.02G117100* | 21 | 630 | 650 | UUUUCUAAUUUAUCAGGGACU |
| Gma-miR10424k | *GLYMA.02G117100* | 21 | 630 | 650 | UUUUCUAAUUUAUCAGGGACU |
| Gma-miR1507c | *GLYMA.02G068000* | 22 | 876 | 897 | CCUCAUUCCAAACAUCAUCUAA |
| Gma-miR1510b | *GLYMA.17G227100* | 21 | 370 | 390 | AGGGAUAGGUAAAACAACUAC |
| Gma-miR1510b | *GLYMA.14G097400* | 21 | 388 | 408 | AGGGAUAGGUAAAACAACUAC |
| Gma-miR1511 | *GLYMA.14G033700* | 21 | 114 | 133 | AACCAGGCUCUGAUACCAUGG |
| Gma-miR1513b | *GLYMA.14G033700* | 21 | 638 | 658 | UCAGAGAAAGCCAUGACUUAC |
| Gma-miR1513b | *GLYMA.02G280800* | 21 | 638 | 658 | UCAGAGAAAGCCAUGACUUAC |
| Gma-miR1514a | *GLYMA.09G019600* | 22 | 450 | 471 | UUCAUUUUUAAAAUAGGCAUUG |
| Gma-miR1514b | *GLYMA.09G019600* | 22 | 450 | 471 | UUCAUUUUUAAAAUAGACAUUG |
| Gma-miR1520a | *GLYMA.02G117100* | 22 | 178 | 199 | UGCCACGUGUCAUGUUCUGAUU |
| Gma-miR159a | *GLYMA.06G159100* | 21 | 987 | 1007 | UUUGGAUUGAAGGGAGCUCUA |
| Gma-miR159e | *GLYMA.06G159100* | 21 | 987 | 1007 | UUUGGAUUGAAGGGAGCUCUA |
| Gma-miR165 | *GLYMA.02G285600* | 21 | 681 | 701 | GGAAUGUUGUCUAGCUCGAGG |
| Gma-miR165 | *GLYMA.15G125900* | 21 | 732 | 752 | GGAAUGUUGUCUAGCUCGAGG |
| Gma-miR166l | *GLYMA.02G285600* | 21 | 681 | 701 | GGAAUGUUGUCUGGCUCGAGG |
| Gma-miR166l | *GLYMA.15G125900* | 21 | 732 | 752 | GGAAUGUUGUCUGGCUCGAGG |
| Gma-miR169a | *GLYMA.17G227100* | 21 | 504 | 524 | CAGCCAAGGAUGACUUGCCGG |
| Gma-miR169aa | *GLYMA.17G227100* | 20 | 504 | 523 | AGCCAAGGAUGACUUGCCGG |
| Gma-miR169ab | *GLYMA.02G117100* | 21 | 136 | 156 | AAGCCAAGGAUGACUUGCAGG |
| Gma-miR169b | *GLYMA.17G227100* | 21 | 504 | 524 | CAGCCAAGGAUGACUUGCCGA |
| Gma-miR169d | *GLYMA.17G227100* | 20 | 504 | 523 | AGCCAAGGAUGACUUGCCGG |
| Gma-miR169e | *GLYMA.17G227100* | 20 | 504 | 523 | AGCCAAGGAUGACUUGCCGG |
| Gma-miR169f | *GLYMA.17G227100* | 21 | 504 | 524 | CAGCCAAGGAUGACUUGCCGG |
| Gma-miR169g | *GLYMA.17G227100* | 21 | 504 | 524 | CAGCCAAGGAUGACUUGCCGG |
| Gma-miR169h | *GLYMA.17G227100* | 20 | 504 | 523 | AGCCAAGGAUGACUUGCCGG |
| Gma-miR169j | *GLYMA.14G097400* | 21 | 522 | 542 | UAGCCAAGAAUGACUUGCCGG |
| Gma-miR169k | *GLYMA.14G097400* | 21 | 522 | 542 | CAGCCAAGAAUGACUUGCCGG |
| Gma-miR169l | *GLYMA.14G097400* | 21 | 522 | 542 | CAGCCAAGAAUGACUUGCCGG |
| Gma-miR169m | *GLYMA.17G227100* | 21 | 504 | 524 | CAGCCAAGGAUGACUUGCCGG |
| Gma-miR169s | *GLYMA.17G227100* | 20 | 504 | 523 | AGCCAAGGAUGACUUGCCGG |
| Gma-miR169u | *GLYMA.17G227100* | 20 | 505 | 524 | CAGCCAAGGAUGACUUGCCG |
| Gma-miR169v | *GLYMA.17G227100* | 21 | 504 | 524 | CAGCCAAGGAUGACUUGCCGG |
| Gma-miR169w | *GLYMA.17G227100* | 20 | 504 | 523 | AGCCAAGGAUGACUUGCCGG |
| Gma-miR169x | *GLYMA.02G117100* | 22 | 38 | 59 | UGAGCUAAGGAUGACUUGCCGG |
| Gma-miR169x | *GLYMA.17G227100* | 22 | 504 | 525 | UGAGCUAAGGAUGACUUGCCGG |
| Gma-miR169x | *GLYMA.14G097400* | 22 | 522 | 543 | UGAGCUAAGGAUGACUUGCCGG |
| Gma-miR169y | *GLYMA.17G227100* | 20 | 504 | 523 | AGCCAAGGAUGACUUGCCGG |
| Gma-miR169z | *GLYMA.17G227100* | 20 | 504 | 523 | AGCCAAGGAUGACUUGCCGG |
| Gma-miR2111a | *GLYMA.14G097400* | 21 | 283 | 303 | GUCCUUGGGAUGCAGAUUACG |
| Gma-miR2111a | *GLYMA.17G227100* | 21 | 265 | 285 | GUCCUUGGGAUGCAGAUUACG |
| Gma-miR2111a | *GLYMA.06G053100* | 21 | 265 | 285 | GUCCUUGGGAUGCAGAUUACG |
| Gma-miR2111a | *GLYMA.02G285600* | 21 | 742 | 762 | GUCCUUGGGAUGCAGAUUACG |
| Gma-miR2118a | *GLYMA.05G168200* | 22 | 241 | 262 | UUGCCGAUUCCACCCAUUCCUA |
| Gma-miR2118a | *GLYMA.08G126600* | 22 | 238 | 259 | UUGCCGAUUCCACCCAUUCCUA |
| Gma-miR2118b | *GLYMA.05G168200* | 22 | 241 | 262 | UUGCCGAUUCCACCCAUUCCUA |
| Gma-miR2118b | *GLYMA.08G126600* | 22 | 238 | 259 | UUGCCGAUUCCACCCAUUCCUA |
| Gma-miR319n | *GLYMA.06G053100* | 21 | 767 | 787 | UUUGGACCGAAGGGAGCCCCU |
| Gma-miR390a | *GLYMA.16G003900* | 21 | 70 | 89 | AAGCUCAGGAGGGAUAGCGCC |
| Gma-miR390c | *GLYMA.16G003900* | 21 | 70 | 89 | AAGCUCAGGAGGGAUAGCGCC |
| Gma-miR390e | *GLYMA.16G003900* | 21 | 70 | 89 | AAGCUCAGGAGGGAUAGCGCC |
| Gma-miR390f | *GLYMA.16G003900* | 21 | 70 | 89 | AAGCUCAGGAGGGAUAGCGCC |
| Gma-miR390g | *GLYMA.16G003900* | 21 | 70 | 89 | AAGCUCAGGAGGGAUAGCGCC |
| Gma-miR395n | *GLYMA.15G125900* | 21 | 397 | 417 | CUGAAGUGUUUGGGGGAGCUU |
| Gma-miR395n | *GLYMA.09G019600* | 21 | 397 | 417 | CUGAAGUGUUUGGGGGAGCUU |
| Gma-miR395t | *GLYMA.13G368400* | 21 | 371 | 391 | UUGAAGUGUUUUGGGGAACUC |
| Gma-miR397d | *GLYMA.12G030100* | 21 | 708 | 728 | UCAUUGAGUGUAGCAUUGAUG |
| Gma-miR397d | *GLYMA.11G105300* | 21 | 135 | 155 | UCAUUGAGUGUAGCAUUGAUG |
| Gma-miR4345 | *GLYMA.05G196600* | 24 | 255 | 278 | AAGACGGAACUUACAAAGAUUGUU |
| Gma-miR4387a | *GLYMA.14G033700* | 21 | 65 | 85 | UGUUAGUGAUAAGGCGUGAUG |
| Gma-miR4387a | *GLYMA.02G280800* | 21 | 65 | 85 | UGUUAGUGAUAAGGCGUGAUG |
| Gma-miR4387c | *GLYMA.14G033700* | 21 | 65 | 85 | UGUUAGUGAUAAGGCGUGAUG |
| Gma-miR4387c | *GLYMA.02G280800* | 21 | 65 | 85 | UGUUAGUGAUAAGGCGUGAUG |
| Gma-miR4392a | *GLYMA.15G125900* | 22 | 527 | 548 | UCUGCGAAAAUGUGAUUUCGGA |
| Gma-miR4392b | *GLYMA.15G125900* | 22 | 527 | 548 | UCUGCGAAAAUGUGAUUUCGGA |
| Gma-miR4401a | *GLYMA.02G117100* | 24 | 719 | 742 | UUUGAAAGUAGGCAUUCUAAGACG |
| Gma-miR4411 | *GLYMA.16G149500* | 22 | 304 | 325 | UUAUUGUAACUAAUUUGUCGGU |
| Gma-miR4412 | *GLYMA.16G149500* | 21 | 1139 | 1159 | UGUUGCGGGUAUCUUUGCCUC |
| Gma-miR4412 | *GLYMA.12G030100* | 21 | 917 | 937 | UGUUGCGGGUAUCUUUGCCUC |
| Gma-miR4413a | *GLYMA.20G210300* | 21 | 479 | 499 | UAAGAGAAUUGUAAGUCACUG |
| Gma-miR4413a | *GLYMA.10G180200* | 21 | 473 | 493 | UAAGAGAAUUGUAAGUCACUG |
| Gma-miR4413a | *GLYMA.02G117100* | 21 | 932 | 952 | UAAGAGAAUUGUAAGUCACUG |
| Gma-miR4413b | *GLYMA.20G210300* | 20 | 480 | 499 | UAAGAGAAUUGUAAGUCACU |
| Gma-miR4413b | *GLYMA.10G180200* | 20 | 474 | 493 | UAAGAGAAUUGUAAGUCACU |
| Gma-miR4413b | *GLYMA.02G117100* | 20 | 933 | 952 | UAAGAGAAUUGUAAGUCACU |
| Gma-miR4414 | *GLYMA.12G030100* | 21 | 476 | 496 | AUCCAACGAUGCGGGAGCUGC |
| Gma-miR4998 | *GLYMA.05G196600* | 24 | 120 | 143 | CAGAAGCUGUAGUCACGAAACCCU |
| Gma-miR4998 | *GLYMA.02G117100* | 24 | 1112 | 1135 | CAGAAGCUGUAGUCACGAAACCCU |
| Gma-miR5030 | *GLYMA.04G005200* | 21 | 841 | 861 | UUCCGGAAGAACAAAGCUACC |
| Gma-miR5042 | *GLYMA.06G159100* | 21 | 521 | 541 | UGGGGCUUGAUCCAAGAUAGG |
| Gma-miR5225 | *GLYMA.10G180200* | 22 | 746 | 767 | CCUGUCGUAGGAGAGAUGACGC |
| Gma-miR5377 | *GLYMA.04G005200* | 21 | 838 | 858 | UGAAGGAUCGAUGUAGAAUGC |
| Gma-miR5672 | *GLYMA.11G255400* | 21 | 40 | 60 | CAUGGUAGUGGAAGAAAUGGA |
| Gma-miR5672 | *GLYMA.02G285600* | 21 | 1356 | 1377 | CAUGGU-AGUGGAAGAAAUGGA |
| Gma-miR5678 | *GLYMA.06G053100* | 22 | 144 | 165 | UUCCAUGAUAAGAUCUUUGACU |
| Gma-miR5678 | *GLYMA.09G019600* | 22 | 55 | 76 | UUCCAUGAUAAGAUCUUUGACU |
| Gma-miR5761a | *GLYMA.02G117100* | 21 | 973 | 993 | UUUUGUGUCGUGAAGCUUUUG |
| Gma-miR5761b | *GLYMA.02G117100* | 21 | 973 | 993 | UUUUGUGUCGUGAAGCUUUUG |
| Gma-miR5768 | *GLYMA.16G149500* | 24 | 166 | 189 | AAGUGCAAUACUGAUCUUCGGAAC |
| Gma-miR5768 | *GLYMA.02G068000* | 24 | 166 | 189 | AAGUGCAAUACUGAUCUUCGGAAC |
| Gma-miR5778 | *GLYMA.09G019600* | 22 | 273 | 294 | CGACGAACUCUUCGUCGGCAUC |
| Gma-miR8603 | *GLYMA.10G180200* | 20 | 108 | 127 | ACAUGUCGAACAACAAGCUC |
| Gma-miR9723 | *GLYMA.06G159100* | 22 | 738 | 759 | AGGAGAUUUGGACAACUCAUUU |
| Gma-miR9760 | *GLYMA.14G097400* | 21 | 1000 | 1020 | UGGAUGAUGUAGUUUUGAUUG |
| Gma-miR9760 | *GLYMA.17G227100* | 21 | 982 | 1002 | UGGAUGAUGUAGUUUUGAUUG |
| Gma-miRN1263 | *GLYMA.13G368400* | 20 | 264 | 283 | GAGUGAGAGAGAGAAAGAGA |
| Gma-miRN1269a | *GLYMA.05G196600* | 21 | 382 | 402 | AUGGACAGGACGAGGAGAUCC |
| Gma-miRN1269b | *GLYMA.05G196600* | 21 | 382 | 402 | AUGGACAGGACGAGGAGAUCC |
| Gma-miRN1272 | *GLYMA.02G285600* | 20 | 1096 | 1115 | AGUCCUCGUGUUGCAUCUCU |
| Gma-miRN1283 | *GLYMA.11G255400* | 22 | 284 | 305 | UUUUUUAAUCUGAGCCGGGAAU |
| Gma-miRN1293 | *GLYMA.05G168200* | 22 | 863 | 884 | UUGGAUUUUUGAUUCUGGUGCC |
| Gma-miRN1293 | *GLYMA.08G126600* | 22 | 860 | 881 | UUGGAUUUUUGAUUCUGGUGCC |
| Gma-miRN1314 | *GLYMA.06G159100* | 20 | 621 | 639 | CCAUGGCUGAGGCUGUGACA |
| Gma-miRN1334 | *GLYMA.02G068000* | 21 | 170 | 190 | AGAAGAUUAUGCUGAUUCUGA |
| Gma-miRN1334 | *GLYMA.16G149500* | 21 | 170 | 190 | AGAAGAUUAUGCUGAUUCUGA |
| Gma-miRN1335 | *GLYMA.02G068000* | 22 | 824 | 845 | UCCUGGAAGUAUAGCAGUCUAA |
| Gma-miRN1336 | *GLYMA.02G285600* | 22 | 525 | 546 | AGGAUGCAAAGAGUGAAGAUGU |
| Gma-miRN1338 | *GLYMA.05G153000* | 21 | 743 | 763 | AUGGUGUAGAUGUGGUAGUUA |
| Gma-miRN1346 | *GLYMA.11G255400* | 21 | 844 | 863 | UUGGAUAAUGAUUGUCACGUG |
| Gma-miRN1347 | *GLYMA.02G068000* | 22 | 328 | 349 | UGAGGGCUGAGGCAUAUAGGGG |
| Gma-miRN1354a | *GLYMA.06G159100* | 21 | 236 | 256 | UUGAAGCAACAAGCAAUGUCA |
| Gma-miRN1354b | *GLYMA.06G159100* | 21 | 236 | 256 | UUGAAGCAACAAGCAAUGUCA |
| Gma-miRN1354c | *GLYMA.06G159100* | 21 | 236 | 256 | UUGAAGCAACAAGCAAUGUCA |
| Gma-miRN1360 | *GLYMA.04G005200* | 21 | 839 | 858 | UGAACAGAACAAUGAAGAGAA |
| Gma-miRN1365 | *GLYMA.20G210300* | 21 | 252 | 272 | GUGAUGAUUAUCUGAAGGUUC |
| Gma-miRN1368 | *GLYMA.03G159400* | 20 | 153 | 172 | GAAUAGGAAGAUCUGAUGCC |
